# Impaired remapping of social relationships in older adults

**DOI:** 10.1038/s41598-021-01258-7

**Published:** 2021-11-09

**Authors:** Jan Oltmer, Thomas Wolbers, Esther Kuehn

**Affiliations:** 1 German Center for Neurodegenerative Diseases (DZNE), 39120 Magdeburg, Germany; 2 Department of Radiology, Athinoula A. Martinos Center for Biomedical Imaging, Massachusetts General Hospital and Harvard Medical School, Charlestown, 02129 USA; 3 Institute for Cognitive Neurology and Dementia Research (IKND), Otto-von-Guericke University Magdeburg, 39120 Magdeburg, Germany; 4 Center for Behavioral Brain Sciences (CBBS) Magdeburg, 39120 Magdeburg, Germany

**Keywords:** Cognitive ageing, Empathy, Social behaviour, Cognitive neuroscience

## Abstract

Social relationships are a central aspect of our everyday life, yet our ability to change established social relationships is an under-investigated topic. Here, we use the concept of cognitive mapping to investigate the plasticity of social relationships in younger and older adults. We describe social relationships within a ‘social space’, defined as a two-dimensional grid composed of the axis ‘power’ and ‘affiliation’, and investigate it using a 3D virtual environment with interacting avatars. We show that participants remap dimensions in ‘social space’ when avatars show conflicting behavior compared to consistent behavior and that, while older adults show similar updating behavior than younger adults, they show a distinct reduction in remapping social space. Our data provide first evidence that older adults show more rigid social behavior when avatars change their behavior in the dimensions of power and affiliation, which may explain age-related social behavior differences in everyday life.

## Introduction

Social relationships are a driver of human interaction and satisfaction. Our ability to recognize other people’s social status and emotions, integrate them, and react appropriately to build long-lasting relationships influences personal satisfaction and financial success^1,2^. Human interactions are shaped by constant evaluations of the social counterpart^3–5^. Misjudgments, such as providing too little respect towards a superior person in a social hierarchy, or showing distant behavior towards a close friend, may lead to unfavorable social behavior and related interpersonal consequences. Such consequences could be missed job opportunities, a reduced social network, or mental health problems^6,7^. The evaluation of social counterparts takes place along two major dimensions: *power* (i.e., competence, hierarchical position towards ourselves) and *affiliation* (sympathy, affection)^8,9^. Both dimensions explain a large variance of behavioral patterns in humans and animals where power and affiliation are major orientation cues for social behavior^10,11^.

**Figure 1:**
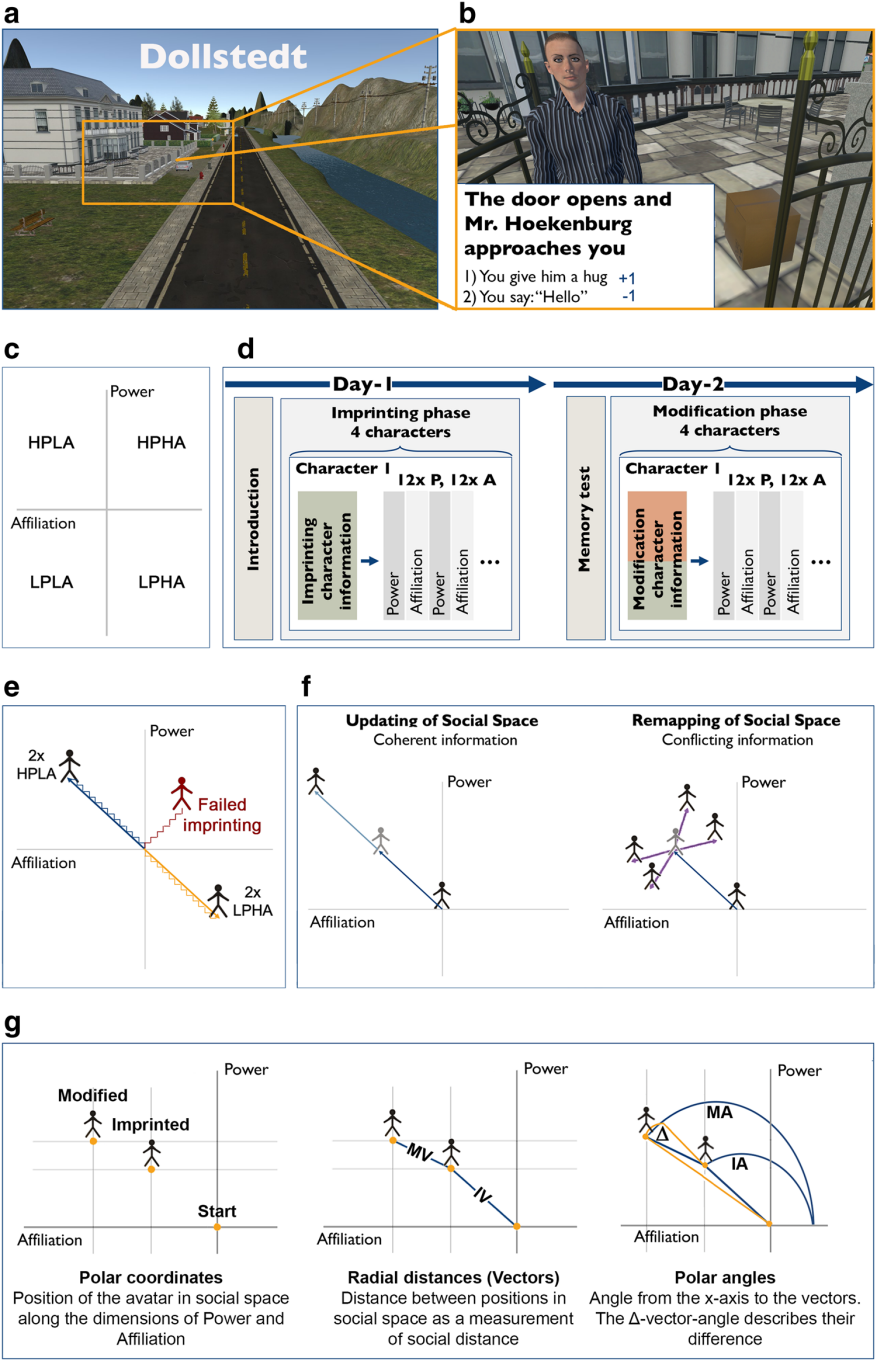
Paradigm and experimental design. (**a**) Scene from the fictional 3D-virtual reality (VR) city ‘Dollstedt’, where participants interacted with avatars. (**b**) Example interaction with an avatar: After a narrative (here: ‘You reach the house of Mr. Hoekenburg and ring the bell’), the participant was confronted with the decision of whether or not to hug Mr. Hoekenburg. Here, choosing 1) would add + 1 to the affiliation towards this avatar, choosing 2) would subtract − 1 of the affiliation towards this avatar. (**c**) Schematic overview of the 4 quadrants of ‘social space’ orthogonalized by the dimensions of power and affiliation. HPLA: high-power/low-affiliation, HPHA: high-power/high-affiliation, LPHA: low-power/high-affiliation, LPLA: low-power/low-affiliation. (**d**) Detailed timeline of the experiments of testing days 1 and 2. P: power, A: affiliation. (**e**) Imprinting-phase: n = 2 avatars were imprinted as HPLA (blue) and n = 2 as LPHA (orange). Participants with non-successful imprinting of at least one avatar were omitted (red). HPLA: high-power/low-affiliation, LPHA: low-power/high-affiliation. (**f**) Modification-phase: n = 1 HPLA- and n = 1 LPHA-avatar showed coherent behavior compared to the imprinting-phase (updating-avatars), whereas n = 1 HPLA- and n = 1 LPHA-avatar showed conflicting behavior compared to the imprinting-phase (remapping-avatars). Different possible scenarios of social remapping are displayed. (**g**) Overview of the dependent variables of interest: Polar coordinates, radial distances (vectors), and polar angles. MV: modification vector, IV: imprinting vector, MA: modification angle, IA: imprinting angle, Δ: delta angle.

Over the long individual life span, financial and social conditions change, but there may also be changes in power and affiliation towards social interaction partners. Within a lifetime, a close friend may become your boss, whereas a respected but unsympathetic stranger may become a fellow friend. Surprisingly little is known about the cognitive mechanisms that underlie such plasticity processes of social relationships in younger and older adults. It has recently been suggested that power and affiliation are encoded in a two-dimensional space into which individual people are ‘mapped’^11^. According to this theory, a two-dimensional ‘social space’ can be categorized into 4 quadrants along the axis of power and affiliation: (1) high-power/low-affiliation (HPLA), (2) high-power/high-affiliation (HPHA), (3) low-power/high-affiliation (LPHA) and (4) low-power/low-affiliation (LPLA), within which social interaction-partners can be categorized (see Fig. 1c). Recent models further assume that cognitive maps are used in various domains of human cognition whenever abstract relationships are encoded, for example object dimensions and scenes^12,13^.

Here, we use the term ‘updating’ to describe the cognitive process of changing relationships towards a social interaction partner based on information on power and affiliation. We also use the term ‘remapping’ to describe the cognitive process of altering the *coordinates* in ‘social space’ based on conflicting information on power and/or affiliation (e.g., a close friend becomes your boss, a stranger becomes your friend). This allows investigating the rigidity of social behavior in a systematic and controlled way.

In social interactions, older adults base their decisions to a lesser extent on novel information about another person’s social behavior than younger adults do^14^. Instead, they rely more on a person’s visual appearance, even if this is in conflict with their actual behavior^15^. Older adults are also biased in categorizing other people’s emotions^16,17^, and present with an age-related positivity effect^16,17^, which may lead to biased social interaction behavior, particularly when the interaction partner changes his/her behavior. Therefore, older adults present an interesting model system to understand basic aspects of the plasticity and rigidity of social interactions, and the influence of emotional biases in this respect. In addition, the investigation of social remapping in older adults allows us to understand fundamental cognitive mechanisms that may underlie altered social behavior in adults.

Here, we introduce a virtual reality (VR) paradigm designed to investigate social interactions and their plasticity in younger and older adults in a real-life context. We developed a virtual town called Dollstedt and investigated the ability of younger and older adults to redefine the ‘position’ of a virtual avatar in ‘social space’ via social interactions in the dimensions of power and affiliation (HPLA, HPHA, LPHA, LPLA, see above). VR allows investigating behavior in a real-life context while offering maximum experimental control^18,19^. We used VR to test the following hypotheses: (i) Do participants modify the position of people in ‘social space’ dependent on the behavior of the avatar (updating (consistent) versus remapping (conflicting))?, (ii) Do older adults show higher affiliation towards avatars compared to younger adults (social bias)?, (iii) Do older adults show less flexible behavior when social interaction partners change their behavior (reduced remapping)? Testing these hypotheses in a virtual environment allows us to gain critical insights regarding the cognitive mechanisms involved in the plasticity and rigidity of social interactions and changes in social behavior that occur with increasing age^20,21^.

## Materials and methods

### Participants

We invited altogether N = 43 participants to the experiment. Whereas participants were invited to two testing days (see below), n = 11 participants were excluded from the final analyses because they did not meet our inclusion criteria at the first testing day (see ‘imprinting-phase’). Participants were healthy, right-handed, fluent in German, and had no history of psychopharmacotherapy, neurological disorders, or psychiatric disorders. Participants were classified into two groups: n = 16 younger adults (mean age: 24.4 ± 3.3 years, 8 male, 8 female; mean ± SD) and n = 16 older adults (mean age: 69.81 ± 6.82 years, 8 male, 8 female: mean ± SD). Older adults successfully underwent a Montreal Cognitive Assessment to screen for Mild Cognitive Impairment (MOCA, score > 26, mean score: 27.94 ± 1.34; mean ± SD).

All participants gave written informed consent and received monetary compensation for taking part. The study was approved by the local ethics committee of the Otto-von-Guericke University Magdeburg (OVGU; Nr. 68/16) and conducted following the regulations of the OVGU Magdeburg and the German Center for Neurodegenerative Diseases (DZNE) Magdeburg.

### Procedure

The experiment consisted of two testing days with one day in between (day-1: imprinting-phase, day-2: modification-phase). On day 1, participants completed session 1 of the experiment (imprinting-phase, see below) and a neuropsychological test battery (Santa Barbara Sense of Direction Scale, Montreal-Cognitive-Assessment for older adults). On day 2, participants first completed a memory test to ensure that they successfully remembered the imprinting-phase and then proceeded to session-2 of the experiment (modification-phase, see below). A detailed timeline of the experimental procedure is provided in Fig. 1d.

### Experimental paradigm

A 3D-VR city called ‘Dollstedt’ was developed and programmed for the present study using the program ‘Unity’ version 5.5 and 3D-models from ‘Sketchup 3D Warehouse’ purpose (Fig. 1a). Dollstedt was composed of 11 houses, 6 streets, and a detailed environment (e.g., trees, moving cars, rivers, and boats). There were 4 avatars with whom each participant interacted, created and animated using the program Adobe Fuse version 1.0. The avatars depended on the participant’s age and gender (older and younger, see below). During the experiment, participants were virtually navigated through Dollstedt using predefined paths and underwent a ‘Question-and-Answer’ (Q + A)-type role-playing game with the avatars. Each of these virtual social interactions was analyzed as one trial, classified either as power-trial, or as affiliation-trial. Accordingly, participants’ response options were either scaled on the dimensions of power or on the dimension of affiliation, dependent on trial-type^22^. The definition of power and affiliation was based on the Stereotype Content Model^8,9^: Power-trials were divided into (1) estimating competence, (2) following/denying of direct orders, and (3) hierarchical behavior, whereas affiliation-trials were divided into (1) warmth of communication, (2) sharing/accepting of private information and (3) allowing/refusing body contact. During the virtual interactions, participants chose either from 2 response options (2-alternative-forced-choice task, imprinting-phase) or from 3 response options (3-alternative-forced-choice task, modification-phase). Each response increased or decreased the power and affiliation-*coordinates* by 1, or kept the dimension neutral (in case of a zero-response during the modification-phase). As an example, Fig. 1b displays a typical affiliation-trial. All trials are provided in the Supplementary Material S5. For more information on avatars and the 3D-VR city, see Supplementary Material S2.

### Imprinting-phase

Before the experiment, participants were informed that they would interact with avatars in a virtual environment (see Supplementary Material S1). The virtual environment was then presented, and participants chose their acting avatar out of 3 gender and age matched options. This was followed by a familiarization-phase in which participants learned the background story of their acting avatar and the other avatars they would be interacting with. In short, it was explained that the acting avatar moved away several years ago, but now travels back to Dollstedt to inherit the house of a deceased aunt (see Supplementary Material S4 for the full story). Afterward, participants were presented the 4 avatars they would be interacting with, and were asked to rate them with respect to power (adjusting the physical distance between participant and avatar) and affiliation (adjusting the avatar's position vertically).

The imprinting-phase was designed to 'position' each of the 4 avatars within his/her designated quadrant, which was predefined as either high-power/low-affiliation (HPLA) or low-power/high-affiliation (LPHA). Note that the avatars behaved in a way to fit into these categories: two avatars were in a powerful position but unfriendly (HPLA), whereas two avatars were in a low-power position but friendly (LPHA). In the virtual interaction trials, participants interacted in a randomized sequence with the gender- and age-matched avatars. Each block of trials started with 'imprinting character information' on the interaction avatar, consisting of self-perception (“How do I remember the avatar?”), perception by others (“What do other people think about the avatar”?) and general information (“What is his/her living situation and job”?). For details on the provided avatar-information, see Supplementary Material S4.

The subsequent 24 trials of one block tested intermittently 12 times power and 12 times affiliation, 4 per definition (i.e., power: (1) estimating competence, (2) following/denying of direct orders, and (3) hierarchical behavior; affiliation: (1) warmth of communication, (2) sharing/accepting of private information and (3) allowing/refusing body contact). Via a 2-alternative-forced-choice task, participants were asked to choose between 2 response options that either increased or decreased the avatar's power- or affiliation-*coordinate* by 1. More precisely, if participants were confronted with an avatar in a typical power-trial, they had the option to either carry the box for the avatar (which would raise the avatar's power-*coordinate* by 1), or to deny carrying it (which would decrease the avatar’s power-*coordinate* by 1)^22^. This is how it was determined whether or not a participant met our inclusion criteria for day 2 (modification-phase): n = 11 participants categorized at least one avatar in the non-intended quadrants (i.e., HPHA or LPLA) (mean amount: 1.36 ± 0.50; mean ± SD). This was, however, expected given the high variability of social interaction profiles^23^. These participants were therefore excluded and n = 32 participants invited to the second day.

This ensured a consistent hierarchical and emotional relationship towards each avatar, established by the background story, and confirmed in the virtual interaction trials. At the end of each block of trials, participants were again asked to rate the interaction avatar with respect to power and affiliation. For a list of trials, see Supplementary Material S5.

### Modification-phase

Only participants who successfully completed the imprinting-phase were invited to the modification-phase (see above). During the modification phase, participants first underwent a memory test. They were presented with 3 power- and 3 affiliation-trials of the imprinting-phase for each avatar (24 trials in total) and were asked to repeat the same way as before. This ensured that all participants memorized the previous testing day.

Participants with an error rate above chance (i.e., above 50%) in 1 avatar or more would have been excluded, which was, however, not the case (error rate younger: 12.24% ± 1.79%; older 12.5% ± 1.48%; mean ± s.e.m.).

Afterward, the background story was presented, specifying that 2 years had passed in Dollstedt after the first interactions, and that the acting avatar decided to visit Dollstedt again to inspect the inherited property (see Supplementary Material S4 for the full story). The acting avatar then again interacted with the 4 avatars. However, before each block of virtual interaction trials, either *consistent* or *conflicting* information on the respective avatar was provided as an experimental intervention (see ‘modification character information’ in Supplementary Material S4). While *consistent* information defined the avatars as part of the *updating-condition* (i.e., same power and affiliation behavior towards the avatar as before), *conflicting information* defined them as part of the *remapping-condition* (i.e., orthogonal power and affiliation behavior towards the avatar). For example, an updating-avatar that was powerful and unfriendly before would show the same behavior again, whereas a remapping-avatar that was powerful and unfriendly before would now be powerless and friendly. Whereas one HPLA and one LPHA avatar showed consistent behavior (updating-avatars), one HPLA and one LPHA avatar showed conflicting behavior (remapping-avatars) (see Fig. 1f). Therefore, one HPLA-avatar was 'remapped' into an LPHA-avatar and one LPHA-avatar was 'remapped' into an HPLA-avatar (n = 2 remapping-avatars), whereas one HPLA-avatar and one LPHA-avatar showed consistent behavior (n = 2 updating-avatars). Each 'modification character information' was followed by 24 trials, intermittently 12 power- and 12 affiliation-trials, 4 per definition (power: (1) estimating competence, (2) following/denying of direct orders, and (3) hierarchical behavior; affiliation: (1) warmth of communication, (2) sharing/accepting of private information and (3) allowing/refusing body contact), and tested using a 3-alternative-forced-choice paradigm. Each response increased or decreased the power and affiliation-*coordinates* by 1, or kept the dimension neutral (in case of a zero-response). At the end of each block of trials, participants were again asked to rate the respecting avatar with respect to power and affiliation. For a full list of trials, see Supplementary Material S5.

### Variables of interest

As outlined above, we here describe social behavior as *coordinates* within a ‘social space’ composed of the axes power (y-axis) and affiliation (x-axis, see Fig. 1e–g). In order to define power- and affiliation-levels, we computed the mean values of the imprinting-phase and the mean values of the modification-phase both for power and affiliation (here referred to as mean power-*coordinates* and mean affiliation-*coordinates*). Given the avatars’ behaviors within one condition were orthogonal (positive in power/negative in affiliation (HPLA), or negative in power/positive in affiliation (LPHA), see Fig. 1e), the *coordinates* were normalized by multiplying the power- and affiliation-*coordinates* of HPLA-avatars with − 1. Otherwise, the expected mean of power-, as well as affiliation-*coordinates* would have been 0 when averaging within one condition.

In addition to *coordinates*, we also computed *radial distances* (*vectors*). *Radial distances* between positions in ‘social space’ were used to quantify social distance taking into account both power and affiliation^22^. Two *vectors* were computed for each condition: The imprinting*-vector* was calculated between the avatar’s starting-point before the experiment started (0/0) and its positions after the imprinting-phase (as seen in Fig. 1g: IV), whereas the modification*-vector* was computed between the avatar’s positions after the imprinting-phase and its position after the modification-phase (as seen in Fig. 1g: MV). Both *vectors* therefore describe the traveled distance in ‘social space’ within one testing day, by taking both power- and affiliation-*coordinates* into account.

Besides *coordinates* and *vectors*, we also computed *polar angles*. *Polar angles* encode power as modulated by affiliation (i.e., they represent the interaction between power and affiliation)^22^. The *polar angle* from the x-axis (affiliation-axis) to the avatar's position after the imprinting-phase is referred to as the imprinting*-angle* (IA). The *polar angle* from the x-axis (affiliation-axis) to the avatar’s position after the modification-phase is referred to as the modification*-angle* (MA). A third angle, the Δ*-angle,* describes the difference between the two angles, and allowed us to compute updating and remapping (Δ-*angle* = imprinting-*angle* (IA) − modification-*angle* (MA), see Fig. 1g). To give an example: If an avatar had a Δ-*angle* that was not significantly different from 0°, it indicated no significant change between imprinting-*angle* and modification-*angle*, which indicates that the avatar was treated as before (here referred to as ‘updating’). On the other hand, a Δ-*angle* that was significantly different from 0° would indicate that the avatar was treated differently in the modification-phase (here referred to as ‘remapping’). For an overview of the different variables of interest, please see Fig. 1g.

### Statistical analysis

Statistical analyses were performed using IBM SPSS version 26 and MathWorks MATLAB version 2019b (CircStat Toolbox for Circular Statistics^24^).

To analyze if participants modify the position of people in ‘social space’ dependent on the behavior of the avatar (updating, remapping; research question i) 3 repeated measures ANOVAs were computed. The first used power-*coordinates* at the end of the imprinting-phase and at the end of the modification-phase as dependent variables (updating and remapping of power), the second used the corresponding affiliation-*coordinates* as dependent variables (updating and remapping of affiliation). The third used the imprinting-*vectors* and modification-*vectors* as dependent variables (traveled distance in ‘social space’). In all three ANOVAs, condition (updating, remapping) and phase (imprinting-phase, modification-phase) were within-subject factors. An alpha level of *p* < .05 was used as a threshold for significance. Post-hoc comparisons were conducted using paired-samples *t*-tests. Further, a post hoc paired *t*-test compared the difference in affiliation-*coordinates* between avatars of different conditions (updating, remapping) in imprinting-phase and modification-phase.

To test if the participants modified the *polar angles* based on condition (updating, remapping), the median Δ-*angles* of the updating- and remapping-avatars were directly compared using a circular one-factor ANOVA (Watson & Williams F-test), using an alpha level of *p* < .05 as a level of significance. We further conducted two nonparametric binomial tests^25,26^ to compare the median Δ-*angles* of the updating- and remapping-avatars to 0°. A significant difference from 0° was used to indicate a remapping of ‘social space’. The alpha level of significance was adjusted for multiple comparisons using Bonferroni and set at *p* < .025. Medians were analyzed because they are not as distorted by outliers as means^27^. multiple Hodges-Ajne Tests^25,26^ were computed to prove a non-uniform distribution of the *polar angles*. Each utilized an alpha level of *p* < .05 as a threshold of significance. Note that *polar angles* were encoded in a two-dimensional *coordinate*-system, which is why the variables required circular analyses.

To test if older adults show higher affiliation towards avatars compared to younger adults (social bias; research question ii), 2 repeated measures ANOVAs with the factors age (young, old), imprinting (HPLA, LPHA), and phase (imprinting-phase, modification-phase) using power-*coordinates* and affiliation-coordinates as dependent variables were computed. Whilst normalized data allows us to compare avatar conditions in absolute position, non-normalized data provides information on the *direction* of position change. In these analyses, data was not normalized and values indicated the direction of the effect.

To reveal age differences in updating and remapping of avatars in ‘social space’ (reduced remapping; research question iii), we conducted 3 repeated measures ANOVAs. 2 used power-*coordinates* as well as affiliation-*coordinates* at the end of the imprinting-phase and at the end of the modification-phase as dependent variables (updating and remapping of power and affiliation). The dependent variables of the third anova were the imprinting-*vectors* and modification-*vectors* (traveled distance in ‘social space’). Each ANOVA utilized age-group (younger, older) as a between-subject factor and condition (updating, remapping) as well as phase (imprinting-phase, modification-phase) as a within-subject factor. Alpha levels of *p* < .05 was used as a threshold for significance. Post-hoc tests based on an alpha level of *p* < .05 were conducted using paired-samples *t*-tests. To analyze if *polar angles* were different between age-groups (younger, older) depending on condition (updating, remapping), Δ-*angles* of younger- and older-adults' updating- and remapping avatars were directly compared using two circular one-factor ANOVAs (Watson & Williams F-tests). The alpha level of significance was adjusted for multiple comparisons using Bonferroni and set at *p* < .025. Further, 2 nonparametric binomial tests were computed, comparing the median Δ-*angle* of younger- as well as older-adults' avatars of each condition (updating, remapping) to 0°. A significant difference from 0° was used to indicate a remapping of ‘social space’. Again, the alpha level of significance was adjusted for multiple comparisons using Bonferroni and set at *p* < .025. Hodges-Ajne Test^25,26^ was used to prove a non-uniform distribution of the *polar angles*.

## Results

### Participants modify the position of people in ‘social space’ dependent on the behavior of the avatar

**Figure 2:**
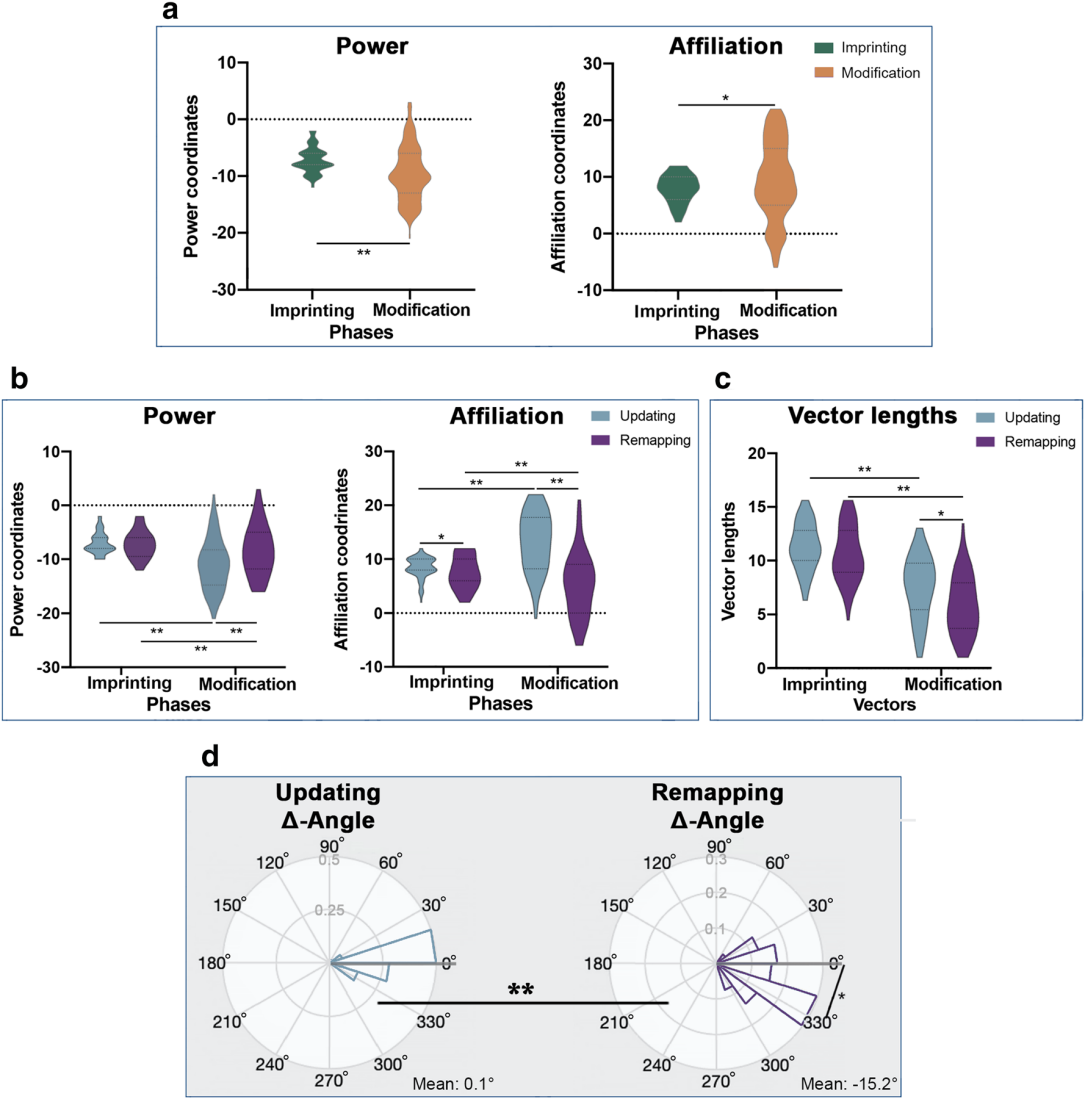
Updating and remapping of ‘social space’ across the group of younger and older adults. (**a**) Main effect of phase in power- and affiliation-coordinates: After modification-phase, avatars had significantly higher absolute power- and higher absolute affiliation-coordinates than after imprinting-phase. Note that the analysis is based on normalized data. (**b**) Interaction between condition and phase in power- and affiliation-coordinates: After the modification-phase, updating-avatars had significantly higher absolute power-coordinates. Affiliation-coordinates were higher for the updating avatars at the imprinting-phase and at the modification-phase, whereas the effect was stronger during the modification-phase. Note that the analysis is based on normalized data. (**c**) Interaction effect of condition and phase in vector lengths: Remapping-avatars show significantly shorter modification-vectors than updating-avatars. (**d**) Difference between conditions: The difference between Δ-angles of conditions was significant. Also, only remapping-avatars’ Δ-angles show a significant difference from 0° (bold grey line).

We first aimed at answering our first research question (i.e., Do participants modify the position of people in ‘social space’ depending on the behavior of the avatar (updating (consistent) versus remapping (conflicting))?) To target this question, we conducted 2 ANOVAs taking the *coordinates* of power and affiliation as dependent variables, with the factors condition (updating, remapping) and phase (imprinting-phase, modification-phase). Both ANOVAs showed a main effect of phase (power: *F*(1,62) = 56.63, *p* < .001, ηp2 = 0.473, day-1: − 7.14 ± 0.24, day-2: − 9.63 ± 0.35, mean ± s.e.m.; affiliation: *F*(1,62) = 7.60, *p* = .008, ηp2 = 0.108, day-1: 7.97 ± 0.20, day-2: 9.25 ± 0.38, mean ± s.e.m.; see Fig. 2a). This shows that independent of condition (updating, remapping), avatars were perceived as higher in absolute power and absolute affiliation at the modification-phase (day 2), compared to the imprinting-phase (day 1). Note that the analysis is based on normalized data.

In addition, both ANOVAs showed a significant interaction between condition and phase (power: *F*(1,62) = 25.15, *p* < .001, ηp2 = 0.285; affiliation: *F*(1,62) = 123.94, *p* < .001, ηp2 = 0.663; see Fig. 2b). Post hoc tests revealed that with respect to power-*coordinates*, this was because absolute power-*coordinates* were significantly higher in updating-avatars compared to remapping-avatars at the modification-phase (*t*(63) = 3.80, *p* < .001; updating: − 10.95 ± 0.48; remapping: − 8.31 ± 0.40; mean ± s.e.m.), but not at the imprinting-phase (*t*(63) = 0.27, *p* = .0789; updating: − 7.19 ± 0.28; remapping: − 7.10 ± 0.24; mean ± s.e.m.). Post hoc tests also revealed that with respect to affiliation-*coordinates*, updating-avatars had significantly higher absolute affiliation-*coordinates* compared to remapping-avatars both at the modification-phase (*t*(63) = 8.96, *p* < .001; updating: 13.25 ± 0.46; remapping: 5.27 ± 0.68; mean ± s.e.m.), and at the imprinting-phase (*t*(63) = 2.017, *p* = .048; day-1 updating: 8.41 ± 0.24; day-1 remapping: 7.53 ± 0.29; mean ± s.e.m.). However, the effect was stronger at the modification-phase and the difference between affiliation-*coordinates* of updating-avatars and remapping-avatars significantly larger in modification-phase in comparison to imprinting-phase (*t*(63) = 11.13, *p* < .001; imprinting: − 0.86 ± 0.43; modification: − 7.98 ± 0.89; mean ± s.e.m.). Together, these results show that the remapping-avatars were treated differently compared to the updating-avatars at the modification-phase (lower power and lower affiliation), whereas there was also an unexpected difference in affiliation between updating-avatars and remapping avatars at the imprinting-phase (lower affiliation for remapping-avatars). Averaged *coordinates* are shown in Fig. 2b.

To test if *radial distances* (i.e., *vectors* in ‘social space’ that take into account both power and affiliation) were modulated by phase and condition, we performed an ANOVA with the factors condition (updating, remapping) and phase (imprinting-phase, remapping-phase using the imprinting-*vector* and the modification-*vector*). We observed a significant main effect of phase (*F*(1,63) = 202.08, *p* < .001, ηp2 = 0.762) and a significant main effect of condition (*F*(1,63) = 8.30, *p* = .005, ηp2 = 0.116). The main effect of phase was found because modification-*vectors* were significantly shorter than imprinting-*vectors* (imprinting-*vectors*: 10.98 ± 0.21; modification-*vectors*: 6.70 ± 0.26; mean ± s.e.m.). The main effect of condition was found because remapping-*vectors* were significantly shorter than updating-*vectors* across phases (updating-*vectors*: 9.42 ± 0.28; remapping-*vectors*: 8.25 ± 0.32; mean ± s.e.m.).

We also found a significant interaction effect between condition and phase (*F*(1,63) = 4.50, *p* = .038, ηp2 = 0.067; see Fig. 2c). This was because the modification-*vector* was significantly longer in updating-avatars compared to remapping-avatars (*t*(63) = 3.13, *p* = .003; updating: 11.30 ± 0.27; remapping 10.66 ± 0.58; updating 7.54 ± 0.36; remapping: 5.84 ± 0.34; mean ± s.e.m.). This is in line with the above-reported results on different *coordinates* between the updating-avatars and remapping-avatars at the remapping-phase, and adds that the traveled two-dimensional distance in ‘social space’ that takes into account power and affiliation was higher in updating-avatars compared to remapping-avatars.

Finally, *polar angles* allow the investigation of the power modulated by affiliation. There was a significant difference in Δ-*angles* between updating- and remapping-conditions (*F*(1,127) = 13.52, *p* = .01; updating: 0.14° ± 14.32°; remapping: − 15.19° ± 26.36°) (Fig. 2d). We used nonparametric binomial tests to compare the median Δ-*angle* of updating- and remapping-avatars to 0°. In addition, we found that the Δ-*angle* of the updating-avatars was not significantly different from 0°, whereas there was a significant difference for remapping-avatars (Fig. 2d) (updating: *p* = .41, 0.14° ± 14.32°, Median = 1.45°; remapping: *p* = .01, − 15.19° ± 26.36°, Median = − 19.41°; Mean ± SD). This analysis indicates greater remapping in the remapping-avatars compared to the updating-avatars. Because post hoc tests revealed that the Δ-*angle* of the updating-avatars was not significantly different from zero, this analysis also indicates that significant remapping only took place in remapping-avatars.

### Older adults show lower affiliation towards avatars

**Figure 3:**
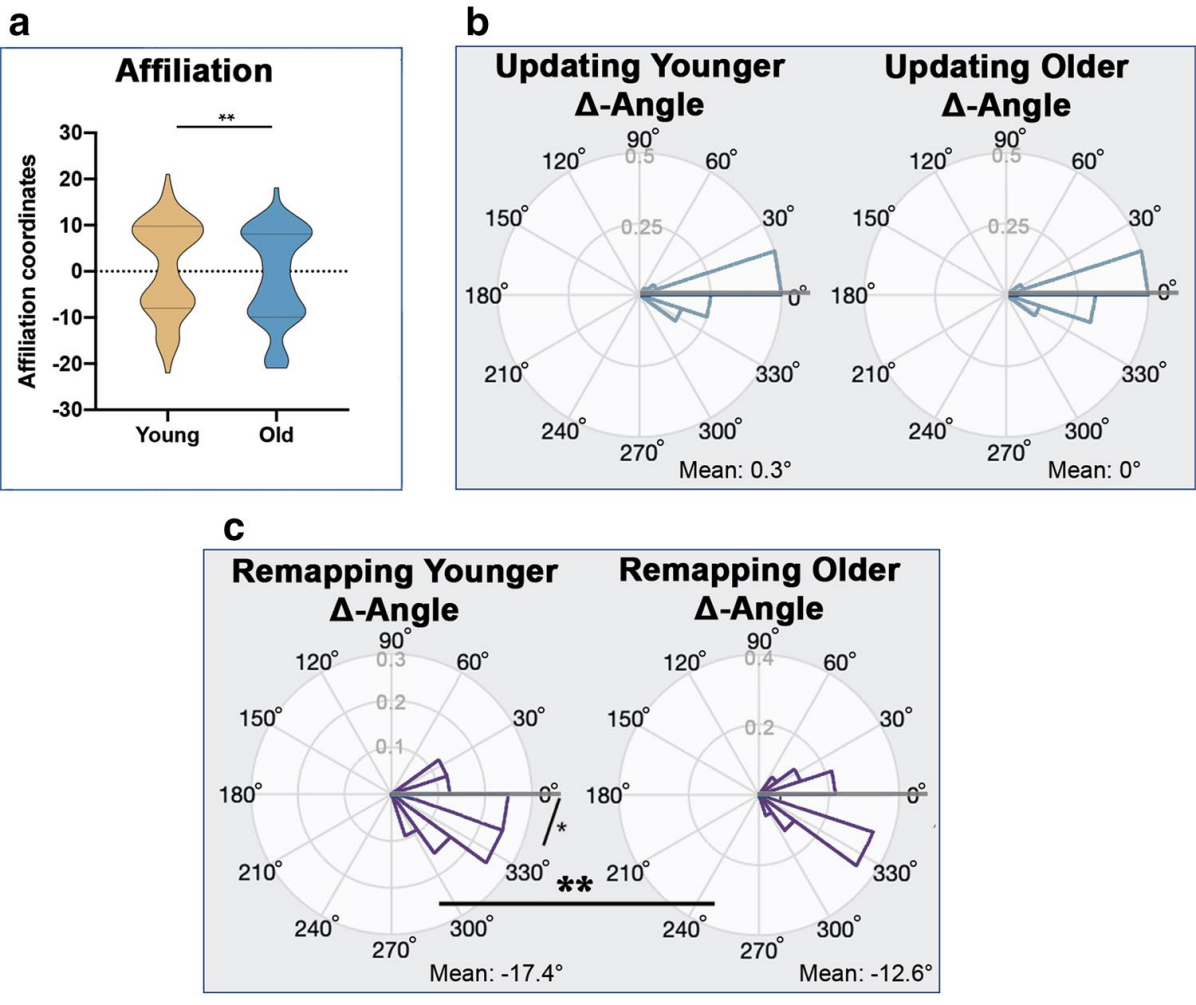
Updating and remapping of ‘social space’ in younger and older adults separately. (**a**) Main effect of age: older adults showed significantly lower affiliation-coordinates than younger adults. (**b**) There was no difference of updating-avatars median Δ-angles between agest. Also, younger and older participants’ median Δ-angles of updating-avatars were not significantly different from 0°. (**c**) The difference of remapping-avatars median Δ-angles between ages was significant. Further, median Δ-angles only significantly differed from 0° in younger adults, not in older adults.

Above, we have provided evidence that our experimental manipulation (i.e., updating vs. remapping) modified participants’ behaviors. With respect to the second research question (i.e., do older adults show higher affiliation towards avatars compared to younger adults (social bias), we computed 2 ANOVAs with the factors age (young, old), imprinting (HPLA, LPHA), and phase (imprinting-phase, modification-phase) using power-*coordinates* and affiliation-*coordinates* as dependent variables. Note that in these analyses, values were not normalized and therefore indicate the direction of the effect. There was no main-effect of age in power-*coordinates* (*F*(1,62) = 0.97, *p* = .756, ηp2 = 0.002), and we observed a main effect of imprinting for power-*coordinates* (*F*(1,62) = 929.94, *p* < .001, 0.937). Compared to LPHA-avatars, HPLA-avatars had significantly higher power-*coordinates* (HPLA: 8.27 ± 0.353; LPHA: − 8.51 ± 0.35; mean ± s.e.m.). However, there was a main effect of age in affiliation-*coordinates* (*F*(1,62) = 5.63, *p* = .021, ηp2 = 0.083; see Fig. 3a): Across imprintings and phases, older adults’ affiliation-*coordinates* were significantly lower than those of younger adults (younger: 0.69 ± 0.88; older: − 1.30 ± 0.89; mean ± s.e.m.). We further observed a main effect of imprinting for affiliation-*coordinates* (*F*(1,62) = 751.99, *p* < .001, ηp2 = 0.924): HPLA-avatars had significantly lower affiliation-*coordinates* than LPHA-avatars (HPLA: − 8.92 ± 0.53; LPHA: − 8.30 ± 0.38; mean ± s.e.m.). There was no interaction-effect between age and phase in power-*coordinates* (*F*(1,62) = 0.06, *p* = .812, ηp2 = 0.001), but there was a trend in affiliation-*coordinates* (*F*(1,62) = 3.13, *p* = .082, ηp2 = 0.048). We did not find interaction-effects between age and imprinting (power: *F*(1,62) = 0.02, *p* = .876, ηp2 = 0.000; affiliation: F(1,62) = 0.00, *p* = .951, ηp2 = 0.000), or age, imprinting and phase (power: *F*(1,62) = 0.27, *p* = .605, ηp2 = 0.004; affiliation: *F*(1,62) = 0.01, *p* = .755, ηp2 = .002).

### Older adults show less remapping of ‘social space’

Above, we have targeted the questions whether our experimental manipulation influenced participants’ social behavior (research question i), and whether there are age-related differences in affiliation towards the avatars (research question ii). To target our final research question (iii, Do older adults show less flexible behavior when social interaction partners change their behavior (reduced remapping)?), we conducted 2 ANOVAs on power-*coordinates* and affiliation-*coordinates* with the factors condition (updating, remapping) and phase (imprinting-phase, remapping-phase) as within-subject factors. Note that here, normalized values had to be used again that do not indicate the direction of the effect. We found no significant main effect of age (power: *F*(1,62) = 0.02, *p* = .896, ηp2 = 0.000; affiliation: *F*(1,62) = 0.00, *p* = .954, ηp2 = 0.000) and no interaction effects between condition and age (power: *F*(1,62) = 0.31, *p* = .579, ηp2 = 0.005; affiliation: *F*(1,62) = 0.62, *p* = .434, ηp2 = 0.108), between phase and age (power: *F*(1,62) = 0.16, *p* = .692, ηp2 = 0.003; affiliation: *F*(1,62) = 0.03, *p* = .856, ηp2 = 0.001) and between condition, phase, and age (power: *F*(1,62) = 0.34, *p* = .908, ηp2 = 0.014; affiliation: *F*(1,62) = 0.31, *p* = .578, ηp2 = 0.005). Power- and affiliation-*coordinates* per se were therefore not differently modulated by conditions between younger and older adults.

Second, we tested for age-related differences in *radial distances (vectors)* by computing an ANOVA that used the imprinting-*vectors* and modification-*vectors* as dependent variables (traveled distance in ‘social space’). Condition (updating, remapping) and phase (imprinting-phase, modification-phase) were within-subject factors, age-group (younger, older) was a between-subject factor. We did not find a main effect of age (*F*(1,62) = 0.09, *p* = .760, ηp2 = 0.002), and we did not find interactions between condition and age (*F*(1,62) = 0.20, *p* = .973, ηp2 = 0.003), between phase and age (*F*(1,62) = 0.307, *p* = .581, ηp2 = 0.005), and between condition, phase, and age (*F*(1,62) = 0.00, *p* = .973, ηp2 = 0.000).

Finally, to reveal differences in remapping of avatars in ‘social space’, Δ-*angles* between age-groups were directly compared using circular one factor ANOVAs (Watson & Williams F-test). We found significantly different Δ-*angles* in the remapping-avatars (*F*(1,63) = 12.04, *p* < .001, younger − 17.44° ± 23.40° (Mean ± SD); older: − 12.85° ± 28.07° (Mean ± SD); see Fig. 3c), but not in the updating-avatars(*F*(1,63) = 0.46, *p* = .50, younger 0.31° ± 0.27° (Mean ± SD); older: − 0.02° ± 0.24° (Mean ± SD); see Fig. 3b). Also, median Δ-*angles* of updating-avatars and remapping-avatars were compared to 0° within each age-group. Δ-*angles* of updating-avatars were not significantly different from 0° both in younger and older adults (younger updating: *p* = .49, 0.31° ± 0.27° (Mean ± SD), Median = 1.52°; older updating: *p* = .73, − 0.02° ± 0.24° (Mean ± SD), Median = 1.45°, see Fig. 3b). However, the Δ-*angle* of remapping-avatars was significantly different from 0° only in younger but not in older participants (younger remapping: *p* = .01, − 17.44° ± 23.40° (Mean ± SD), Median = 17.41°; older remapping: *p* = .38, − 12.85° ± 28.07° (Mean ± SD), Median = − 21.55°, see Fig. 3c).

Taken together, whereas older adults did not show condition-specific differences when looking at each dimension separately (i.e., power*-coordinates* and affiliation*-coordinates*), and also do not show a condition-specific difference in the traveled distance in ‘social space’ (i.e., *radial distances*), they displayed a difference between updating and remapping conditions when the interaction between power and affiliation is taken as a dependent measure (i.e., *polar angles*). This is relevant, because this variable has been discussed to represent the cognitive mapping of social relationships before^22^.

## Discussion

We here investigate social behavior in younger and older adults along the dimensions of power and affiliation using a virtual reality task. During the task, avatars either showed consistent or conflicting behavior over repeated sessions, and virtual interactions with avatars were used to induce real-life social behavior in participants. Social behavior was encoded in a two-dimensional ‘social space’ with the axes of power and affiliation to quantify the amount of updating behavior (behavior towards the avatar after it showed consistent behavior) and remapping behavior (behavior towards the avatar after it showed conflicting behavior) within a systematic coordinate system. Our results show that participants remap the dimensions of ‘social space’ when avatars show conflicting behavior in comparison to consistent behavior. While older and younger adults show similar updating behavior, our data indicate a distinct reduction in remapping ‘social space’ in older participants.

We observed that participants modify the direction of the path on which avatars were moved within a social coordinate system dependent on the avatars’ behavior (conflicting or consistent with previous behavior). This was in accordance with our hypothesis that people modify their social behavior based on the reaction of the counterpart. Our data indicated that the direction of the path on which avatars were moved in ‘social space’ changed significantly between conditions. Avatars displaying consistent behavior showed no change of path direction, while avatars displaying conflicting behavior did. This was expected, because a powerful and unsympathetic avatar that gets even more powerful and shows even more antisocial behavior ‘moved’ towards even higher power levels and even lower affiliation levels. On the other hand, a powerful avatar that lost its status but changed to sympathetic behavior ‘moved’ towards lower power and higher affiliation levels, which explains the change in path direction.

In accordance to this, we found that avatars who displayed consistent behavior across two testing days received a lower degree of position modulation within the dimensions of power and affiliation than those who showed conflicting behavior. Power and affiliation are two dimensions that are major cues for social behavior and influence human and animal behavior^8,10,11^. In a social coordinate system, consistent behavior therefore leads to a constant updating of an avatar’s position (e.g., a powerful avatar gets even more powerful), whereas a change of behavior does not lead to the same degree of position modification (e.g., a powerful avatar who loses its status now remains at the same power position as before). The lower degree of position modulation within ‘social space’ of avatars who showed conflicting behavior could be indicative of a mechanism that reduces the influence of short-term behavioral fluctuations on how we perceive others. More precisely, our results indicate that whereas we constantly update a person’s power and affiliation levels when he/she shows behavior that is consistent with previous behavior, we do not readily change a person’s power and affiliation levels when he/she shows behavior that is conflicting with previous behavior. It also indicates that the further development of an already categorized avatar (here referred to as updating) is easier and faster than the recategorization of an avatar in ‘social space’ (here referred to as remapping). Indeed, social relationships underlie constant evaluations of the counterpart^3–5^, and inappropriate behavior, such as hierarchical misconduct and/or inappropriate display of affection, may lead to unfavorable social behavior and severe interpersonal consequences, like missed job opportunities, a reduced social network, or mental health problems^6,7^. When interaction partners show consistent behavior, a constant updating of social relationships may allow the steady manifestation of the existing categorization concerning the dimensions of social hierarchy (power) and sympathy (affiliation). Remapping of social relationships may be needed in cases of conflicting behavior, which could allow a repositioning of the person within the established social map and would hence enable a behavioral change towards this person^22^.

With respect to the possible neuronal basis of the above described effects, a prior study suggested that social relationships are encoded in a flexible cognitive map (‘social space’) composed of power (hierarchy) and affiliation (sympathy)^22^. This cognitive mapping theory of social relationships was inspired by recent advances in hippocampal research, according to which abstract relationships are encoded in the medial temporal lobe. Similar to categorizing object dimensions^12^ or scenes^12,13^, ‘social navigation’ has also been related to brain networks in which the medial temporal lobe plays a crucial role^22^. The hippocampus and associated networks in the frontal cortex and limbic system may also be involved in mediating plasticity and stability of social hierarchies^22,28^. In addition, analogous to hippocampal place cells, which represent aspects of the physical environment^29,30^, hippocampal social-place-cells have been linked to identifying a person’s position in space^31–33^, which may facilitate spatial navigation^21^. The hippocampal-based cognitive mapping framework was recently supported by a study of Park et al. showing that similar to physical space, social knowledge is encoded as a grid-like representation of a two-dimensional cognitive map mediated by the entorhinal cortex and hippocampus^34^. On the basis of this framework, one may speculate that the medial temporal lobe and associated networks in the frontal cortex and limbic system are responsible for encoding and altering the ‘position’ of a social counterpart within an internal map composed of the axes of power and affiliation.

Based on this framework, one may expect older adults to show particular less remapping of ‘social space’, based on age-related impairments in hippocampal-based spatial and cognitive mapping^35,36^. Within our experimental framework, one may in particular expect older adults to differ in the variable ‘power modulated by affiliation’ because this behavioral variable has been linked to hippocampal-based mapping of social space before^22^. There were no significant differences in power-*coordinates* or affiliation-*coordinates* between age-groups in the different conditions. As expected, we however observed significant differences particularly in the variable ‘power modulated by affiliation’ (referred to as *polar angle* in the results section). We found significant differences in polar angles between age groups, where younger adults showed more social remapping compared to older adults. This led us to hypothesize that the age-related differences we observe may be due to hippocampal-dependent impairments in remapping behavior^37^. We are aware that also other theories might be used to explain this finding, such as age-related differences in emotional control, consistency bias, cognitive flexibility, or internal model update^15^. Previous work also found a positive association between social desirability and age^38^, as well as differences in social norms between ages^39^, which might contribute to the observed differences in interaction behavior between ages.

However, plasticity mechanisms in the medial temporal lobe have been suggested to be particularly vulnerable during aging^35,40^, where grid cell function is compromised in rodents and humans^41^. Also, place-cells display reduced plasticity in older rodents^42,43^ and relate to impaired spatial remapping^24^. Nevertheless, future neuroimaging work will have to clarify which networks are underlying the described behavioral differences. Irrespective of the neuronal basis that may explain our effects, our findings may be relevant to explain increasing behavioral rigidity observed in aging humans^44^ and primates^45^, which could explain differences in social behavior. *Future studies need to address if the observed rigidity in older adults' interaction behavior might be based on a possible mechanism of resisting conflicting information with increasing age.*

Our results further show that older adults rated the avatars generally as less sympathetic (i.e., reduced affiliation) across experimental conditions. This does not corroborate our hypothesis of an age-related positivity effect on the basis of which we would have expected that older adults generally classify the avatars as more sympathetic. Further experiments will have to investigate whether older adults have a tendency towards less affiliative behavior compared to younger adults, or whether this effect is specific for the virtual interaction with avatars, or the specific avatars we chose.

Taken together, our study provides first insights into the cognitive mechanisms that may be involved in the plasticity and rigidity of social interactions in everyday life, and in age-related differences in social behavior. Our results provide evidence for the intriguing hypothesis that older adults show more stable and rigid social behavior due to their reduced remapping behavior when interaction partners show conflicting behavior. Whether this behavioral pattern is adaptive or maladaptive cannot finally be clarified based on our data. On the one hand, this behavioral pattern may prevent older adults from overinterpreting mood changes or short-term fluctuations in other’s behavior, stabilizing their behavioral reaction, and increasing regulation of their emotional state. Older adults have smaller social circles, but overall better social relationships^46^, which might be attributed to passive behavior in problematic social situations in comparison to behavioral changes^47^. On the other hand, it may in certain situations refrain older adults from performing necessary adaptations to their social behavior when environmental conditions change. As an example, older adults tend to base their judgement of trustworthiness less on novel behavioral information about another person’s social behavior than younger adults do, which leads to more financial loss in social interaction games^14^.

While our results provide a novel framework on how social interactions may be classified and investigated, future neuroimaging studies are needed to describe the neuronal mechanisms that underlie the observed behavioral differences in younger and older adults. In particular, they will have to clarify whether hippocampal circuits are involved in these behavioral patterns, and if so to what extent. In addition, it is important to clarify whether the observed changes correlate with hippocampal atrophy or other markers of hippocampal-based mapping deficits. Before interacting, both age groups rated the avatars with respect to power and affiliation. A possible influence of this on the interactions throughout the experiment should be considered in future studies. Also testing larger samples may help to generalize our findings across the aging population, and to detect even more subtle differences between age groups. Finally, we investigated the impact of drastic changes in behavior (e.g., a respected lawyer develops into a poor and disrespected person)—exaggerations that are useful for a first experimental setting, but that are rare in everyday life. Future studies should aim to develop more fine-grained experimental paradigms to investigate the mechanisms that guide everyday behavior.

## Supplementary Information

Supplementary Information.

## Data Availability

The datasets generated during and/or analysed during the current study are available from the corresponding author on reasonable request.
