# Supplementary material for: Impaired remapping of social relationships in older adults
Source: Sci Rep. 2021 Nov 9;11:21910. doi: 10.1038/s41598-021-01258-7 (PMC8578667; doi:10.1038/s41598-021-01258-7)
Supplement: Supplementary file 1 — Supplementary Information. [file 41598_2021_1258_MOESM1_ESM.pdf]

Impaired Remapping of Social Relationships in Older Adults

Jan Oltmer* [1,2,3] Thomas Wolbers [1,4] Esther Kuehn [1,3,4]

[1] German Center for Neurodegenerative Diseases (DZNE), Magdeburg, 30120, Germany

[2] Athinoula A. Martinos Center for Biomedical Imaging, Department of Radiology, Massachusetts General Hospital & Harvard Medical School, Charlestown, 02129, USA

[3] Institute for Cognitive Neurology and Dementia Research (IKND), Otto-von-Guericke University Magdeburg, 39120, Germany

[4] Center for Behavioral Brain Sciences (CBBS) Magdeburg, Magdeburg, 39120, Germany

Supplementary Material - Table of Contents

S1 Instructions **2**

S1.1 Imprinting-Phase *2*

S1.2 Modification-Phase *3*

S1.3 Memory test *4*

S2 Avatars and Virtual-environment **5**

S2.1 Avatars *5*

S2.2 Virtual-environment *5*

S3 Backstory and presentation of the virtual-environment (Dollstedt) **7**

S3.1 Imprinting-phase *7*

S3.2 Modification-Phase *12*

S4 Character information **15**

S4.1 Imprinting-phase *15*
S4.1.1 Lerte (High-Power/Low-Affiliation, Updating-Avatar) 15
S4.1.2 Hoekenburg (High-Power/Low-Affiliation, Remapping-Avatar) 16
S4.1.3 Berger (Low-Power/High-Affiliation, Updating-Avatar) 17
S4.1.4 Karoske (Low-Power/High-Affiliation, Remapping-Avatar) 18

S4.2 Modification-Phase (experimental intervention) *19*
S4.2.1 Lerte (High-Power/Low-Affiliation, Updating-Avatar) 19
S4.2.2 Hoekenburg (High-Power/Low-Affiliation, Remapping-Avatar) 20
S4.2.3 Berger (Low-Power/High-Affiliation, Updating-Avatar) 21
S4.2.4 Karoske (Low-Power/High-Affiliation, Remapping-Avatar) 22

S5 Trials/Interactions **23**

S5.1 Imprinting-phase *23*
S5.1.1 Lerte (High-Power/Low-Affiliation, Updating-Avatar) 23
S5.1.2 Hoekenburg (High-Power/Low-Affiliation, Remapping-Avatar) 30
S5.1.3 Berger (Low-Power/High-Affiliation, Updating-Avatar) 37
S5.1.4 Karoske (Low-Power/High-Affiliation, Remapping-Avatar) 44

S5.2 Modification-Phase *51*
S5.2.1 Lerte (High-Power/Low-Affiliation, Updating-Avatar) 51
S5.2.2 Hoekenburg (High-Power/Low-Affiliation, Remapping-Avatar) 59
S5.2.3 Berger (Low-Power/High-Affiliation, Updating-Avatar) 67
S5.2.4 Karoske (Low-Power/High-Affiliation, Remapping-Avatar) 75

S1 Instructions

The following screenshots display the instructions provided digitally to the participants before Imprinting-Phase (testing day 1), Updating/Remapping-phase (testing day 2) and specifically before the memory test on testing day 2.

S1.1 Imprinting-Phase

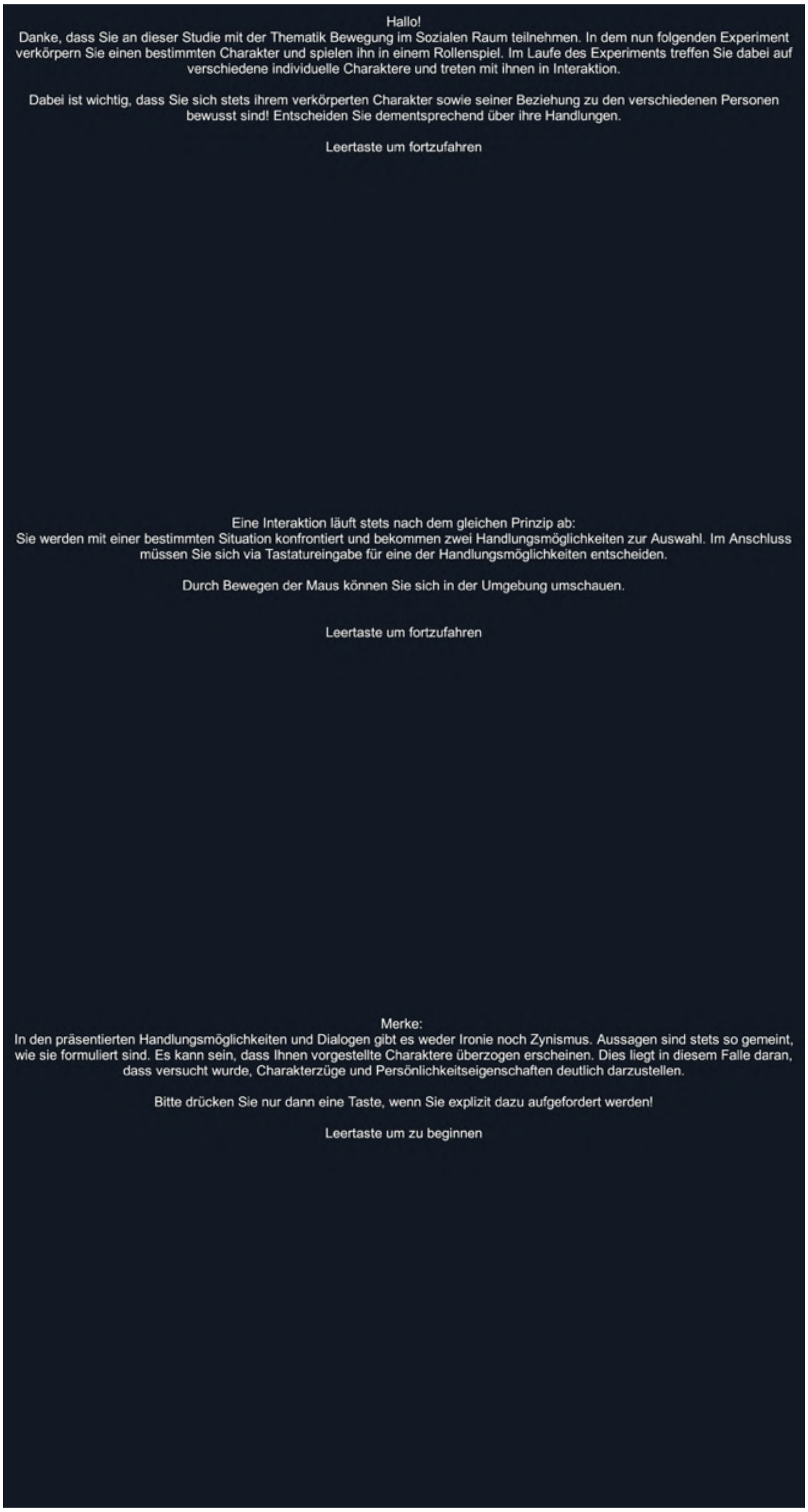

S1.2 Modification-Phase

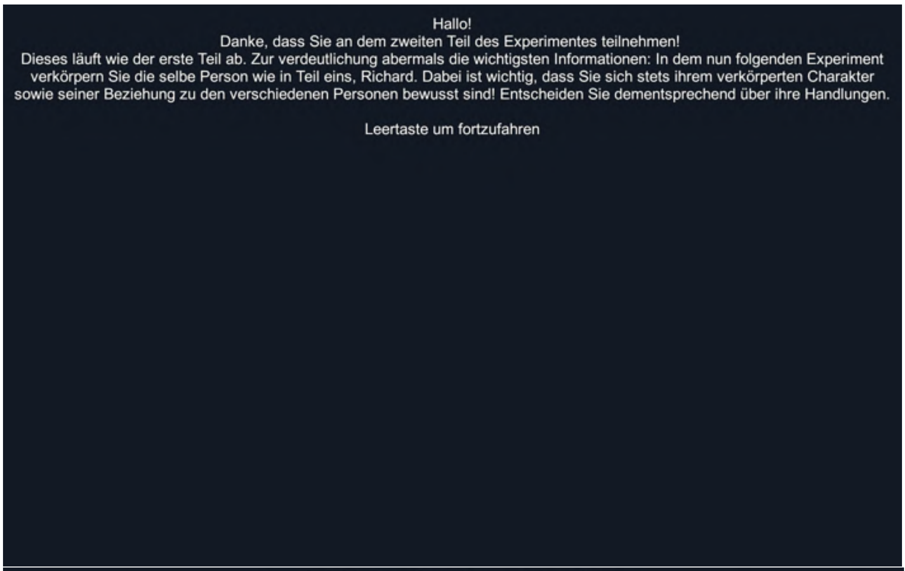

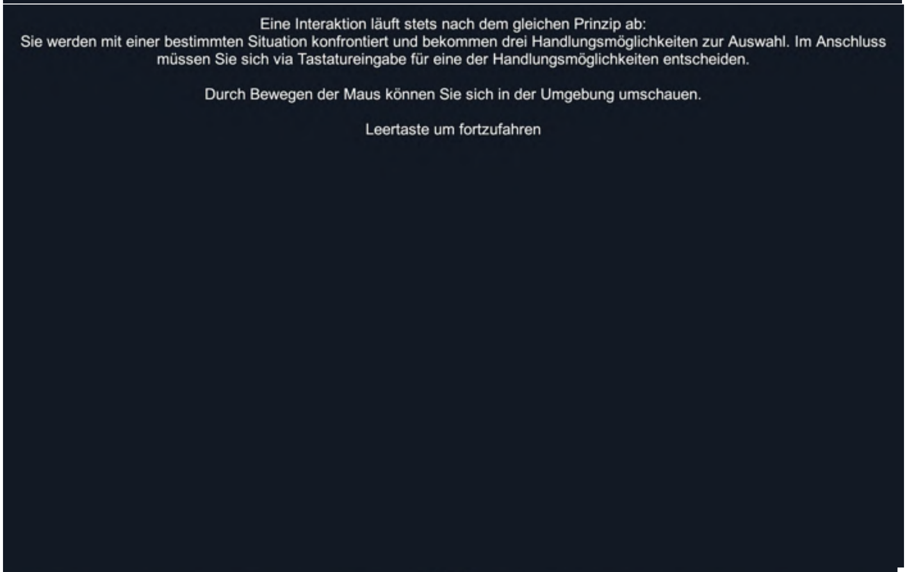

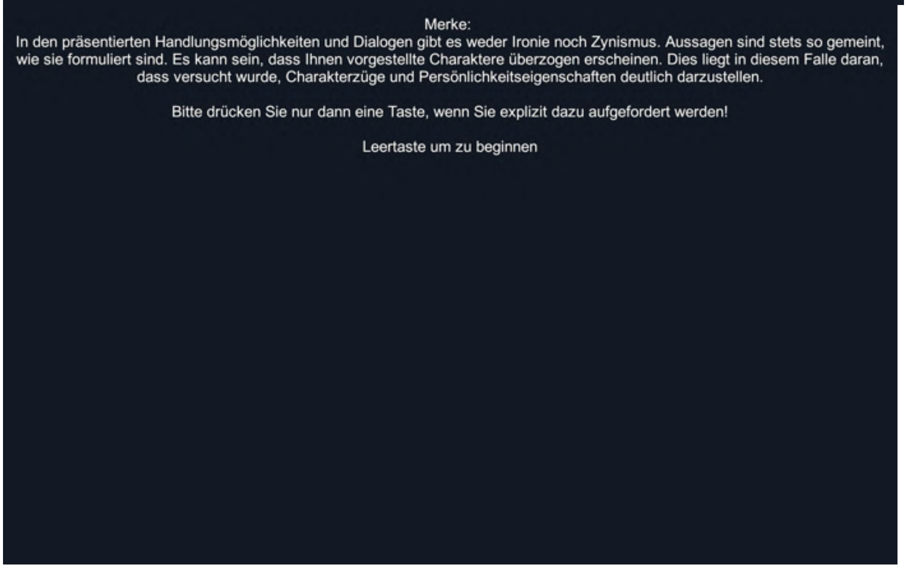

S1.3 Memory test

In dem nun folgenden Teil des Experimentes sehen Sie eine interaktive Zusammenfassung der Ereignisse und Entscheidungen aus dem vorherigen Teil.

Leertaste um fortzufahren

Dabei werden Ihnen die entsprechenden Situationen präsentiert und Ihnen abwechselnd in der rechten oberen Ecke Ihre gewählte Entscheidung oder nichts präsentiert.

Leertaste um fortzufahren

Fühlen Sie sich abermals in die Situation herein und bestätigen Sie Ihre Auswahl aus Phase 1 durch Drücken der entsprechenden Taste sowohl bei eingeblendeter als auch nicht eingeblendeter Entscheidung.

Leertaste um zu beginnen

S2 Avatars and Virtual-environment

S2.1 Avatars

The avatars used for the experiment were manually created and animated using the program *Adobe Fuse, version 1.0* (https://www.adobe.com/de/products/fuse.html). Avatars were matched to the gender and age of the participant. First, one set of young avatars were created for each gender. The corresponding old avatars were created using the same model, but adjusting the 'age' parameter in Adobe Fuse to the maximum. While updating-avatars do not change their appearance from imprinting-phase to updating/remapping-phase, the clothes of remapping-avatars were changed to display a change in social status. As a last step, every avatar was animated, using the 'idle-animation' of Adobe Fuse. As an example, the male young avatars are presented in S4.

S2.2 Virtual-environment

The landscape of the virtual-environment was manually designed using the environment development tools provided by the program *Unity 3D, version 5.5*. (https://unity.com). The 3D-models used for creation of the Virtual-environment were taken from the *Sketchup 3D-warehouse* (https://www.sketchup.com/products/3d-warehouse). A list of the model names is attached. For detailed screenshots of the environment, please see 3.

[2016+train+test]+test+layout+2+++trains+v2.skp
3D_+MAN.skp
3D_+MAN.skp
3D+Man+7.skp
3d+Man+11.skp
3D+People+05.skp
3d+photorealistic+man1.skp
019.skp
46xx4.skp
2002+Tioga+24D.skp
2010+Dodge+Ram+3500.skp
2011+FIAT+DUCATO.skp
2011+Smart+Fortwo+W451.skp
2012+Fiat+Palio.skp
AldiEarth2.skp
Arentsburgh.skp
Arentsburghlaan_1.skp
audi_A4.skp
AutoSave_2104.skp
benzinkanister.skp
Big+Red+Urban+House.skp
bmw.skp
BoxesEtc01.skp
Briefkasten_modern.skp
Burgdorf.skp
bus+2.skp
Busstop.skp
caja_carton.skp
cart+return.skp
Caterpilare+Chargeur.skp
Cats 1.skp
chiesa+bodega+3.skp
chiesa+bodega+3.skp
CMan0011-HD2-O01P06-S.skp
CMan0020-HD2-O01P06-S.skp
CMan0204-HD2-O01P01-S_STD.skp
Component_1-2.skp
Component_1-3.skp
Component_1-4.skp
Component_1.skp
Component_7[1].skp
D45HFR+MBTA.skp
dave2.skp
dave3.skp
de_kat.skp
deri+canta 1.skp
desk+fan.skp
Desk+stuff.skp
doenninghaus4-2.skp
doenninghaus4-2.skp
doenninghaus4-2~.skp
Dog_4p1.skp
Dorfkoppel.skp
Duck-Pintail-Drake[SKU_CFTT1084].skp
Duck-Pintail-Hen[SKU_CFTT1085].skp
Electronics_Black-Desktop-Telephone.skp
Envelope 1.skp
ESCRITORIO+02.skp
F.skp
fan.skp
Fiat+500.skp
File_Kia_Carnival_LX_2011.skp
File_Kia_Carnival_LX_2012.skp
File_Subaru_Forester.skp
flat+screen+tv+2.9.16.skp
forklift.skp
Garden_chair.skp
Gate+and+Fence.skp
GATTO_SORIANO_SDRAIATO_2.skp
Green+House+1.skp
greenhouse.skp
Greenhouse+Unit 1.skp
Group_6.skp
Group_16.skp
Group1_1-2.skp
Group1_1-3.skp
Group1_1-4.skp
Group1_1.skp
Group2_1.skp
Grupo1_1-2.skp
Grupo1_1.skp
GS32ra_V20a.skp
hardenberstr(2).skp
Haus_V07c_nl.skp
haus1big.skp
haus1small.skp
haus2big.skp
haus2small.skp
Hedge+01-01.skp
Hedge+01-05.skp
HM101.skp
HM102.skp
hoekenburg büro.skp
Hoekenburg.skp
Honda+Prelude+Part+1.skp
hotelAzurLjubljana_v1.skp
House-3.skp
House-3.skp
House-4.skp
house+finish.skp
House01.skp

House03.skp
ICE+2.skp
JekPol+Chair.skp
La+Mamy.skp
Lagarto+(Helodermatidae).skp
lao+lambieng.skp
laptop+32.skp
leader+book 1.skp
lerte.skp
lobby.skp
LOLY.skp
LUCAS_C.skp
luke.skp
lux+boat.skp
lux+boat.skp
magazines 1.skp
mailbox 1.skp
mailbox~.skp
Male03_b.skp
maleta 1.skp
ManCassual2.skp
MANKN.skp
Mercedes-Benz+CLS+350+CDI+W218+2012.skp
Mercedes+Benz+SLS+AMG.skp
Milos+-+Yacht34.skp
MINI+Cooper+S+Mk+1+2005.skp
MiniCooper+Low+Poly.skp
mnknnn.skp
Modern+planters.skp
motelroom.skp
MTC+Bus+GE.skp
Mulher+3D+Caminhando+-+Aarquiteta.skp
mutfak+kaplı.skp
Newt.skp
người.skp
Ortsschild_Remshart 1.skp
Park's+Bistro+&+Wine+Bar+Exterior.skp
PC_5thW101.skp
Pens_02.skp
pixel.skp
plattenbau+bahnhof.skp
Porsche+911+Turbo+82+.skp
Postkasten2.skp
Power+yacht.skp
raised+bed.skp
Retail_Shopping-Cart.skp
Road+01.skp
rotary+clothes+line.skp
RS5_V13b.skp
rund+pavillon+-+holz.skp
s15.skp
Sans+titre.skp
SeeSawRaymondPark.skp
Sem+nome.skp
Senza-2.skp
Senza.skp
ShirleyWilliams.skp
Shop+truck,+years+later.skp
skp56F0.skp
skpDA54.skp
Sold sign.skp
Stan.skp
stone+table.skp
Stone+wall+04-01.skp
Stone+wall+04-05.skp
SULO_MGB_120.skp
SULO_MGB_1100.skp
supermarket furnished.skp
supermarket.skp
Teressa.skp
test+train+5.skp
Thomas.skp
tormods+cabin.skp
Tower+Crane.skp
Track+Hoe.skp
tuinvleugel_oost.skp
Turner+Trailer.skp
Unbenannt-3.skp
Unbenannt-4.skp
Unbenannt.skp
Unbenannt.skp
Unna,_Telefonzelle-kamener_Stra_e[2].skp
Untitled 1.skp
Untitled-2.skp
Untitled-2.skp
Untitled-3.skp
Untitled-3.skp
Untitled-3.skp
Untitled-4.skp
Untitled-5.skp
Untitled.skp
vectra_c.skp
VEO+800+MILELE+SE.skp
volvo2.skp
VW+Golf+II+GTI 1.skp
W202+Sport.skp
waste+basket.skp
WindmuehleEtelsen_V02a.skp
WoodenJetty.skp
WoodenJetty.skp
WoodenJettyWhite.skp
YBCVTPOEPLE#8.skp
YBCVTPOEPLE#10.skp
YBCVTPOEPLE#21.skp
YBCVTPOEPLE#23.skp
YBCVTPOEPLE#31.skp
ZBpM+-+Beetle+Convertible.skp

S3 Backstory and presentation of the virtual-environment (Dollstedt)

At the beginning of the Imprinting-Phase, the backstory of the impersonated avatar was presented in an interactive way, allowing the participants to get used to the controls of the experiment. The participants impersonate a person that inherited a house in it's hometown called 'Dollstedt' and decides to travel there. On arrival, the virtual environment ('Dollstedt'), was presented.

S3.1 Imprinting-phase

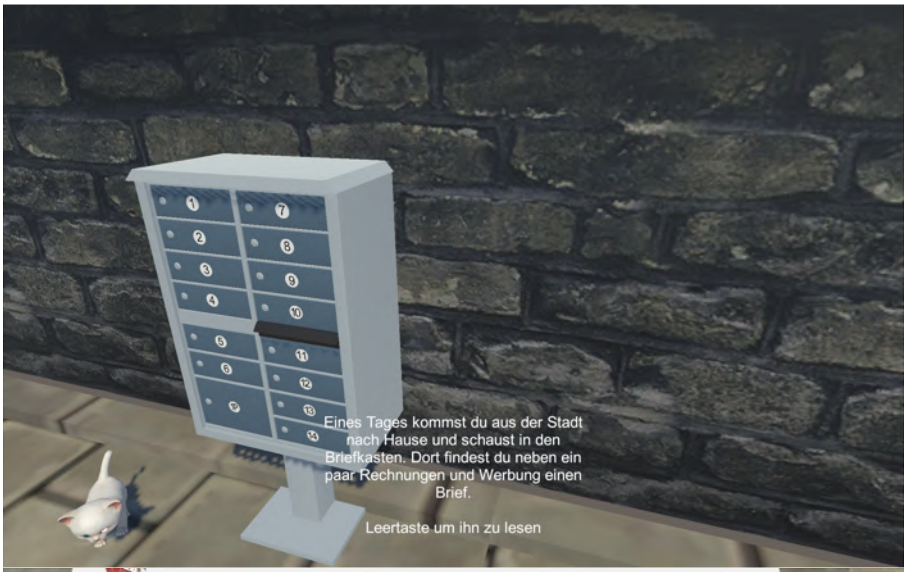

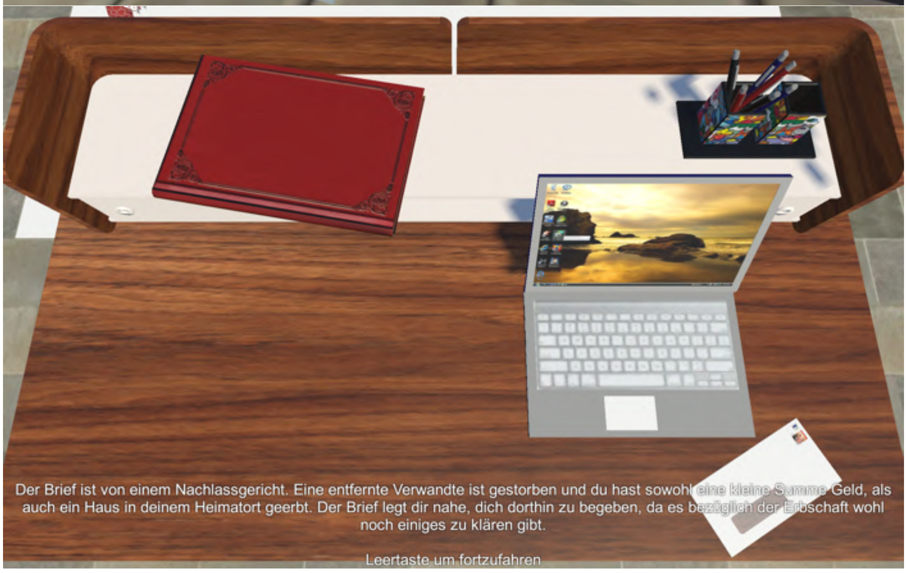

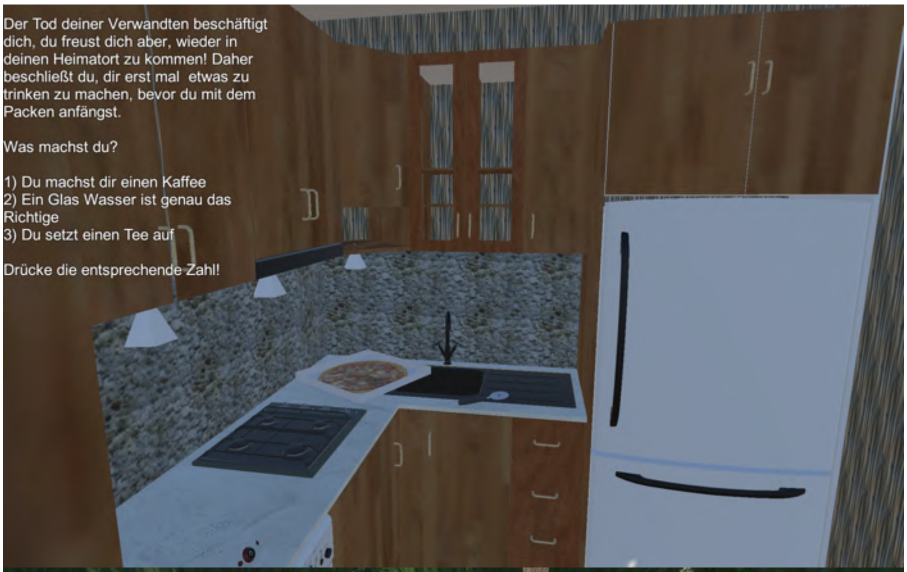
Der Tod deiner Verwandten beschäftigt dich, du freust dich aber, wieder in deinen Heimatort zu kommen! Daher beschließt du, dir erst mal etwas zu trinken zu machen, bevor du mit dem Packen anfängst.
Was machst du?
1) Du machst dir einen Kaffee
2) Ein Glas Wasser ist genau das Richtige
3) Du setzt einen Tee auf
Drücke die entsprechende Zahl!

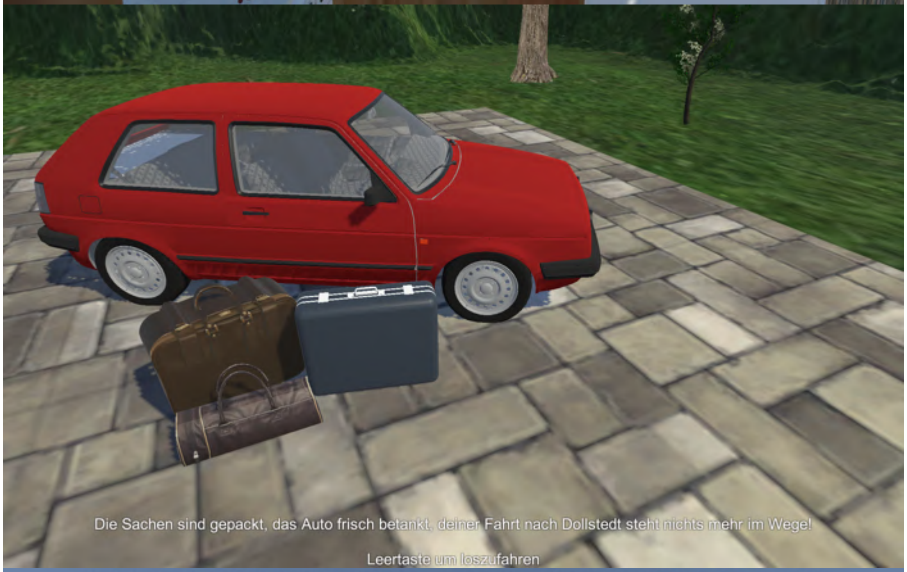
Die Sachen sind gepackt, das Auto frisch betankt, deiner Fahrt nach Dollstedt steht nichts mehr im Wege!
Leertaste um loszufahren

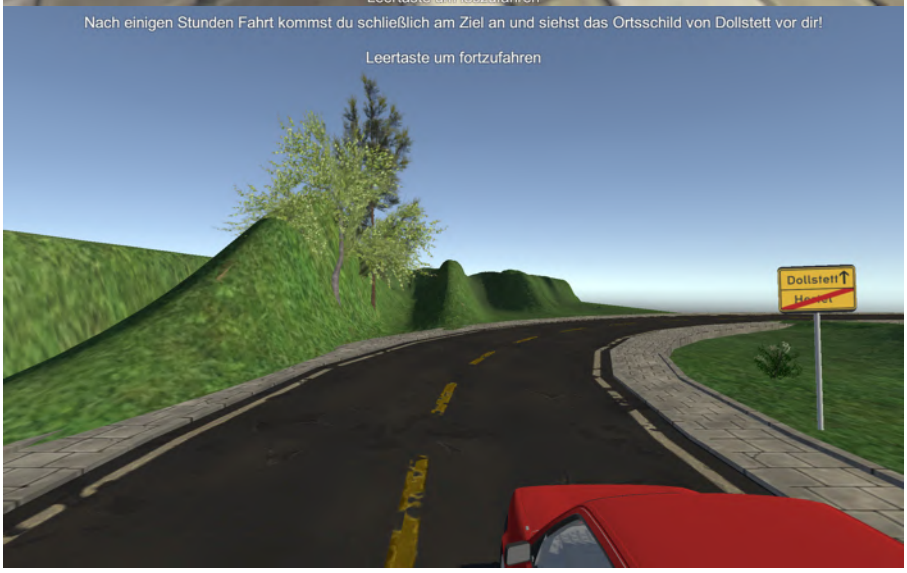
Nach einigen Stunden Fahrt kommst du schließlich am Ziel an und siehst das Ortsschild von Dollstett vor dir!
Leertaste um fortzufahren
Dollstett

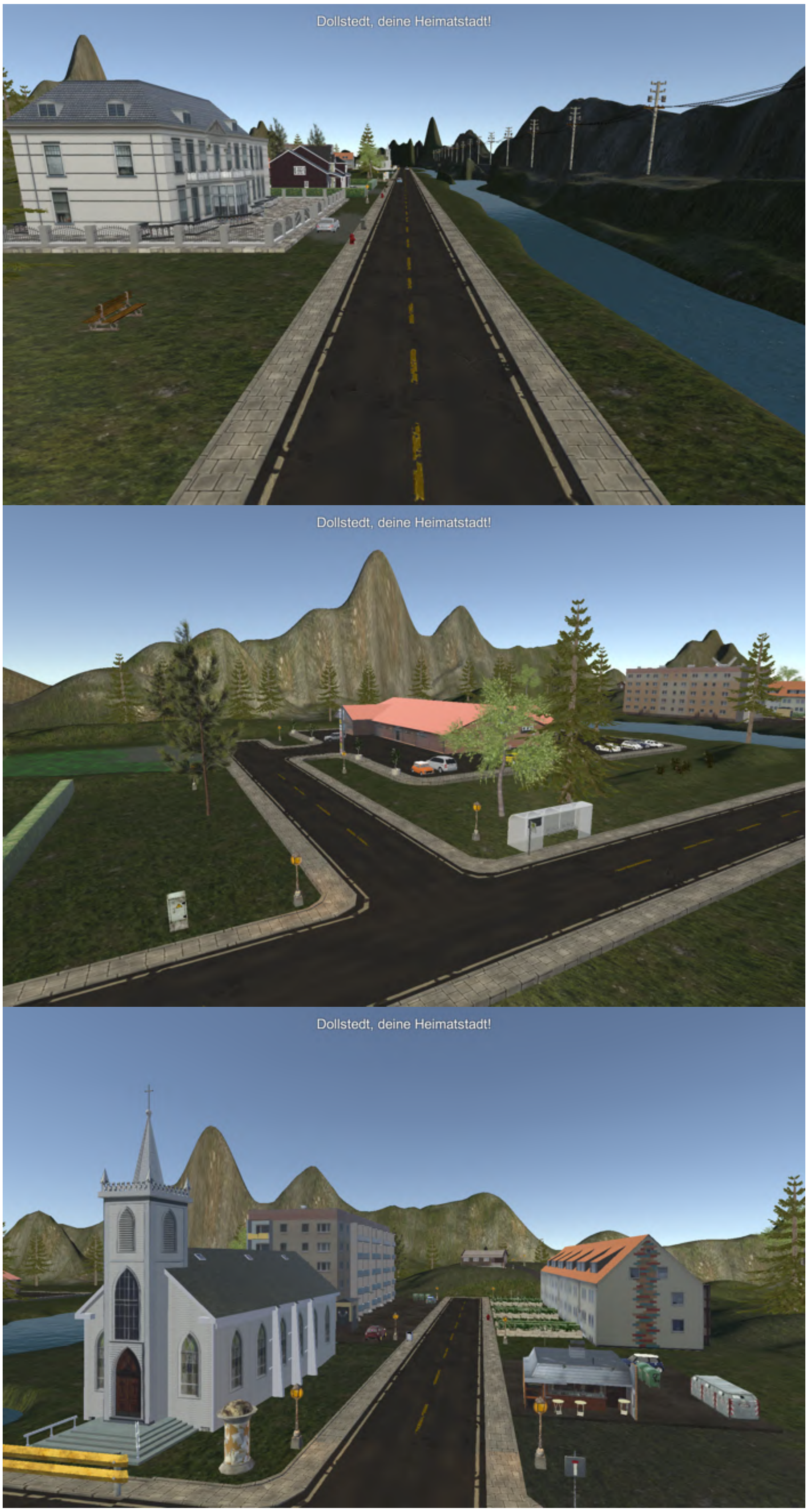
Dollstedt, deine Heimatstadt!
Dollstedt, deine Heimatstadt!
Dollstedt, deine Heimatstadt!

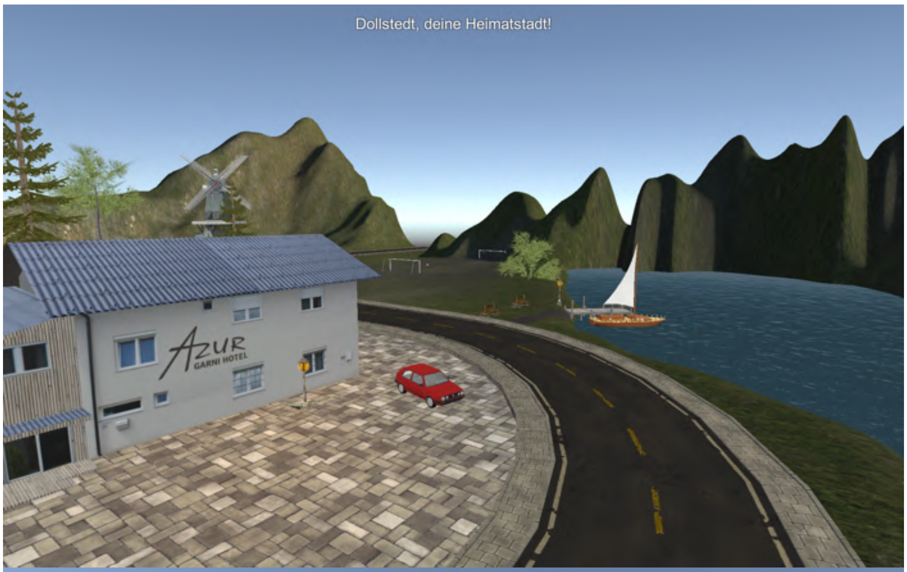
Dollstedt, deine Heimatstadt!
Azur
GARNI HOTEL

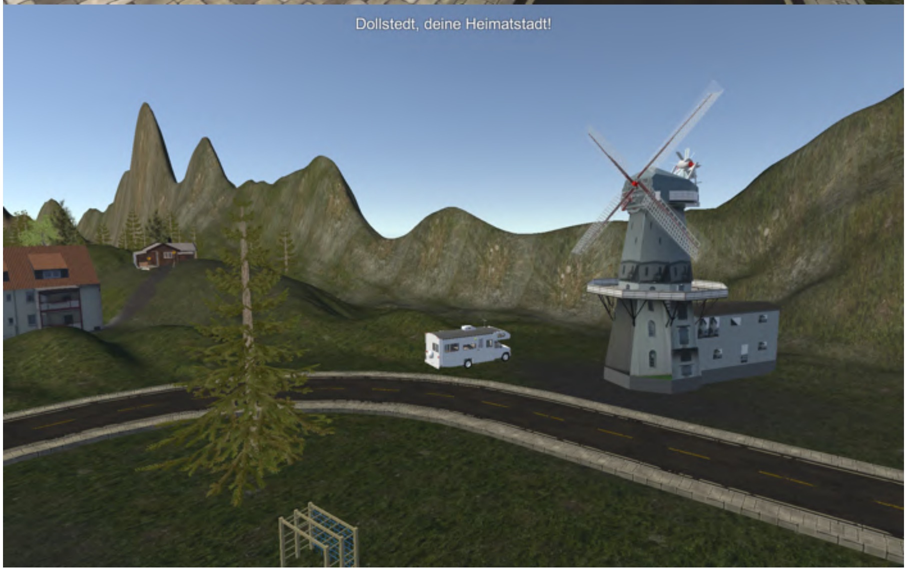
Dollstedt, deine Heimatstadt!

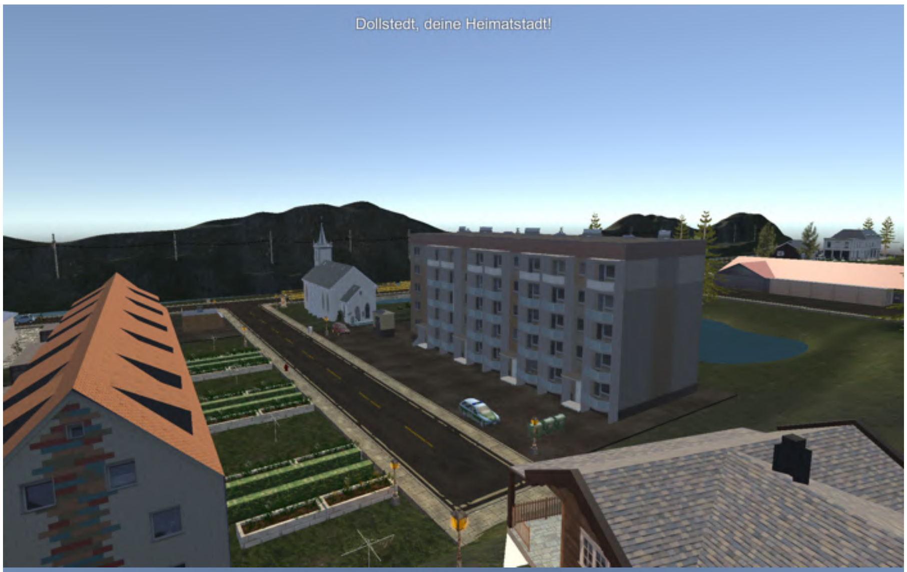
Dollstedt, deine Heimatstadt!

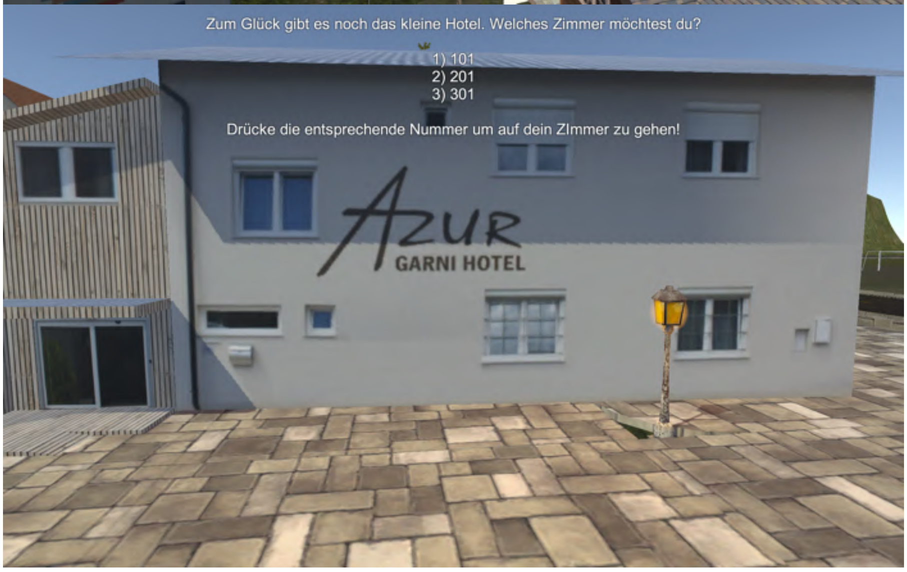
Zum Glück gibt es noch das kleine Hotel. Welches Zimmer möchtest du?
1) 101
2) 201
3) 301
Drücke die entsprechende Nummer um auf dein ZImmer zu gehen!
AZUR
GARNI HOTEL

S3.2 Modification-Phase

At the beginning of the Modification-Phase, the backstory of testing-day-2 was provided: Two years have passed and the avatar decided to travel back to Dollstedt. On arrival, changes of the virtual environment were presented. Changes of the virtual environment resembled changes of the interaction avatars (e.g. a high-power/low-affiliation avatar was modified to low-power/high-affiliation in Modification-Phase and had to sell his/her villa).

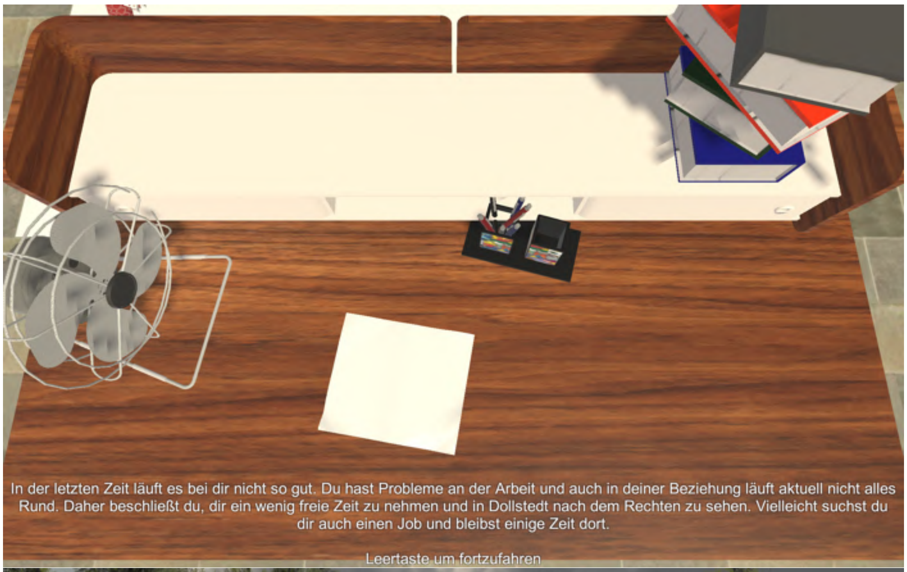

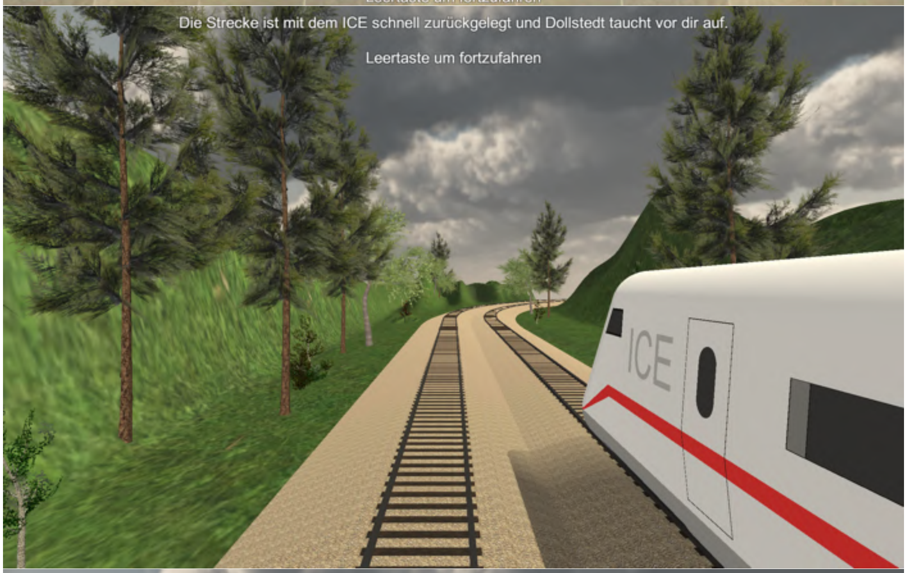

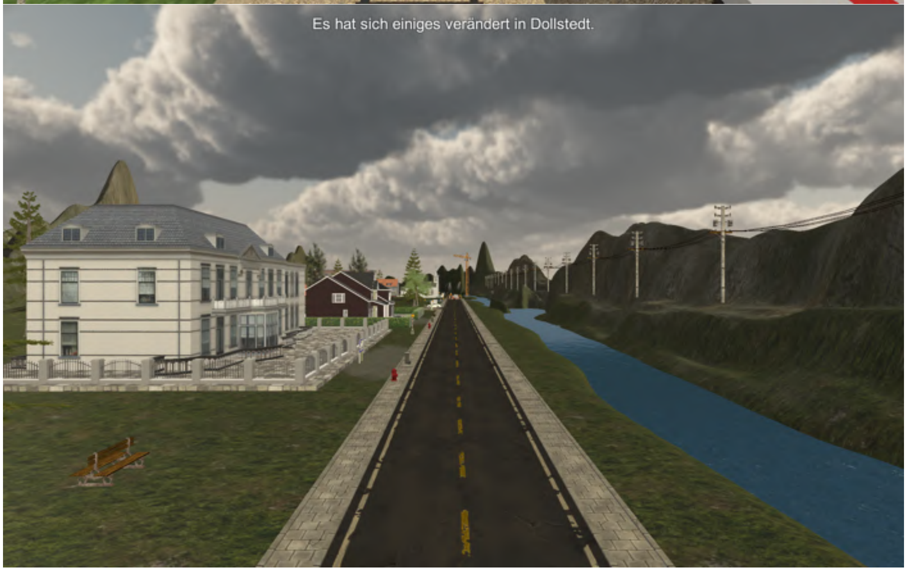

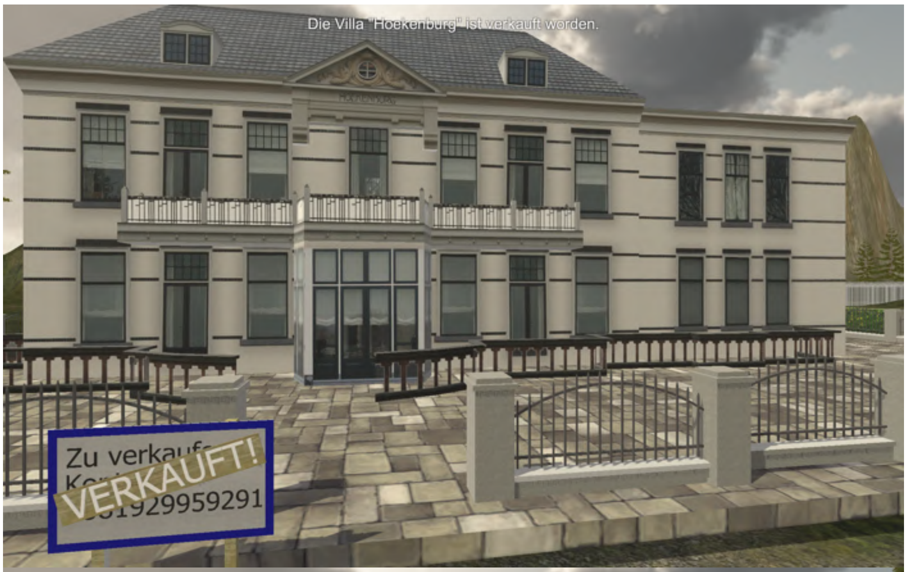
Die Villa "Hoekenburg" ist verkauft worden.
VERKAUFT!

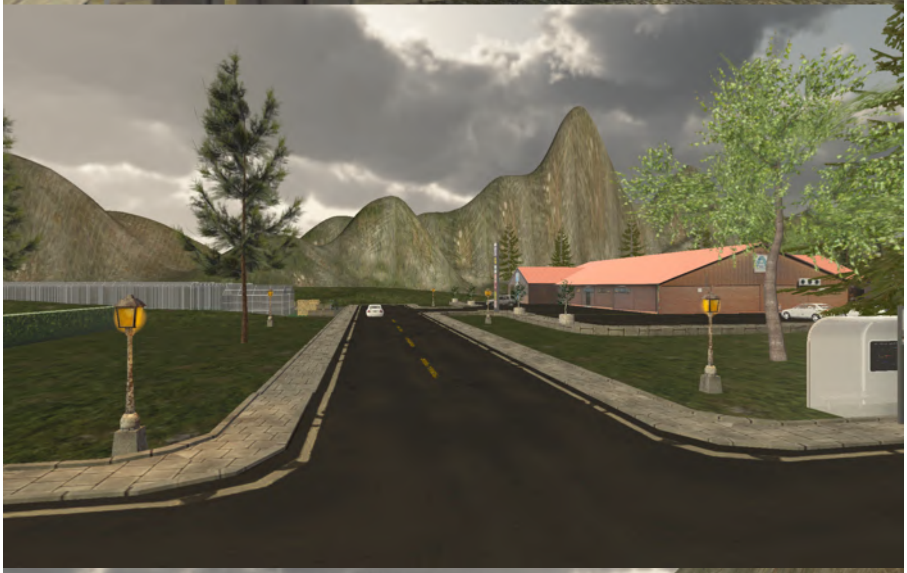

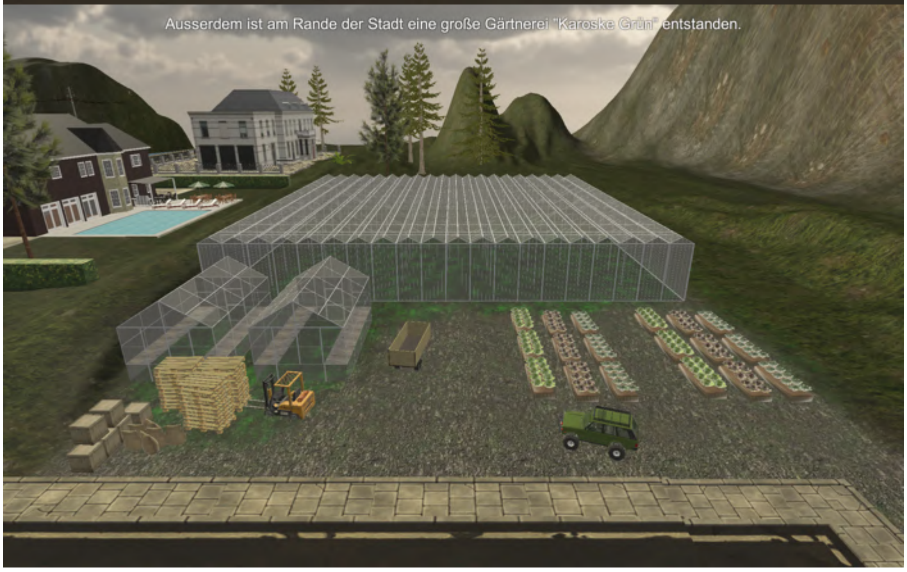
Ausserdem ist am Rande der Stadt eine große Gärtnerei "Karoske Grün" entstanden.

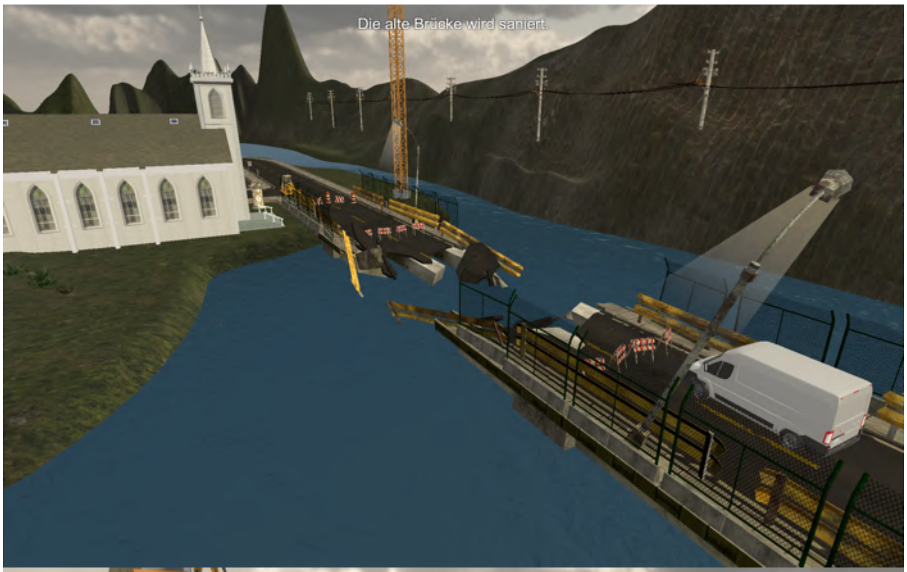
Die alte Brücke wird saniert.

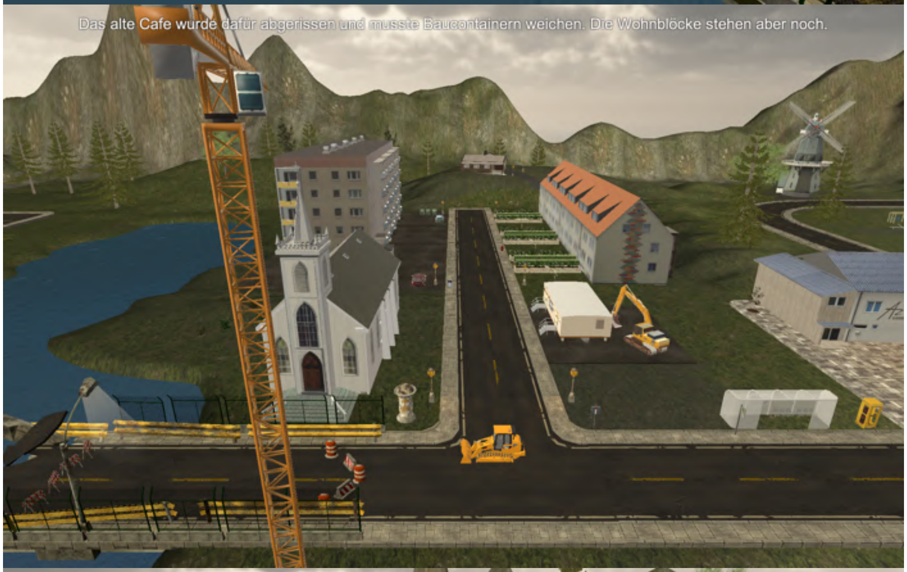
Das alte Cafe wurde dafür abgerissen und musste Baucontainern weichen. Die Wohnblöcke stehen aber noch.

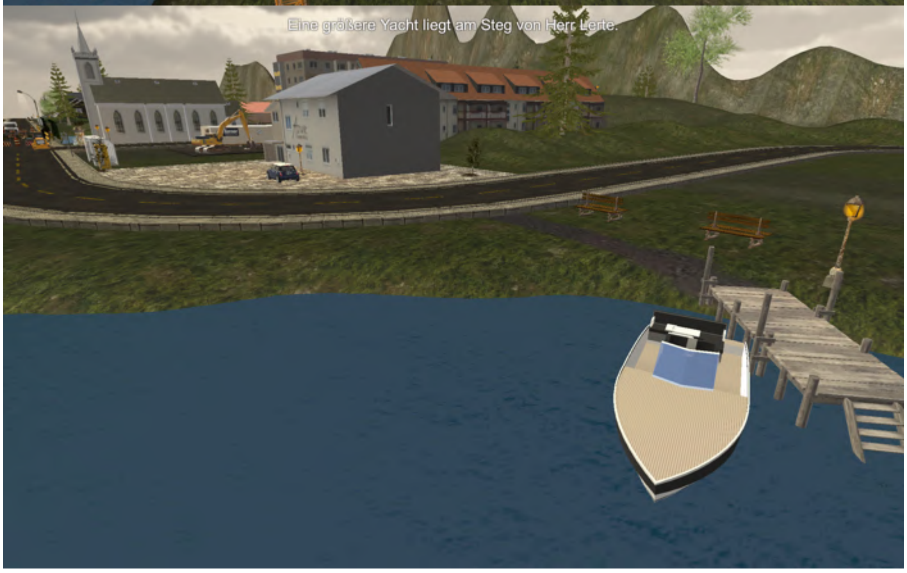
Eine größere Yacht liegt am Steg von Herr Lerte.

S4 Character information

In both phases, participants were provided information on the respective avatar at the beginning of a block of interactions/trials. While information on Updating-Avatars was consistent from Imprinting- to Modification-Phase, participants were provided conflicting information on Remapping-Avatars.

S4.1 Imprinting-phase

S4.1.1 Lerte (High-Power/Low-Affiliation, Updating-Avatar)

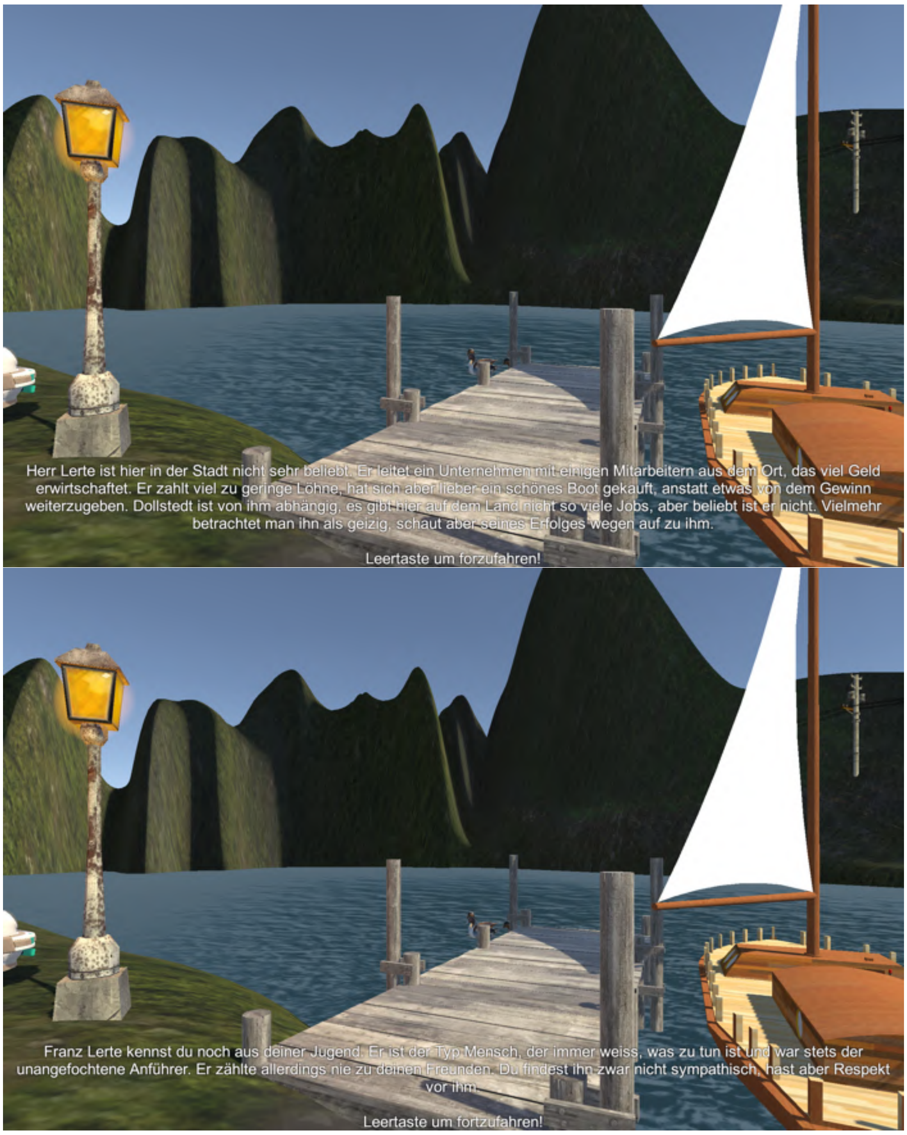

S4.1.2 Hoekenburg (High-Power/Low-Affiliation, Remapping-Avatar)

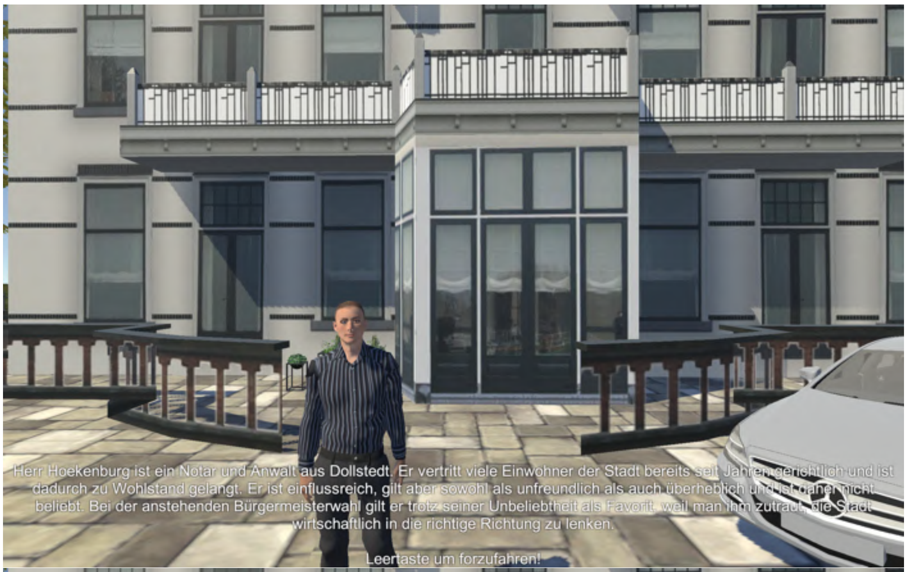

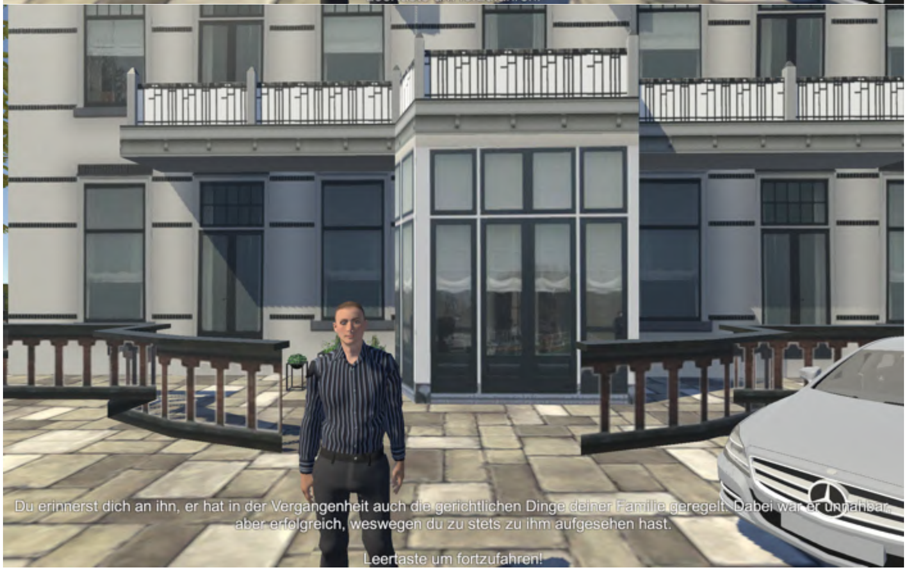

S4.1.3 Berger (Low-Power/High-Affiliation, Updating-Avatar)

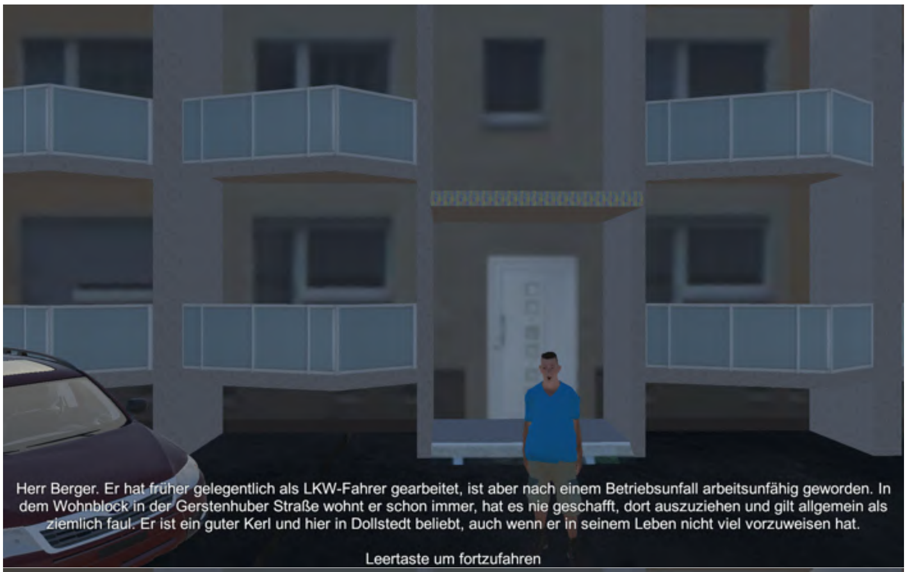

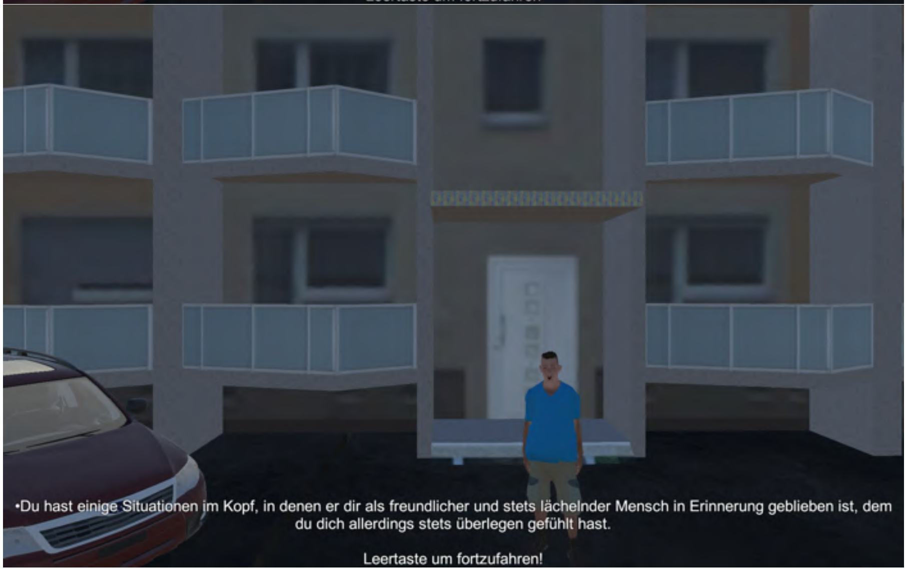

S4.1.4 Karoske (Low-Power/High-Affiliation, Remapping-Avatar)

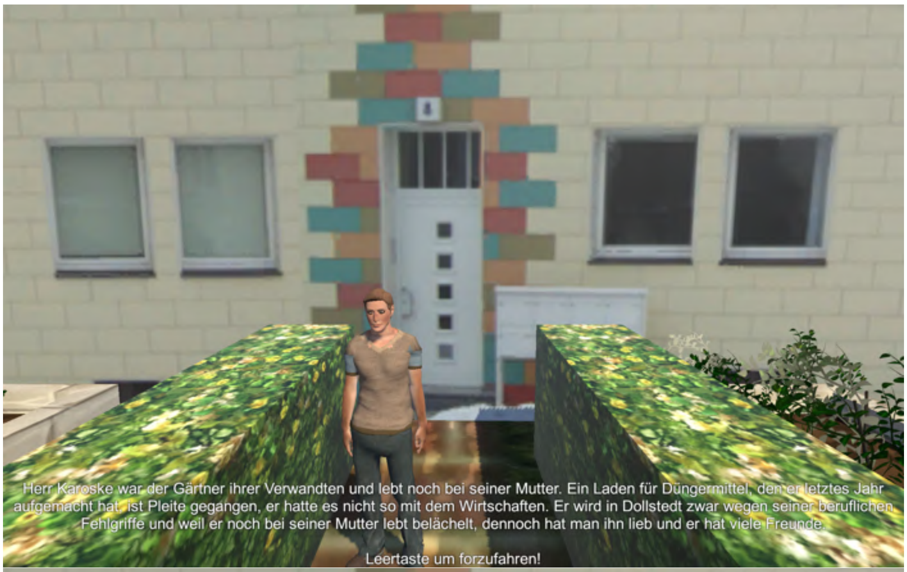

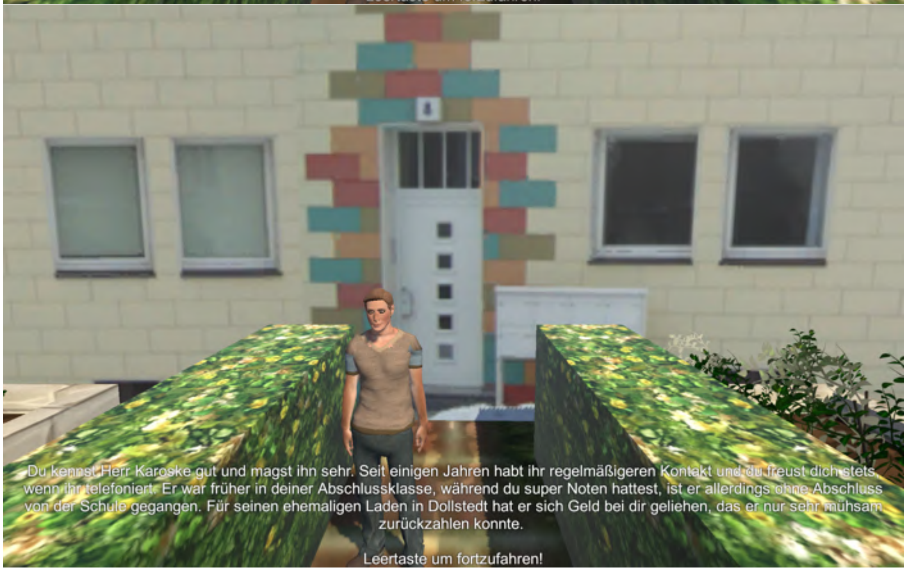

S4.2 Modification-Phase (experimental intervention)

S4.2.1 Lerte (High-Power/Low-Affiliation, Updating-Avatar)

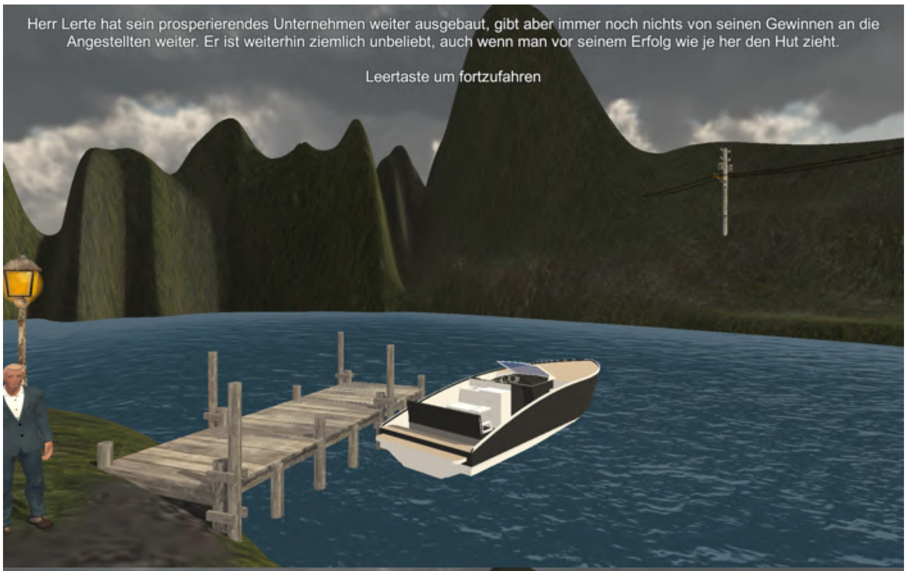

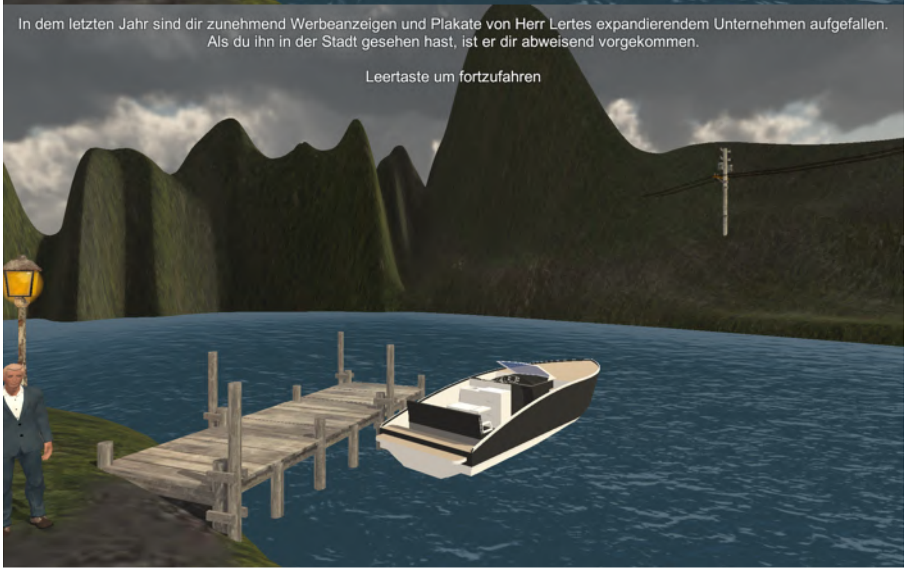

S4.2.2 Hoekenburg (High-Power/Low-Affiliation, Remapping-Avatar)

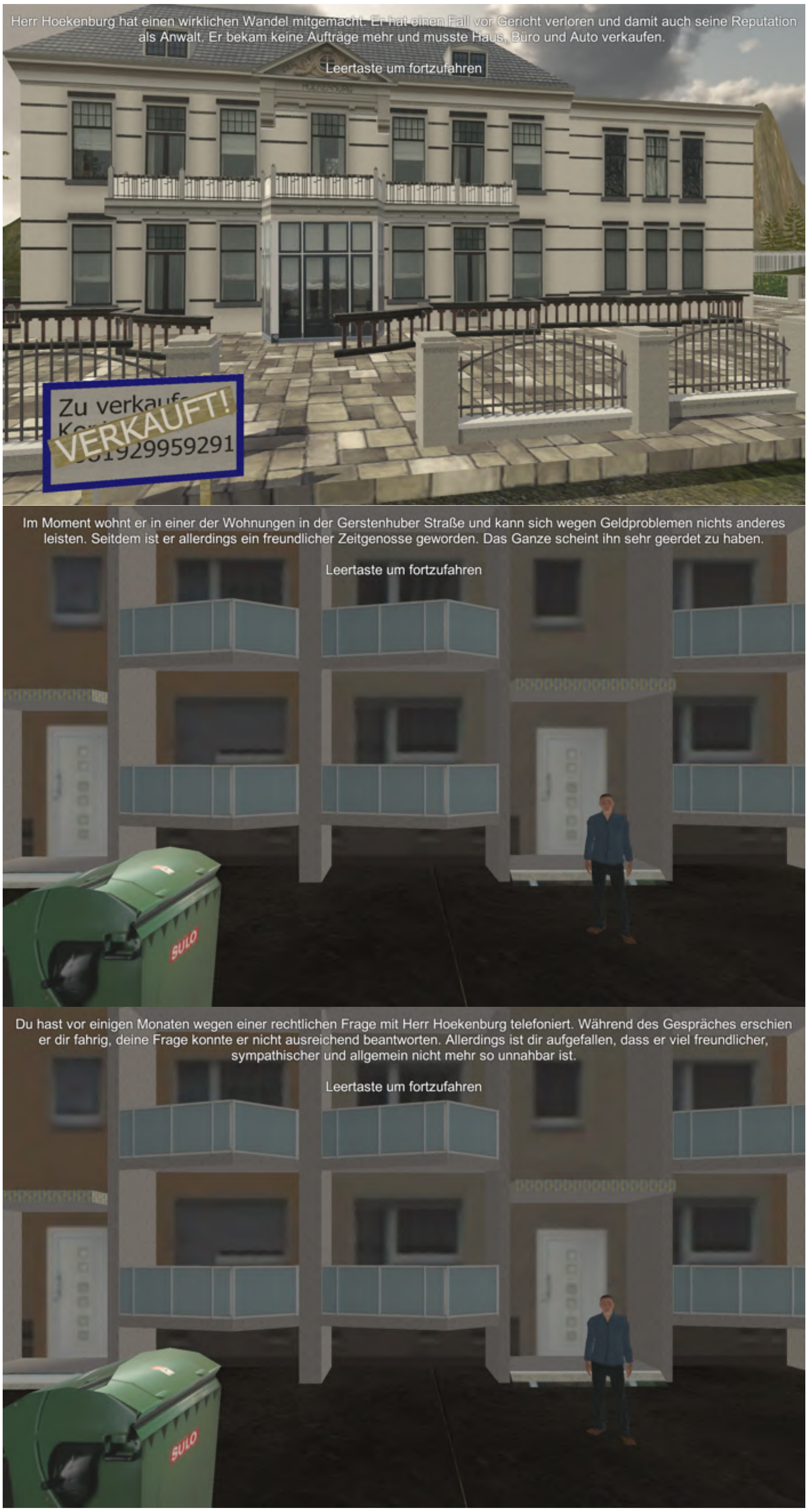

S4.2.3 Berger (Low-Power/High-Affiliation, Updating-Avatar)

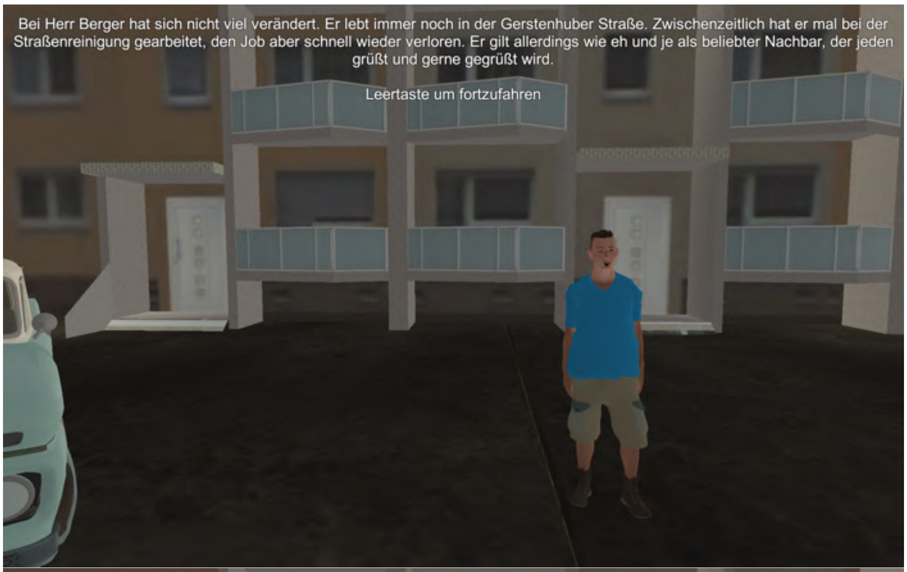

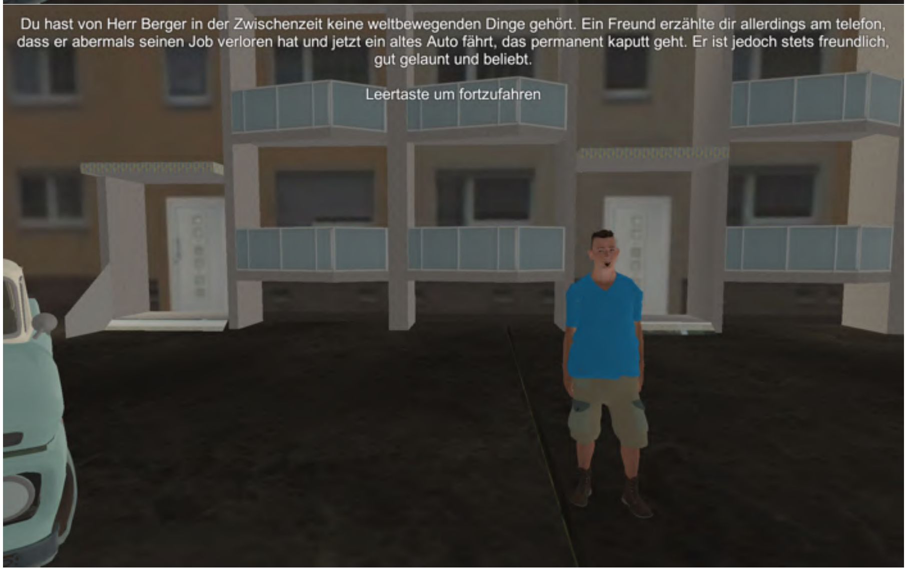

S4.2.4 Karoske (Low-Power/High-Affiliation, Remapping-Avatar)

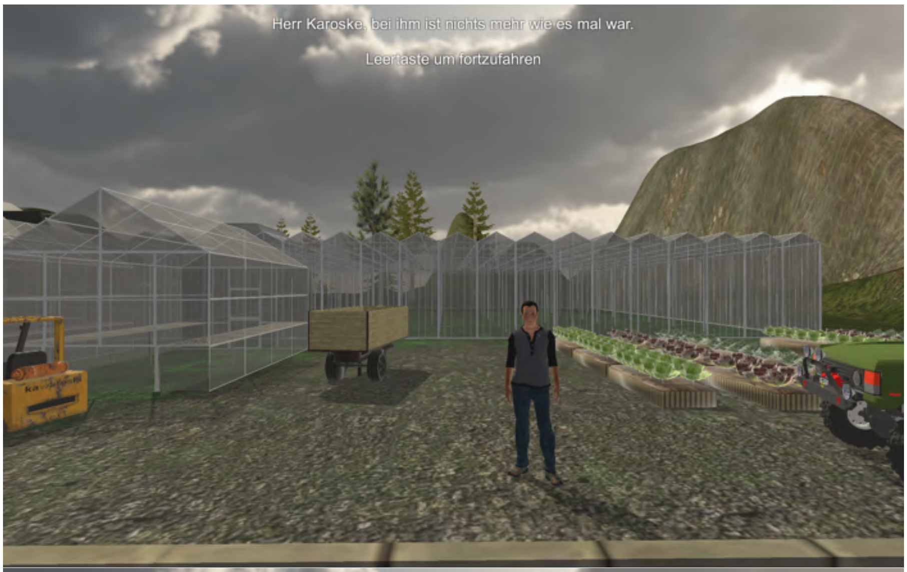

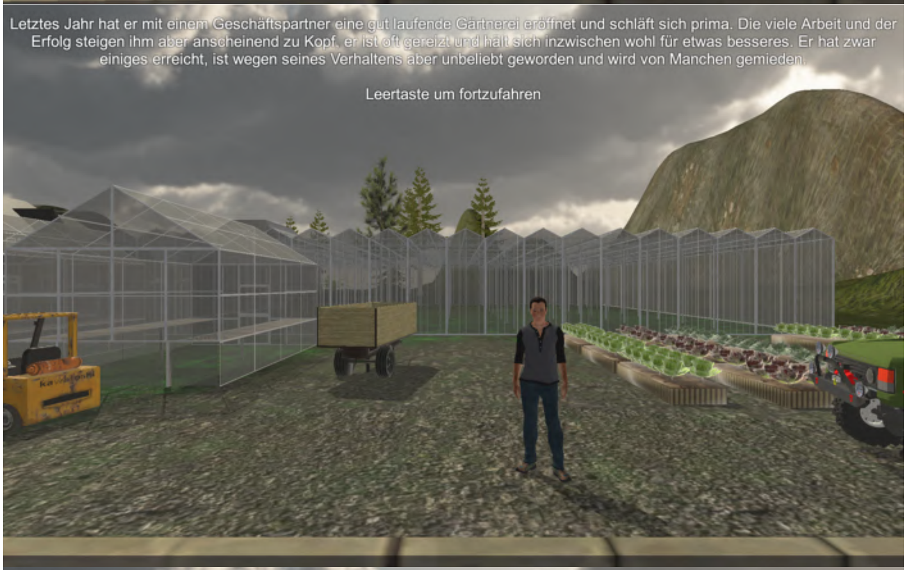

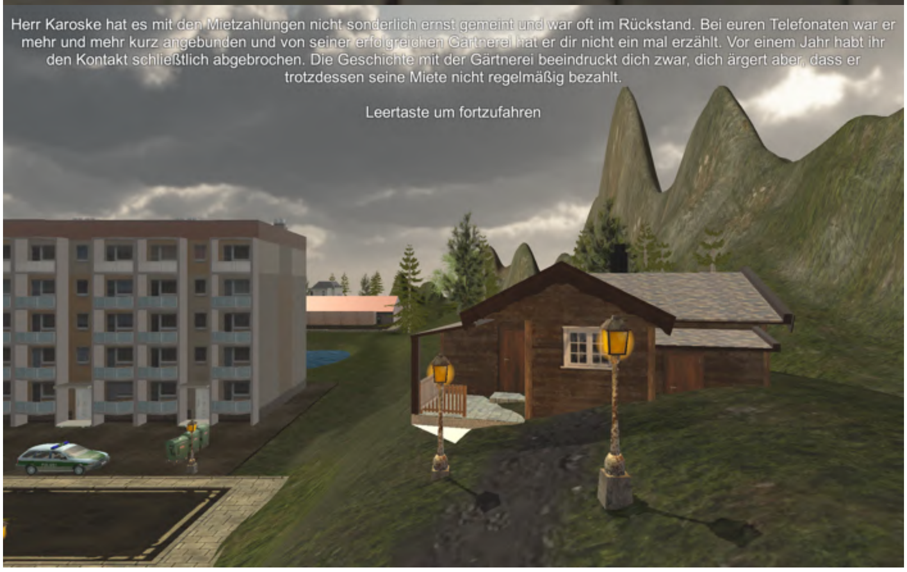

S5 Trials/Interactions

The following screenshots display the different interactions with each avatar in Imprinting- as well as Updating/Remapping-Phase. Narratives used to develop the storyline and increase immersion are not included.

S5.1 Imprinting-phase

S5.1.1 Lerte (High-Power/Low-Affiliation, Updating-Avatar)

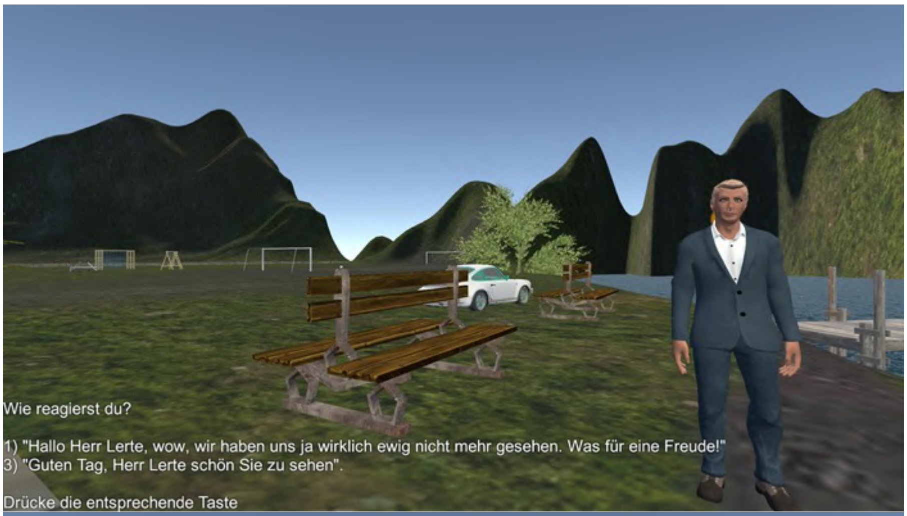

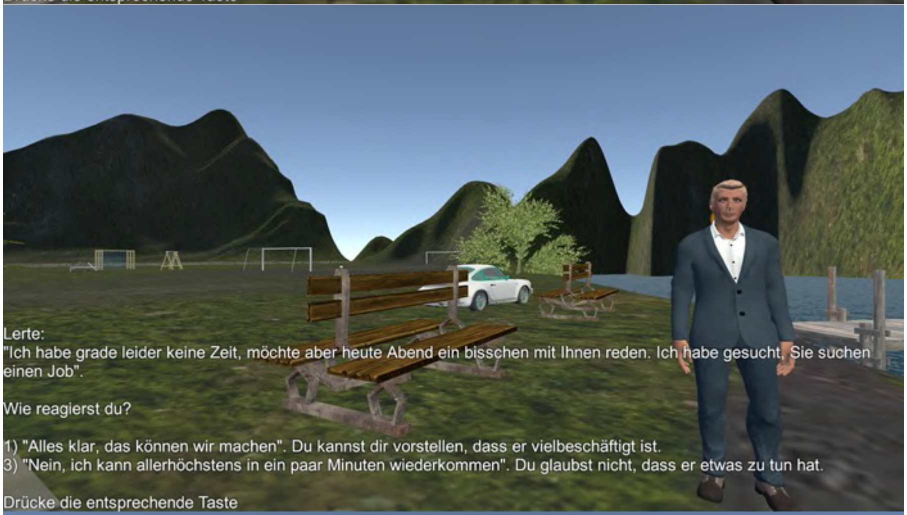

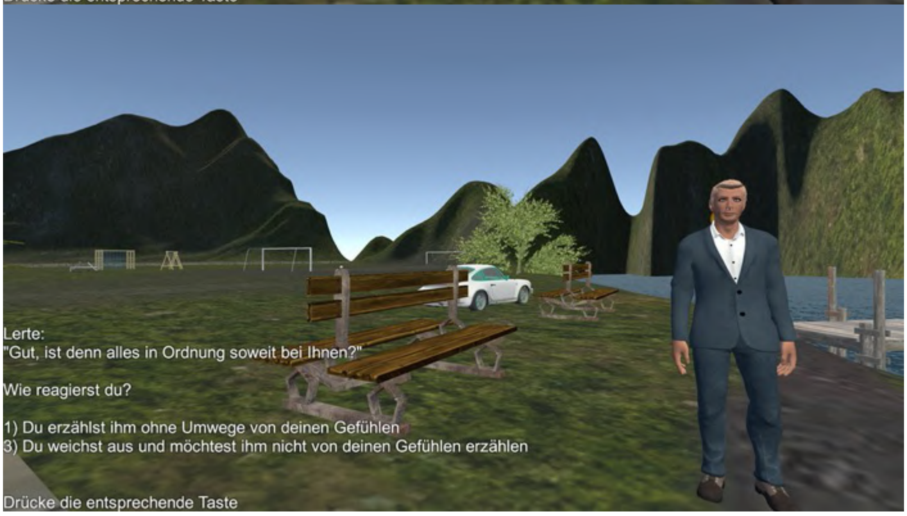

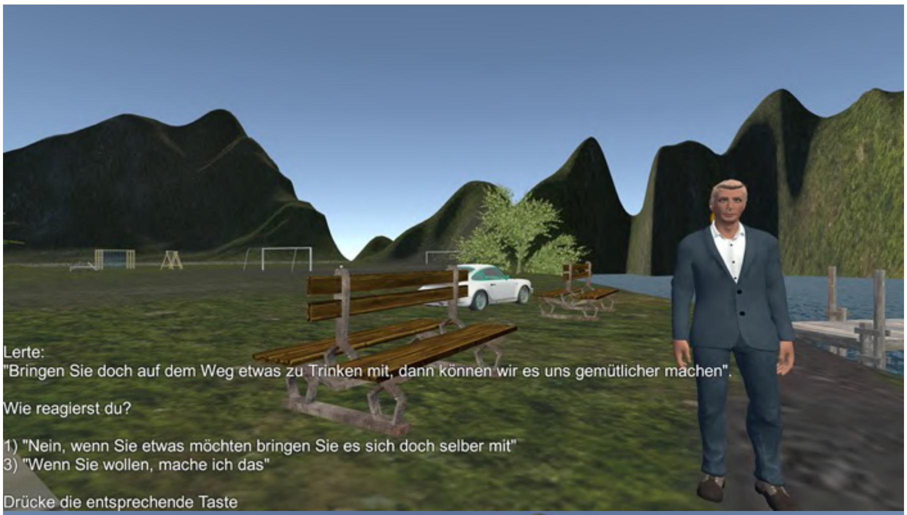
Lerte:
"Bringen Sie doch auf dem Weg etwas zu Trinken mit, dann können wir es uns gemütlicher machen".
Wie reagierst du?
1) "Nein, wenn Sie etwas möchten bringen Sie es sich doch selber mit"
3) "Wenn Sie wollen, mache ich das"
Drücke die entsprechende Taste

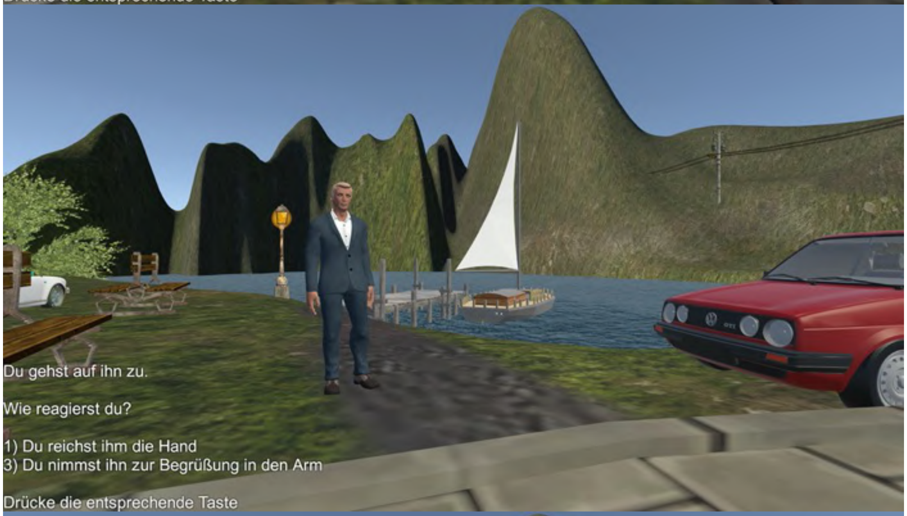
Du gehst auf ihn zu.
Wie reagierst du?
1) Du reichst ihm die Hand
3) Du nimmst ihn zur Begrüßung in den Arm
Drücke die entsprechende Taste

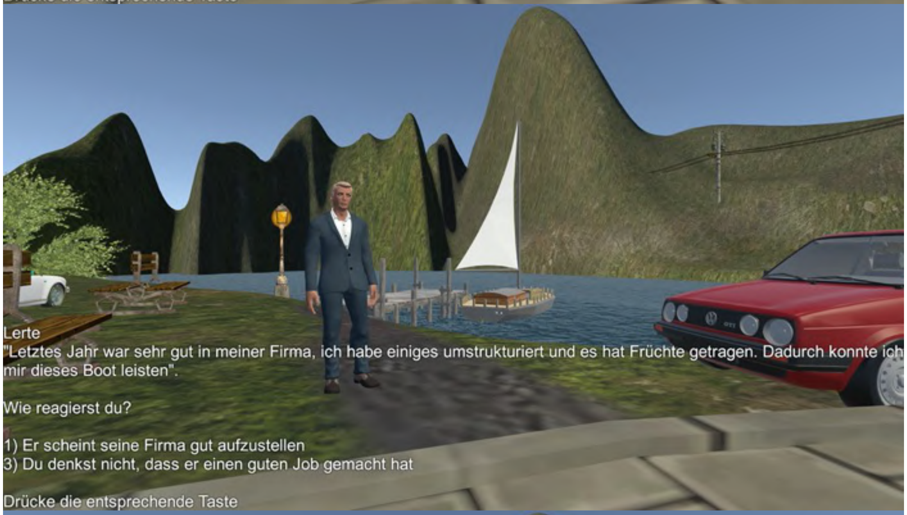
Lerte
"Letztes Jahr war sehr gut in meiner Firma, ich habe einiges umstrukturiert und es hat Früchte getragen. Dadurch konnte ich mir dieses Boot leisten".
Wie reagierst du?
1) Er scheint seine Firma gut aufzustellen
3) Du denkst nicht, dass er einen guten Job gemacht hat
Drücke die entsprechende Taste

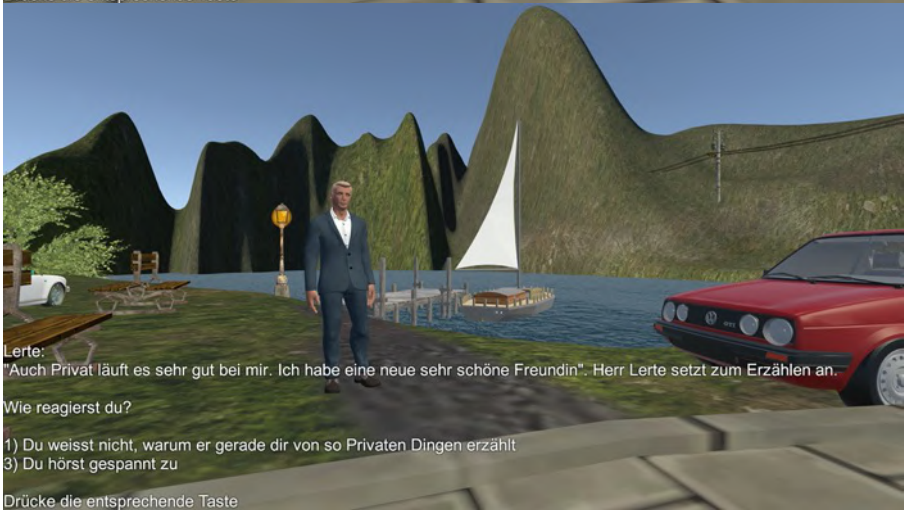
Lerte:
"Auch Privat läuft es sehr gut bei mir. Ich habe eine neue sehr schöne Freundin". Herr Lerte setzt zum Erzählen an.
Wie reagierst du?
1) Du weisst nicht, warum er gerade dir von so Privaten Dingen erzählt
3) Du hörst gespannt zu
Drücke die entsprechende Taste

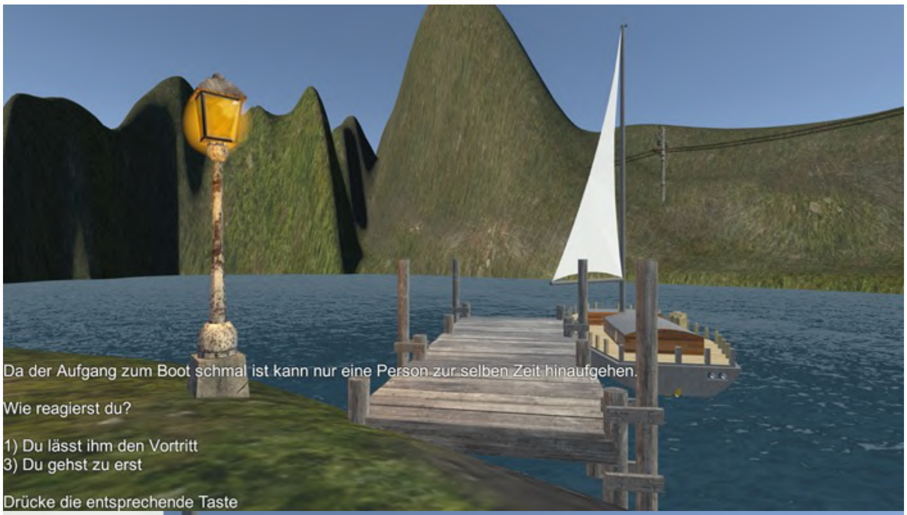
Da der Aufgang zum Boot schmal ist kann nur eine Person zur selben Zeit hinaufgehen.
Wie reagierst du?
1) Du lässt ihm den Vortritt
3) Du gehst zu erst
Drücke die entsprechende Taste

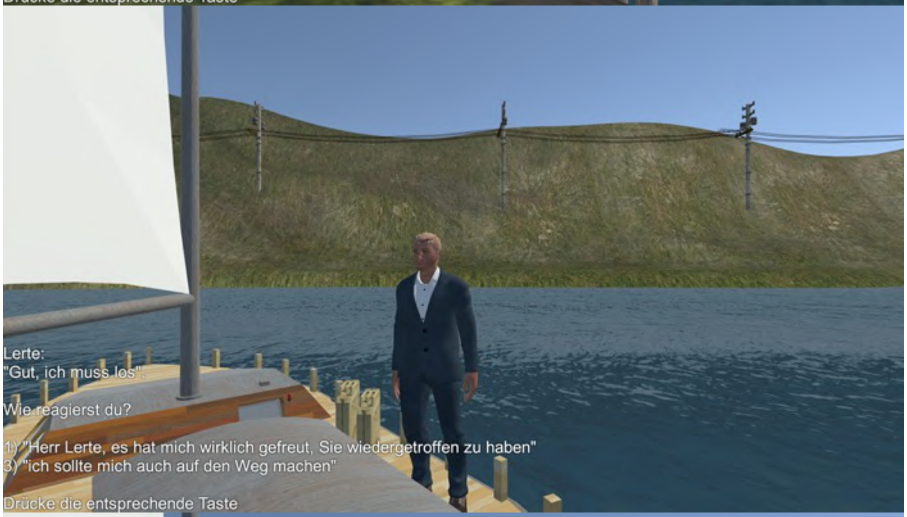
Lerte:
"Gut, ich muss los"
Wie reagierst du?
1) "Herr Lerte, es hat mich wirklich gefreut, Sie wiedergetroffen zu haben"
3) "ich sollte mich auch auf den Weg machen"
Drücke die entsprechende Taste

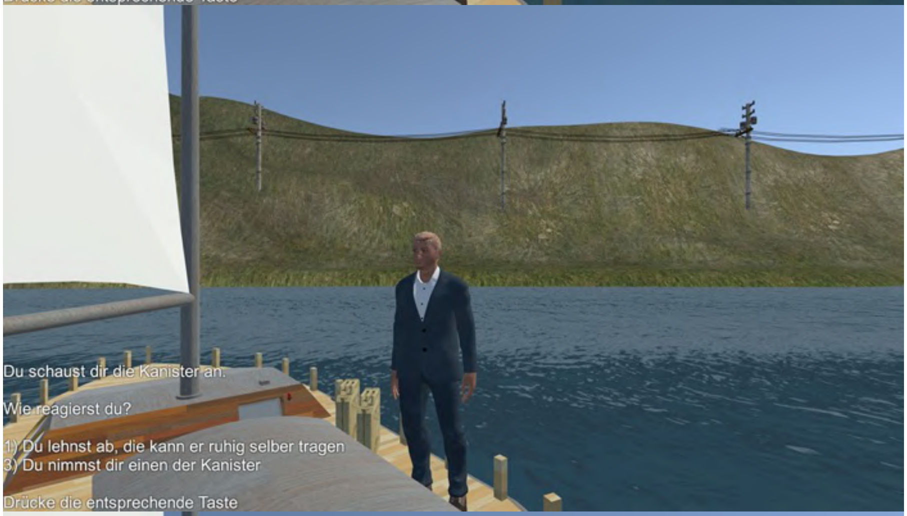
Du schaust dir die Kanister an.
Wie reagierst du?
1) Du lehnst ab, die kann er ruhig selber tragen
3) Du nimmst dir einen der Kanister
Drücke die entsprechende Taste

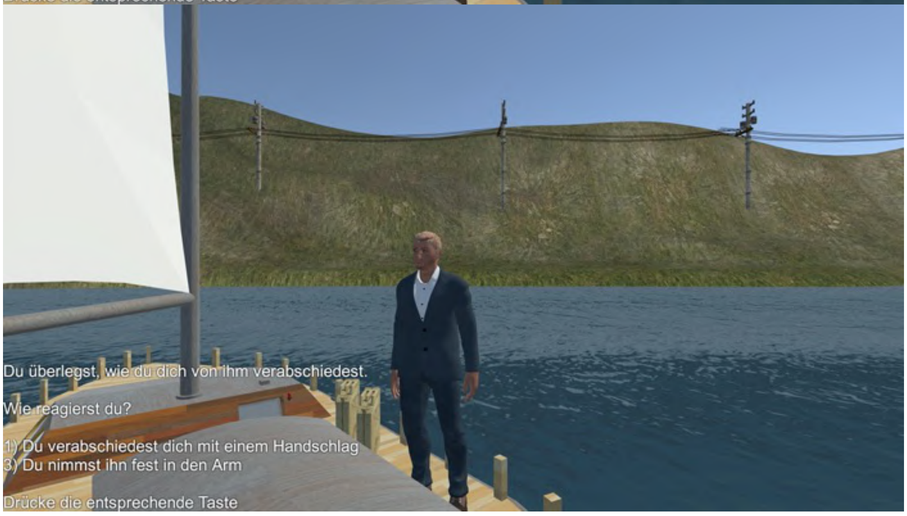
Du überlegst, wie du dich von ihm verabschiedest.
Wie reagierst du?
1) Du verabschiedest dich mit einem Handschlag
3) Du nimmst ihn fest in den Arm
Drücke die entsprechende Taste

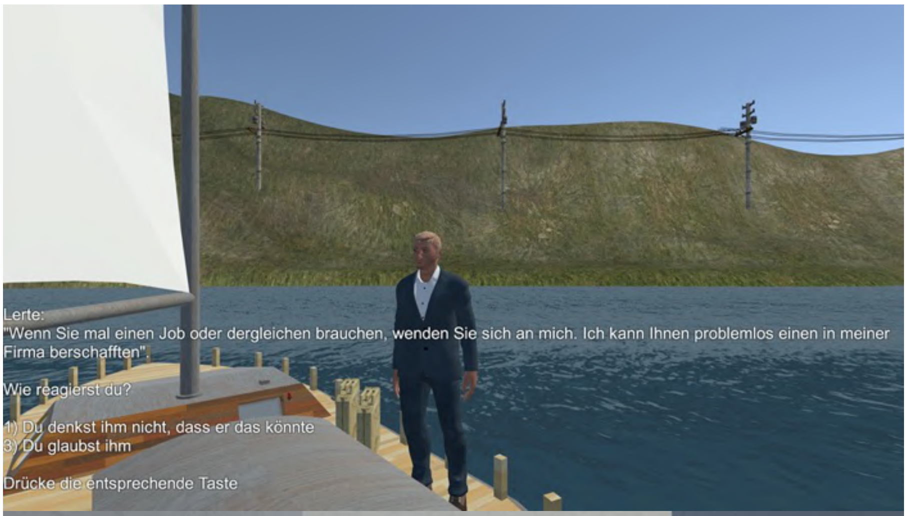
Lerte:
"Wenn Sie mal einen Job oder dergleichen brauchen, wenden Sie sich an mich. Ich kann Ihnen problemlos einen in meiner Firma berschafften".
Wie reagierst du?
1) Du denkst ihm nicht, dass er das könnte
3) Du glaubst ihm
Drücke die entsprechende Taste
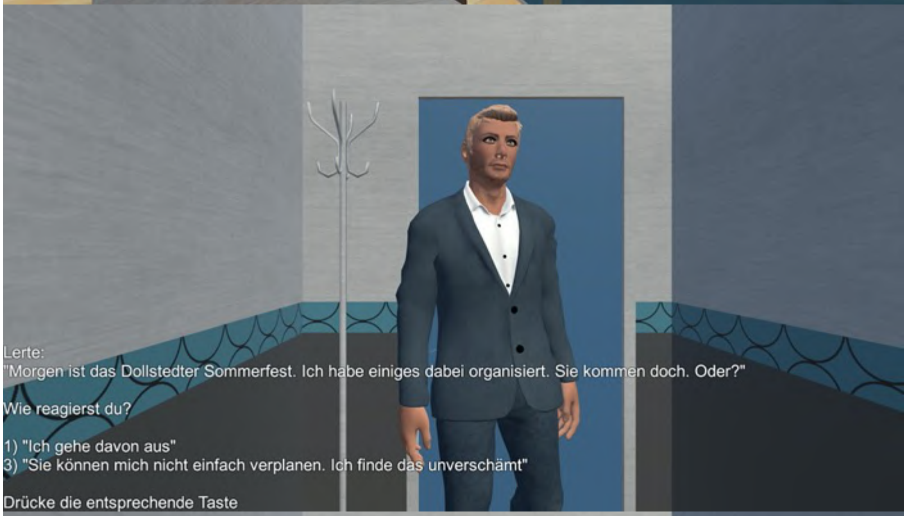
Lerte:
"Morgen ist das Dollstedter Sommerfest. Ich habe einiges dabei organisiert. Sie kommen doch. Oder?"
Wie reagierst du?
1) "Ich gehe davon aus"
3) "Sie können mich nicht einfach verplanen. Ich finde das unverschämt"
Drücke die entsprechende Taste
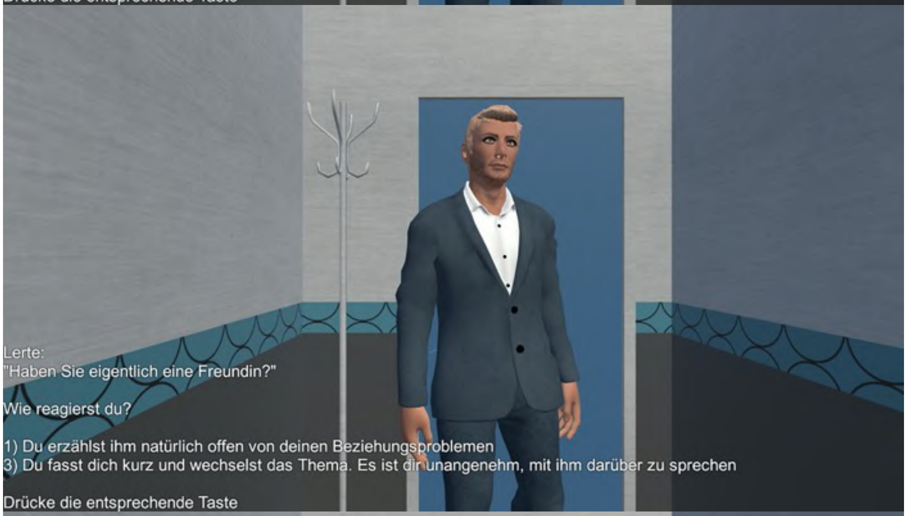
Lerte:
"Haben Sie eigentlich eine Freundin?"
Wie reagierst du?
1) Du erzählst ihm natürlich offen von deinen Beziehungsproblemen
3) Du fasst dich kurz und wechselst das Thema. Es ist dir unangenehm, mit ihm darüber zu sprechen
Drücke die entsprechende Taste
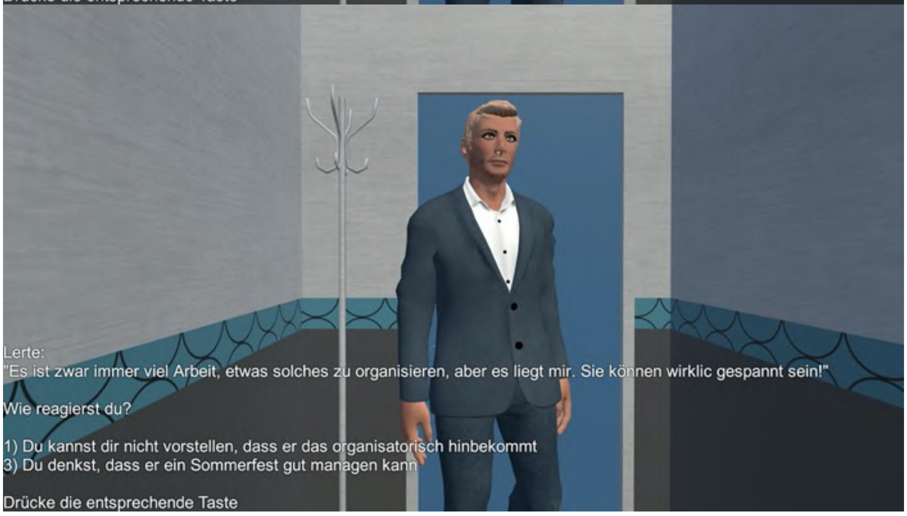
Lerte:
"Es ist zwar immer viel Arbeit, etwas solches zu organisieren, aber es liegt mir. Sie können wirklic gespannt sein!"
Wie reagierst du?
1) Du kannst dir nicht vorstellen, dass er das organisatorisch hinbekommt
3) Du denkst, dass er ein Sommerfest gut managen kann
Drücke die entsprechende Taste

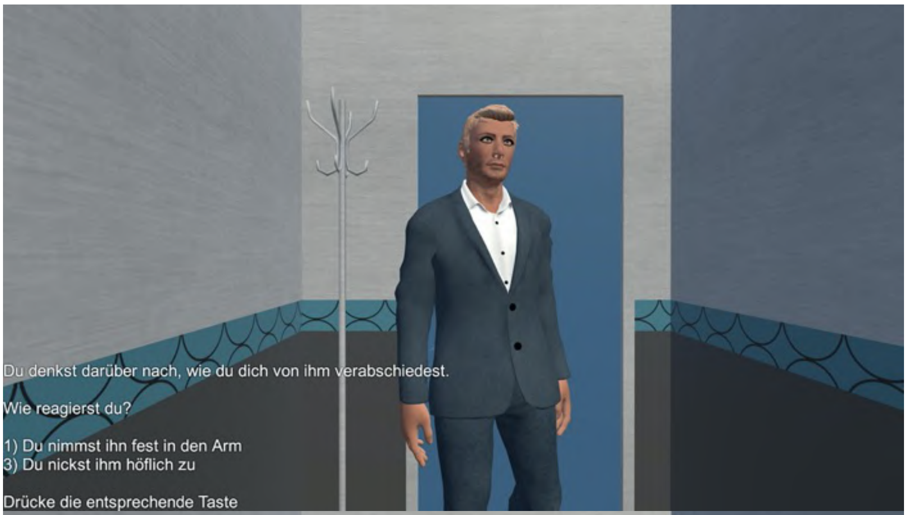
Du denkst darüber nach, wie du dich von ihm verabschiedest.
Wie reagierst du?
1) Du nimmst ihn fest in den Arm
3) Du nickst ihm höflich zu
Drücke die entsprechende Taste

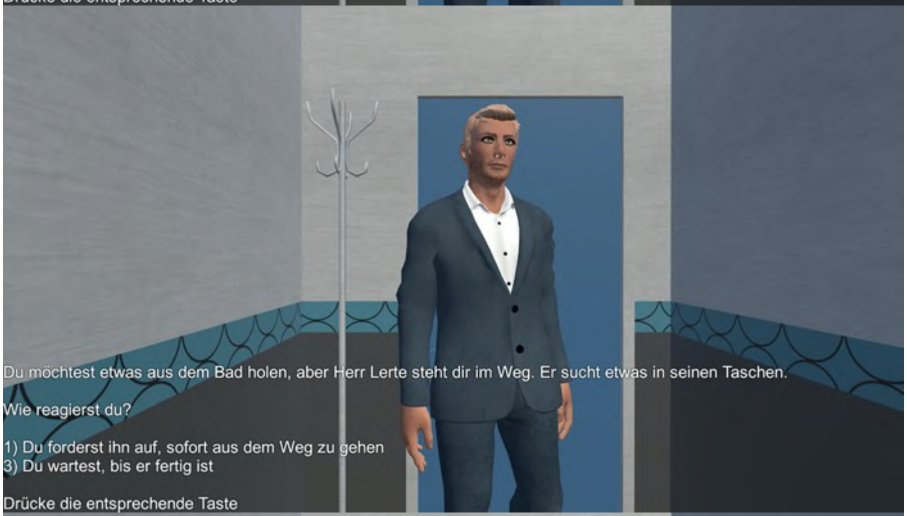
Du möchtest etwas aus dem Bad holen, aber Herr Lerte steht dir im Weg. Er sucht etwas in seinen Taschen.
Wie reagierst du?
1) Du forderst ihn auf, sofort aus dem Weg zu gehen
3) Du wartest, bis er fertig ist
Drücke die entsprechende Taste

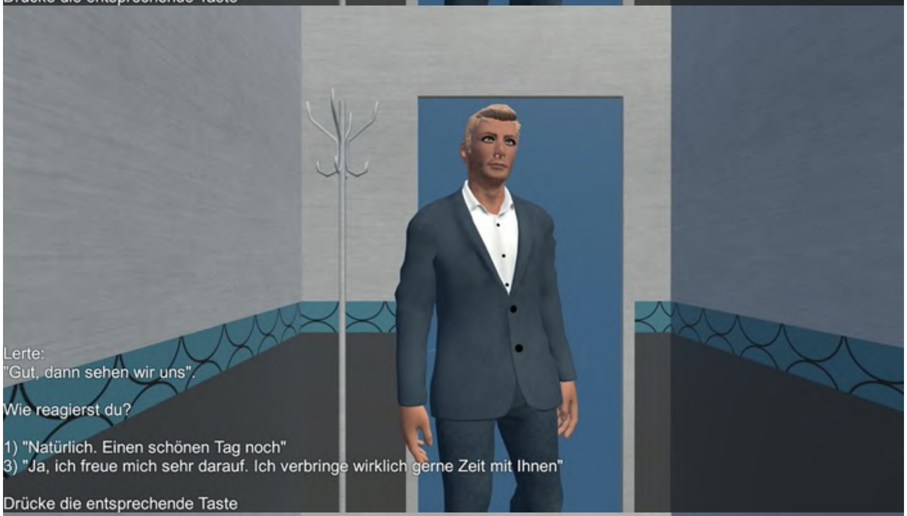
Lerte:
"Gut, dann sehen wir uns".
Wie reagierst du?
1) "Natürlich. Einen schönen Tag noch"
3) "Ja, ich freue mich sehr darauf. Ich verbringe wirklich gerne Zeit mit Ihnen"
Drücke die entsprechende Taste

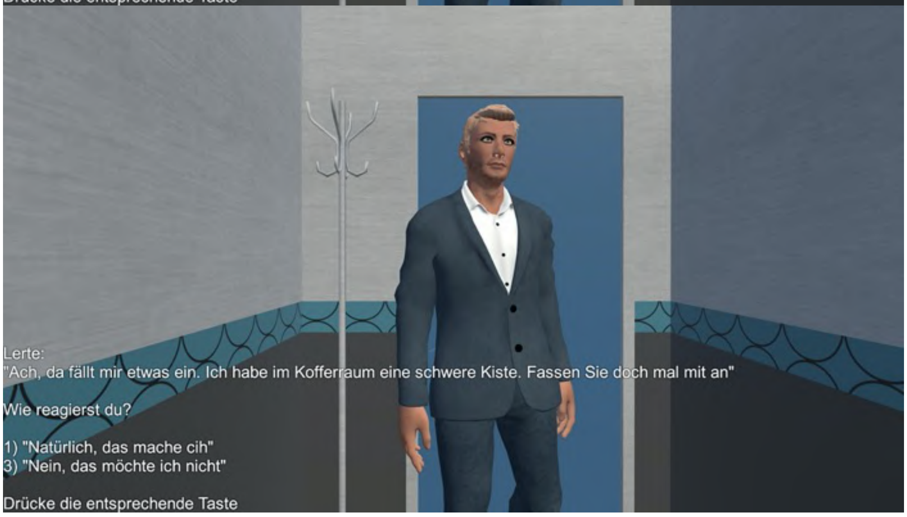
Lerte:
"Ach, da fällt mir etwas ein. Ich habe im Kofferraum eine schwere Kiste. Fassen Sie doch mal mit an"
Wie reagierst du?
1) "Natürlich, das mache cih"
3) "Nein, das möchte ich nicht"
Drücke die entsprechende Taste

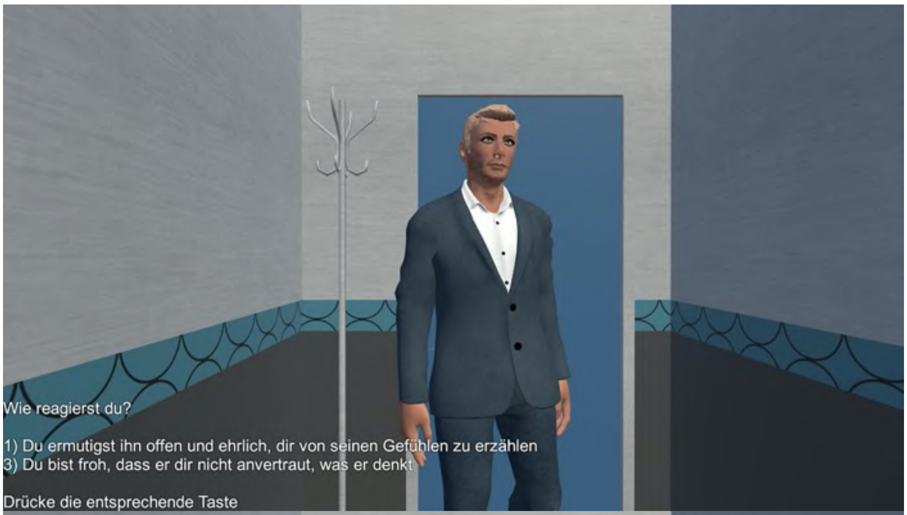
Wie reagierst du?
1) Du ermutigst ihn offen und ehrlich, dir von seinen Gefühlen zu erzählen
3) Du bist froh, dass er dir nicht anvertraut, was er denkt
Drücke die entsprechende Taste

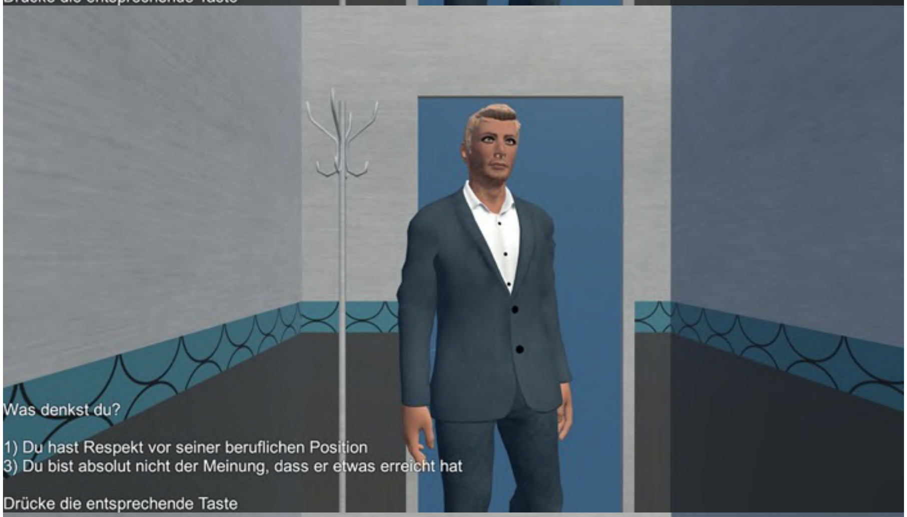
Was denkst du?
1) Du hast Respekt vor seiner beruflichen Position
3) Du bist absolut nicht der Meinung, dass er etwas erreicht hat
Drücke die entsprechende Taste

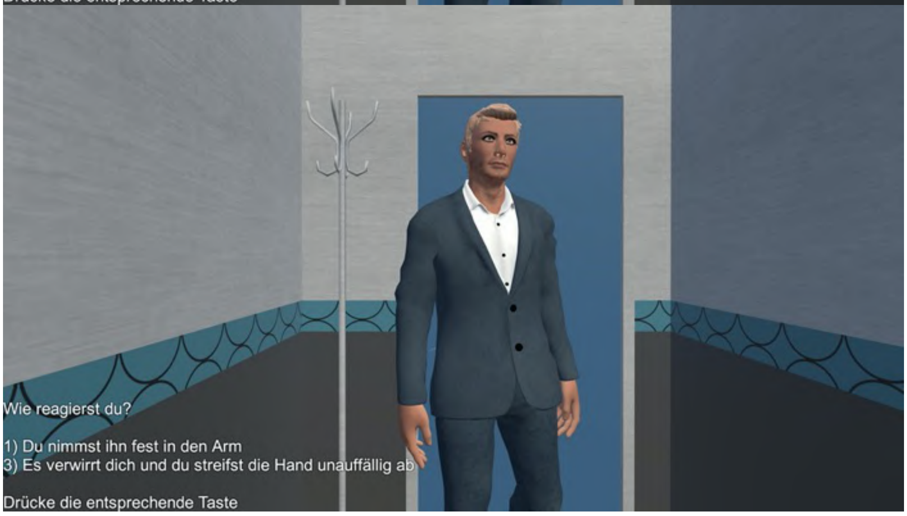
Wie reagierst du?
1) Du nimmst ihn fest in den Arm
3) Es verwirrt dich und du streifst die Hand unauffällig ab
Drücke die entsprechende Taste

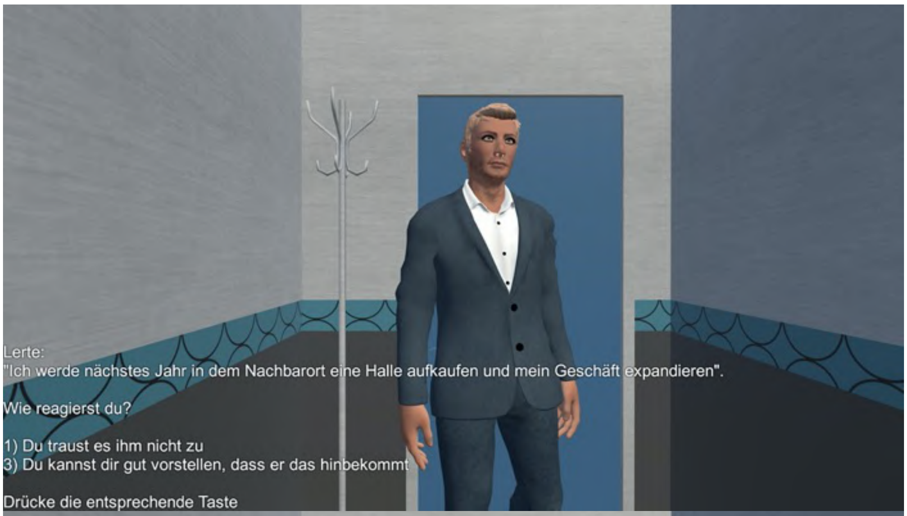
Lerte:
"Ich werde nächstes Jahr in dem Nachbarort eine Halle aufkaufen und mein Geschäft expandieren".
Wie reagierst du?
1) Du traust es ihm nicht zu
3) Du kannst dir gut vorstellen, dass er das hinbekommt
Drücke die entsprechende Taste

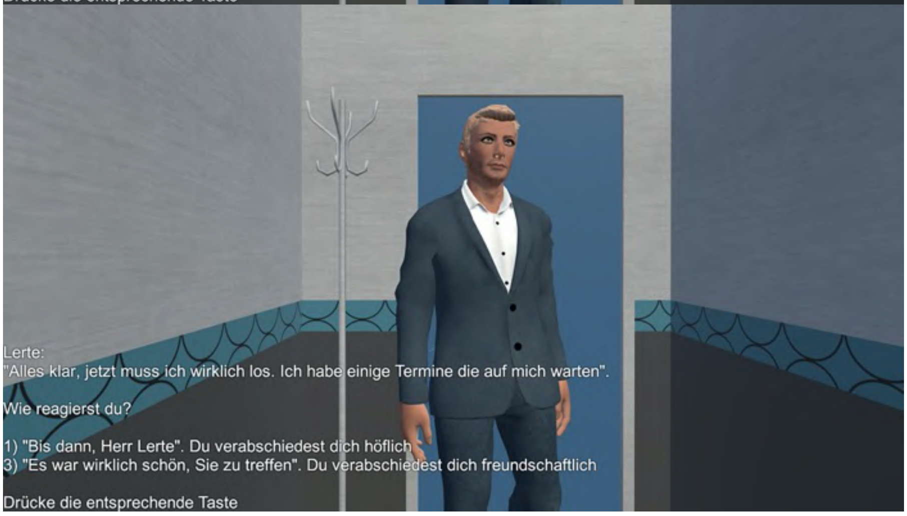
Lerte:
"Alles klar, jetzt muss ich wirklich los. Ich habe einige Termine die auf mich warten".
Wie reagierst du?
1) "Bis dann, Herr Lerte". Du verabschiedest dich höflich
3) "Es war wirklich schön, Sie zu treffen". Du verabschiedest dich freundschaftlich
Drücke die entsprechende Taste

S5.1.2 Hoekenburg (High-Power/Low-Affiliation, Remapping-Avatar)

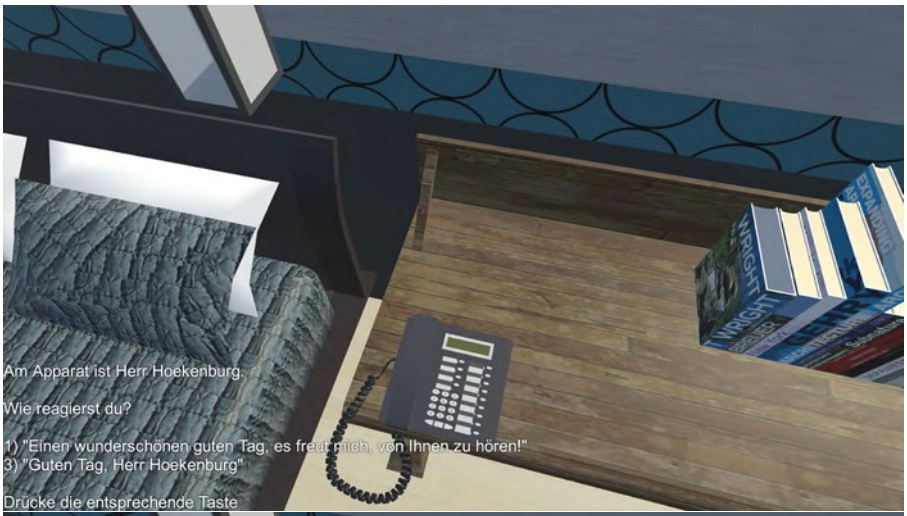

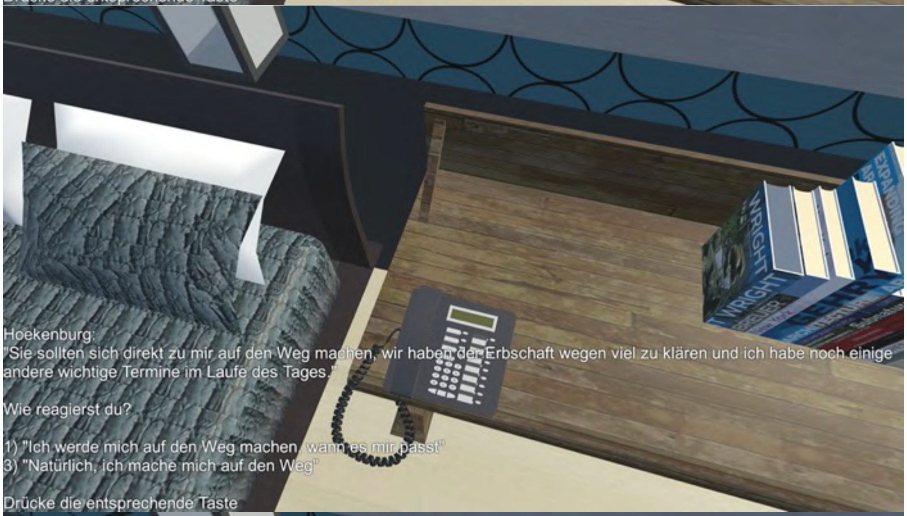

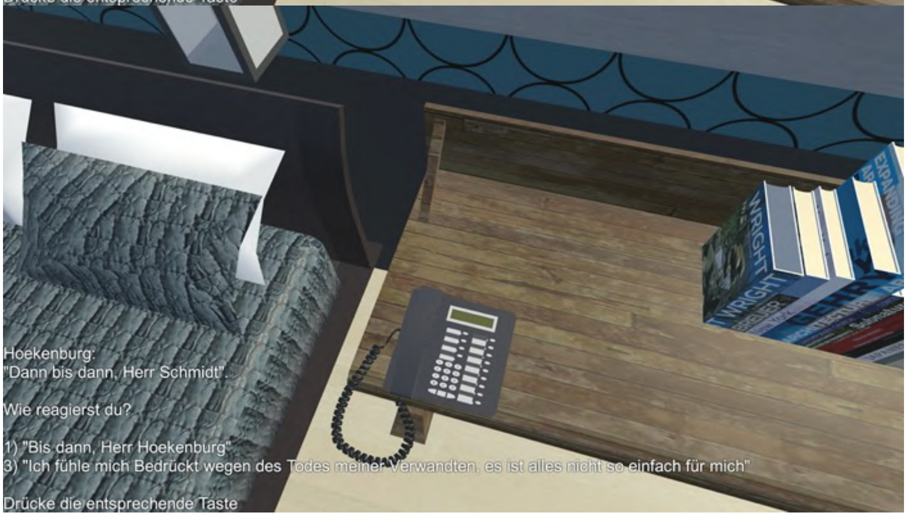

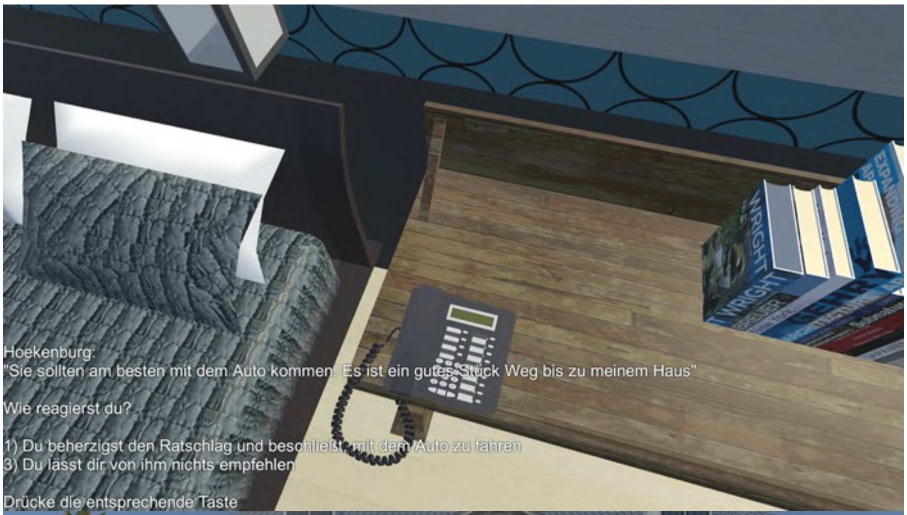
Hoekenburg:
"Sie sollten am besten mit dem Auto kommen. Es ist ein gutes Stück Weg bis zu meinem Haus"
Wie reagierst du?
1) Du beherzigst den Ratschlag und beschließt, mit dem Auto zu fahren
3) Du lässt dir von ihm nichts empfehlen
Drücke die entsprechende Taste

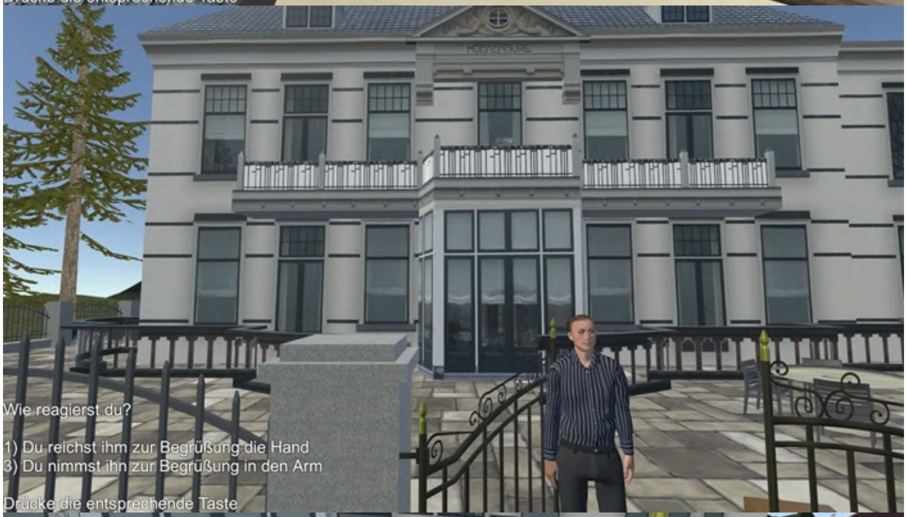
Wie reagierst du?
1) Du reichst ihm zur Begrüßung die Hand
3) Du nimmst ihn zur Begrüßung in den Arm
Drücke die entsprechende Taste

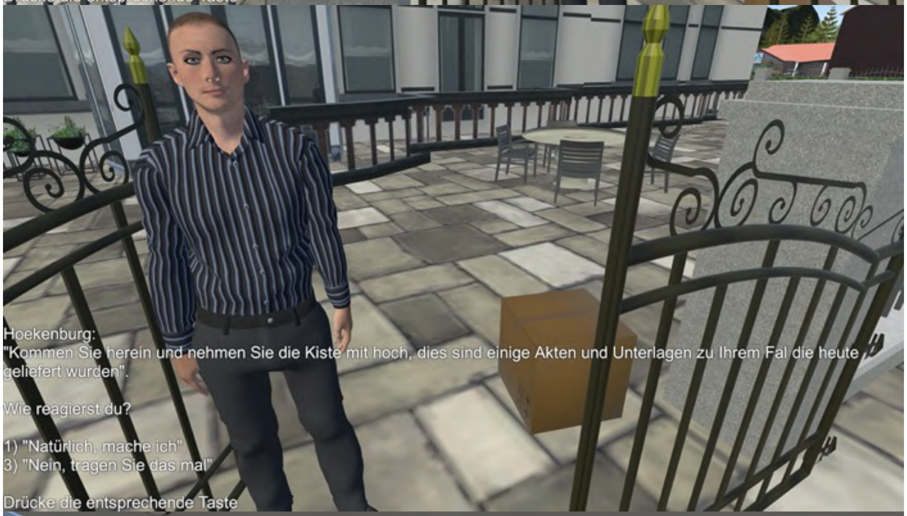
Hoekenburg:
"Kommen Sie herein und nehmen Sie die Kiste mit hoch, dies sind einige Akten und Unterlagen zu Ihrem Fal die heute geliefert wurden".
Wie reagierst du?
1) "Natürlich, mache ich"
3) "Nein, tragen Sie das mal"
Drücke die entsprechende Taste

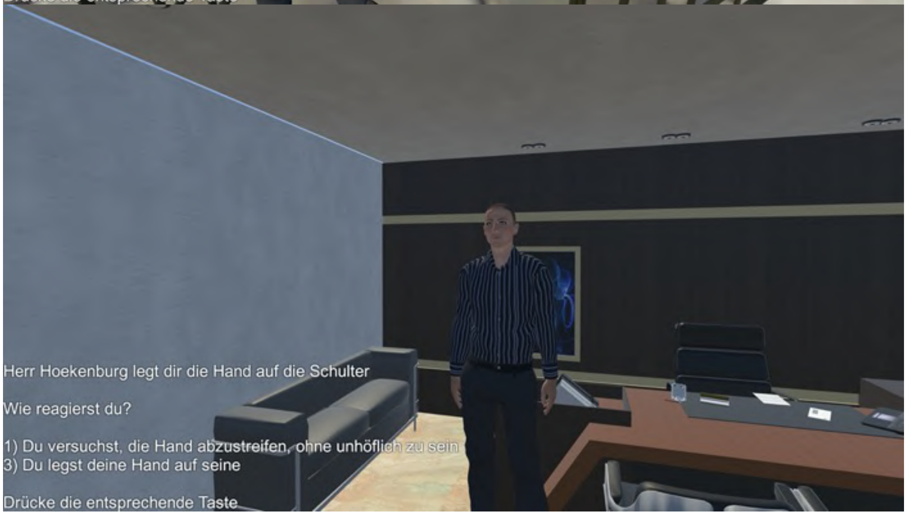
Herr Hoekenburg legt dir die Hand auf die Schulter
Wie reagierst du?
1) Du versuchst, die Hand abzustreifen, ohne unhöflich zu sein
3) Du legst deine Hand auf seine
Drücke die entsprechende Taste

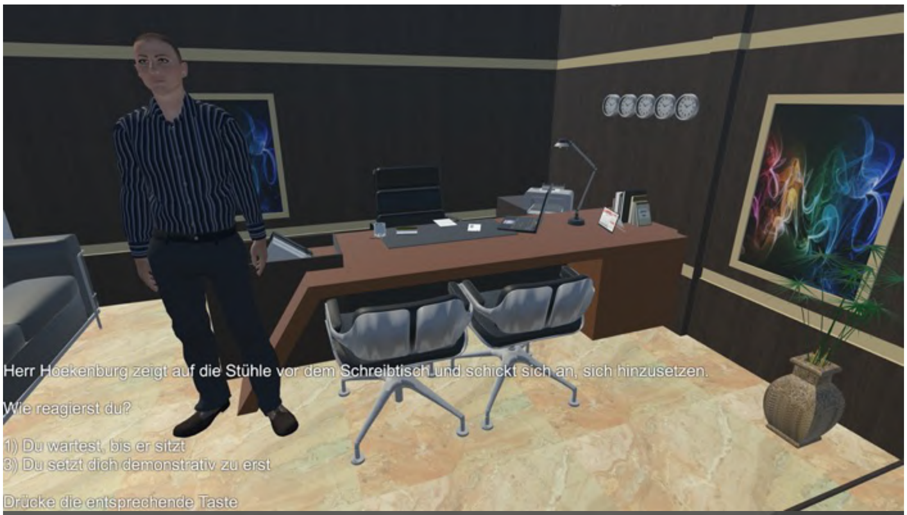
Herr Hoekenburg zeigt auf die Stühle vor dem Schreibtisch und schickt sich an, sich hinzusetzen.
Wie reagierst du?
1) Du wartest, bis er sitzt
3) Du setzt dich demonstrativ zu erst
Drücke die entsprechende Taste

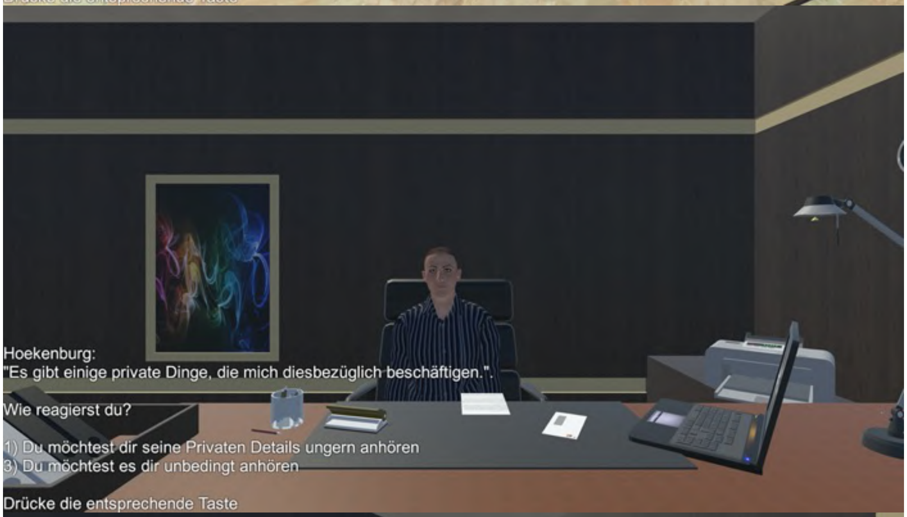
Hoekenburg:
"Es gibt einige private Dinge, die mich diesbezüglich beschäftigen.".
Wie reagierst du?
1) Du möchtest dir seine Privaten Details ungern anhören
3) Du möchtest es dir unbedingt anhören
Drücke die entsprechende Taste

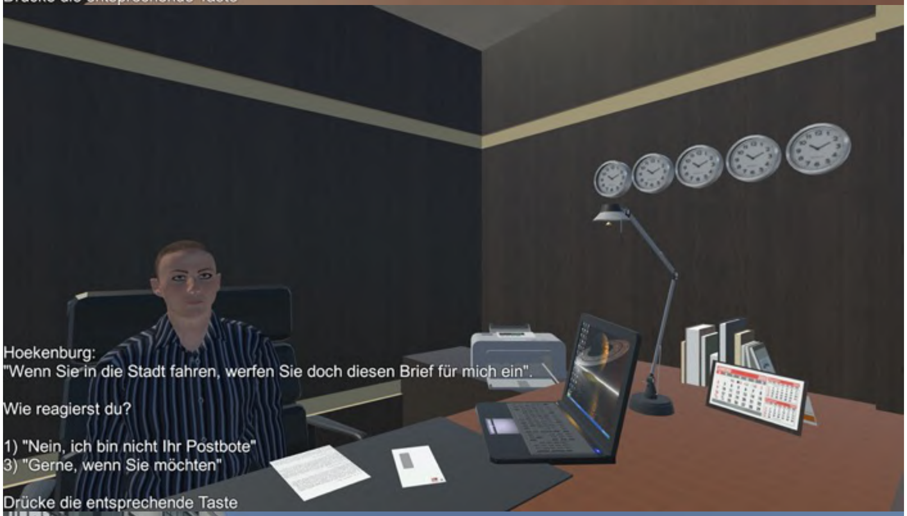
Hoekenburg:
"Wenn Sie in die Stadt fahren, werfen Sie doch diesen Brief für mich ein".
Wie reagierst du?
1) "Nein, ich bin nicht Ihr Postbote"
3) "Gerne, wenn Sie möchten"
Drücke die entsprechende Taste

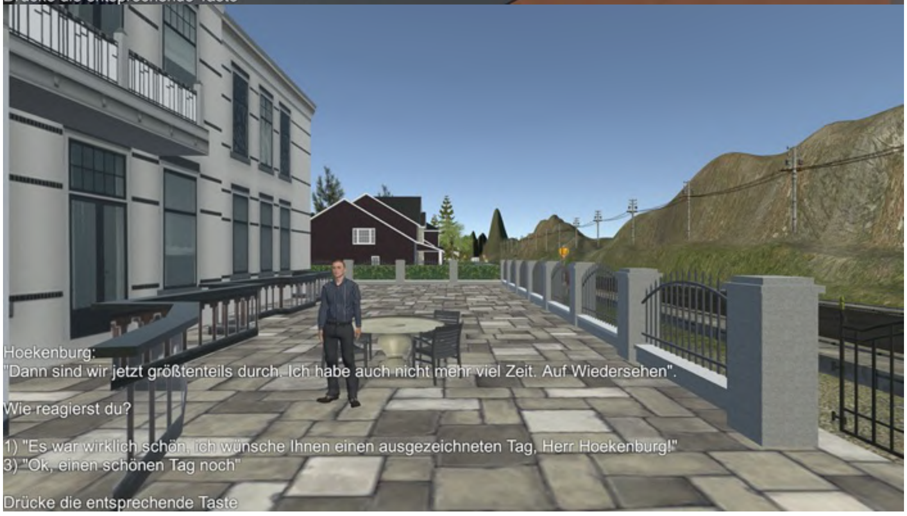
Hoekenburg:
"Dann sind wir jetzt größtenteils durch. Ich habe auch nicht mehr viel Zeit. Auf Wiedersehen".
Wie reagierst du?
1) "Es war wirklich schön, ich wünsche Ihnen einen ausgezeichneten Tag, Herr Hoekenburg!"
3) "Ok, einen schönen Tag noch"
Drücke die entsprechende Taste

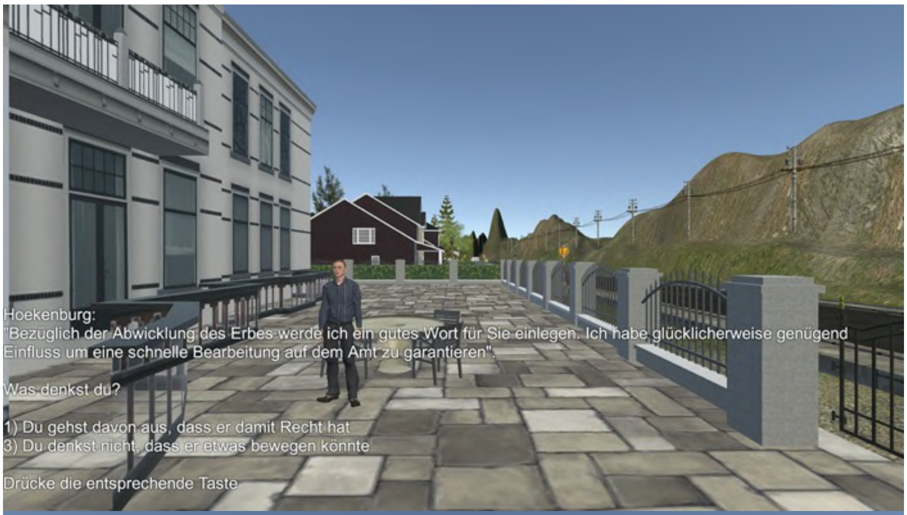
Hoekenburg:
"Bezüglich der Abwicklung des Erbes werde ich ein gutes Wort für Sie einlegen. Ich habe glücklicherweise genügend Einfluss um eine schnelle Bearbeitung auf dem Amt zu garantieren".
Was denkst du?
1) Du gehst davon aus, dass er damit Recht hat
3) Du denkst nicht, dass er etwas bewegen könnte
Drücke die entsprechende Taste

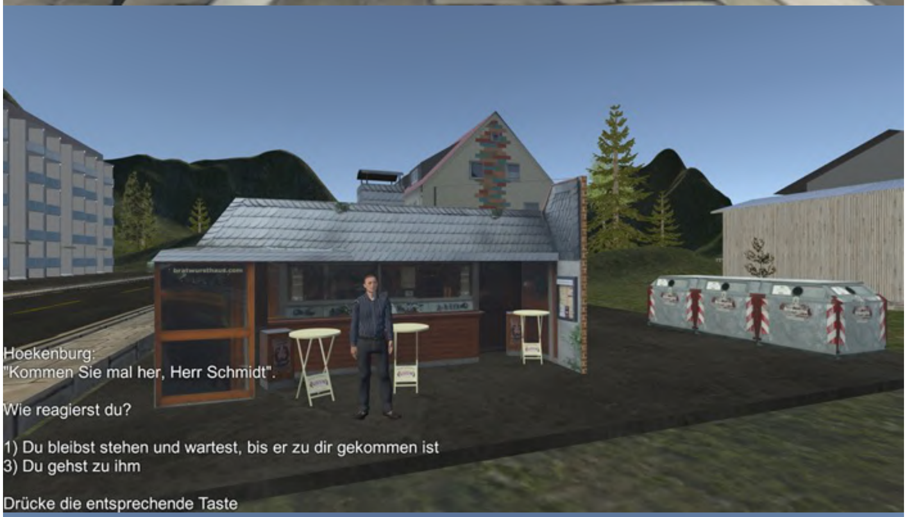
Hoekenburg:
"Kommen Sie mal her, Herr Schmidt".
Wie reagierst du?
1) Du bleibst stehen und wartest, bis er zu dir gekommen ist
3) Du gehst zu ihm
Drücke die entsprechende Taste

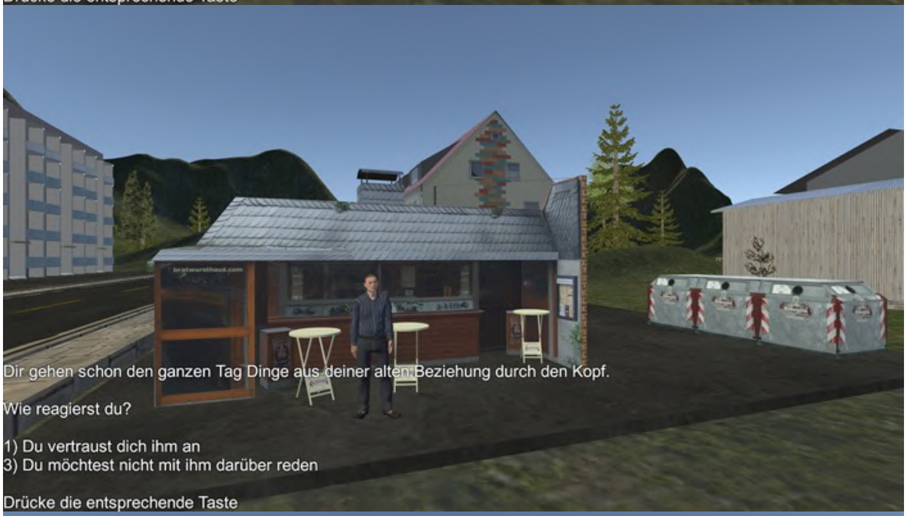
Dir gehen schon den ganzen Tag Dinge aus deiner alten Beziehung durch den Kopf.
Wie reagierst du?
1) Du vertraust dich ihm an
3) Du möchtest nicht mit ihm darüber reden
Drücke die entsprechende Taste

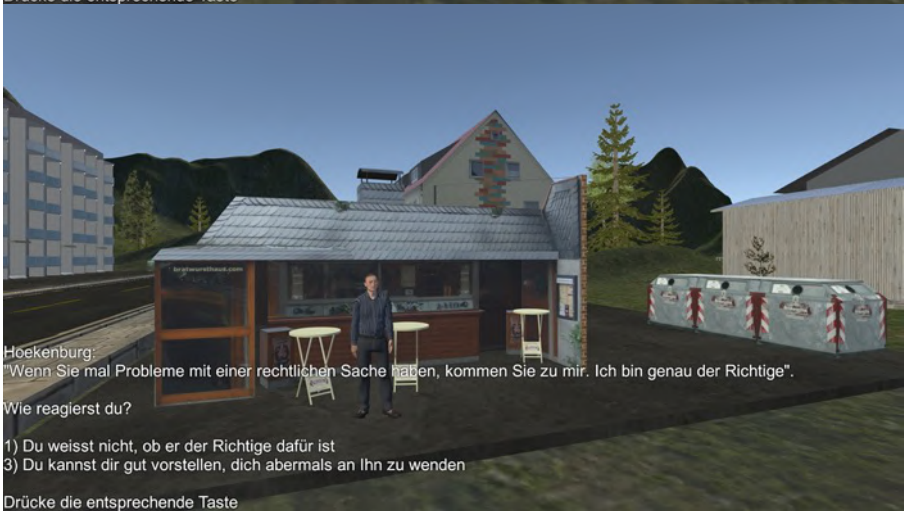
Hoekenburg:
"Wenn Sie mal Probleme mit einer rechtlichen Sache haben, kommen Sie zu mir. Ich bin genau der Richtige".
Wie reagierst du?
1) Du weisst nicht, ob er der Richtige dafür ist
3) Du kannst dir gut vorstellen, dich abermals an Ihn zu wenden
Drücke die entsprechende Taste

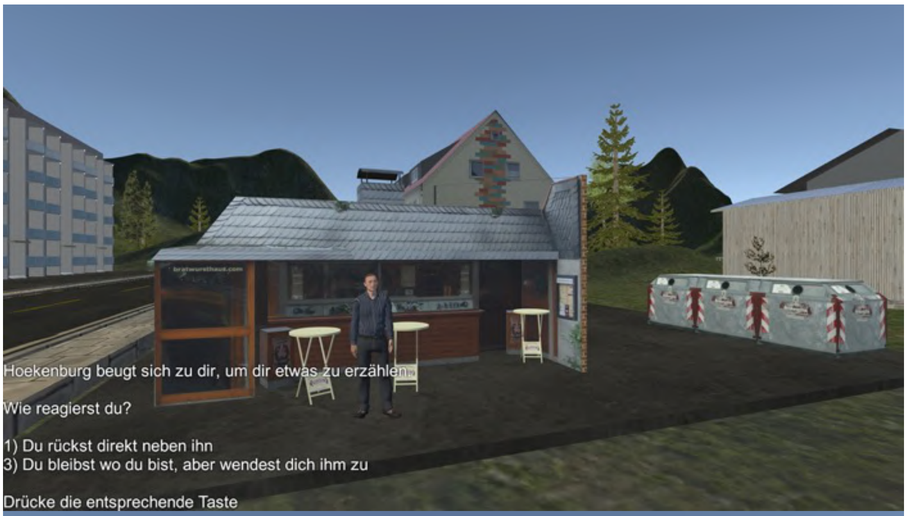
Hoekenburg beugt sich zu dir, um dir etwas zu erzählen
Wie reagierst du?
1) Du rückst direkt neben ihn
3) Du bleibst wo du bist, aber wendest dich ihm zu
Drücke die entsprechende Taste

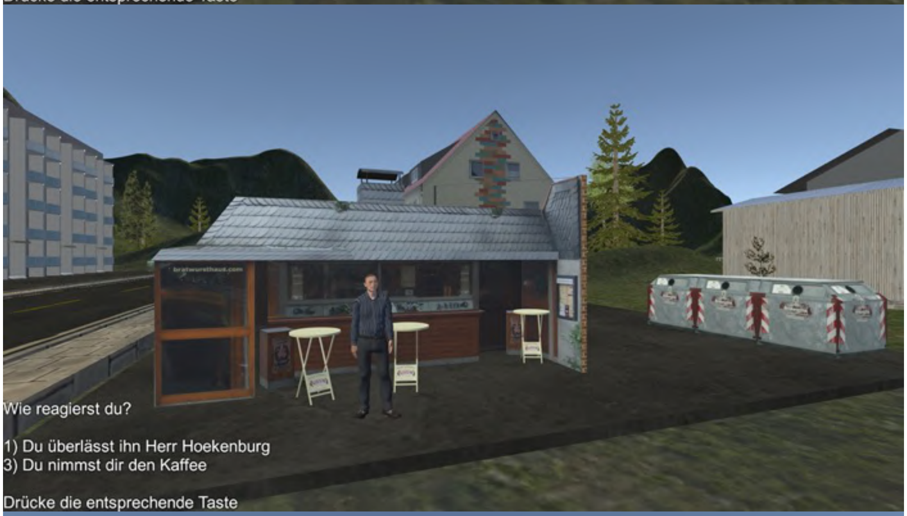
Wie reagierst du?
1) Du überlässt ihn Herr Hoekenburg
3) Du nimmst dir den Kaffee
Drücke die entsprechende Taste

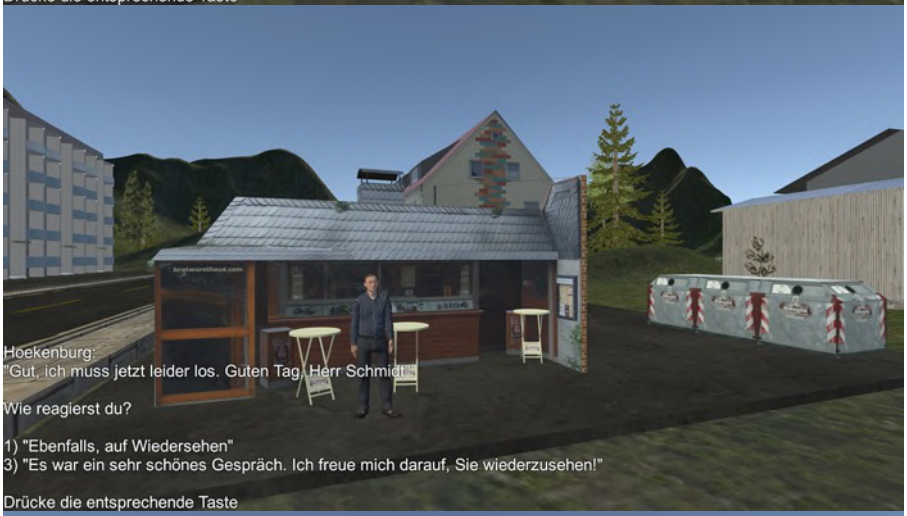
Hoekenburg:
"Gut, ich muss jetzt leider los. Guten Tag, Herr Schmidt."
Wie reagierst du?
1) "Ebenfalls, auf Wiedersehen"
3) "Es war ein sehr schönes Gespräch. Ich freue mich darauf, Sie wiederzusehen!"
Drücke die entsprechende Taste

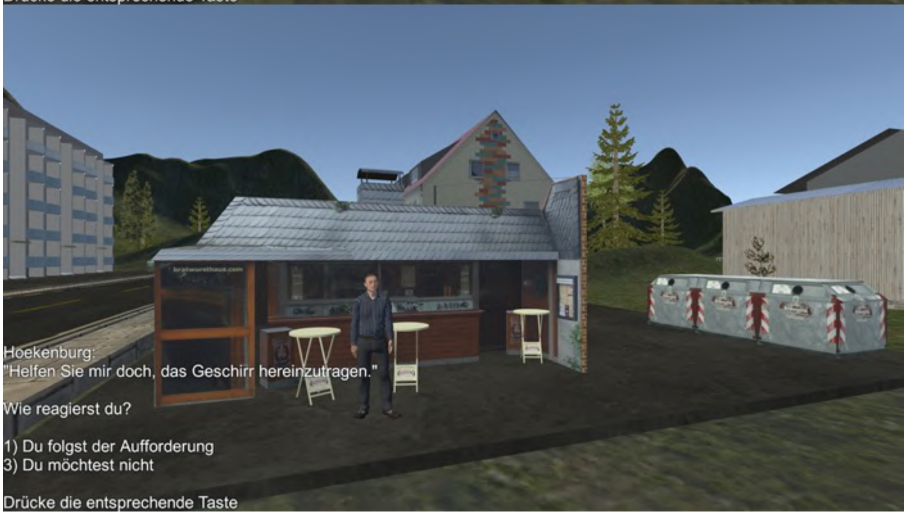
Hoekenburg:
"Helfen Sie mir doch, das Geschirr hereinzutragen."
Wie reagierst du?
1) Du folgst der Aufforderung
3) Du möchtest nicht
Drücke die entsprechende Taste

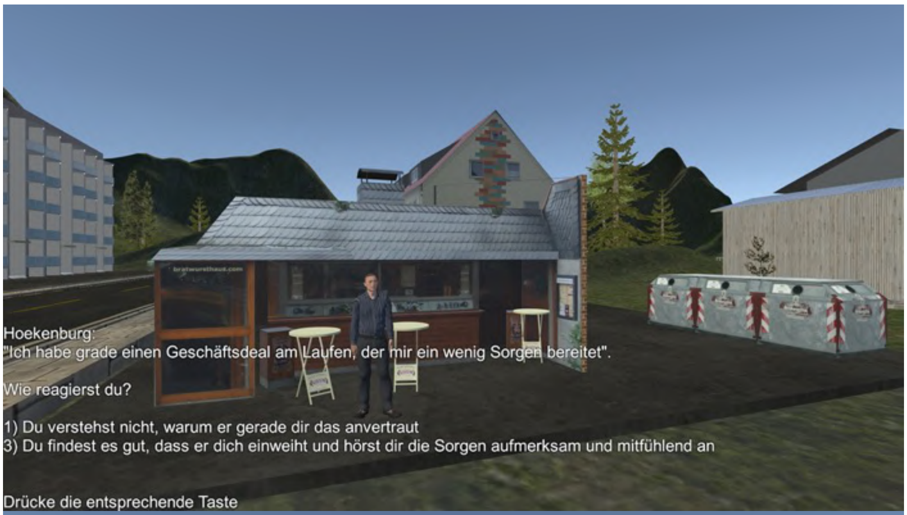
Hoekenburg:
"Ich habe grade einen Geschäftsdeal am Laufen, der mir ein wenig Sorgen bereitet".
Wie reagierst du?
1) Du verstehst nicht, warum er gerade dir das anvertraut
3) Du findest es gut, dass er dich einweiht und hörst dir die Sorgen aufmerksam und mitfühlend an
Drücke die entsprechende Taste

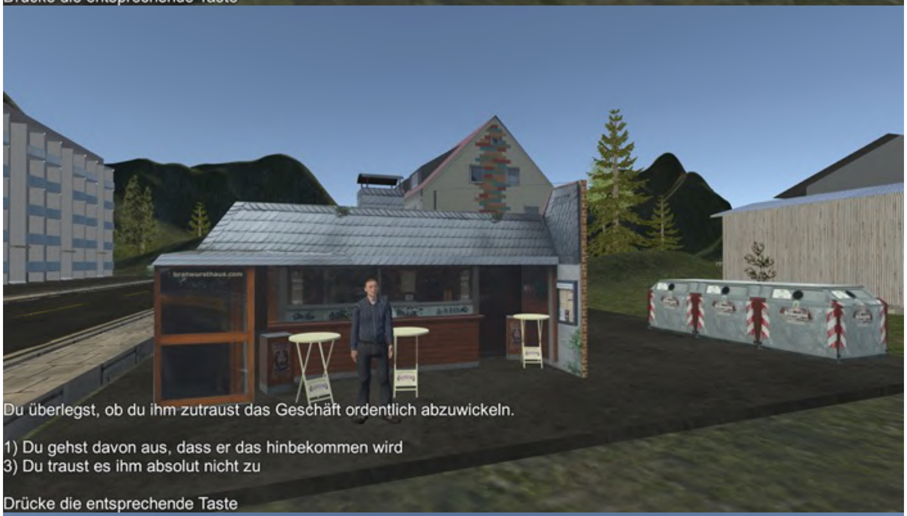
Du überlegst, ob du ihm zutraust das Geschäft ordentlich abzuwickeln.
1) Du gehst davon aus, dass er das hinbekommen wird
3) Du traust es ihm absolut nicht zu
Drücke die entsprechende Taste

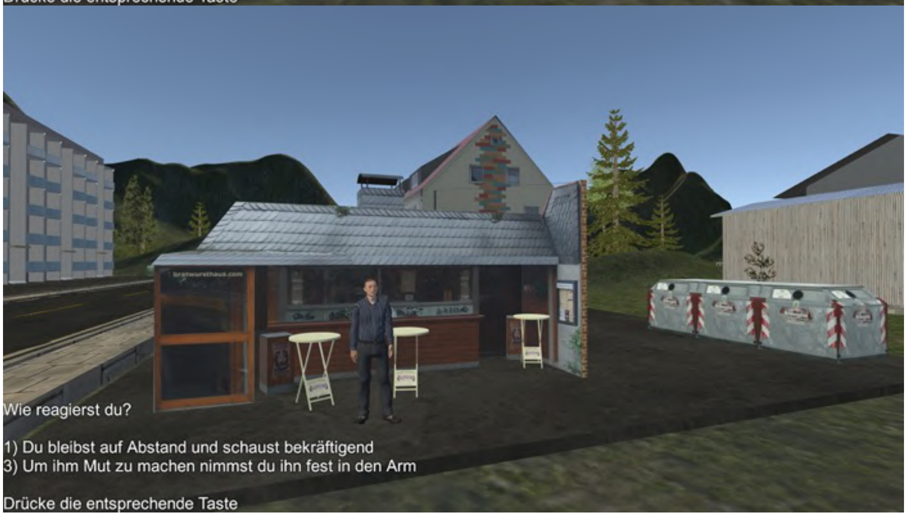
Wie reagierst du?
1) Du bleibst auf Abstand und schaust bekräftigend
3) Um ihm Mut zu machen nimmst du ihn fest in den Arm
Drücke die entsprechende Taste

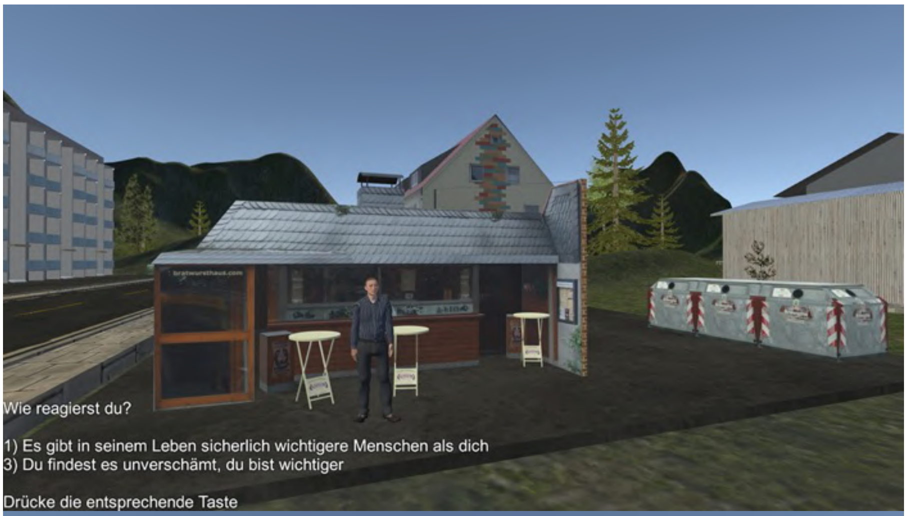
Wie reagierst du?
1) Es gibt in seinem Leben sicherlich wichtigere Menschen als dich
3) Du findest es unverschämt, du bist wichtiger
Drücke die entsprechende Taste

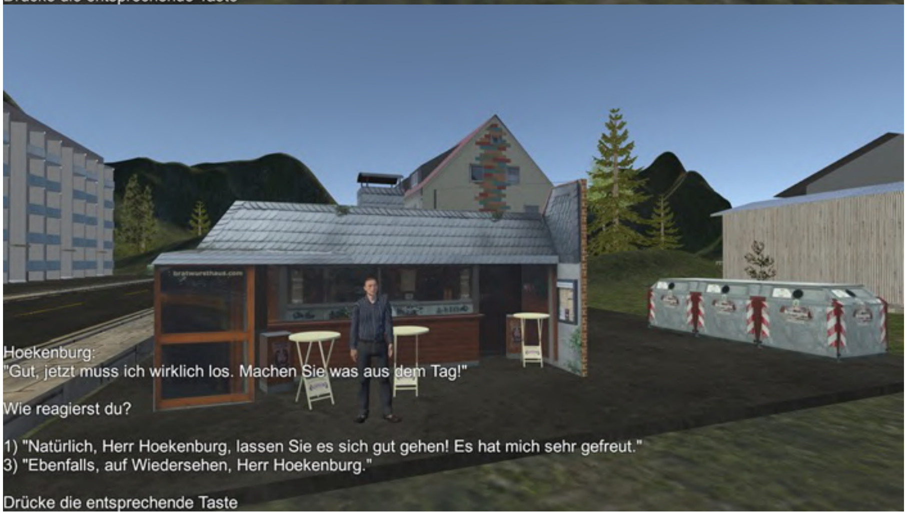
Hoekenburg:
"Gut, jetzt muss ich wirklich los. Machen Sie was aus dem Tag!"
Wie reagierst du?
1) "Natürlich, Herr Hoekenburg, lassen Sie es sich gut gehen! Es hat mich sehr gefreut."
3) "Ebenfalls, auf Wiedersehen, Herr Hoekenburg."
Drücke die entsprechende Taste

S5.1.3 Berger (Low-Power/High-Affiliation, Updating-Avatar)

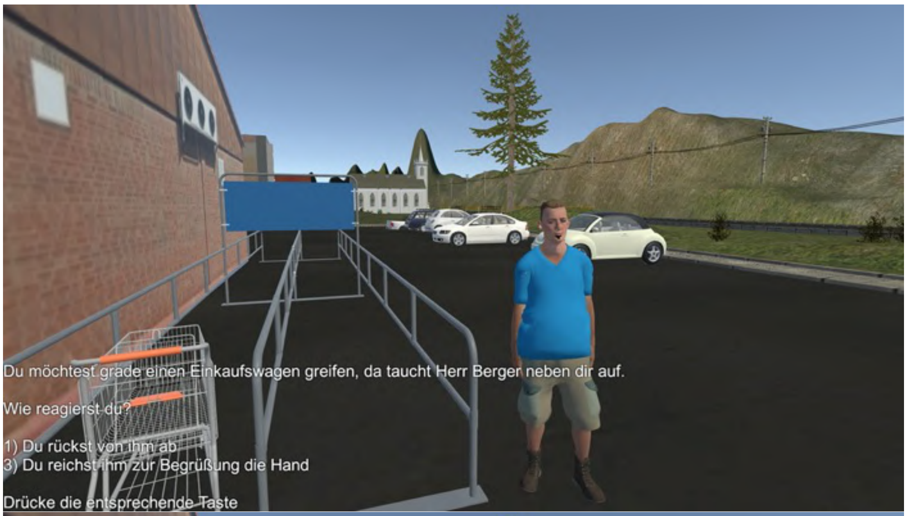

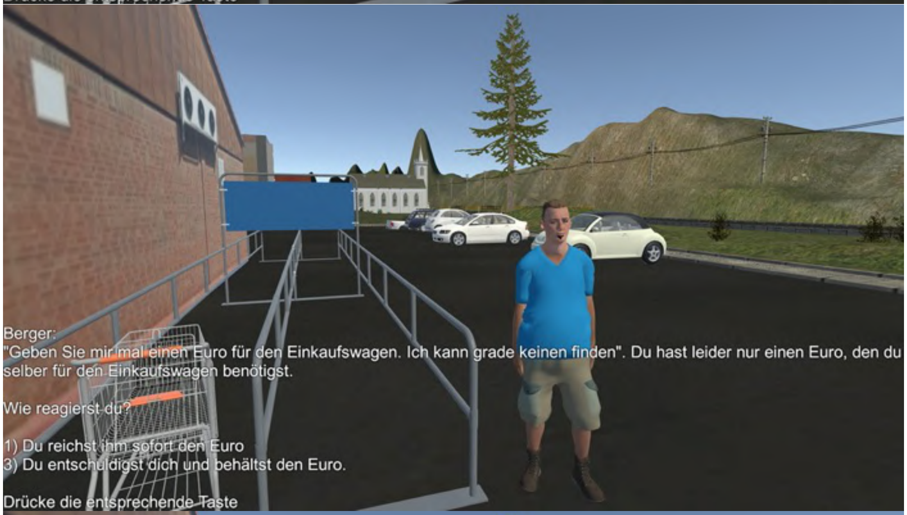

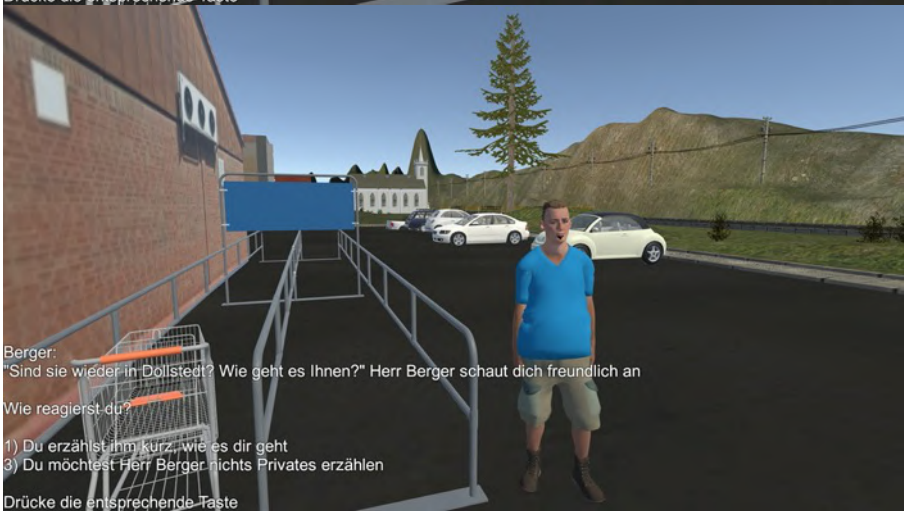

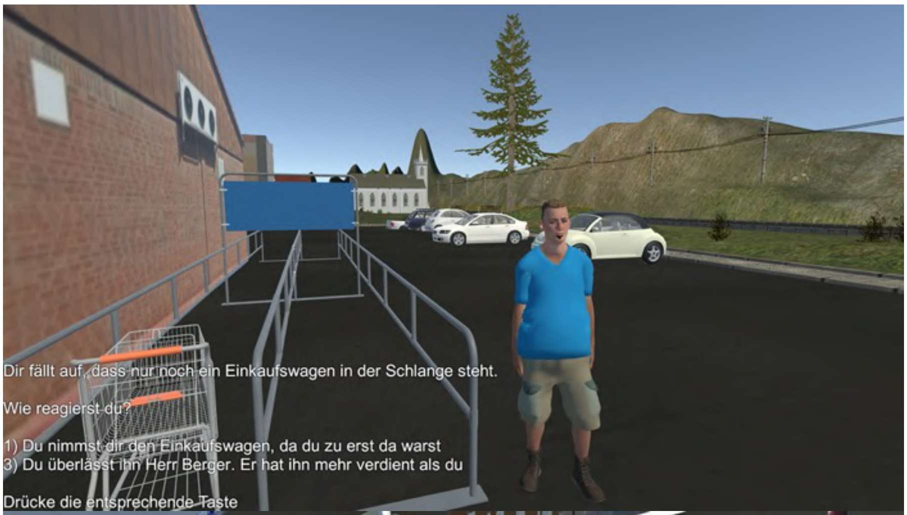
Dir fällt auf, dass nur noch ein Einkaufswagen in der Schlange steht.
Wie reagierst du?
1) Du nimmst dir den Einkaufswagen, da du zu erst da warst
3) Du überlässt ihn Herr Berger. Er hat ihn mehr verdient als du
Drücke die entsprechende Taste

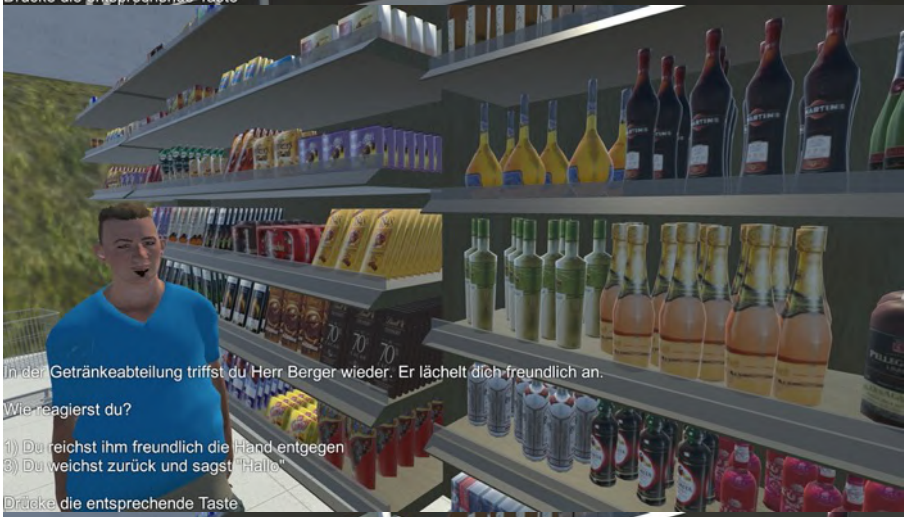
In der Getränkeabteilung triffst du Herr Berger wieder. Er lächelt dich freundlich an.
Wie reagierst du?
1) Du reichst ihm freundlich die Hand entgegen
3) Du weichst zurück und sagst "Hallo"
Drücke die entsprechende Taste

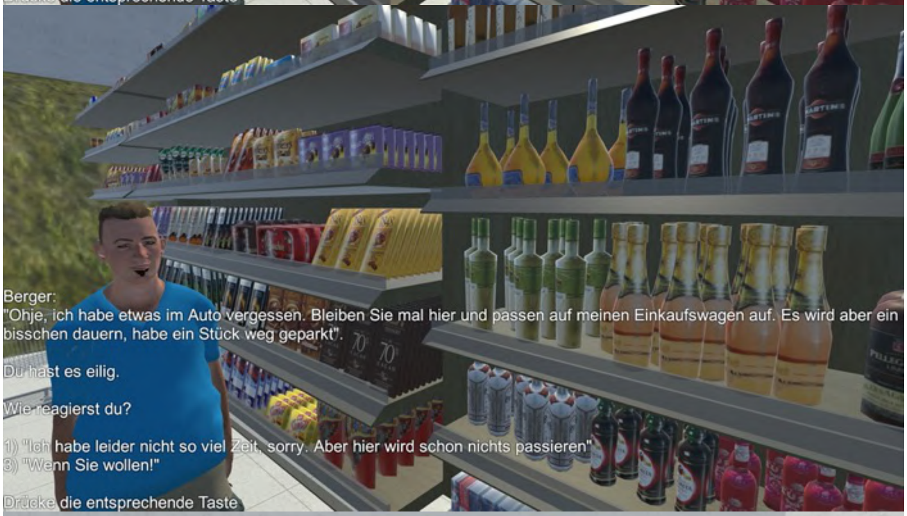
Berger:
"Ohje, ich habe etwas im Auto vergessen. Bleiben Sie mal hier und passen auf meinen Einkaufswagen auf. Es wird aber ein bisschen dauern, habe ein Stück weg geparkt".
Du hast es eilig.
Wie reagierst du?
1) "Ich habe leider nicht so viel Zeit, sorry. Aber hier wird schon nichts passieren"
3) "Wenn Sie wollen!"
Drücke die entsprechende Taste

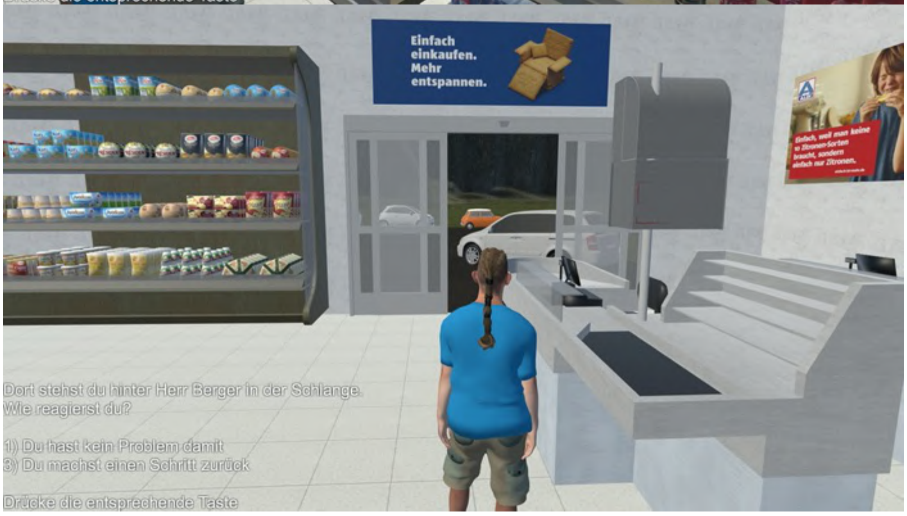
Einfach einkaufen. Mehr entspannen.
Dort stehst du hinter Herr Berger in der Schlange.
Wie reagierst du?
1) Du hast kein Problem damit
3) Du machst einen Schritt zurück
Drücke die entsprechende Taste

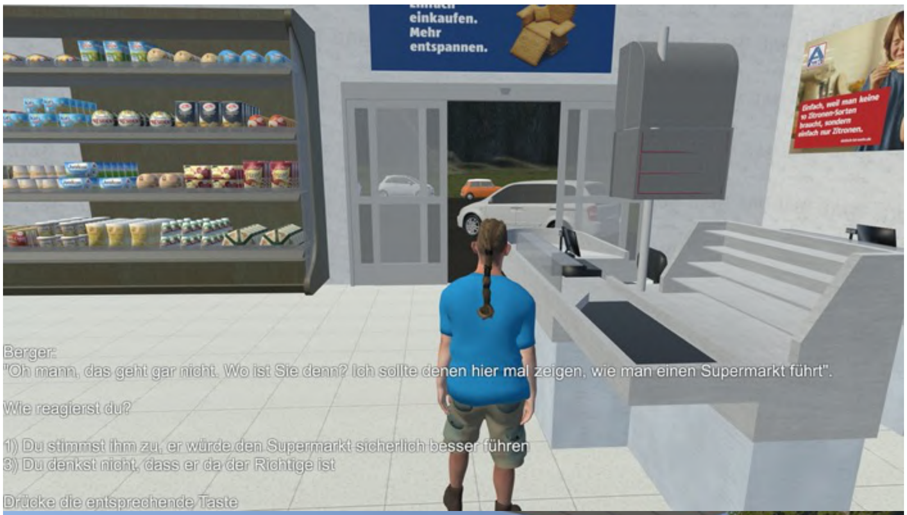
einkaufen.
Mehr
entspannen.
Einfach, weil man keine 10 Zitronen-Sorten braucht, sondern einfach nur Zitronen.
Berger:
"Oh mann, das geht gar nicht. Wo ist Sie denn? Ich sollte denen hier mal zeigen, wie man einen Supermarkt führt".
Wie reagierst du?
1) Du stimmst ihm zu, er würde den Supermarkt sicherlich besser führen
3) Du denkst nicht, dass er da der Richtige ist
Drücke die entsprechende Taste

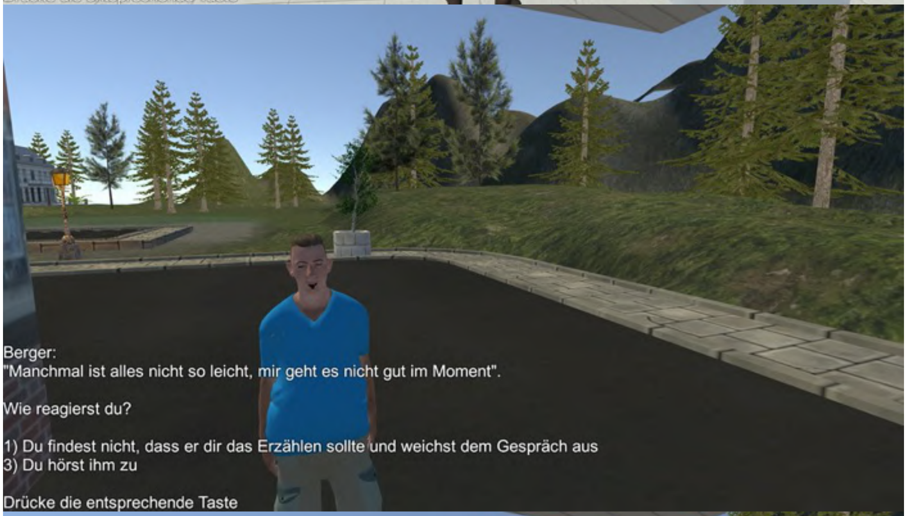
Berger:
"Manchmal ist alles nicht so leicht, mir geht es nicht gut im Moment".
Wie reagierst du?
1) Du findest nicht, dass er dir das Erzählen sollte und weichst dem Gespräch aus
3) Du hörst ihm zu
Drücke die entsprechende Taste

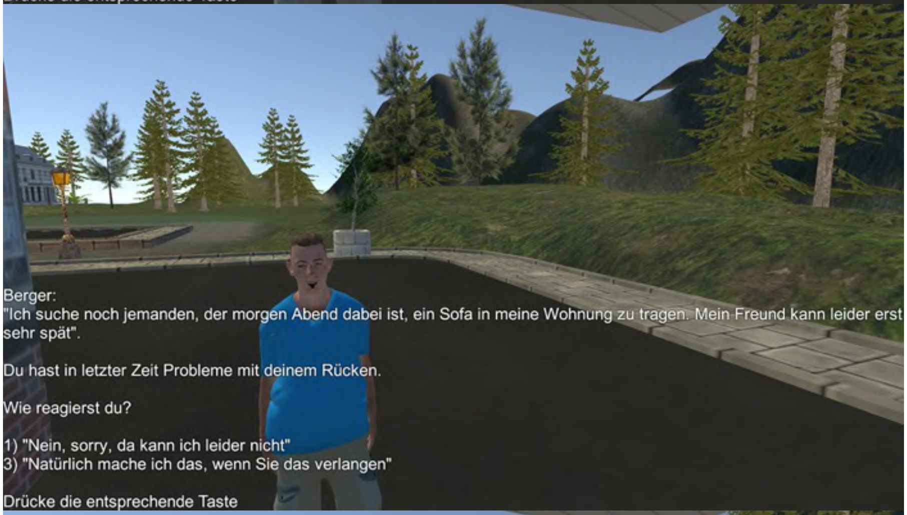
Berger:
"Ich suche noch jemanden, der morgen Abend dabei ist, ein Sofa in meine Wohnung zu tragen. Mein Freund kann leider erst sehr spät".
Du hast in letzter Zeit Probleme mit deinem Rücken.
Wie reagierst du?
1) "Nein, sorry, da kann ich leider nicht"
3) "Natürlich mache ich das, wenn Sie das verlangen"
Drücke die entsprechende Taste

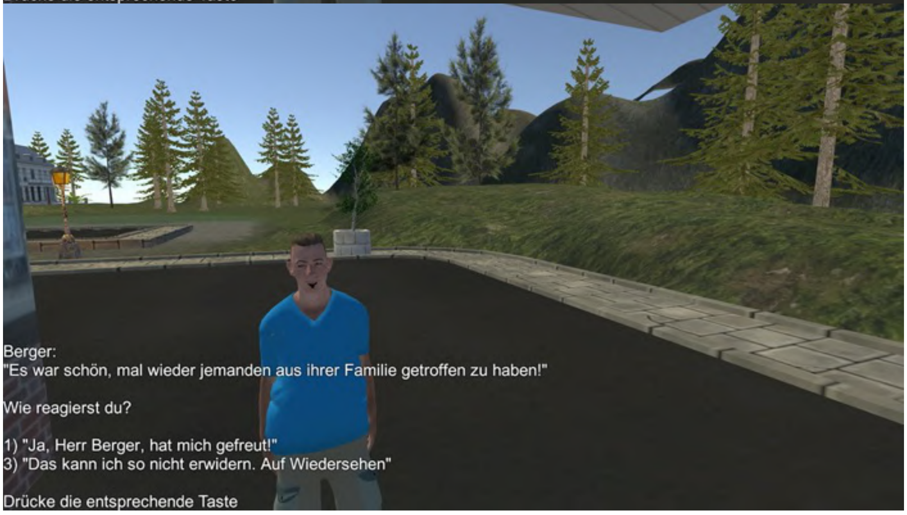
Berger:
"Es war schön, mal wieder jemanden aus ihrer Familie getroffen zu haben!"
Wie reagierst du?
1) "Ja, Herr Berger, hat mich gefreut!"
3) "Das kann ich so nicht erwidern. Auf Wiedersehen"
Drücke die entsprechende Taste

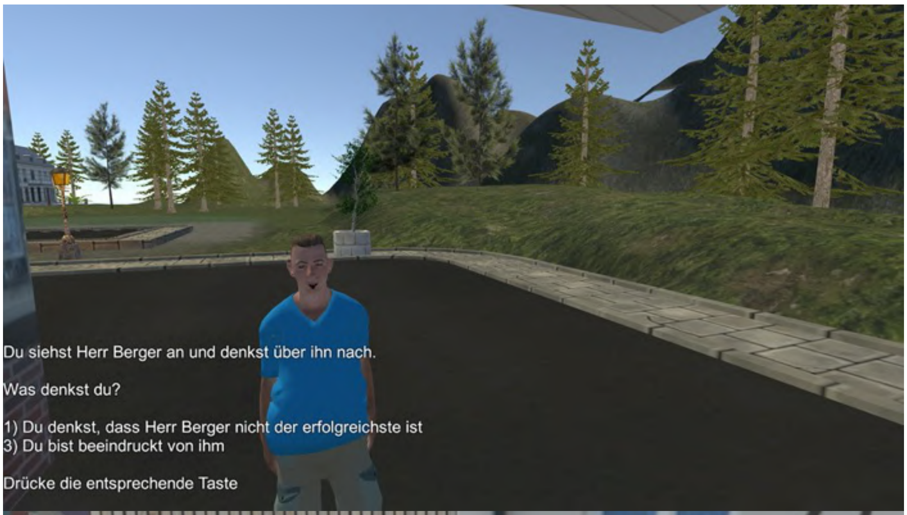
Du siehst Herr Berger an und denkst über ihn nach.
Was denkst du?
1) Du denkst, dass Herr Berger nicht der erfolgreichste ist
3) Du bist beeindruckt von ihm
Drücke die entsprechende Taste

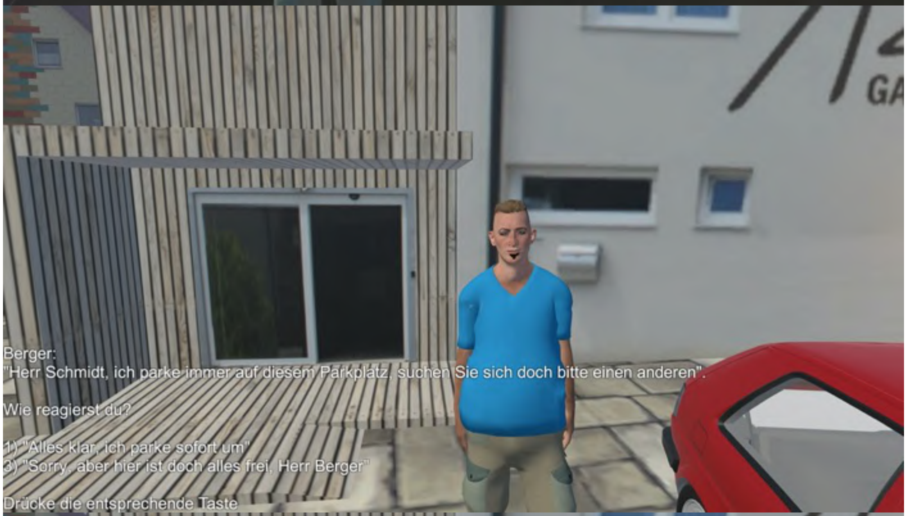
Berger:
"Herr Schmidt, ich parke immer auf diesem Parkplatz, suchen Sie sich doch bitte einen anderen".
Wie reagierst du?
1) "Alles klar, ich parke sofort um"
3) "Sorry, aber hier ist doch alles frei, Herr Berger"
Drücke die entsprechende Taste

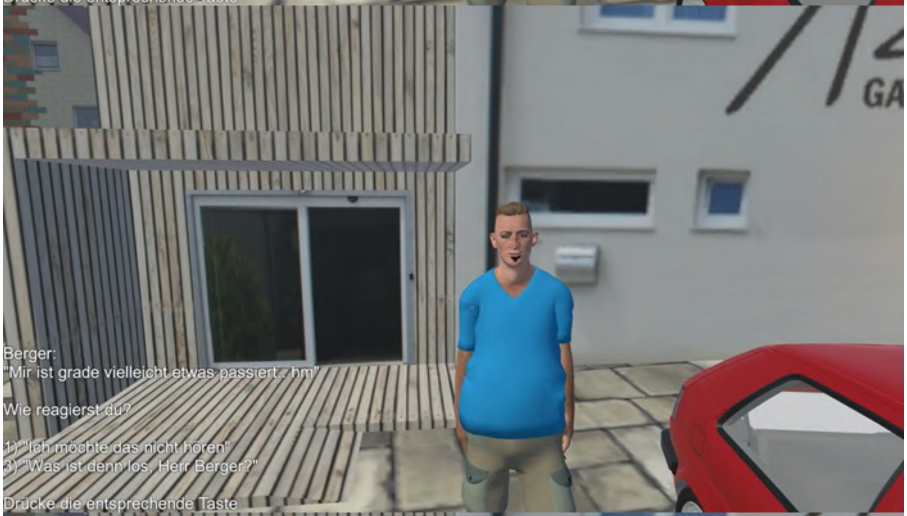
Berger:
"Mir ist grade vielleicht etwas passiert...hm"
Wie reagierst du?
1) "Ich möchte das nicht hören"
3) "Was ist denn los, Herr Berger?"
Drücke die entsprechende Taste

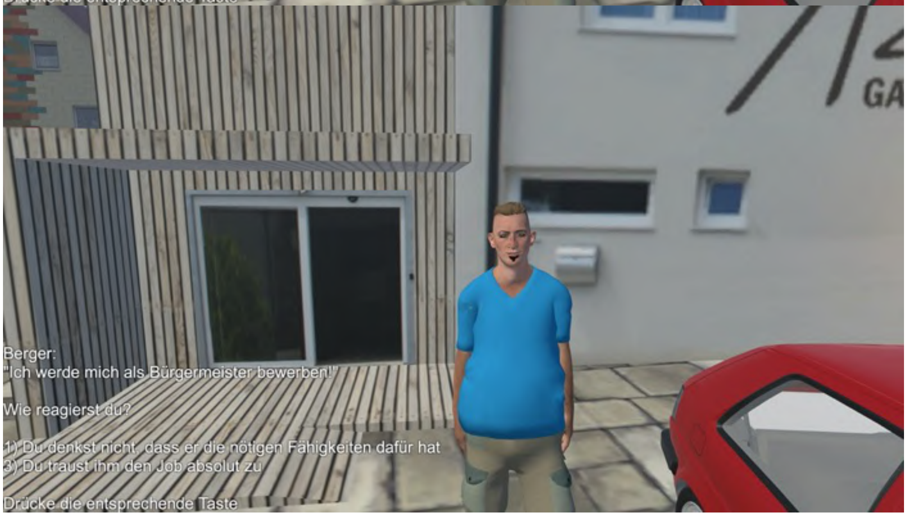
Berger:
"Ich werde mich als Bürgermeister bewerben!"
Wie reagierst du?
1) Du denkst nicht, dass er die nötigen Fähigkeiten dafür hat
3) Du traust ihm den Job absolut zu
Drücke die entsprechende Taste

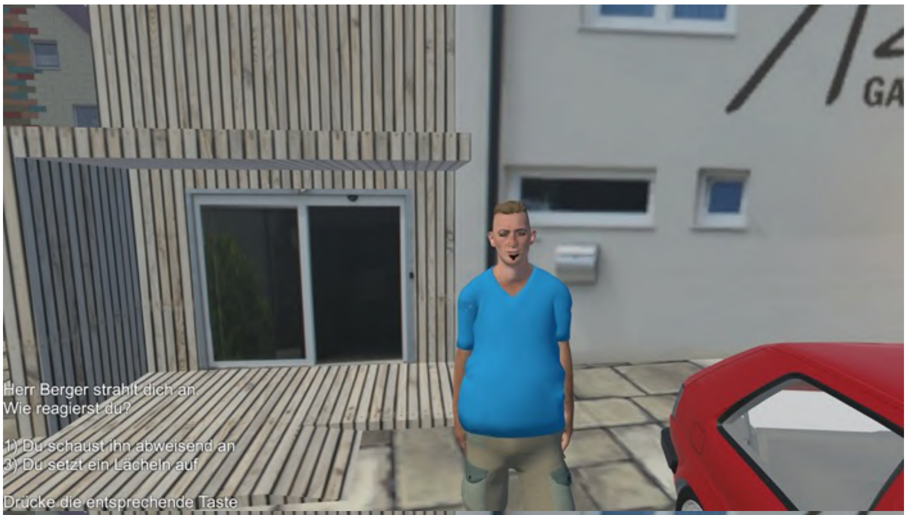
Herr Berger strahlt dich an
Wie reagierst du?
1) Du schaust ihn abweisend an
3) Du setzt ein Lächeln auf
Drücke die entsprechende Taste

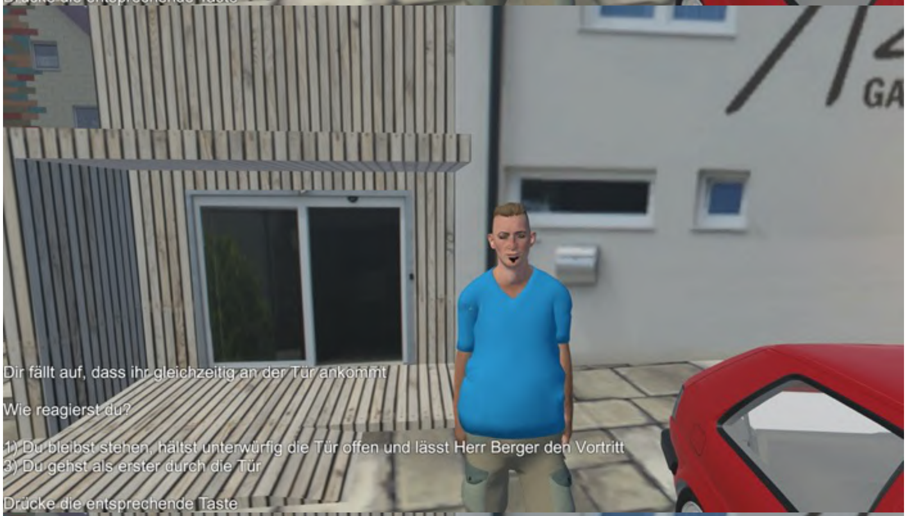
Dir fällt auf, dass ihr gleichzeitig an der Tür ankommt
Wie reagierst du?
1) Du bleibst stehen, hältst unterwürfig die Tür offen und lässt Herr Berger den Vortritt
3) Du gehst als erster durch die Tür
Drücke die entsprechende Taste

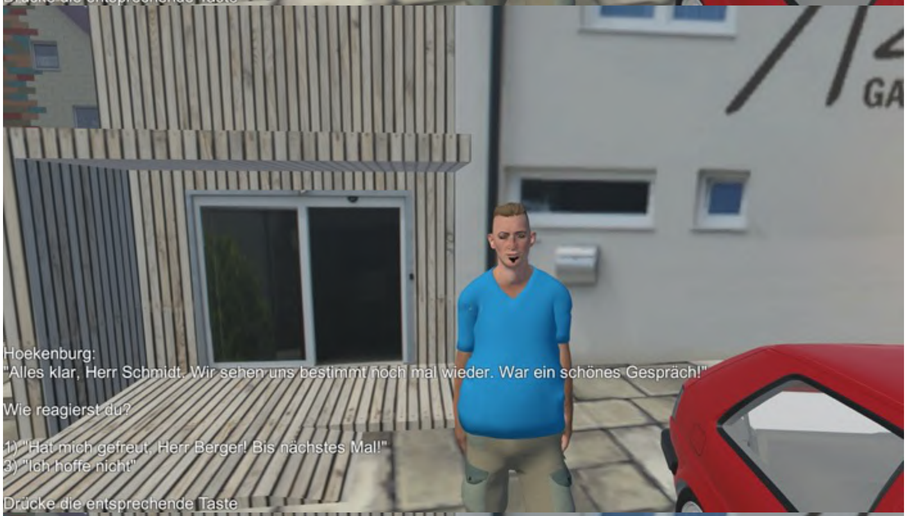
Hoekenburg:
"Alles klar, Herr Schmidt. Wir sehen uns bestimmt noch mal wieder. War ein schönes Gespräch!"
Wie reagierst du?
1) "Hat mich gefreut, Herr Berger! Bis nächstes Mal!"
3) "Ich hoffe nicht"
Drücke die entsprechende Taste

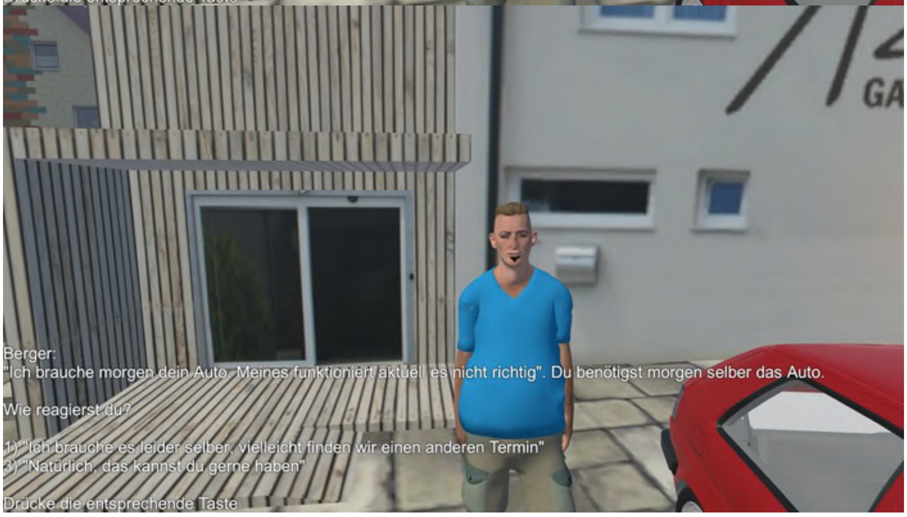
Berger:
"Ich brauche morgen dein Auto. Meines funktioniert aktuell es nicht richtig". Du benötigst morgen selber das Auto.
Wie reagierst du?
1) "Ich brauche es leider selber, vielleicht finden wir einen anderen Termin"
3) "Natürlich, das kannst du gerne haben"
Drücke die entsprechende Taste

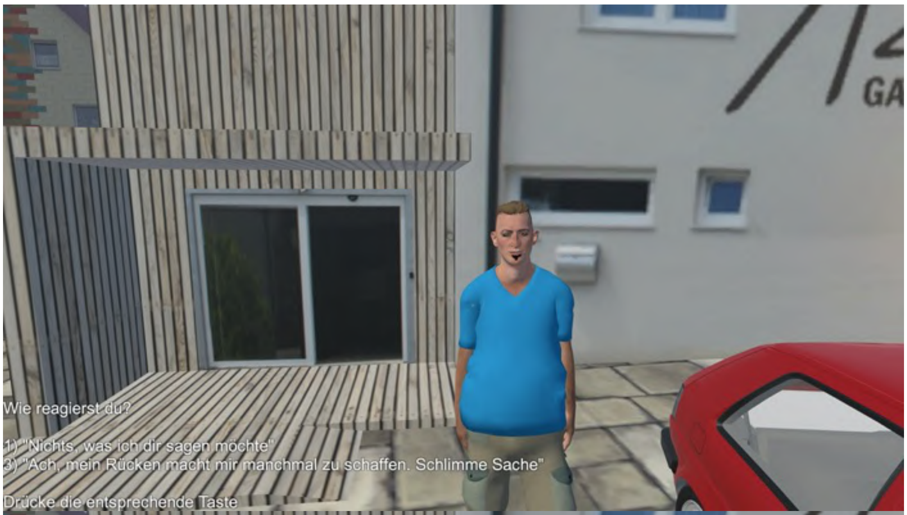
Wie reagierst du?
1) "Nichts, was ich dir sagen möchte"
3) "Ach, mein Rücken macht mir manchmal zu schaffen. Schlimme Sache"
Drücke die entsprechende Taste

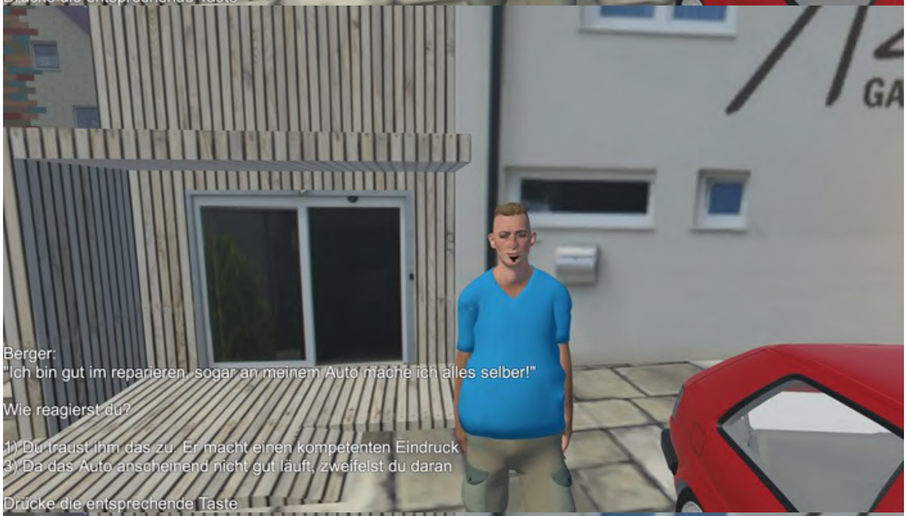
Berger:
"Ich bin gut im reparieren, sogar an meinem Auto mache ich alles selber!"
Wie reagierst du?
1) Du traust ihm das zu. Er macht einen kompetenten Eindruck
3) Da das Auto anscheinend nicht gut läuft, zweifelst du daran
Drücke die entsprechende Taste

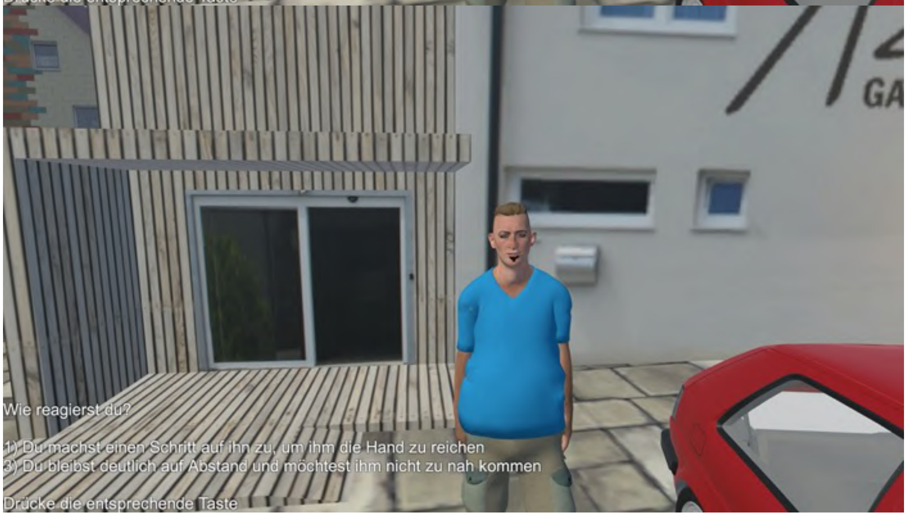
Wie reagierst du?
1) Du machst einen Schritt auf ihn zu, um ihm die Hand zu reichen
3) Du bleibst deutlich auf Abstand und möchtest ihm nicht zu nah kommen
Drücke die entsprechende Taste

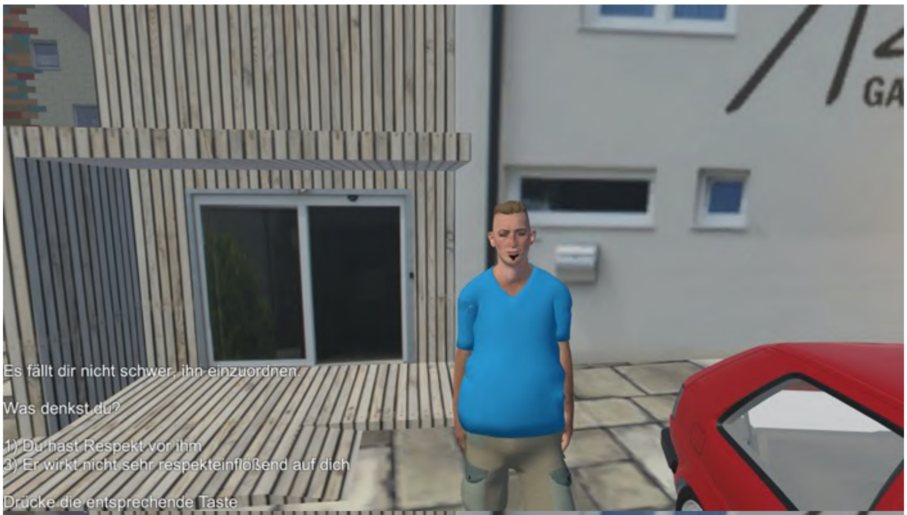
Es fällt dir nicht schwer, ihn einzuordnen.
Was denkst du?
1) Du hast Respekt vor ihm
3) Er wirkt nicht sehr respekteinflößend auf dich
Drücke die entsprechende Taste

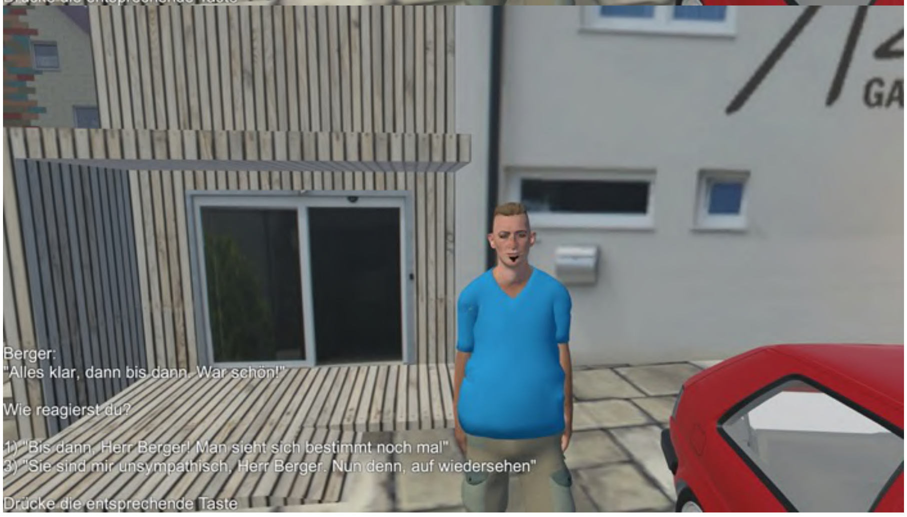
Berger:
"Alles klar, dann bis dann. War schön!"
Wie reagierst du?
1) "Bis dann, Herr Berger! Man sieht sich bestimmt noch mal"
3) "Sie sind mir unsympathisch, Herr Berger. Nun denn, auf wiedersehen"
Drücke die entsprechende Taste

S5.1.4 Karoske (Low-Power/High-Affiliation, Remapping-Avatar)

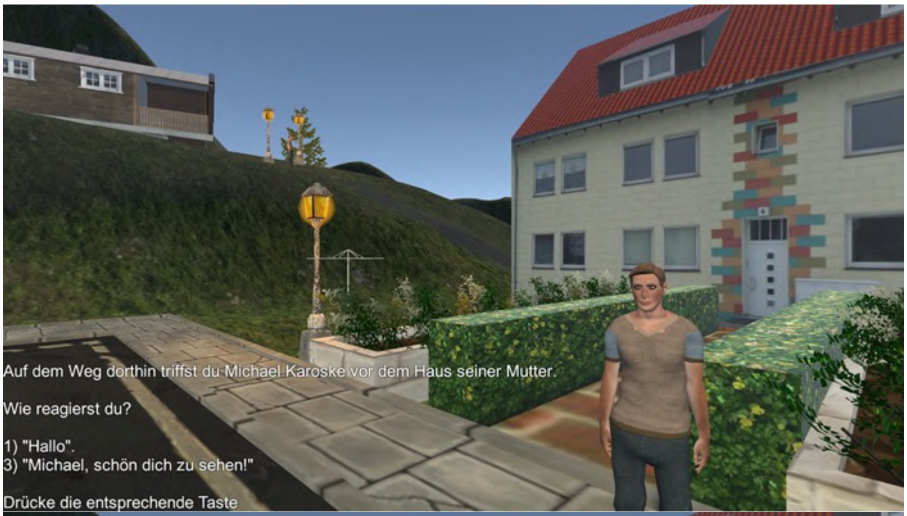

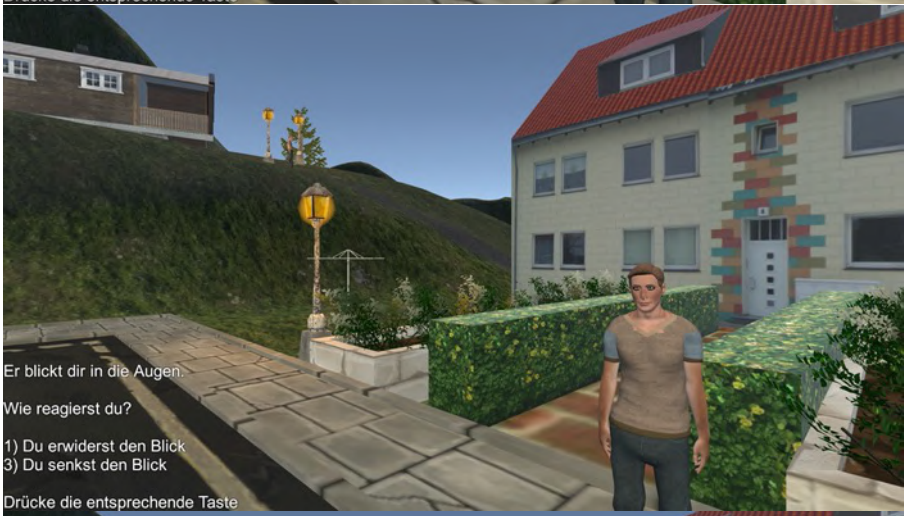

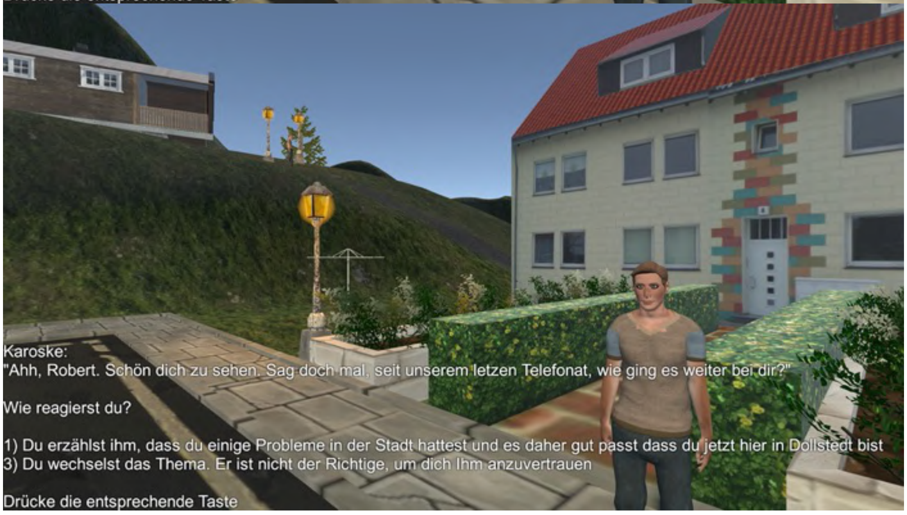

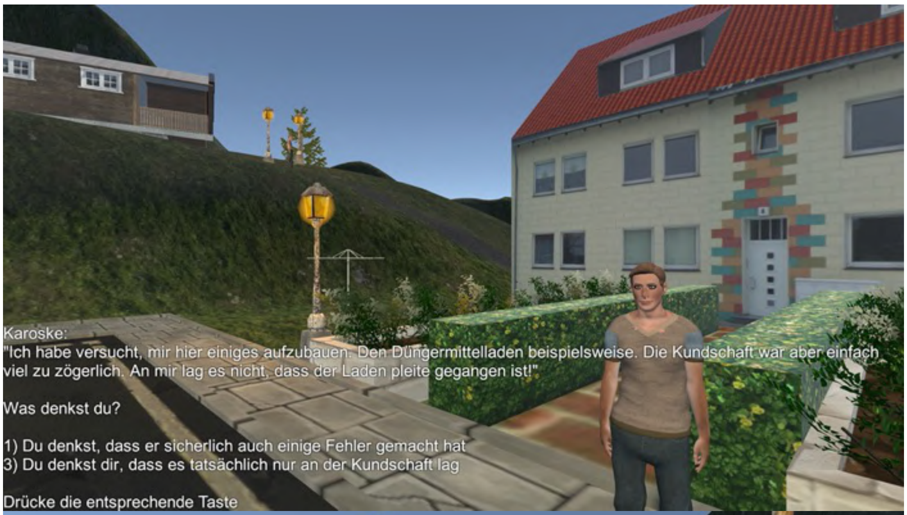
Karoske:
"Ich habe versucht, mir hier einiges aufzubauen. Den Düngermittelladen beispielsweise. Die Kundschaft war aber einfach viel zu zögerlich. An mir lag es nicht, dass der Laden pleite gegangen ist!"
Was denkst du?
1) Du denkst, dass er sicherlich auch einige Fehler gemacht hat
3) Du denkst dir, dass es tatsächlich nur an der Kundschaft lag
Drücke die entsprechende Taste

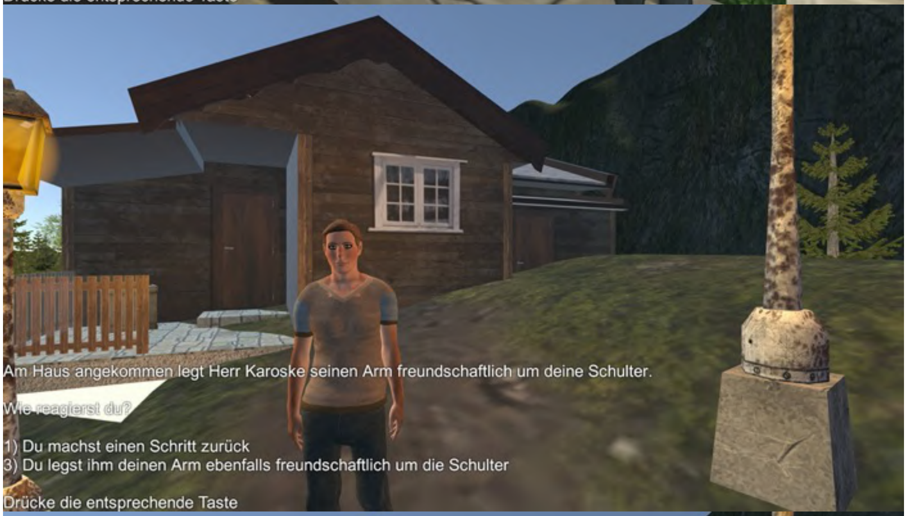
Am Haus angekommen legt Herr Karoske seinen Arm freundschaftlich um deine Schulter.
Wie reagierst du?
1) Du machst einen Schritt zurück
3) Du legst ihm deinen Arm ebenfalls freundschaftlich um die Schulter
Drücke die entsprechende Taste

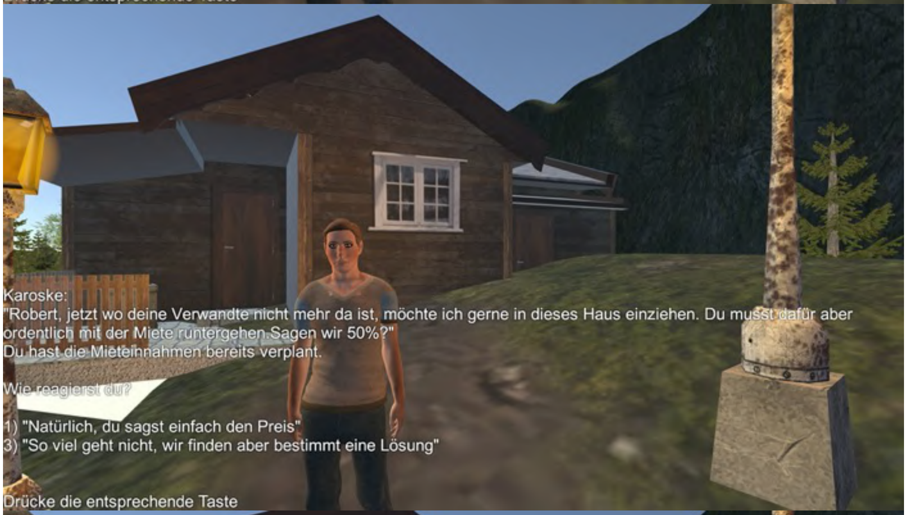
Karoske:
"Robert, jetzt wo deine Verwandte nicht mehr da ist, möchte ich gerne in dieses Haus einziehen. Du musst dafür aber ordentlich mit der Miete runtergehen.Sagen wir 50%?"
Du hast die Mieteinnahmen bereits verplant.
Wie reagierst du?
1) "Natürlich, du sagst einfach den Preis"
3) "So viel geht nicht, wir finden aber bestimmt eine Lösung"
Drücke die entsprechende Taste

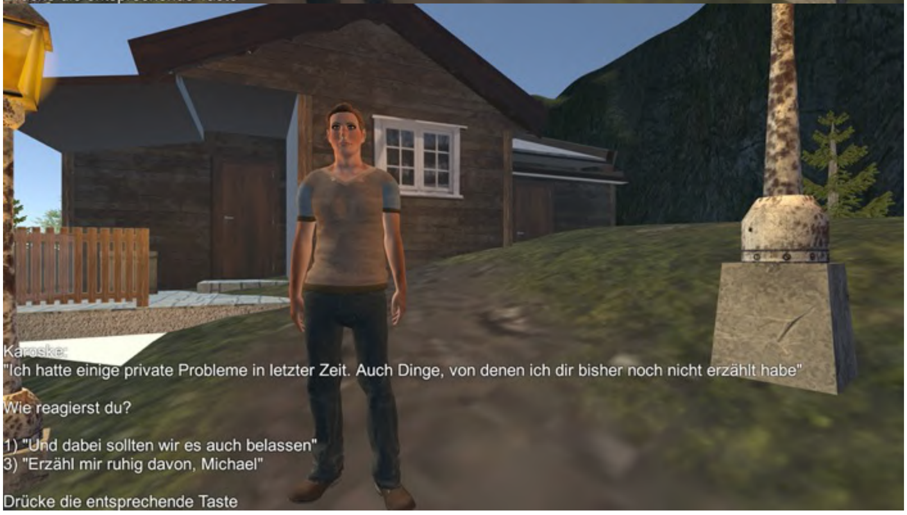
Karoske:
"Ich hatte einige private Probleme in letzter Zeit. Auch Dinge, von denen ich dir bisher noch nicht erzählt habe"
Wie reagierst du?
1) "Und dabei sollten wir es auch belassen"
3) "Erzähl mir ruhig davon, Michael"
Drücke die entsprechende Taste

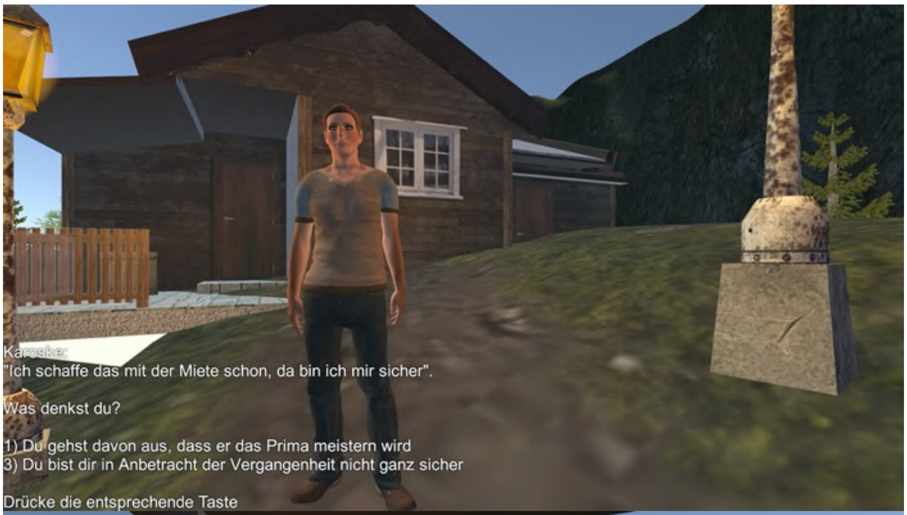
Karoske:
"Ich schaffe das mit der Miete schon, da bin ich mir sicher".
Was denkst du?
1) Du gehst davon aus, dass er das Prima meistern wird
3) Du bist dir in Anbetracht der Vergangenheit nicht ganz sicher
Drücke die entsprechende Taste

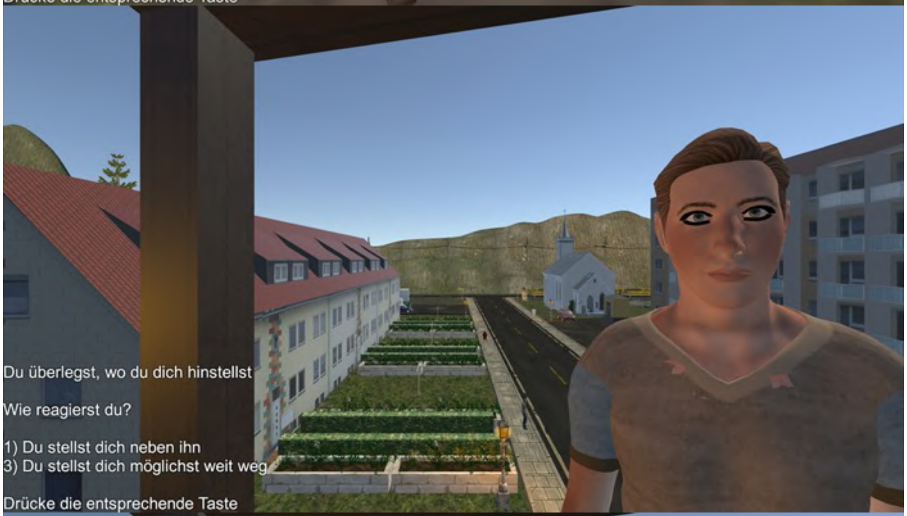
Du überlegst, wo du dich hinstellst
Wie reagierst du?
1) Du stellst dich neben ihn
3) Du stellst dich möglichst weit weg
Drücke die entsprechende Taste

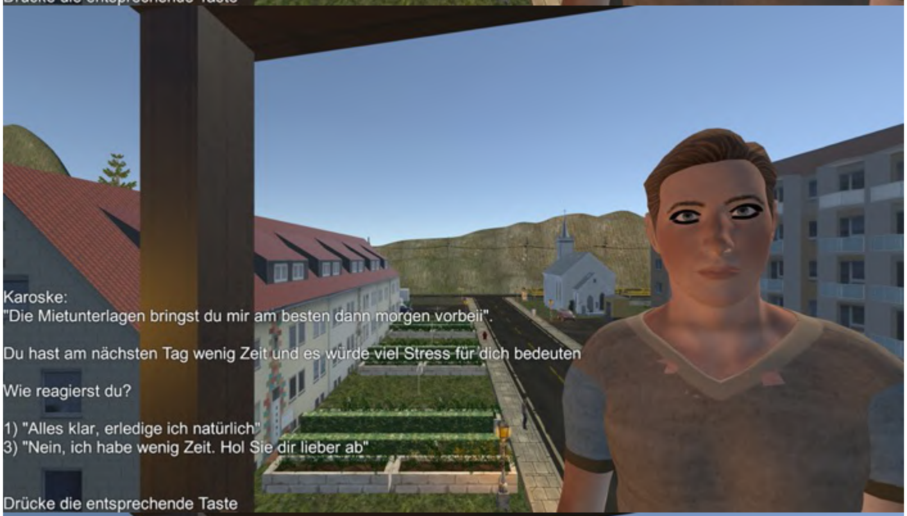
Karoske:
"Die Mietunterlagen bringst du mir am besten dann morgen vorbei".
Du hast am nächsten Tag wenig Zeit und es würde viel Stress für dich bedeuten
Wie reagierst du?
1) "Alles klar, erledige ich natürlich"
3) "Nein, ich habe wenig Zeit. Hol Sie dir lieber ab"
Drücke die entsprechende Taste

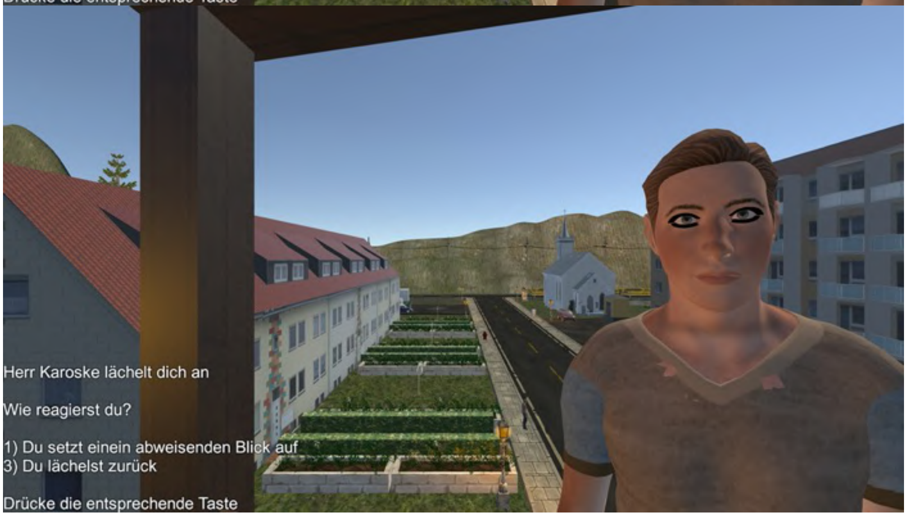
Herr Karoske lächelt dich an
Wie reagierst du?
1) Du setzt einein abweisenden Blick auf
3) Du lächelst zurück
Drücke die entsprechende Taste

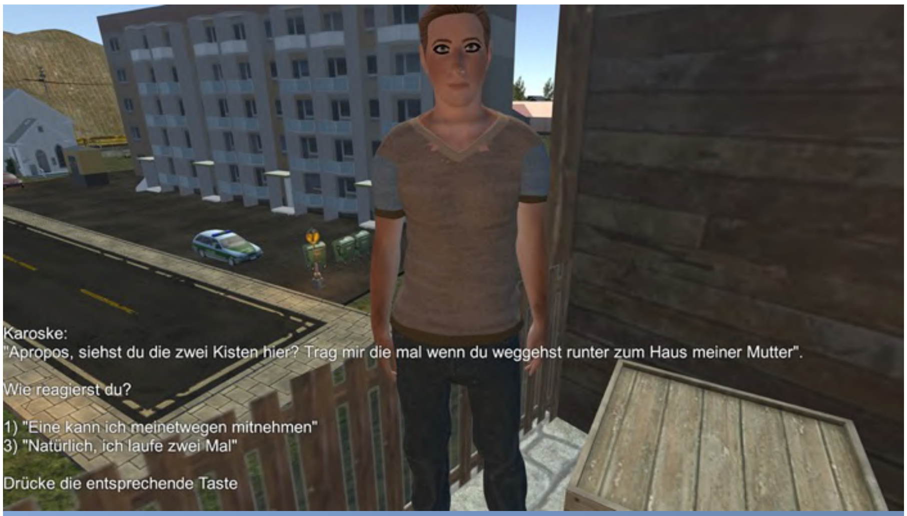
Karoske:
"Apropos, siehst du die zwei Kisten hier? Trag mir die mal wenn du weggehst runter zum Haus meiner Mutter".
Wie reagierst du?
1) "Eine kann ich meinetwegen mitnehmen"
3) "Natürlich, ich laufe zwei Mal"
Drücke die entsprechende Taste

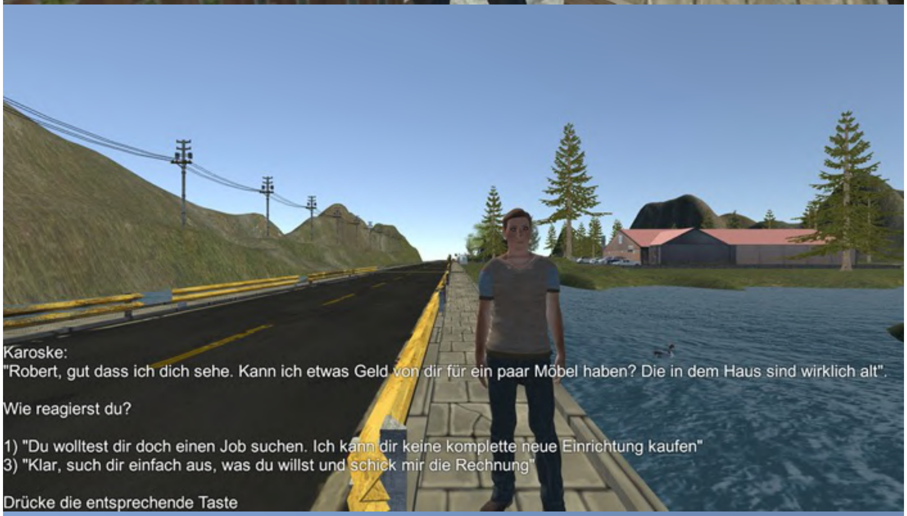
Karoske:
"Robert, gut dass ich dich sehe. Kann ich etwas Geld von dir für ein paar Möbel haben? Die in dem Haus sind wirklich alt".
Wie reagierst du?
1) "Du wolltest dir doch einen Job suchen. Ich kann dir keine komplette neue Einrichtung kaufen"
3) "Klar, such dir einfach aus, was du willst und schick mir die Rechnung"
Drücke die entsprechende Taste

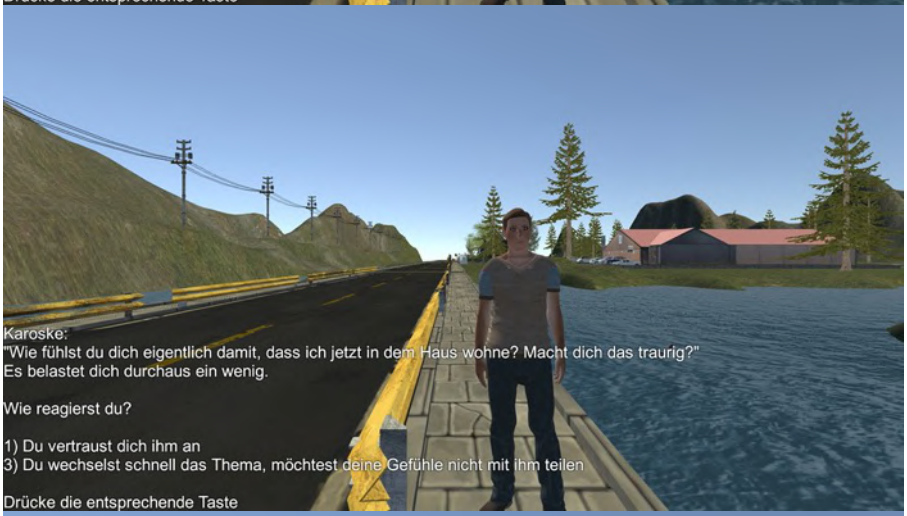
Karoske:
"Wie fühlst du dich eigentlich damit, dass ich jetzt in dem Haus wohne? Macht dich das traurig?"
Es belastet dich durchaus ein wenig.
Wie reagierst du?
1) Du vertraust dich ihm an
3) Du wechselst schnell das Thema, möchtest deine Gefühle nicht mit ihm teilen
Drücke die entsprechende Taste

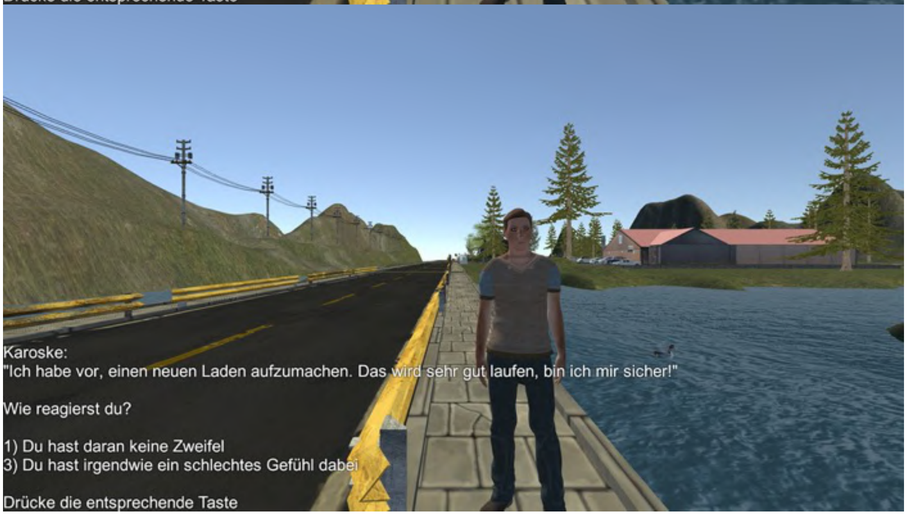
Karoske:
"Ich habe vor, einen neuen Laden aufzumachen. Das wird sehr gut laufen, bin ich mir sicher!"
Wie reagierst du?
1) Du hast daran keine Zweifel
3) Du hast irgendwie ein schlechtes Gefühl dabei
Drücke die entsprechende Taste

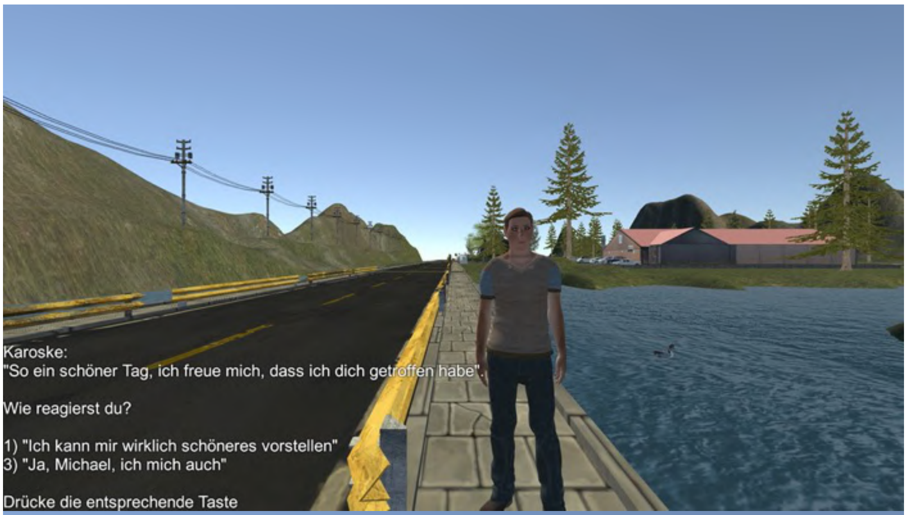
Karoske:
"So ein schöner Tag, ich freue mich, dass ich dich getroffen habe".
Wie reagierst du?
1) "Ich kann mir wirklich schöneres vorstellen"
3) "Ja, Michael, ich mich auch"
Drücke die entsprechende Taste

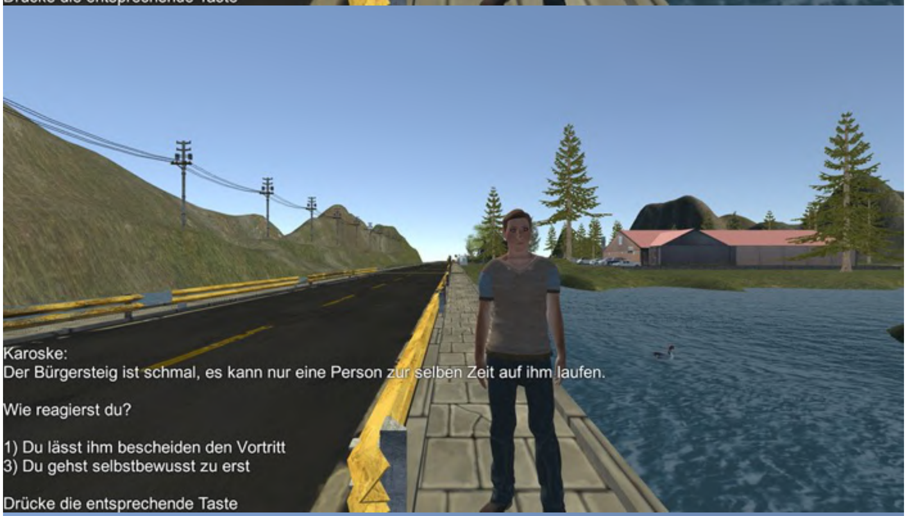
Karoske:
Der Bürgersteig ist schmal, es kann nur eine Person zur selben Zeit auf ihm laufen.
Wie reagierst du?
1) Du lässt ihm bescheiden den Vortritt
3) Du gehst selbstbewusst zu erst
Drücke die entsprechende Taste

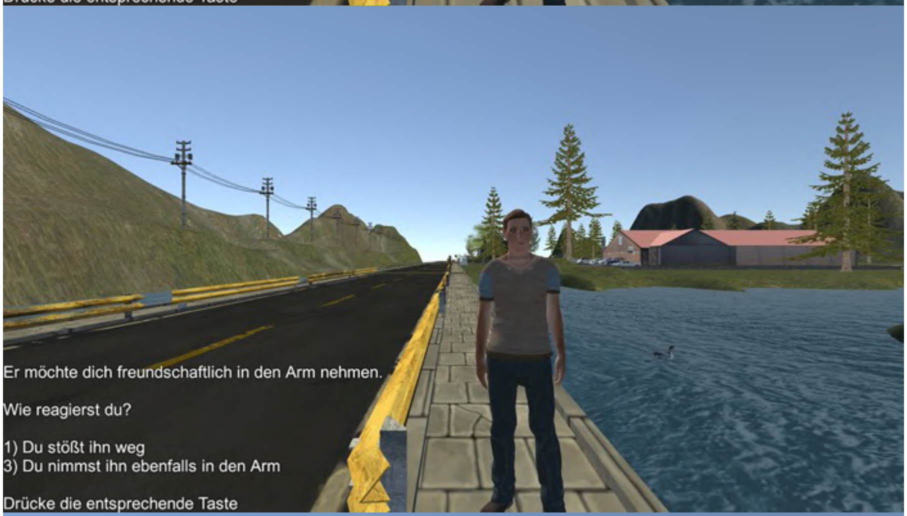
Er möchte dich freundschaftlich in den Arm nehmen.
Wie reagierst du?
1) Du stößt ihn weg
3) Du nimmst ihn ebenfalls in den Arm
Drücke die entsprechende Taste

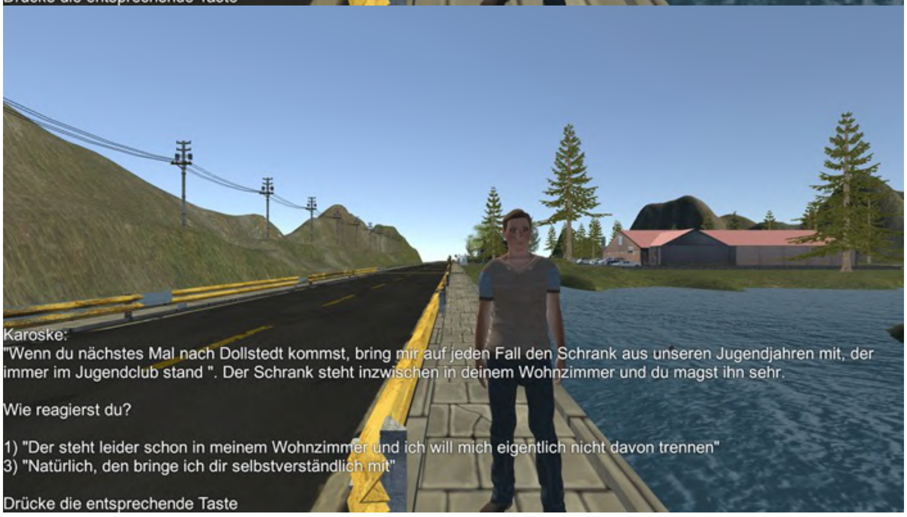
Karoske:
"Wenn du nächstes Mal nach Dollstedt kommst, bring mir auf jeden Fall den Schrank aus unseren Jugendjahren mit, der immer im Jugendclub stand ". Der Schrank steht inzwischen in deinem Wohnzimmer und du magst ihn sehr.
Wie reagierst du?
1) "Der steht leider schon in meinem Wohnzimmer und ich will mich eigentlich nicht davon trennen"
3) "Natürlich, den bringe ich dir selbstverständlich mit"
Drücke die entsprechende Taste

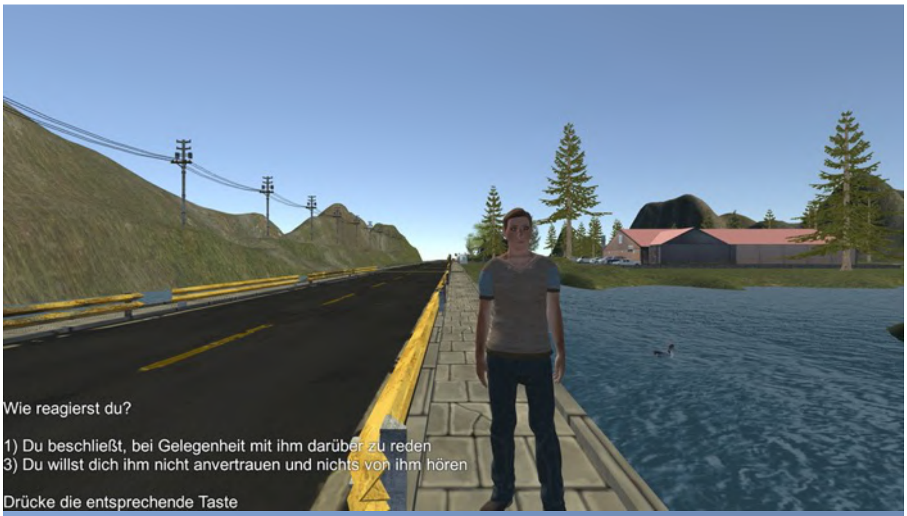
Wie reagierst du?
1) Du beschließt, bei Gelegenheit mit ihm darüber zu reden
3) Du willst dich ihm nicht anvertrauen und nichts von ihm hören
Drücke die entsprechende Taste

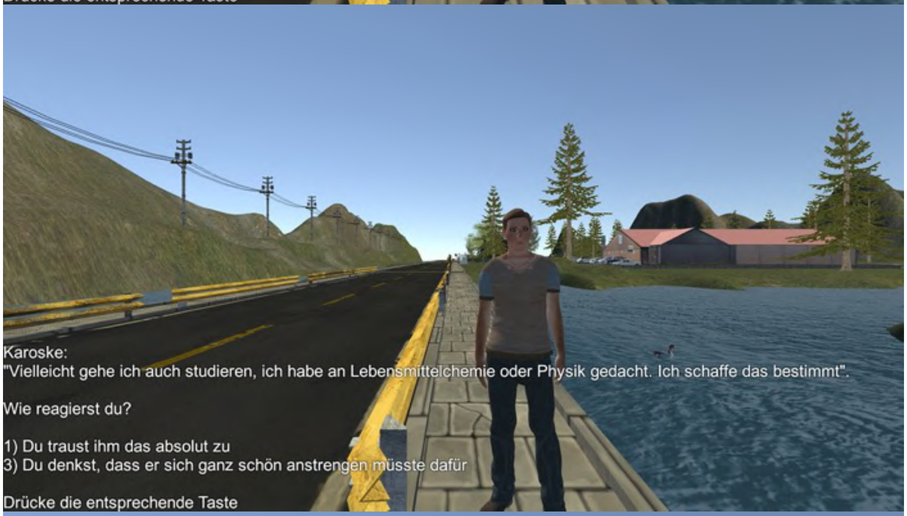
Karoske:
"Vielleicht gehe ich auch studieren, ich habe an Lebensmittelchemie oder Physik gedacht. Ich schaffe das bestimmt".
Wie reagierst du?
1) Du traust ihm das absolut zu
3) Du denkst, dass er sich ganz schön anstrengen müsste dafür
Drücke die entsprechende Taste

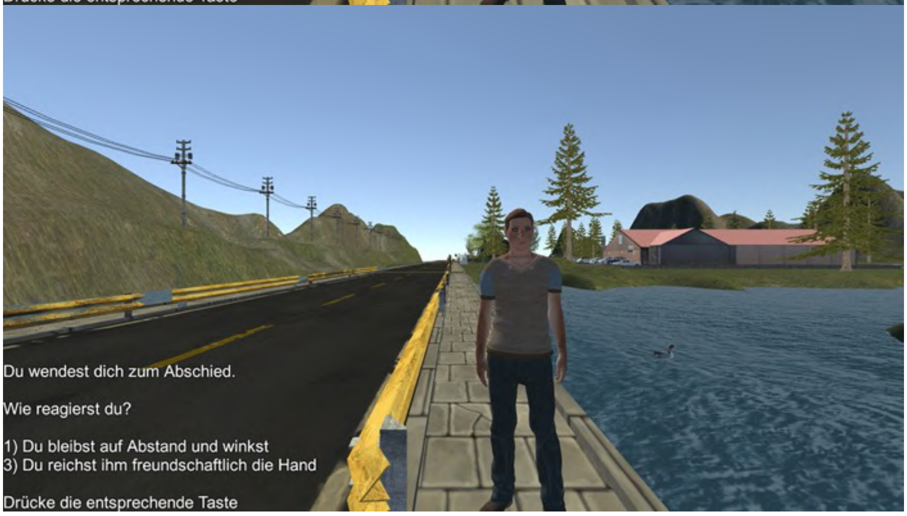
Du wendest dich zum Abschied.
Wie reagierst du?
1) Du bleibst auf Abstand und winkst
3) Du reichst ihm freundschaftlich die Hand
Drücke die entsprechende Taste

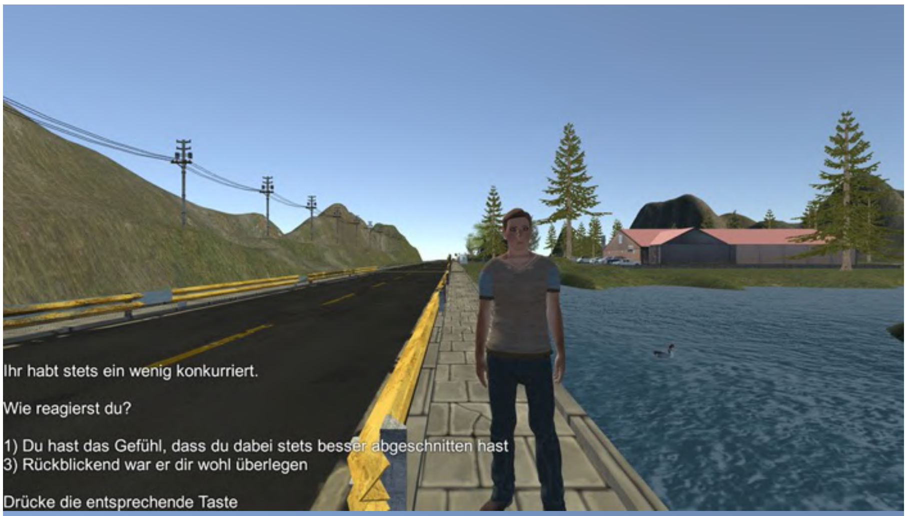
Ihr habt stets ein wenig konkurriert.
Wie reagierst du?
1) Du hast das Gefühl, dass du dabei stets besser abgeschnitten hast
3) Rückblickend war er dir wohl überlegen
Drücke die entsprechende Taste

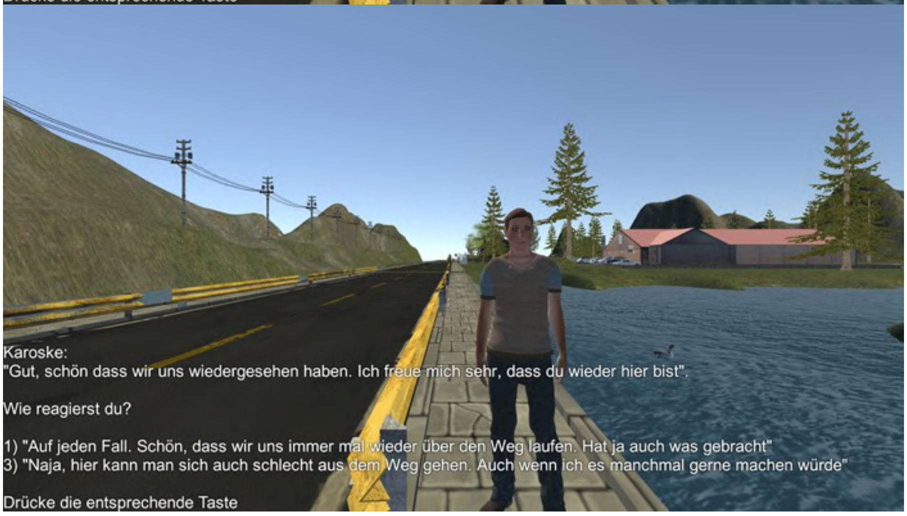
Karoske:
"Gut, schön dass wir uns wiedergesehen haben. Ich freue mich sehr, dass du wieder hier bist".
Wie reagierst du?
1) "Auf jeden Fall. Schön, dass wir uns immer mal wieder über den Weg laufen. Hat ja auch was gebracht"
3) "Naja, hier kann man sich auch schlecht aus dem Weg gehen. Auch wenn ich es manchmal gerne machen würde"
Drücke die entsprechende Taste

S5.2 Modification-Phase

S5.2.1 Lerte (High-Power/Low-Affiliation, Updating-Avatar)

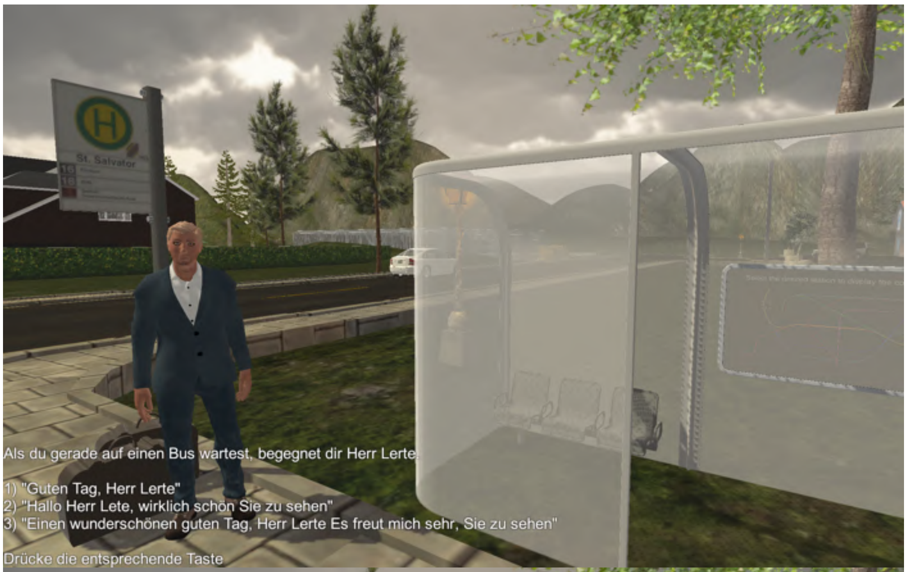

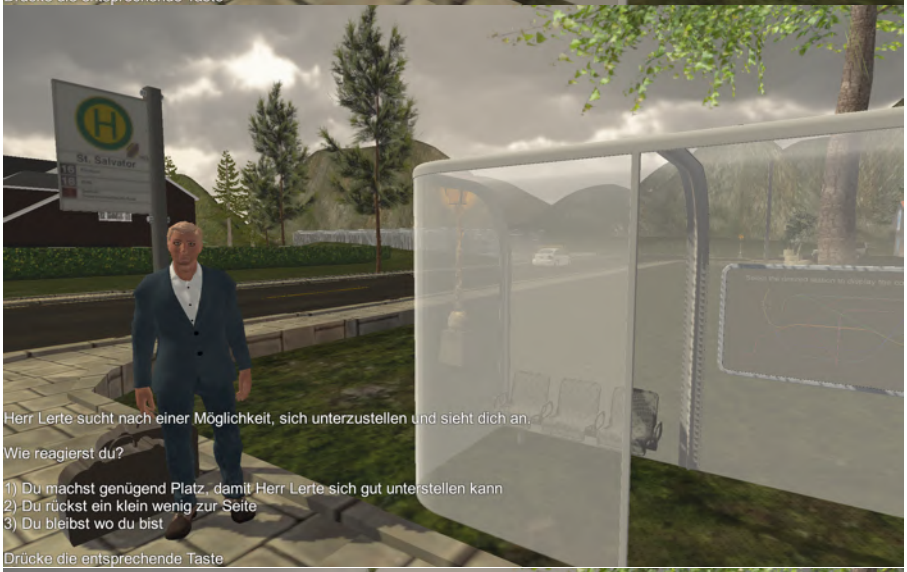

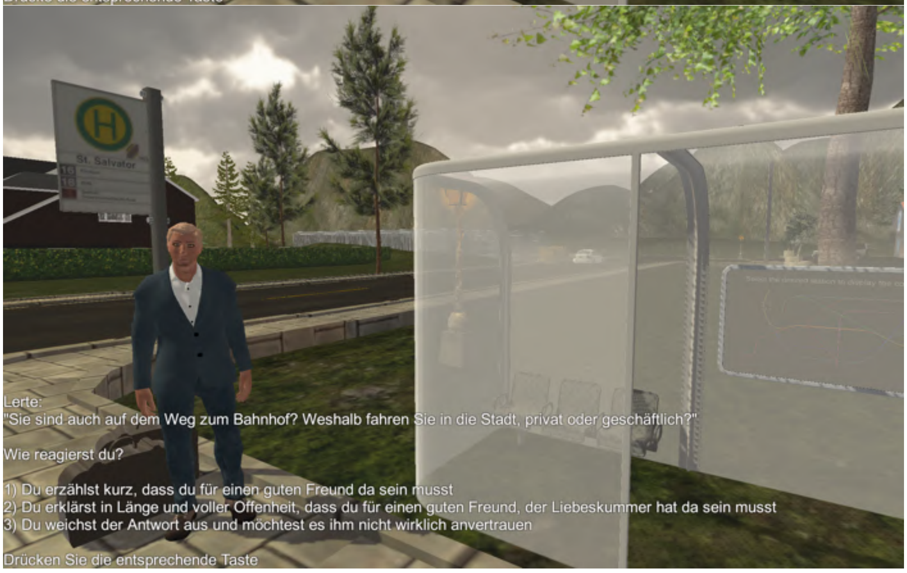

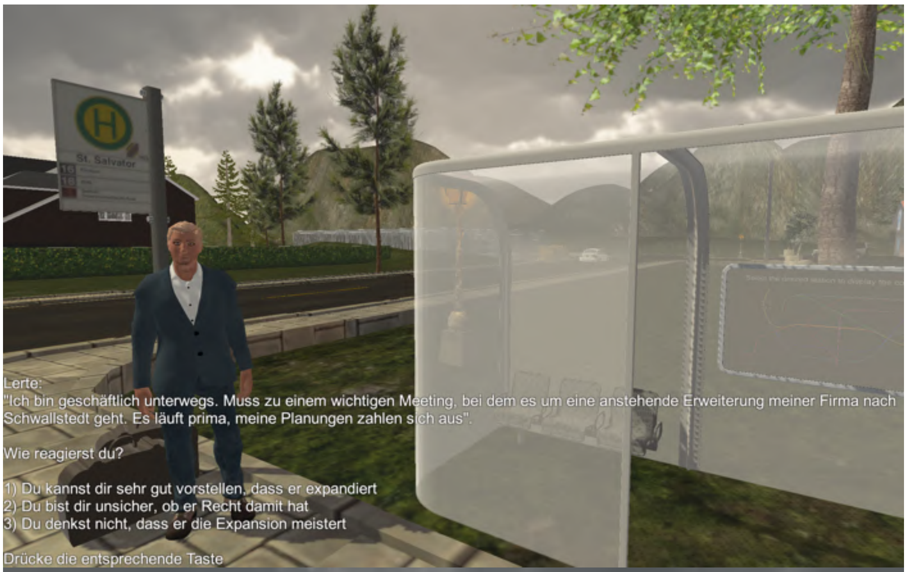
Lerte:
"Ich bin geschäftlich unterwegs. Muss zu einem wichtigen Meeting, bei dem es um eine anstehende Erweiterung meiner Firma nach Schwallstedt geht. Es läuft prima, meine Planungen zahlen sich aus".
Wie reagierst du?
1) Du kannst dir sehr gut vorstellen, dass er expandiert
2) Du bist dir unsicher, ob er Recht damit hat
3) Du denkst nicht, dass er die Expansion meistert
Drücke die entsprechende Taste

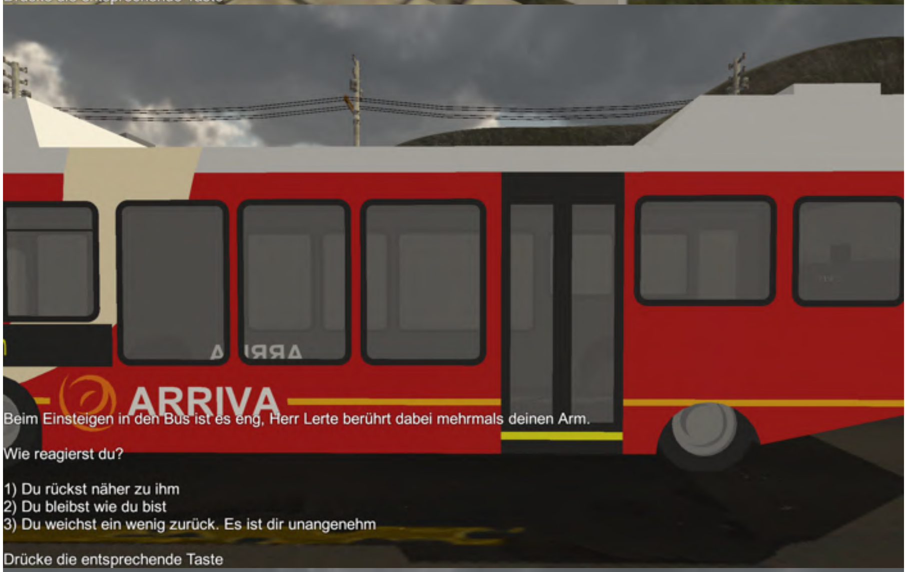
ARRIVA
Beim Einsteigen in den Bus ist es eng, Herr Lerte berührt dabei mehrmals deinen Arm.
Wie reagierst du?
1) Du rückst näher zu ihm
2) Du bleibst wie du bist
3) Du weichst ein wenig zurück. Es ist dir unangenehm
Drücke die entsprechende Taste

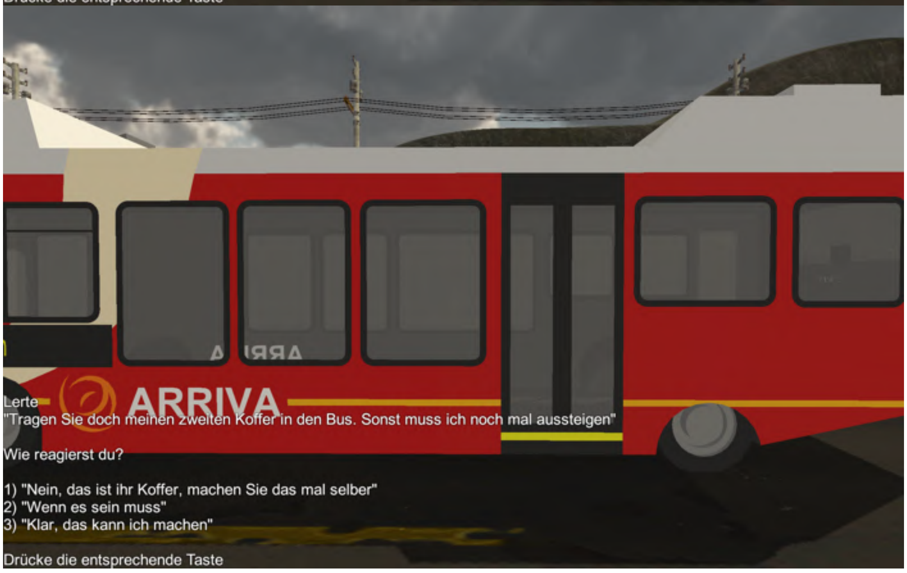
ARRIVA
Lerte
"Tragen Sie doch meinen zweiten Koffer in den Bus. Sonst muss ich noch mal aussteigen"
Wie reagierst du?
1) "Nein, das ist ihr Koffer, machen Sie das mal selber"
2) "Wenn es sein muss"
3) "Klar, das kann ich machen"
Drücke die entsprechende Taste

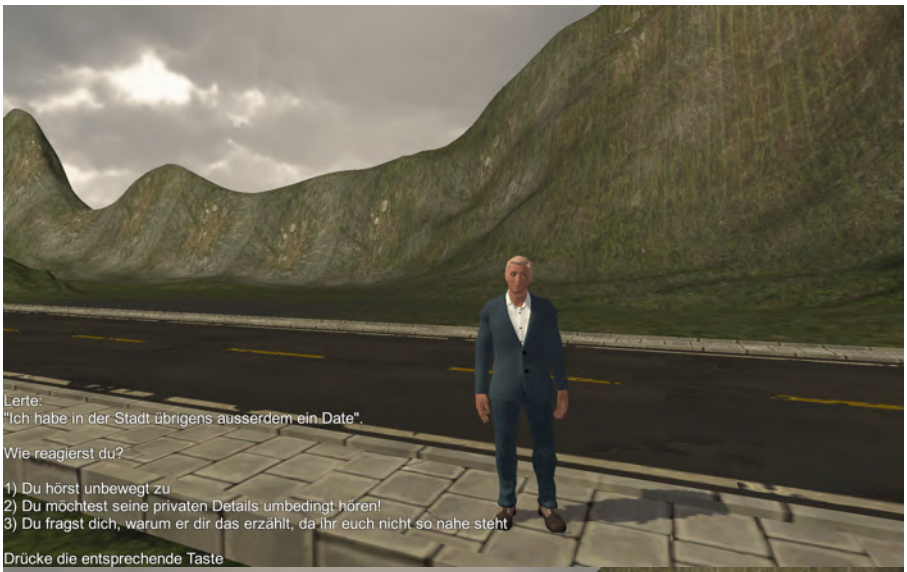
Lerte:
"Ich habe in der Stadt übrigens ausserdem ein Date".
Wie reagierst du?
1) Du hörst unbewegt zu
2) Du möchtest seine privaten Details umbedingt hören!
3) Du fragst dich, warum er dir das erzählt, da ihr euch nicht so nahe steht
Drücke die entsprechende Taste

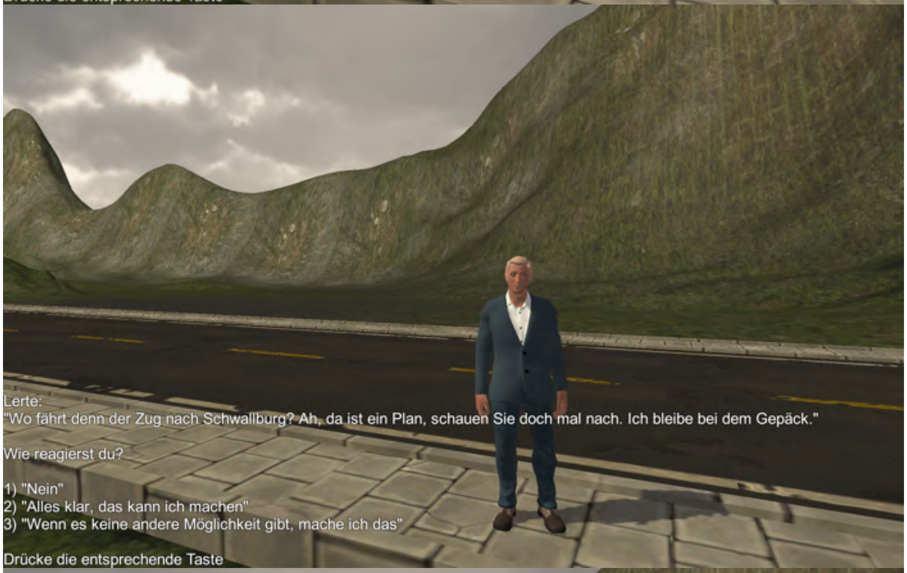
Lerte:
"Wo fährt denn der Zug nach Schwallburg? Ah, da ist ein Plan, schauen Sie doch mal nach. Ich bleibe bei dem Gepäck."
Wie reagierst du?
1) "Nein"
2) "Alles klar, das kann ich machen"
3) "Wenn es keine andere Möglichkeit gibt, mache ich das"
Drücke die entsprechende Taste

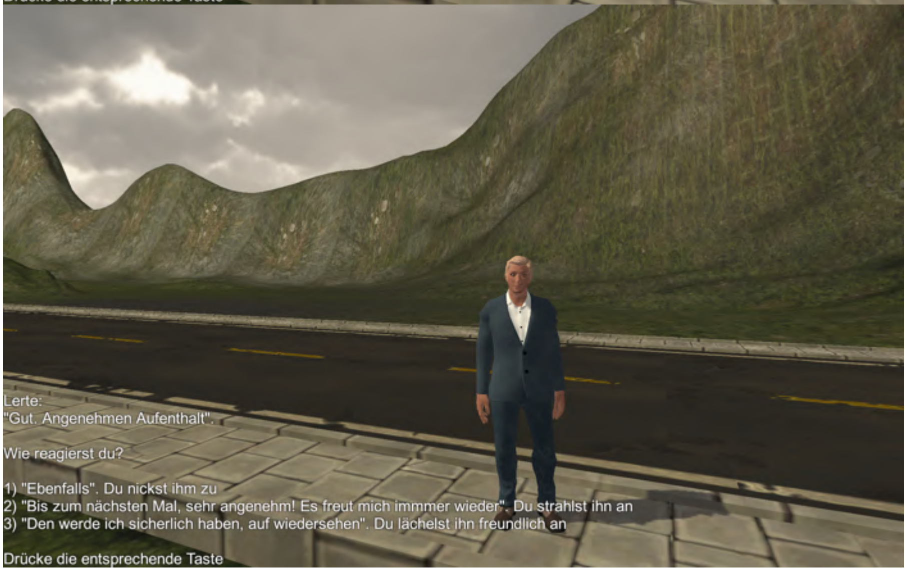
Lerte:
"Gut. Angenehmen Aufenthalt".
Wie reagierst du?
1) "Ebenfalls". Du nickst ihm zu
2) "Bis zum nächsten Mal, sehr angenehm! Es freut mich immmer wieder". Du strahlst ihn an
3) "Den werde ich sicherlich haben, auf wiedersehen". Du lächelst ihn freundlich an
Drücke die entsprechende Taste

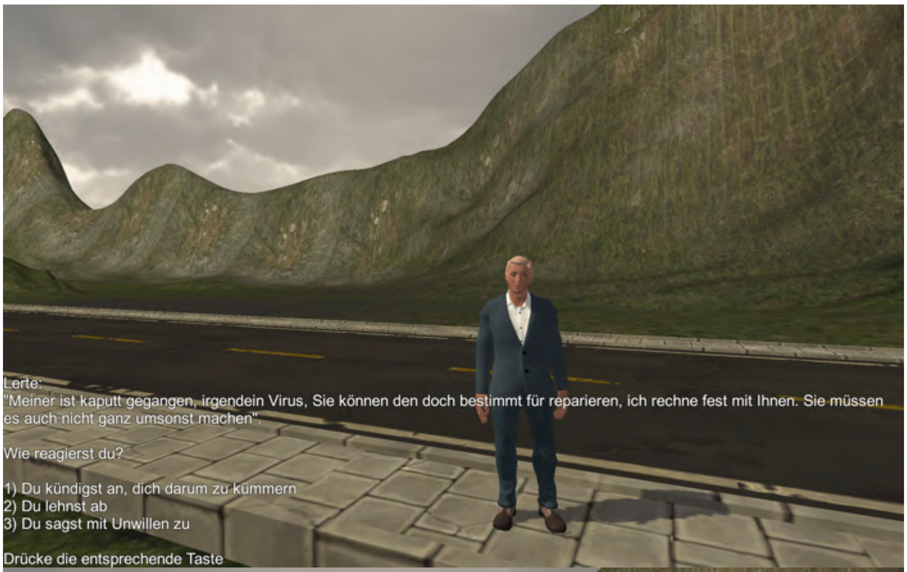
Lerte:
"Meiner ist kaputt gegangen, irgendein Virus, Sie können den doch bestimmt für reparieren, ich rechne fest mit Ihnen. Sie müssen es auch nicht ganz umsonst machen".
Wie reagierst du?
1) Du kündigst an, dich darum zu kümmern
2) Du lehnst ab
3) Du sagst mit Unwillen zu
Drücke die entsprechende Taste

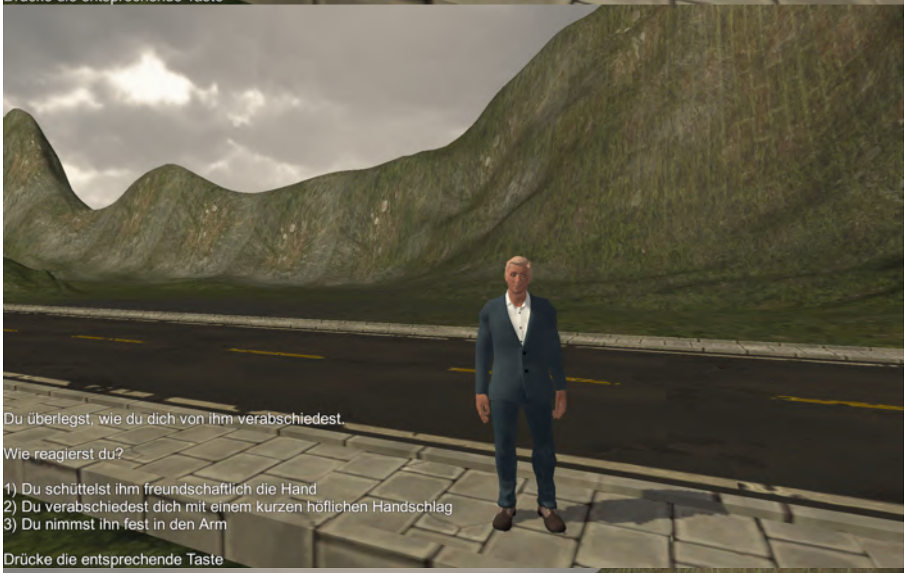
Du überlegst, wie du dich von ihm verabschiedest.
Wie reagierst du?
1) Du schüttelst ihm freundschaftlich die Hand
2) Du verabschiedest dich mit einem kurzen höflichen Handschlag
3) Du nimmst ihn fest in den Arm
Drücke die entsprechende Taste

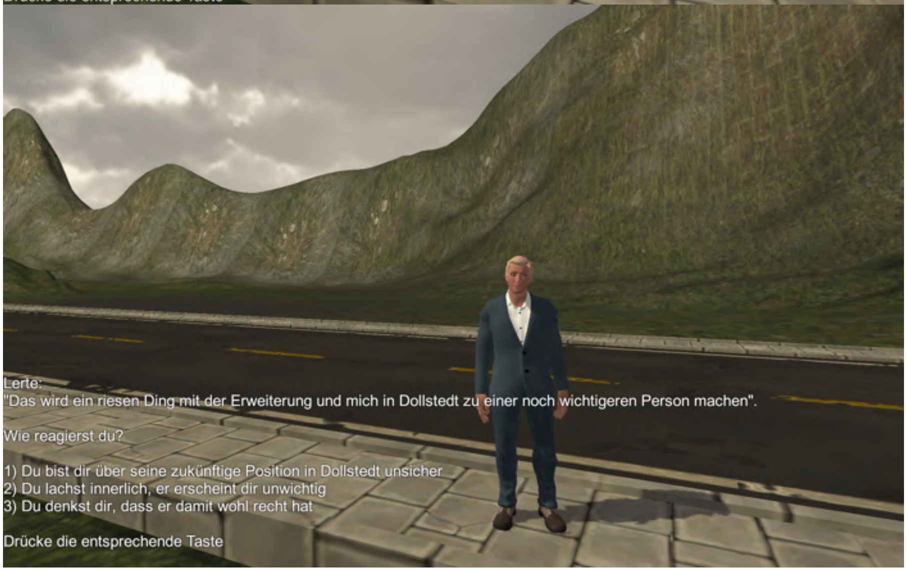
Lerte:
"Das wird ein riesen Ding mit der Erweiterung und mich in Dollstedt zu einer noch wichtigeren Person machen".
Wie reagierst du?
1) Du bist dir über seine zukünftige Position in Dollstedt unsicher
2) Du lachst innerlich, er erscheint dir unwichtig
3) Du denkst dir, dass er damit wohl recht hat
Drücke die entsprechende Taste

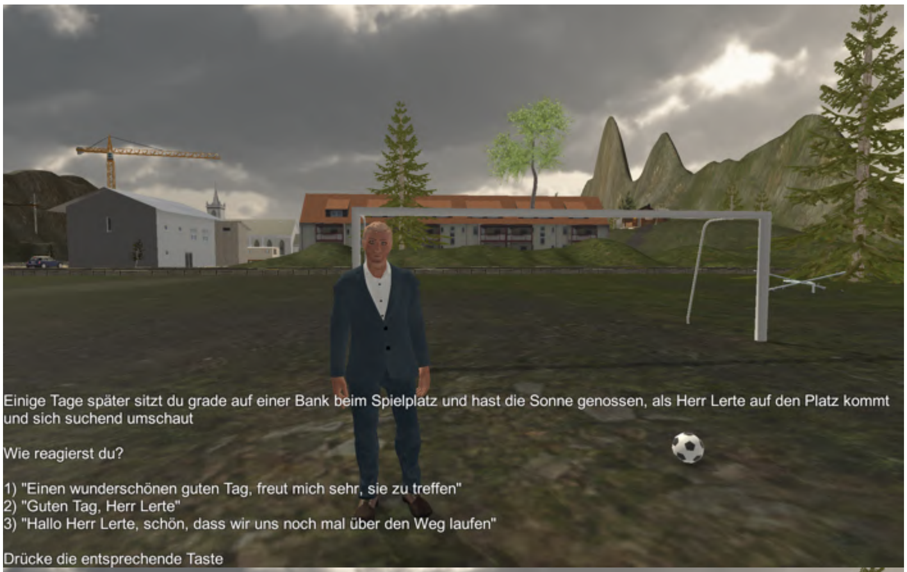
Einige Tage später sitzt du grade auf einer Bank beim Spielplatz und hast die Sonne genossen, als Herr Lerte auf den Platz kommt und sich suchend umschaut
Wie reagierst du?
1) "Einen wunderschönen guten Tag, freut mich sehr, sie zu treffen"
2) "Guten Tag, Herr Lerte"
3) "Hallo Herr Lerte, schön, dass wir uns noch mal über den Weg laufen"
Drücke die entsprechende Taste
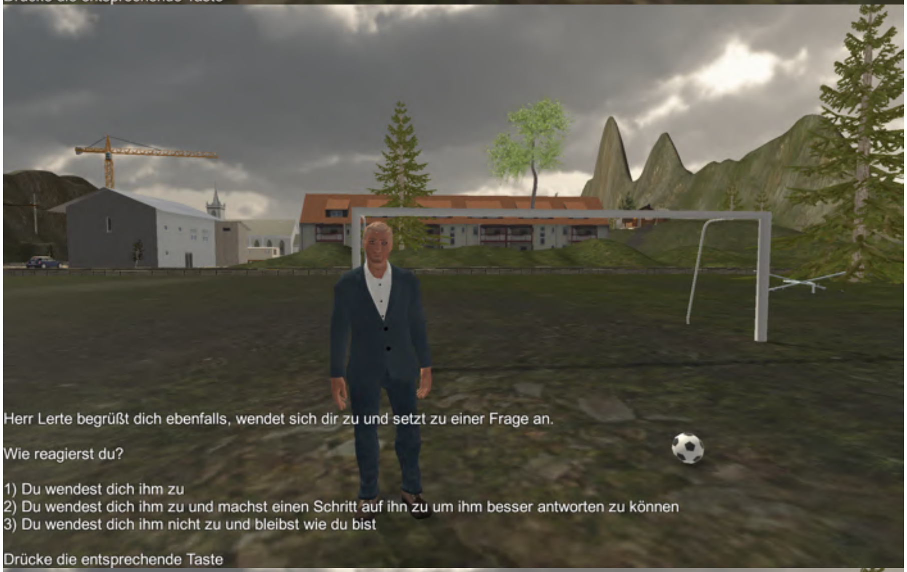
Herr Lerte begrüßt dich ebenfalls, wendet sich dir zu und setzt zu einer Frage an.
Wie reagierst du?
1) Du wendest dich ihm zu
2) Du wendest dich ihm zu und machst einen Schritt auf ihn zu um ihm besser antworten zu können
3) Du wendest dich ihm nicht zu und bleibst wie du bist
Drücke die entsprechende Taste
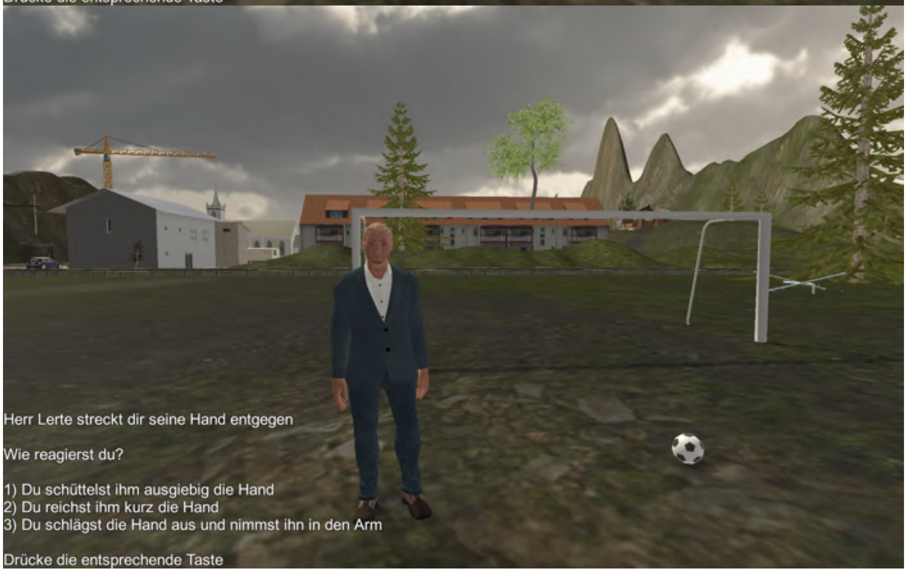
Herr Lerte streckt dir seine Hand entgegen
Wie reagierst du?
1) Du schüttelst ihm ausgiebig die Hand
2) Du reichst ihm kurz die Hand
3) Du schlägst die Hand aus und nimmst ihn in den Arm
Drücke die entsprechende Taste

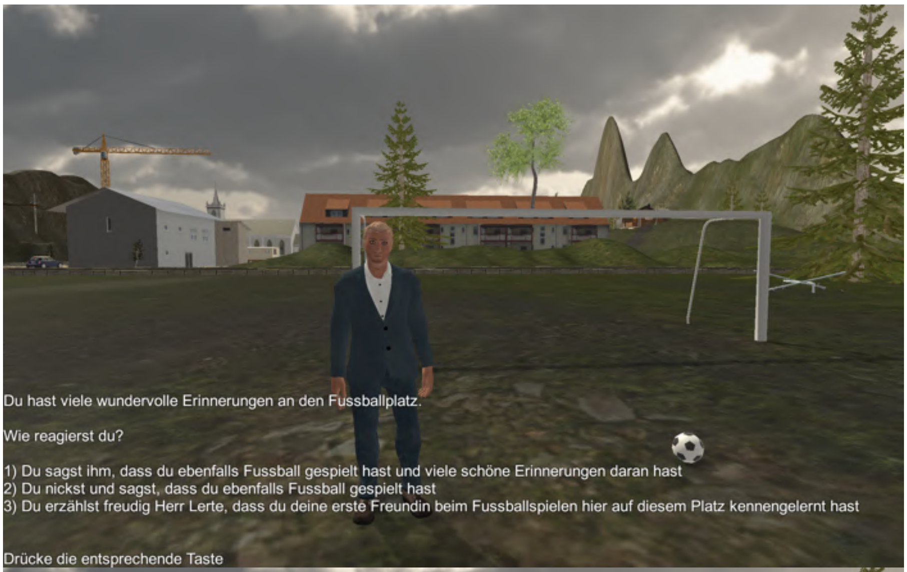
Du hast viele wundervolle Erinnerungen an den Fussballplatz.
Wie reagierst du?
1) Du sagst ihm, dass du ebenfalls Fussball gespielt hast und viele schöne Erinnerungen daran hast
2) Du nickst und sagst, dass du ebenfalls Fussball gespielt hast
3) Du erzählst freudig Herr Lerte, dass du deine erste Freundin beim Fussballspielen hier auf diesem Platz kennengelernt hast
Drücke die entsprechende Taste

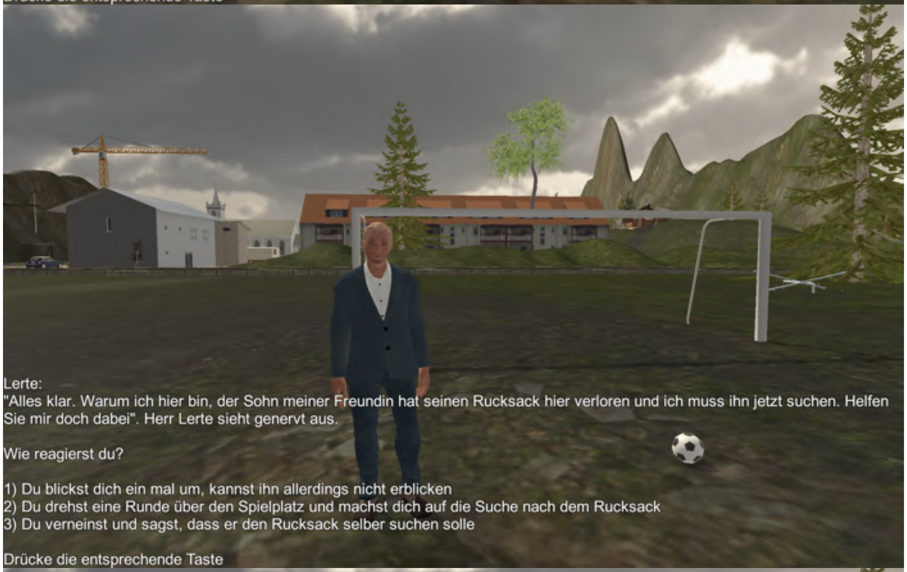
Lerte:
"Alles klar. Warum ich hier bin, der Sohn meiner Freundin hat seinen Rucksack hier verloren und ich muss ihn jetzt suchen. Helfen Sie mir doch dabei". Herr Lerte sieht genervt aus.
Wie reagierst du?
1) Du blickst dich ein mal um, kannst ihn allerdings nicht erblicken
2) Du drehst eine Runde über den Spielplatz und machst dich auf die Suche nach dem Rucksack
3) Du verneinst und sagst, dass er den Rucksack selber suchen solle
Drücke die entsprechende Taste

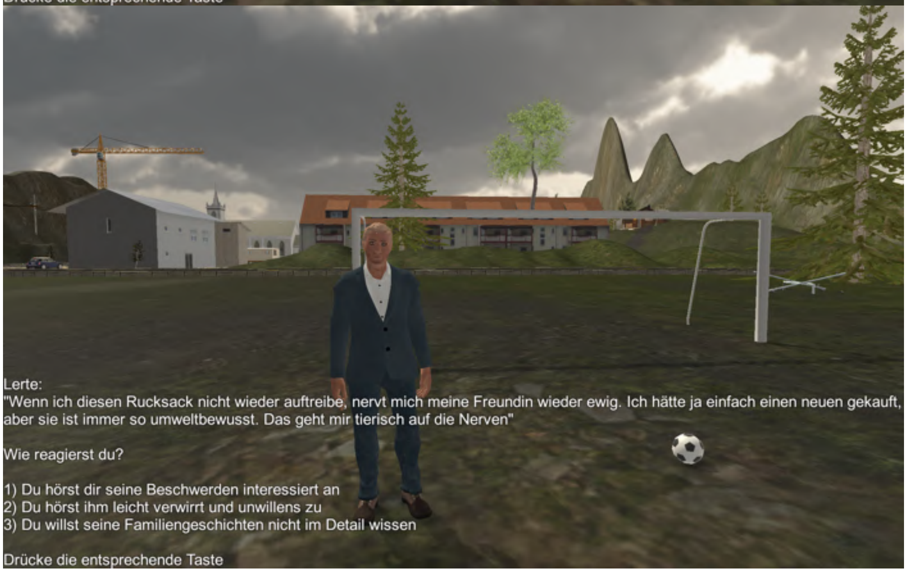
Lerte:
"Wenn ich diesen Rucksack nicht wieder auftreibe, nervt mich meine Freundin wieder ewig. Ich hätte ja einfach einen neuen gekauft, aber sie ist immer so umweltbewusst. Das geht mir tierisch auf die Nerven"
Wie reagierst du?
1) Du hörst dir seine Beschwerden interessiert an
2) Du hörst ihm leicht verwirrt und unwillens zu
3) Du willst seine Familiengeschichten nicht im Detail wissen
Drücke die entsprechende Taste

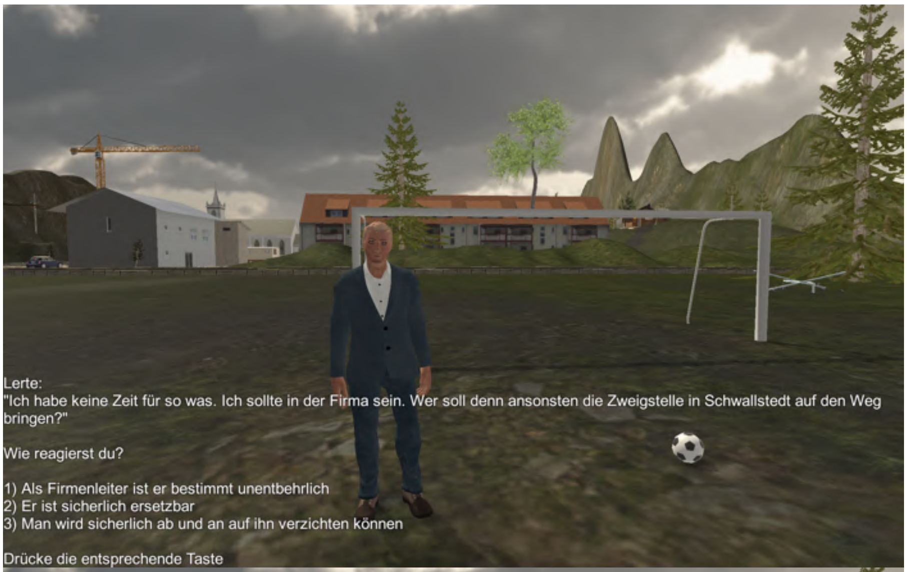
Lerte:
"Ich habe keine Zeit für so was. Ich sollte in der Firma sein. Wer soll denn ansonsten die Zweigstelle in Schwallstedt auf den Weg bringen?"
Wie reagierst du?
1) Als Firmenleiter ist er bestimmt unentbehrlich
2) Er ist sicherlich ersetzbar
3) Man wird sicherlich ab und an auf ihn verzichten können
Drücke die entsprechende Taste

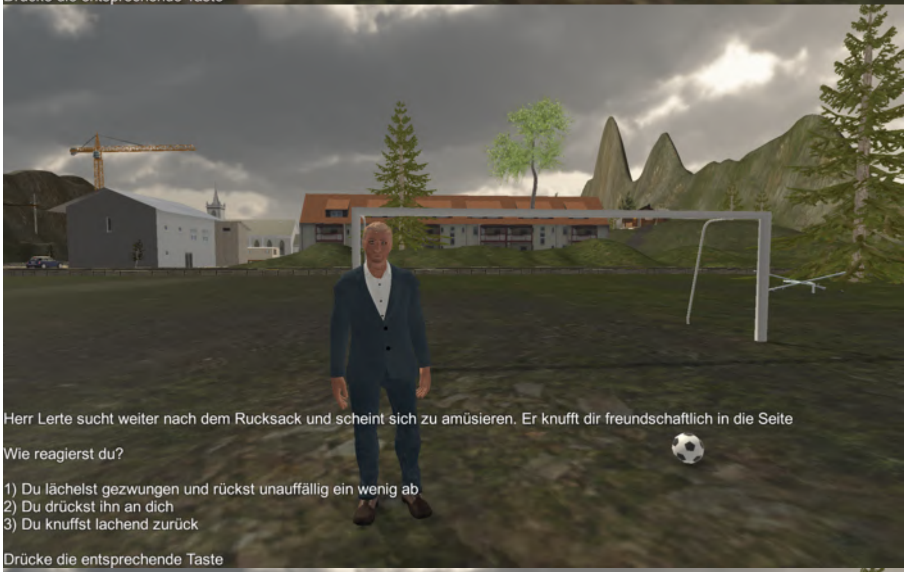
Herr Lerte sucht weiter nach dem Rucksack und scheint sich zu amüsieren. Er knufft dir freundschaftlich in die Seite
Wie reagierst du?
1) Du lächelst gezwungen und rückst unauffällig ein wenig ab
2) Du drückst ihn an dich
3) Du knuffst lachend zurück
Drücke die entsprechende Taste

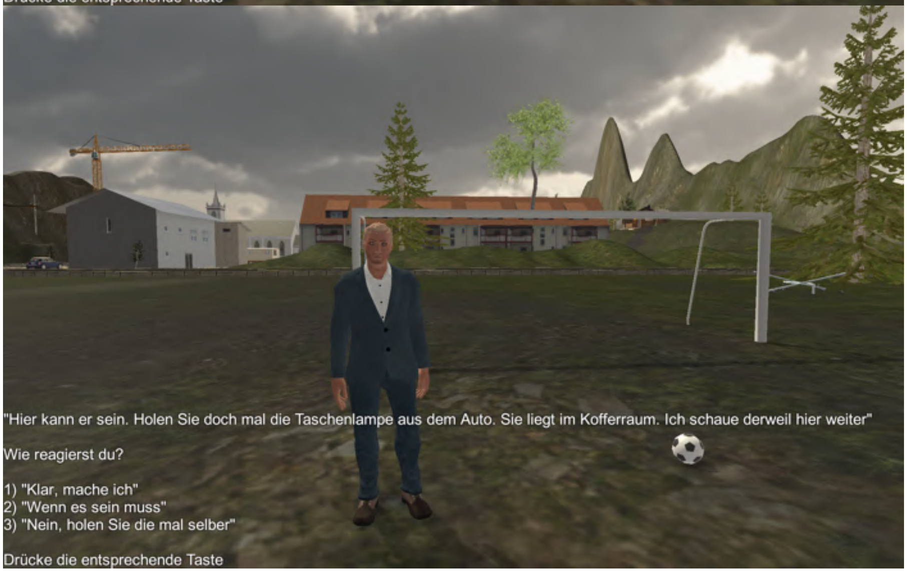
"Hier kann er sein. Holen Sie doch mal die Taschenlampe aus dem Auto. Sie liegt im Kofferraum. Ich schaue derweil hier weiter"
Wie reagierst du?
1) "Klar, mache ich"
2) "Wenn es sein muss"
3) "Nein, holen Sie die mal selber"
Drücke die entsprechende Taste

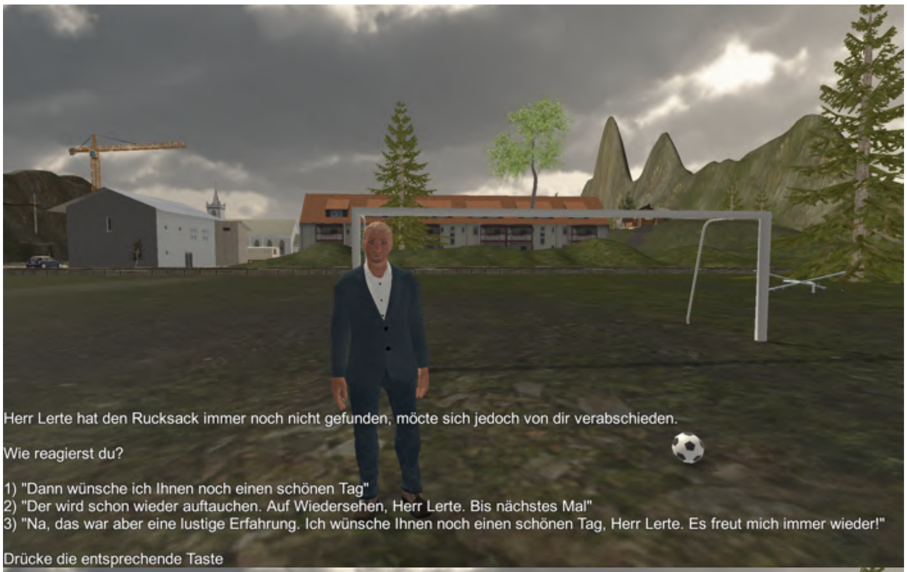
Herr Lerte hat den Rucksack immer noch nicht gefunden, möcte sich jedoch von dir verabschieden.
Wie reagierst du?
1) "Dann wünsche ich Ihnen noch einen schönen Tag"
2) "Der wird schon wieder auftauchen. Auf Wiedersehen, Herr Lerte. Bis nächstes Mal"
3) "Na, das war aber eine lustige Erfahrung. Ich wünsche Ihnen noch einen schönen Tag, Herr Lerte. Es freut mich immer wieder!"
Drücke die entsprechende Taste

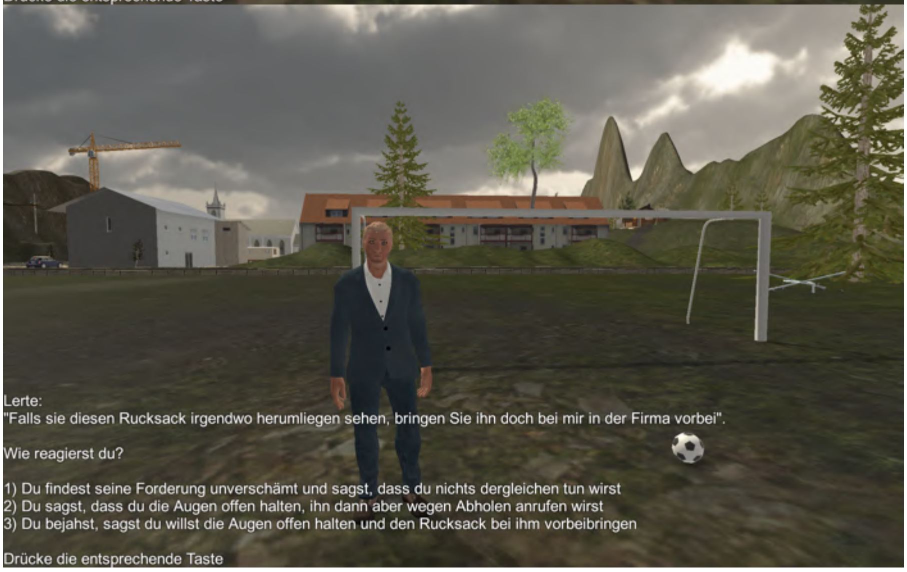
Lerte:
"Falls sie diesen Rucksack irgendwo herumliegen sehen, bringen Sie ihn doch bei mir in der Firma vorbei".
Wie reagierst du?
1) Du findest seine Forderung unverschämt und sagst, dass du nichts dergleichen tun wirst
2) Du sagst, dass du die Augen offen halten, ihn dann aber wegen Abholen anrufen wirst
3) Du bejahst, sagst du willst die Augen offen halten und den Rucksack bei ihm vorbeibringen
Drücke die entsprechende Taste

S5.2.2 Hoekenburg (High-Power/Low-Affiliation, Remapping-Avatar)

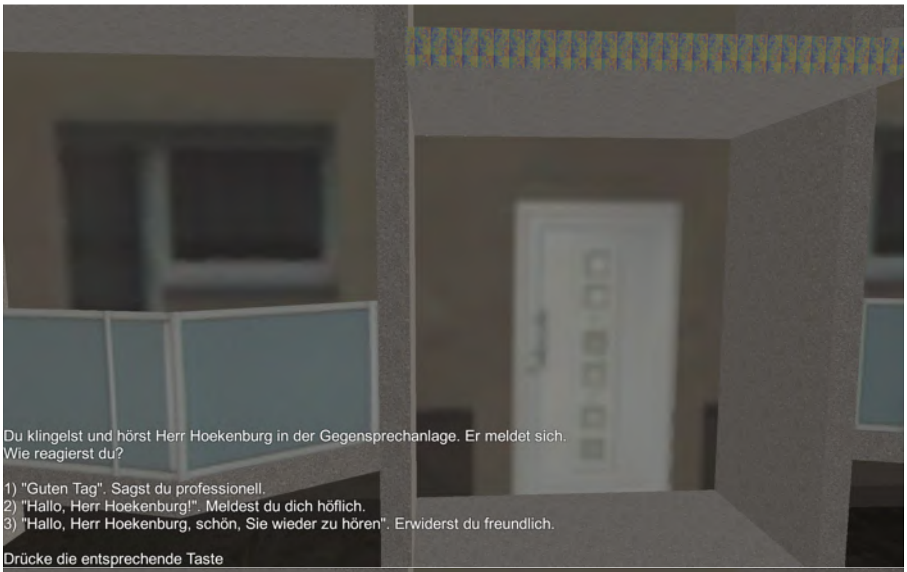

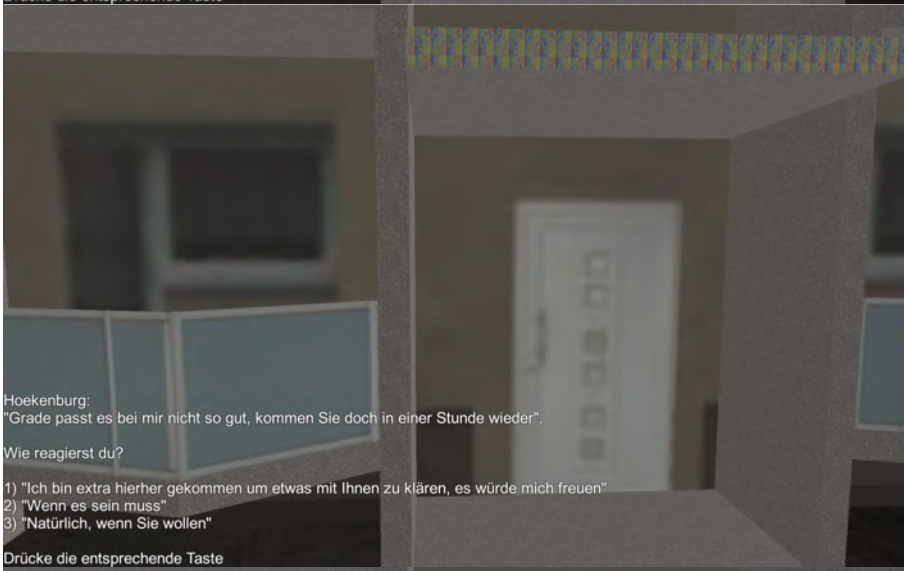

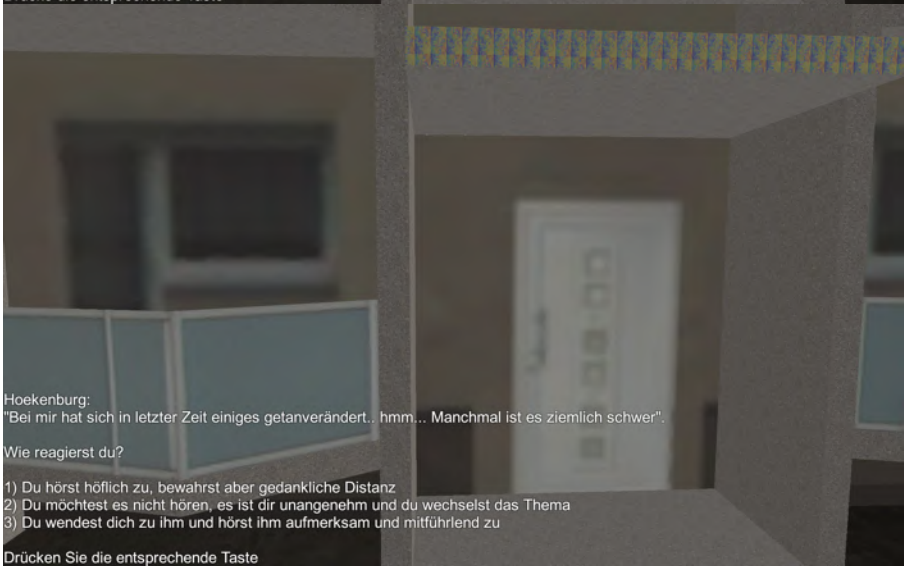

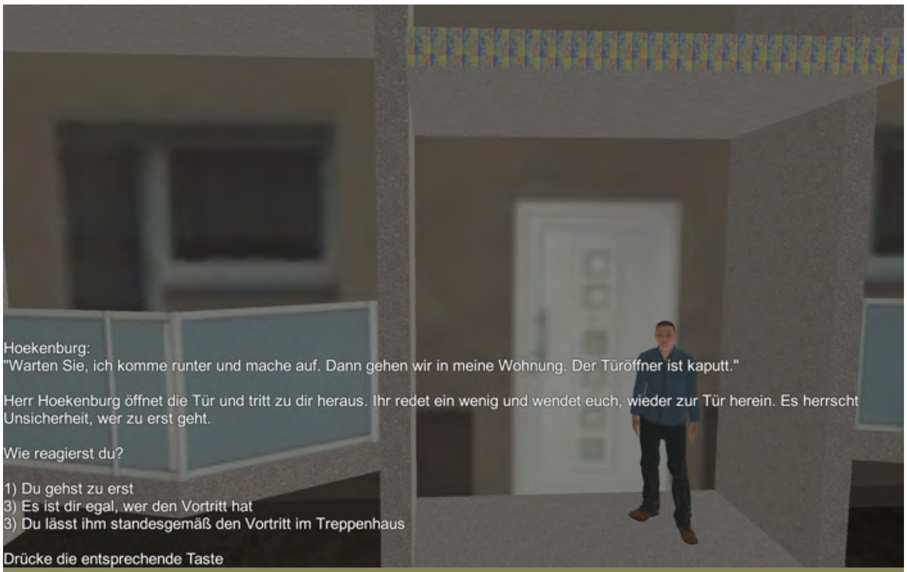
Hoekenburg:
"Warten Sie, ich komme runter und mache auf. Dann gehen wir in meine Wohnung. Der Türöffner ist kaputt."
Herr Hoekenburg öffnet die Tür und tritt zu dir heraus. Ihr redet ein wenig und wendet euch, wieder zur Tür herein. Es herrscht Unsicherheit, wer zu erst geht.
Wie reagierst du?
1) Du gehst zu erst
3) Es ist dir egal, wer den Vortritt hat
3) Du lässt ihm standesgemäß den Vortritt im Treppenhaus
Drücke die entsprechende Taste

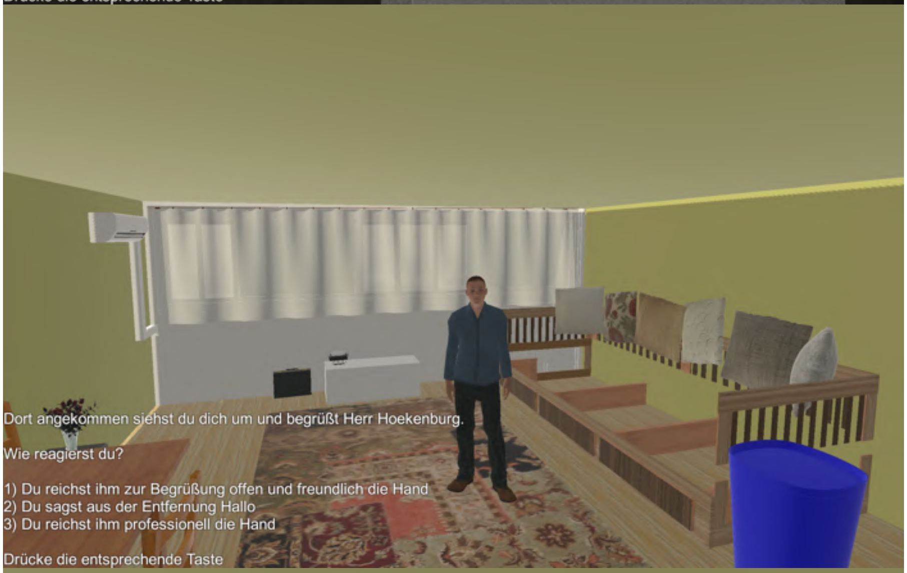
Dort angekommen siehst du dich um und begrüßt Herr Hoekenburg.
Wie reagierst du?
1) Du reichst ihm zur Begrüßung offen und freundlich die Hand
2) Du sagst aus der Entfernung Hallo
3) Du reichst ihm professionell die Hand
Drücke die entsprechende Taste

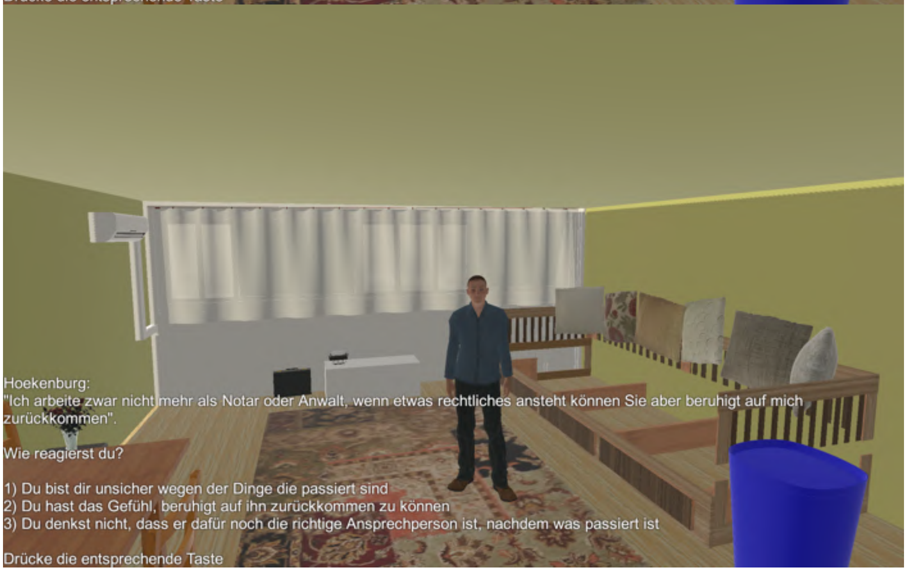
Hoekenburg:
"Ich arbeite zwar nicht mehr als Notar oder Anwalt, wenn etwas rechtliches ansteht können Sie aber beruhigt auf mich zurückkommen".
Wie reagierst du?
1) Du bist dir unsicher wegen der Dinge die passiert sind
2) Du hast das Gefühl, beruhigt auf ihn zurückkommen zu können
3) Du denkst nicht, dass er dafür noch die richtige Ansprechperson ist, nachdem was passiert ist
Drücke die entsprechende Taste

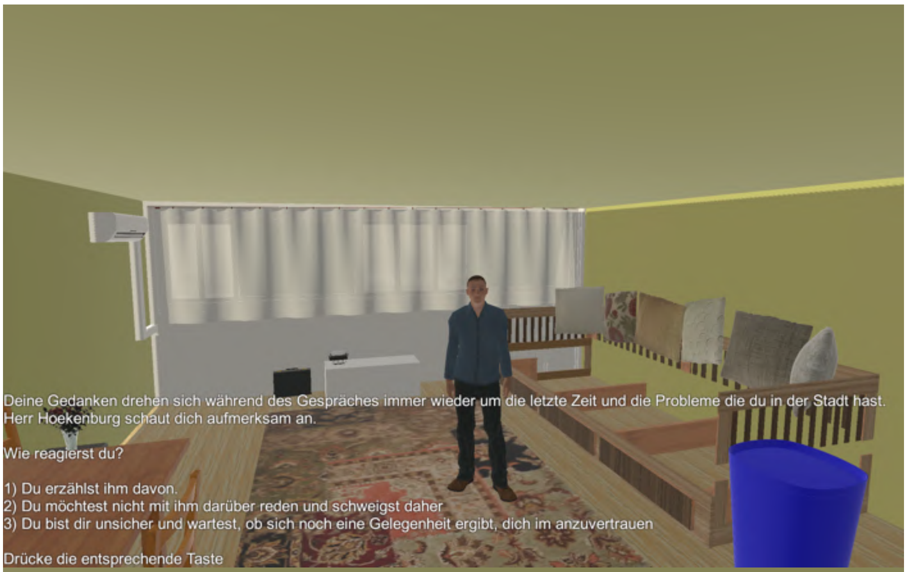
Deine Gedanken drehen sich während des Gespräches immer wieder um die letzte Zeit und die Probleme die du in der Stadt hast.
Herr Hoekenburg schaut dich aufmerksam an.
Wie reagierst du?
1) Du erzählst ihm davon.
2) Du möchtest nicht mit ihm darüber reden und schweigst daher
3) Du bist dir unsicher und wartest, ob sich noch eine Gelegenheit ergibt, dich im anzuvertrauen
Drücke die entsprechende Taste

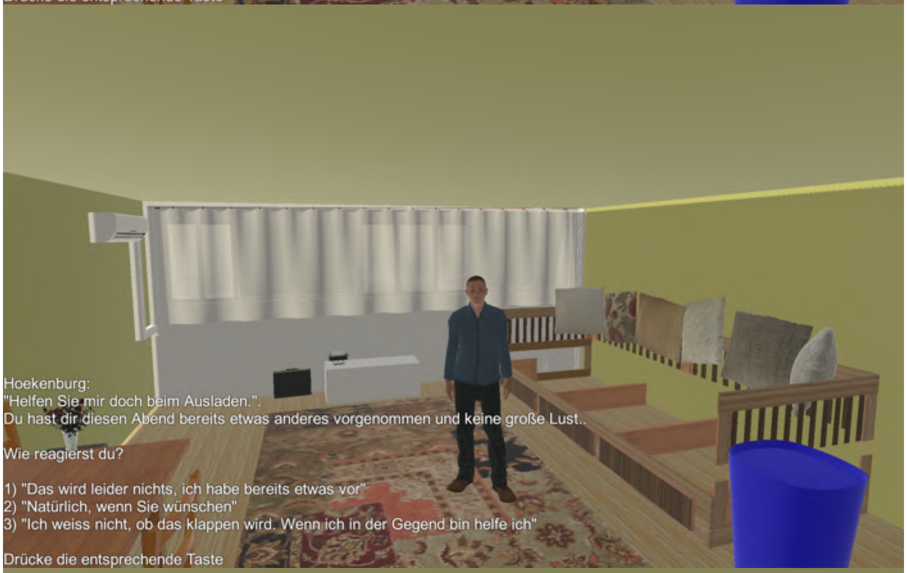
Hoekenburg:
"Helfen Sie mir doch beim Ausladen.".
Du hast dir diesen Abend bereits etwas anderes vorgenommen und keine große Lust..
Wie reagierst du?
1) "Das wird leider nichts, ich habe bereits etwas vor"
2) "Natürlich, wenn Sie wünschen"
3) "Ich weiss nicht, ob das klappen wird. Wenn ich in der Gegend bin helfe ich"
Drücke die entsprechende Taste

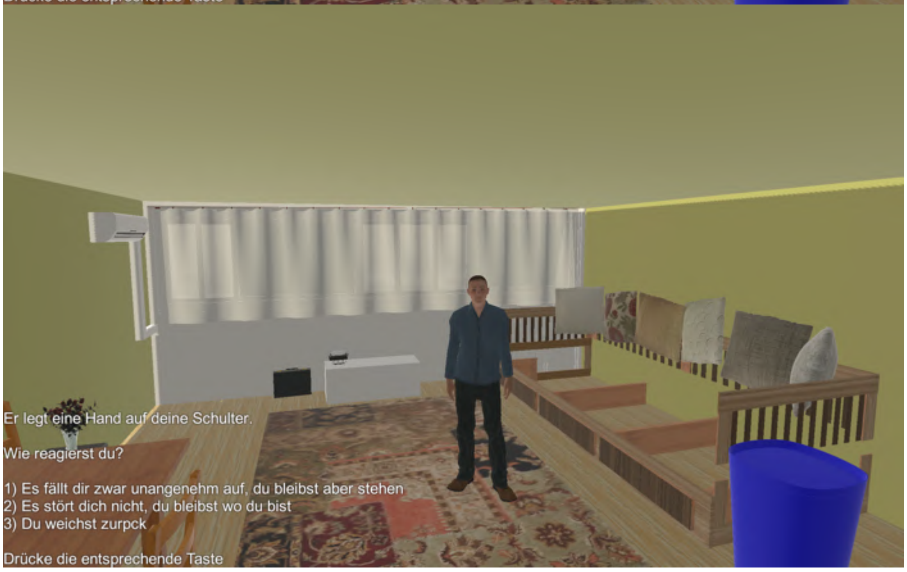
Er legt eine Hand auf deine Schulter.
Wie reagierst du?
1) Es fällt dir zwar unangenehm auf, du bleibst aber stehen
2) Es stört dich nicht, du bleibst wo du bist
3) Du weichst zurpck
Drücke die entsprechende Taste

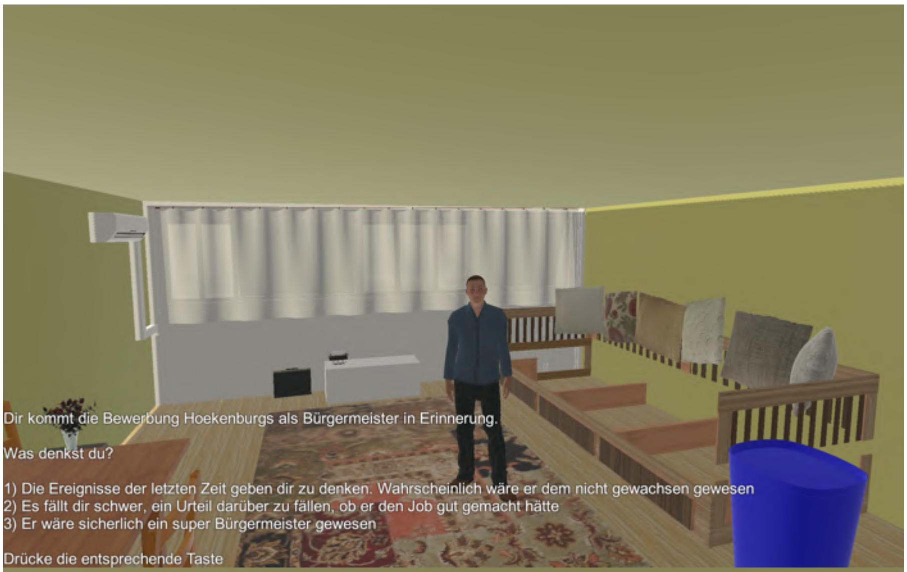
Dir kommt die Bewerbung Hoekenburgs als Bürgermeister in Erinnerung.
Was denkst du?
1) Die Ereignisse der letzten Zeit geben dir zu denken. Wahrscheinlich wäre er dem nicht gewachsen gewesen
2) Es fällt dir schwer, ein Urteil darüber zu fällen, ob er den Job gut gemacht hätte
3) Er wäre sicherlich ein super Bürgermeister gewesen
Drücke die entsprechende Taste

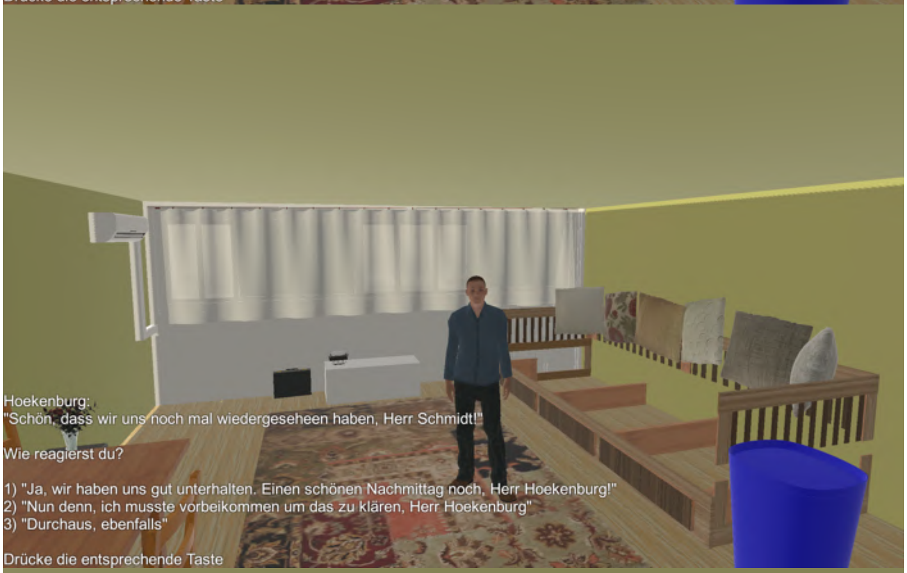
Hoekenburg:
"Schön, dass wir uns noch mal wiedergeseheen haben, Herr Schmidt!"
Wie reagierst du?
1) "Ja, wir haben uns gut unterhalten. Einen schönen Nachmittag noch, Herr Hoekenburg!"
2) "Nun denn, ich musste vorbeikommen um das zu klären, Herr Hoekenburg"
3) "Durchaus, ebenfalls"
Drücke die entsprechende Taste

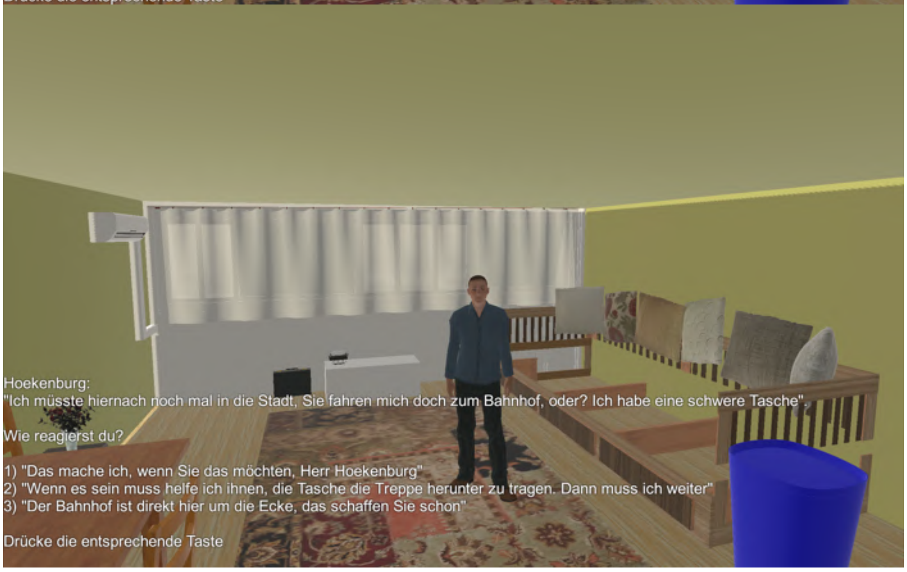
Hoekenburg:
"Ich müsste hiernach noch mal in die Stadt, Sie fahren mich doch zum Bahnhof, oder? Ich habe eine schwere Tasche".
Wie reagierst du?
1) "Das mache ich, wenn Sie das möchten, Herr Hoekenburg"
2) "Wenn es sein muss helfe ich ihnen, die Tasche die Treppe herunter zu tragen. Dann muss ich weiter"
3) "Der Bahnhof ist direkt hier um die Ecke, das schaffen Sie schon"
Drücke die entsprechende Taste

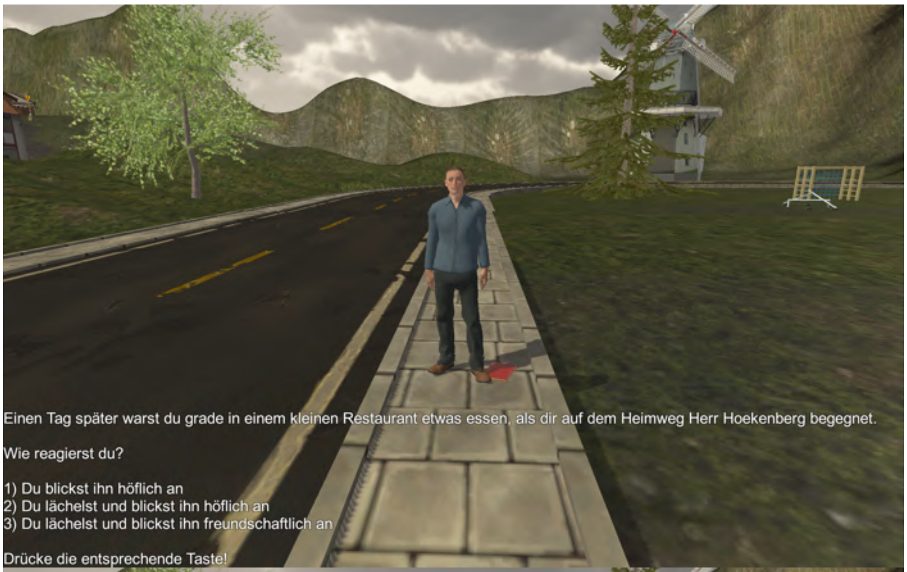
Einen Tag später warst du grade in einem kleinen Restaurant etwas essen, als dir auf dem Heimweg Herr Hoekenberg begegnet.
Wie reagierst du?
1) Du blickst ihn höflich an
2) Du lächelst und blickst ihn höflich an
3) Du lächelst und blickst ihn freundschaftlich an
Drücke die entsprechende Taste!

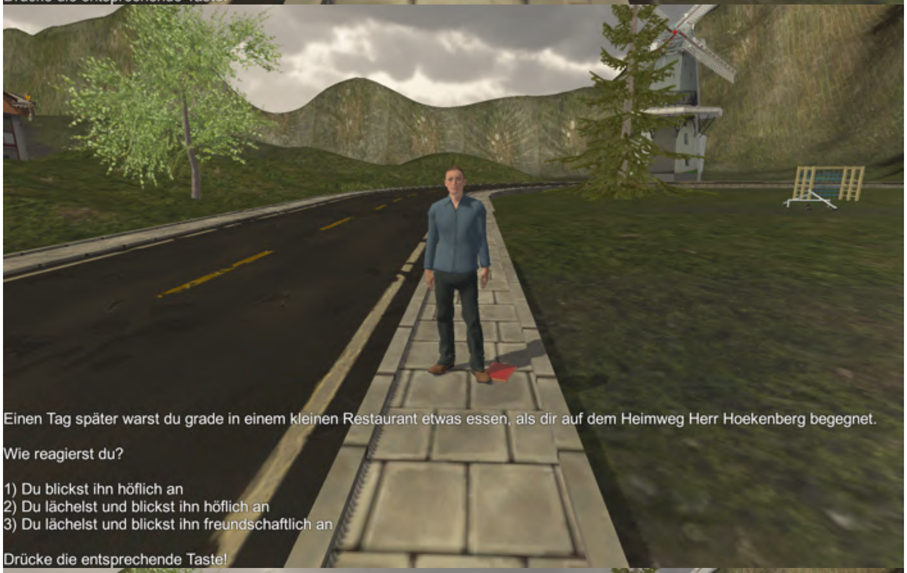
Einen Tag später warst du grade in einem kleinen Restaurant etwas essen, als dir auf dem Heimweg Herr Hoekenberg begegnet.
Wie reagierst du?
1) Du blickst ihn höflich an
2) Du lächelst und blickst ihn höflich an
3) Du lächelst und blickst ihn freundschaftlich an
Drücke die entsprechende Taste!

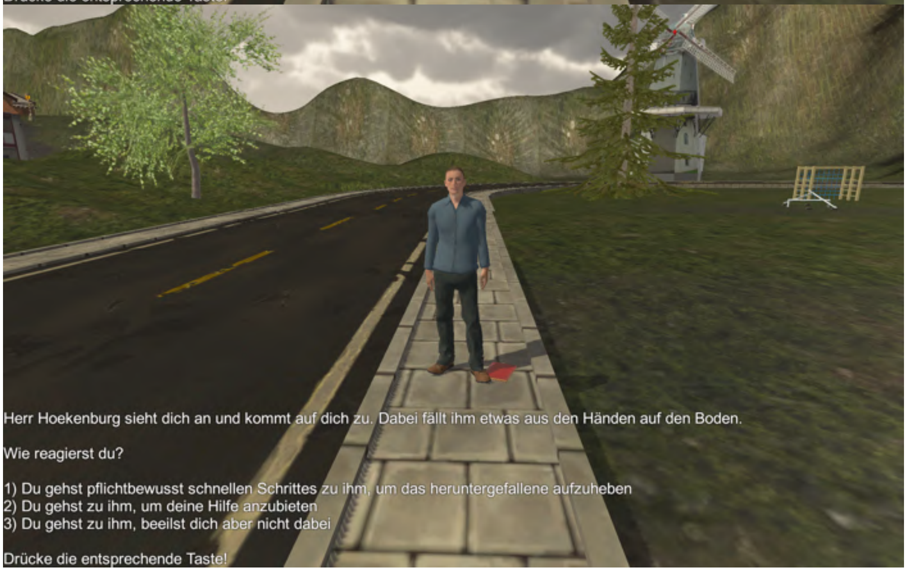
Herr Hoekenburg sieht dich an und kommt auf dich zu. Dabei fällt ihm etwas aus den Händen auf den Boden.
Wie reagierst du?
1) Du gehst pflichtbewusst schnellen Schrittes zu ihm, um das heruntergefallene aufzuheben
2) Du gehst zu ihm, um deine Hilfe anzubieten
3) Du gehst zu ihm, beeilst dich aber nicht dabei
Drücke die entsprechende Taste!

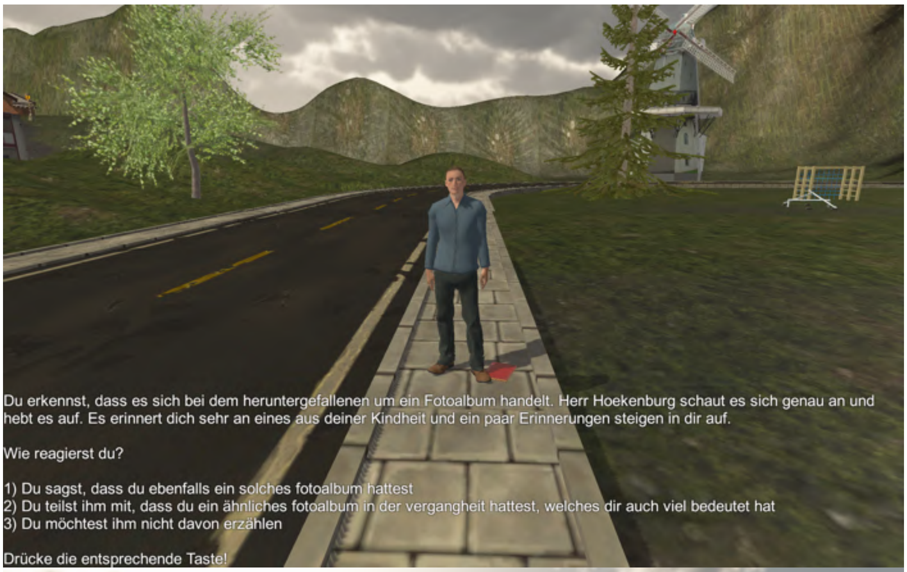
Du erkennst, dass es sich bei dem heruntergefallenen um ein Fotoalbum handelt. Herr Hoekenburg schaut es sich genau an und hebt es auf. Es erinnert dich sehr an eines aus deiner Kindheit und ein paar Erinnerungen steigen in dir auf.
Wie reagierst du?
1) Du sagst, dass du ebenfalls ein solches fotoalbum hattest
2) Du teilst ihm mit, dass du ein ähnliches fotoalbum in der vergangheit hattest, welches dir auch viel bedeutet hat
3) Du möchtest ihm nicht davon erzählen
Drücke die entsprechende Taste!

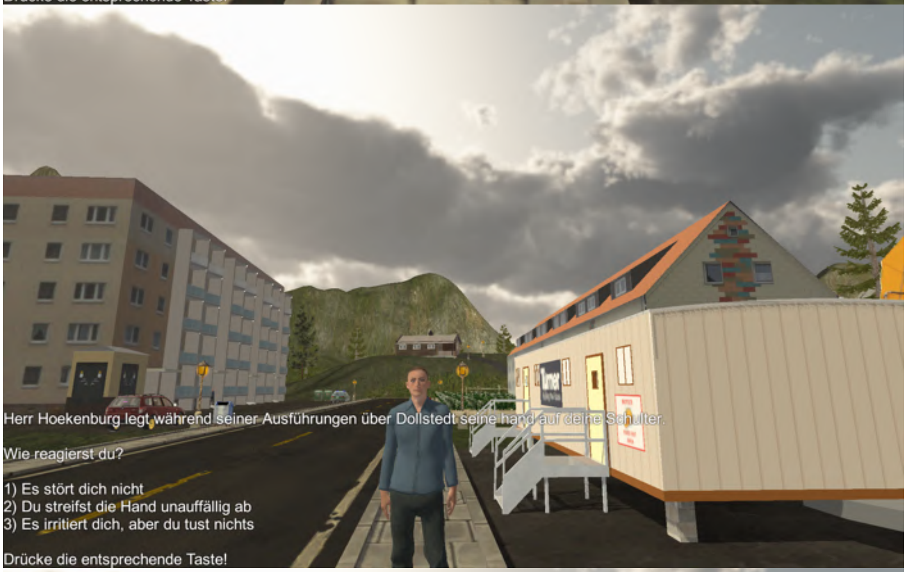
Herr Hoekenburg legt während seiner Ausführungen über Dollstedt seine hand auf deine Schulter.
Wie reagierst du?
1) Es stört dich nicht
2) Du streifst die Hand unauffällig ab
3) Es irritiert dich, aber du tust nichts
Drücke die entsprechende Taste!

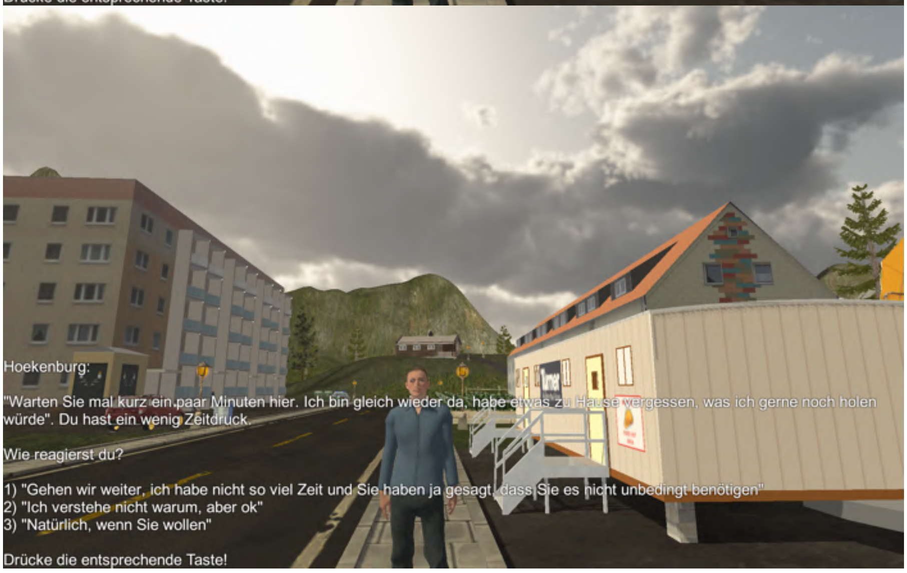
Hoekenburg:
"Warten Sie mal kurz ein paar Minuten hier. Ich bin gleich wieder da, habe etwas zu Hause vergessen, was ich gerne noch holen würde". Du hast ein wenig Zeitdruck.
Wie reagierst du?
1) "Gehen wir weiter, ich habe nicht so viel Zeit und Sie haben ja gesagt, dass Sie es nicht unbedingt benötigen"
2) "Ich verstehe nicht warum, aber ok"
3) "Natürlich, wenn Sie wollen"
Drücke die entsprechende Taste!

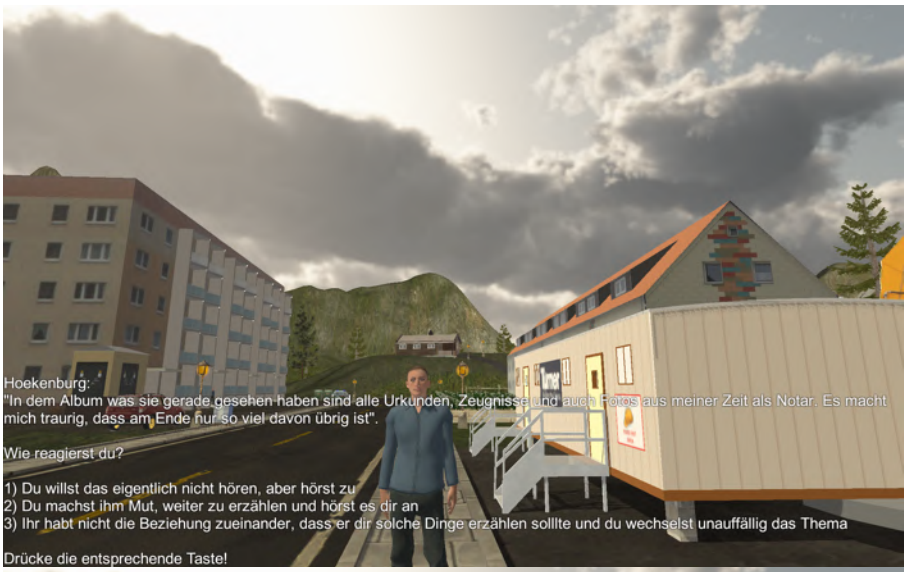
Hoekenburg:
"In dem Album was sie gerade gesehen haben sind alle Urkunden, Zeugnisse und auch Fotos aus meiner Zeit als Notar. Es macht mich traurig, dass am Ende nur so viel davon übrig ist".
Wie reagierst du?
1) Du willst das eigentlich nicht hören, aber hörst zu
2) Du machst ihm Mut, weiter zu erzählen und hörst es dir an
3) Ihr habt nicht die Beziehung zueinander, dass er dir solche Dinge erzählen sollte und du wechselst unauffällig das Thema
Drücke die entsprechende Taste!

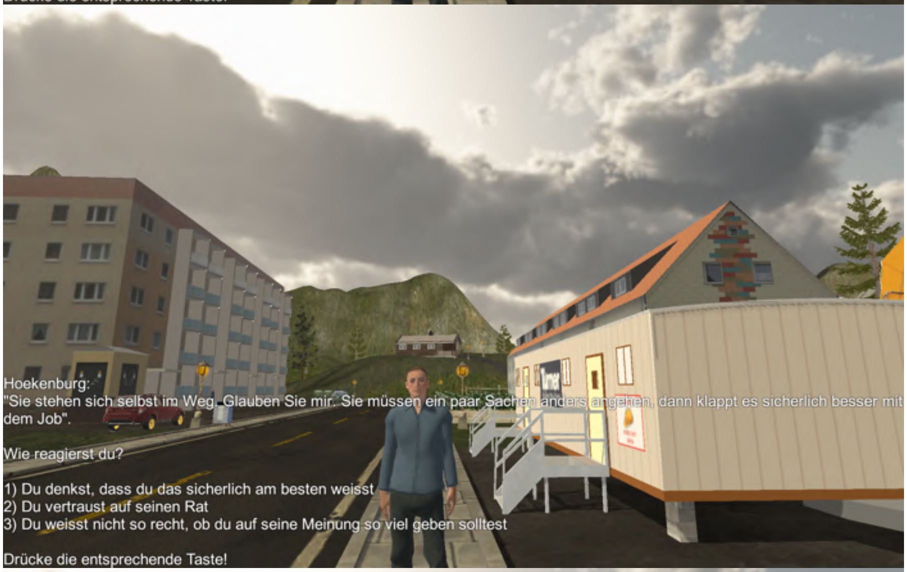
Hoekenburg:
"Sie stehen sich selbst im Weg. Glauben Sie mir. Sie müssen ein paar Sachen anders angehen, dann klappt es sicherlich besser mit dem Job".
Wie reagierst du?
1) Du denkst, dass du das sicherlich am besten weisst
2) Du vertraust auf seinen Rat
3) Du weisst nicht so recht, ob du auf seine Meinung so viel geben solltest
Drücke die entsprechende Taste!

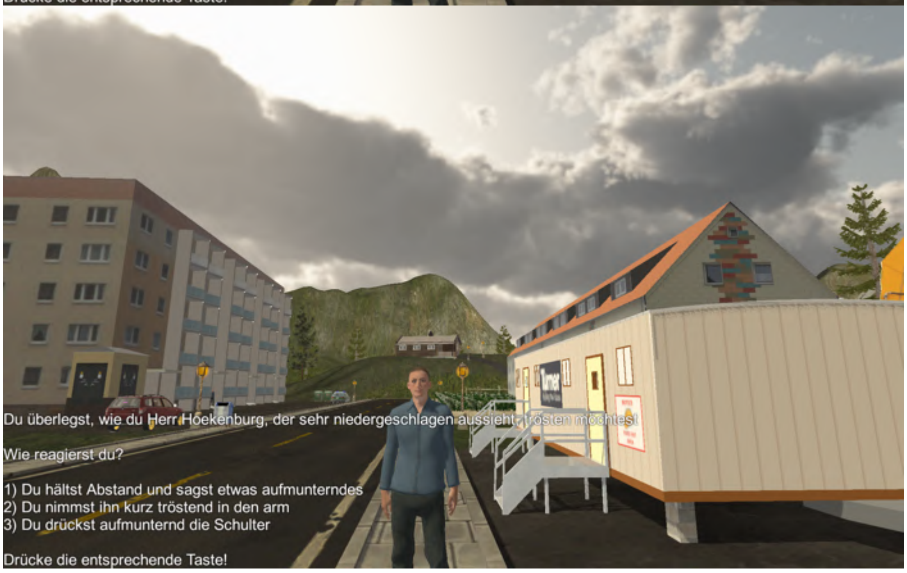
Du überlegst, wie du Herr Hoekenburg, der sehr niedergeschlagen aussieht, trösten möchtest
Wie reagierst du?
1) Du hältst Abstand und sagst etwas aufmunterndes
2) Du nimmst ihn kurz tröstend in den arm
3) Du drückst aufmunternd die Schulter
Drücke die entsprechende Taste!

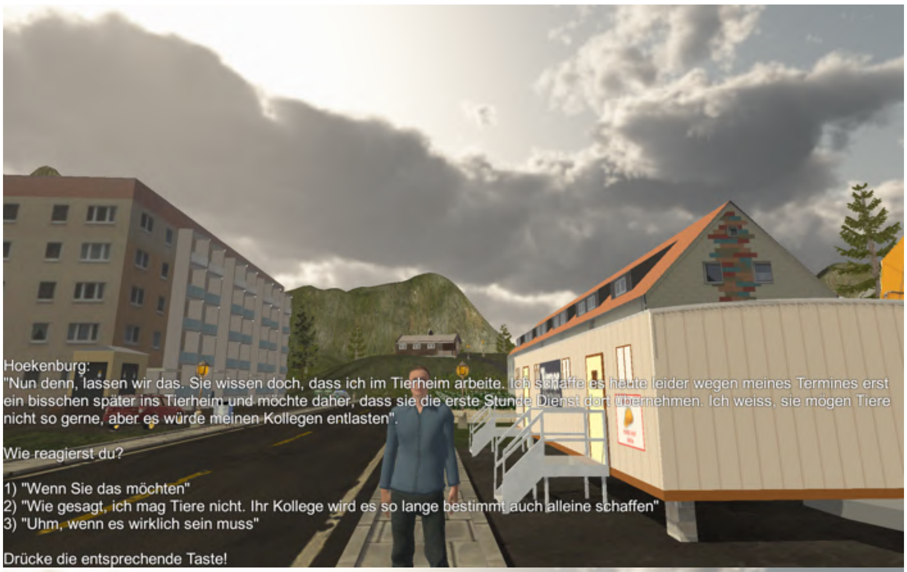
Hoekenburg:
"Nun denn, lassen wir das. Sie wissen doch, dass ich im Tierheim arbeite. Ich schaffe es heute leider wegen meines Termines erst ein bisschen später ins Tierheim und möchte daher, dass sie die erste Stunde Dienst dort übernehmen. Ich weiss, sie mögen Tiere nicht so gerne, aber es würde meinen Kollegen entlasten".
Wie reagierst du?
1) "Wenn Sie das möchten"
2) "Wie gesagt, ich mag Tiere nicht. Ihr Kollege wird es so lange bestimmt auch alleine schaffen"
3) "Uhm, wenn es wirklich sein muss"
Drücke die entsprechende Taste!

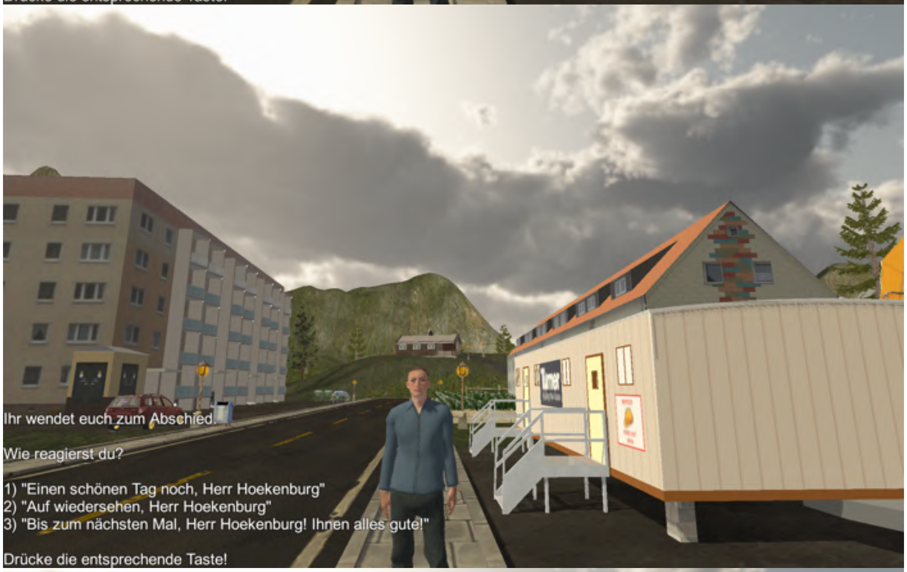
Ihr wendet euch zum Abschied.
Wie reagierst du?
1) "Einen schönen Tag noch, Herr Hoekenburg"
2) "Auf wiedersehen, Herr Hoekenburg"
3) "Bis zum nächsten Mal, Herr Hoekenburg! Ihnen alles gute!"
Drücke die entsprechende Taste!

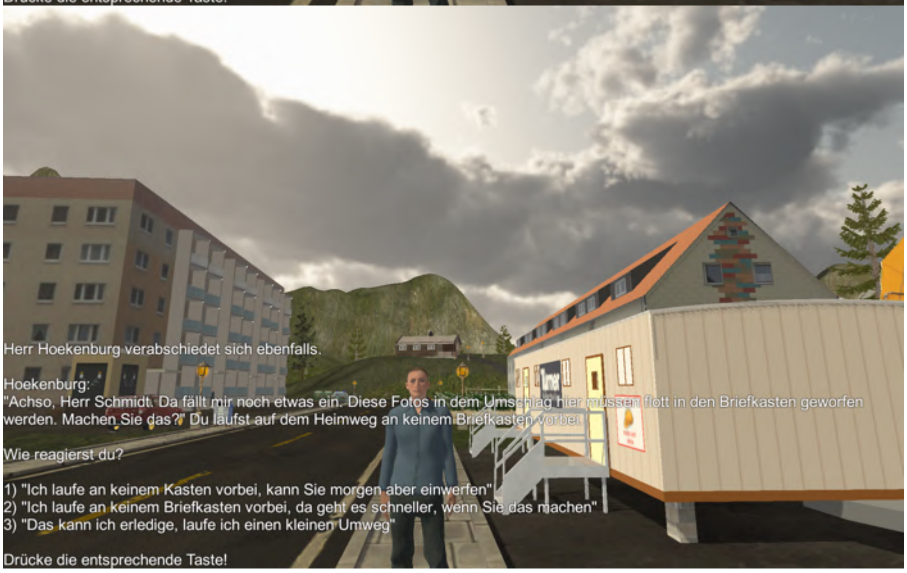
Herr Hoekenburg verabschiedet sich ebenfalls.
Hoekenburg:
"Achso, Herr Schmidt. Da fällt mir noch etwas ein. Diese Fotos in dem Umschlag hier müssen flott in den Briefkasten geworfen werden. Machen Sie das?" Du läufst auf dem Heimweg an keinem Briefkasten vorbei.
Wie reagierst du?
1) "Ich laufe an keinem Kasten vorbei, kann Sie morgen aber einwerfen"
2) "Ich laufe an keinem Briefkasten vorbei, da geht es schneller, wenn Sie das machen"
3) "Das kann ich erledige, laufe ich einen kleinen Umweg"
Drücke die entsprechende Taste!

S5.2.3 Berger (Low-Power/High-Affiliation, Updating-Avatar)

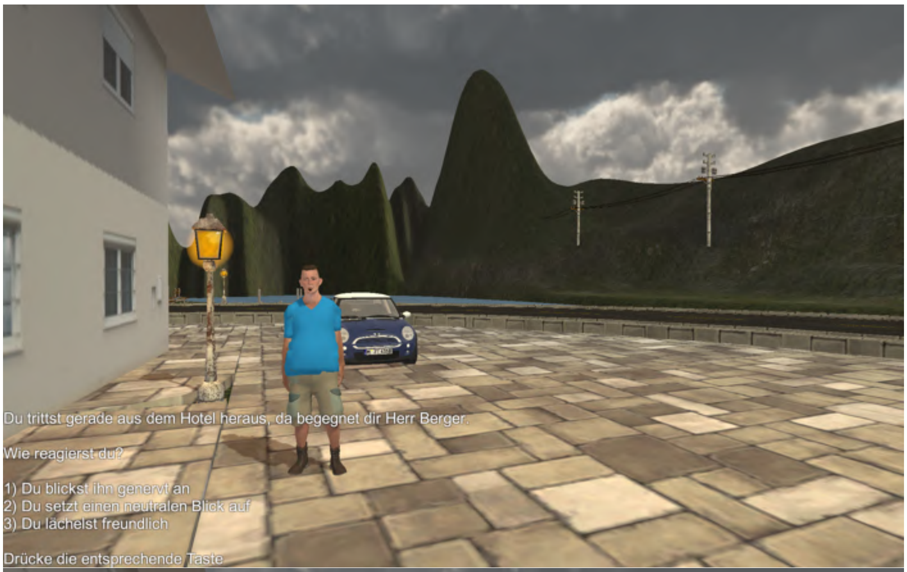

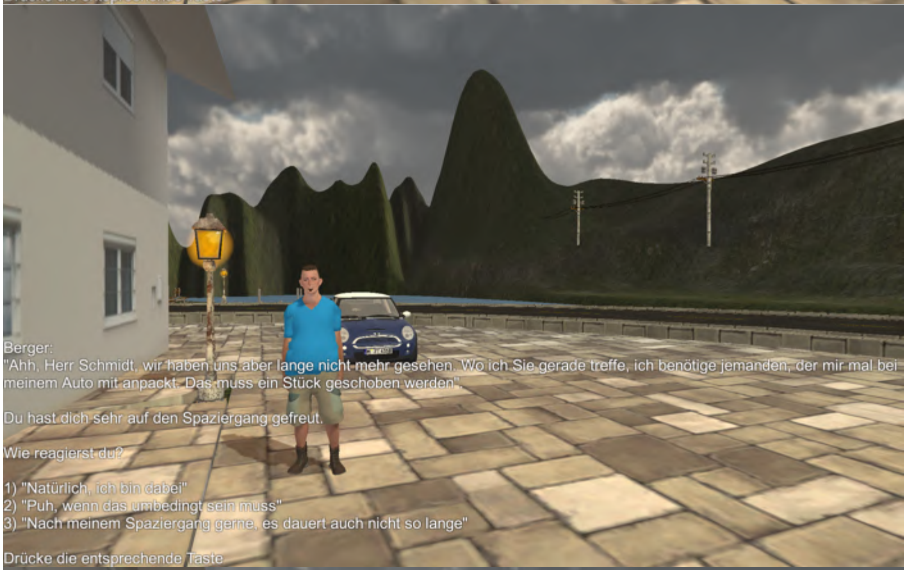

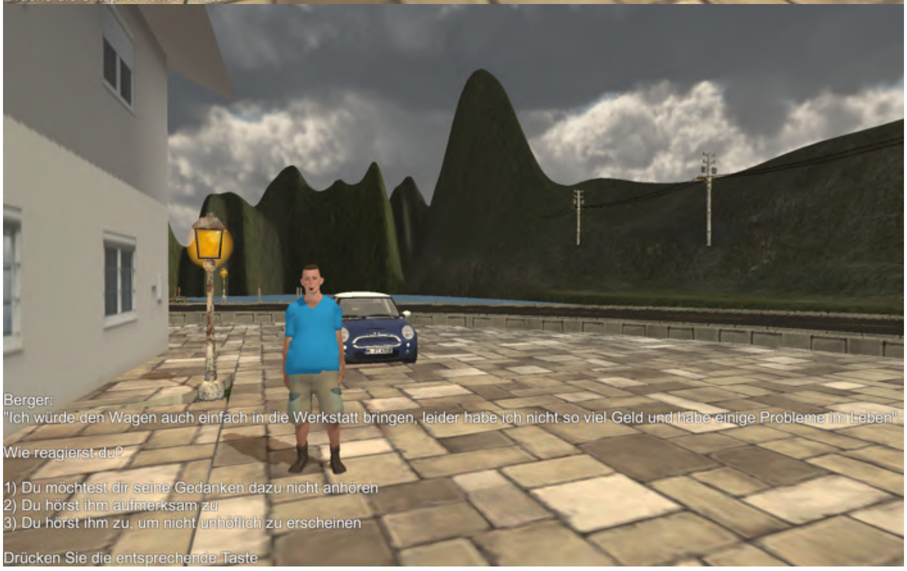

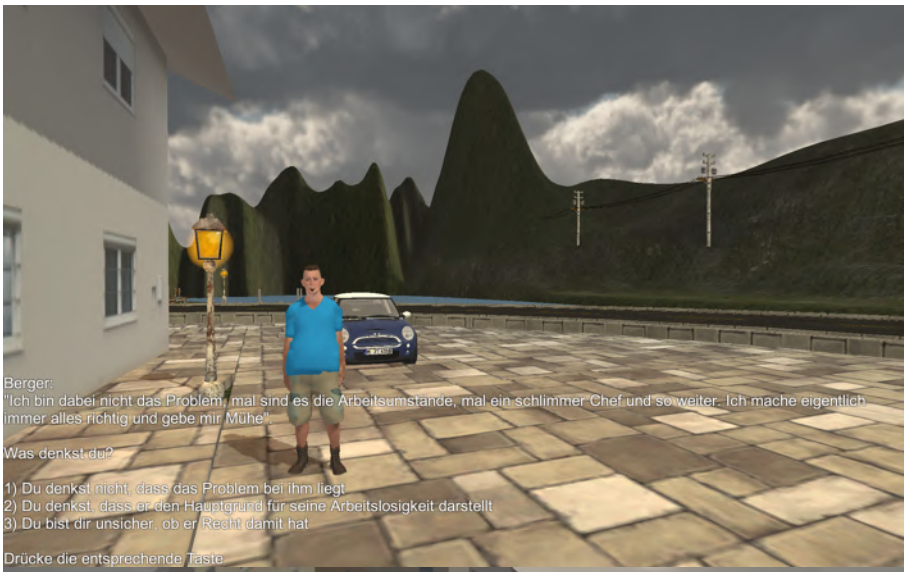
Berger:
"Ich bin dabei nicht das Problem, mal sind es die Arbeitsumstände, mal ein schlimmer Chef und so weiter. Ich mache eigentlich immer alles richtig und gebe mir Mühe".
Was denkst du?
1) Du denkst nicht, dass das Problem bei ihm liegt
2) Du denkst, dass er den Hauptgrund für seine Arbeitslosigkeit darstellt
3) Du bist dir unsicher, ob er Recht damit hat
Drücke die entsprechende Taste

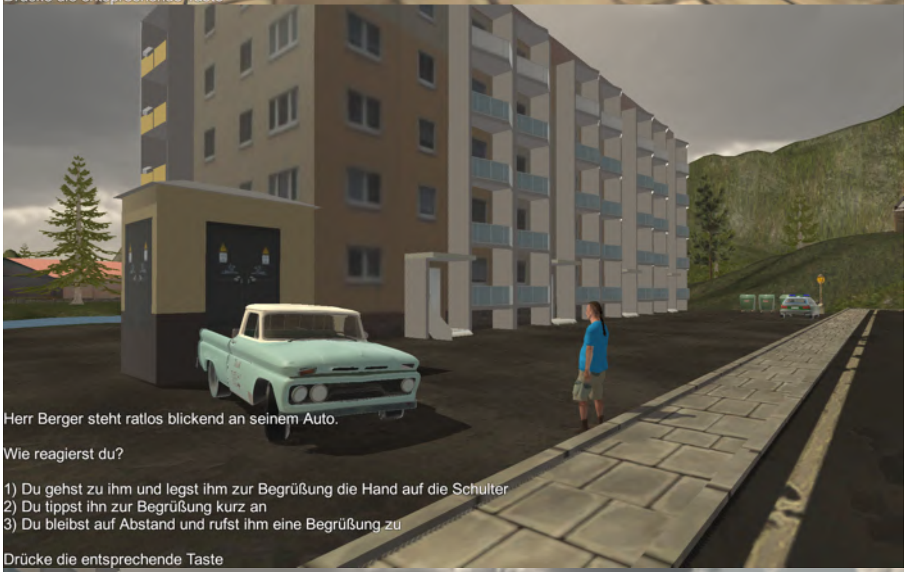
Herr Berger steht ratlos blickend an seinem Auto.
Wie reagierst du?
1) Du gehst zu ihm und legst ihm zur Begrüßung die Hand auf die Schulter
2) Du tippst ihn zur Begrüßung kurz an
3) Du bleibst auf Abstand und rufst ihm eine Begrüßung zu
Drücke die entsprechende Taste

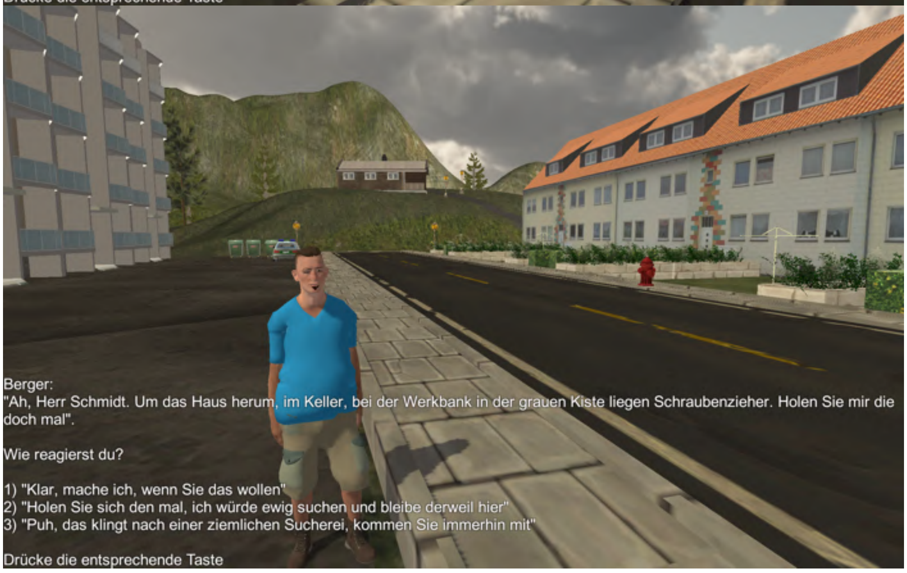
Berger:
"Ah, Herr Schmidt. Um das Haus herum, im Keller, bei der Werkbank in der grauen Kiste liegen Schraubenzieher. Holen Sie mir die doch mal".
Wie reagierst du?
1) "Klar, mache ich, wenn Sie das wollen"
2) "Holen Sie sich den mal, ich würde ewig suchen und bleibe derweil hier"
3) "Puh, das klingt nach einer ziemlichen Sucherei, kommen Sie immerhin mit"
Drücke die entsprechende Taste

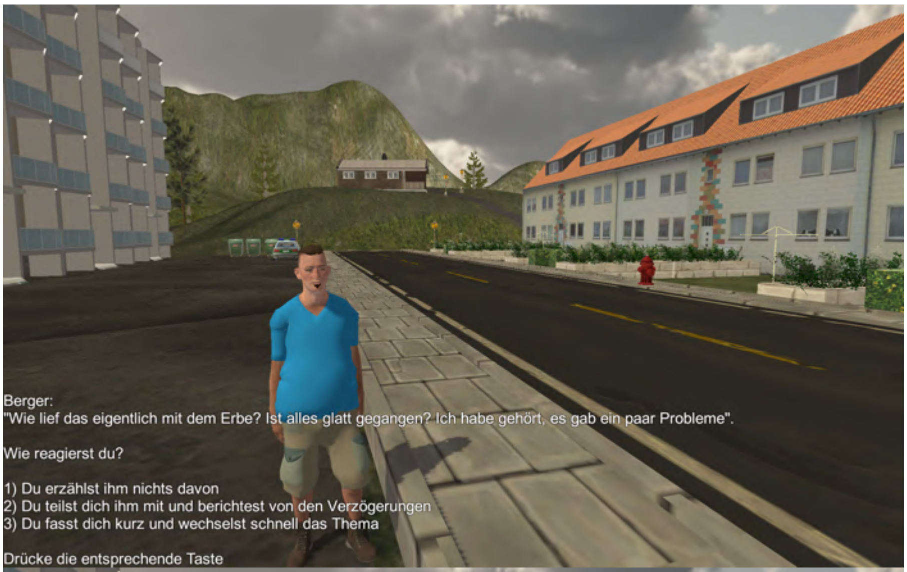
Berger:
"Wie lief das eigentlich mit dem Erbe? Ist alles glatt gegangen? Ich habe gehört, es gab ein paar Probleme".
Wie reagierst du?
1) Du erzählst ihm nichts davon
2) Du teilst dich ihm mit und berichtest von den Verzögerungen
3) Du fasst dich kurz und wechselst schnell das Thema
Drücke die entsprechende Taste

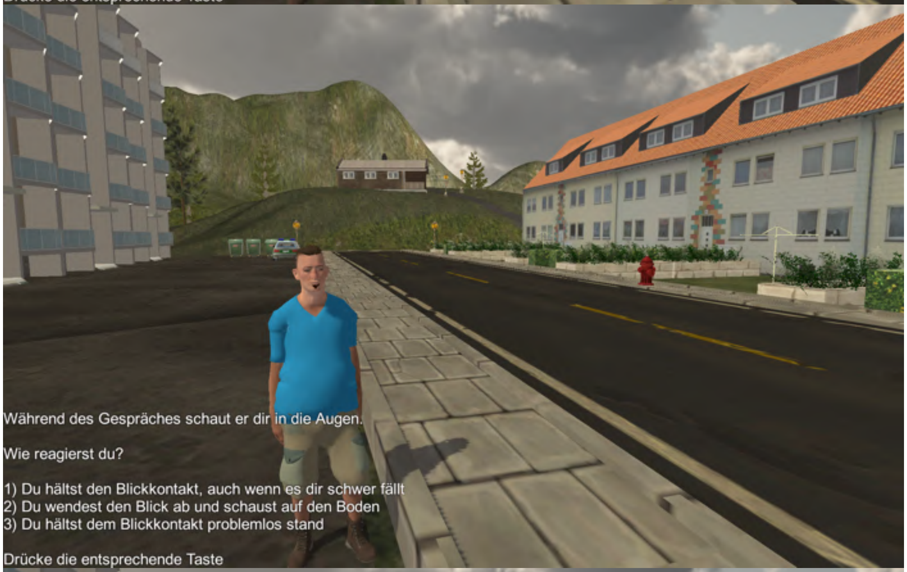
Während des Gespräches schaut er dir in die Augen.
Wie reagierst du?
1) Du hältst den Blickkontakt, auch wenn es dir schwer fällt
2) Du wendest den Blick ab und schaust auf den Boden
3) Du hältst dem Blickkontakt problemlos stand
Drücke die entsprechende Taste

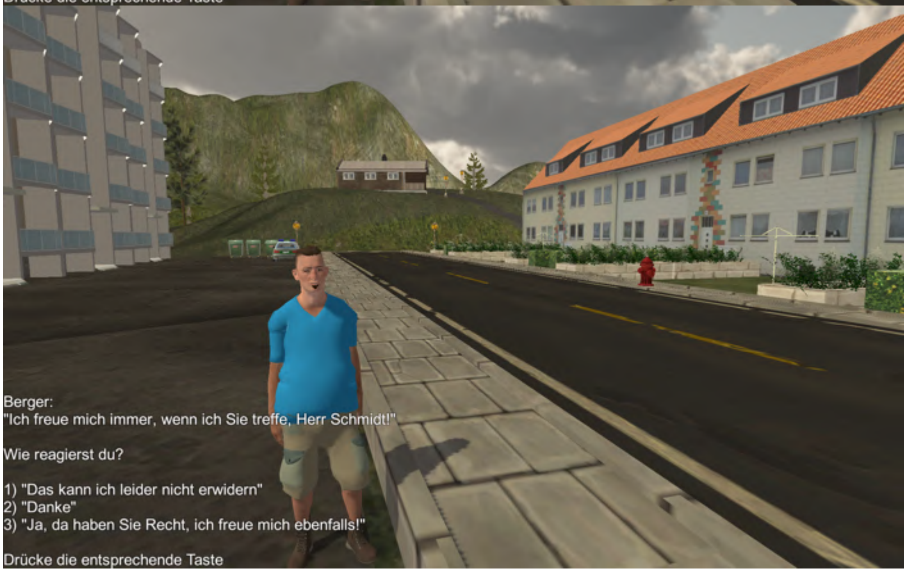
Berger:
"Ich freue mich immer, wenn ich Sie treffe, Herr Schmidt!"
Wie reagierst du?
1) "Das kann ich leider nicht erwidern"
2) "Danke"
3) "Ja, da haben Sie Recht, ich freue mich ebenfalls!"
Drücke die entsprechende Taste

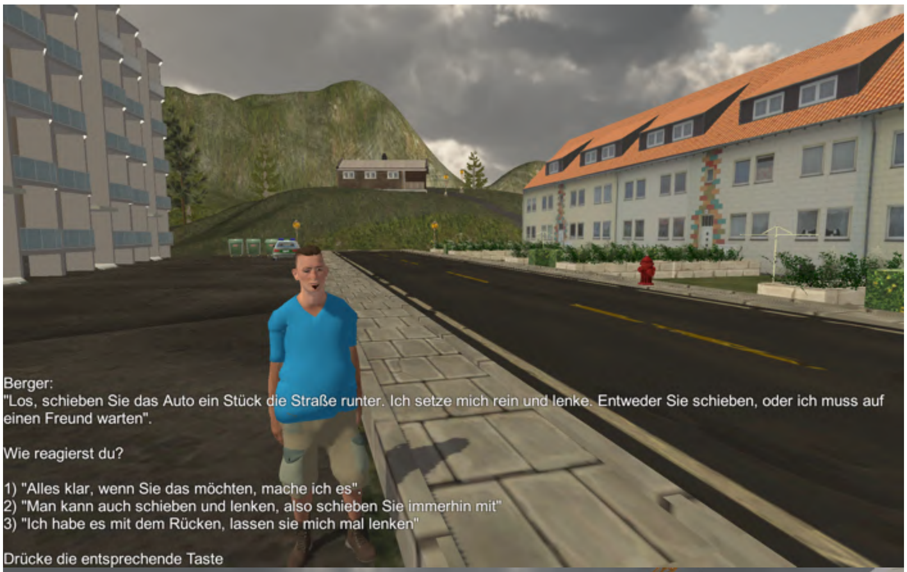
Berger:
"Los, schieben Sie das Auto ein Stück die Straße runter. Ich setze mich rein und lenke. Entweder Sie schieben, oder ich muss auf einen Freund warten".
Wie reagierst du?
1) "Alles klar, wenn Sie das möchten, mache ich es".
2) "Man kann auch schieben und lenken, also schieben Sie immerhin mit"
3) "Ich habe es mit dem Rücken, lassen sie mich mal lenken"
Drücke die entsprechende Taste

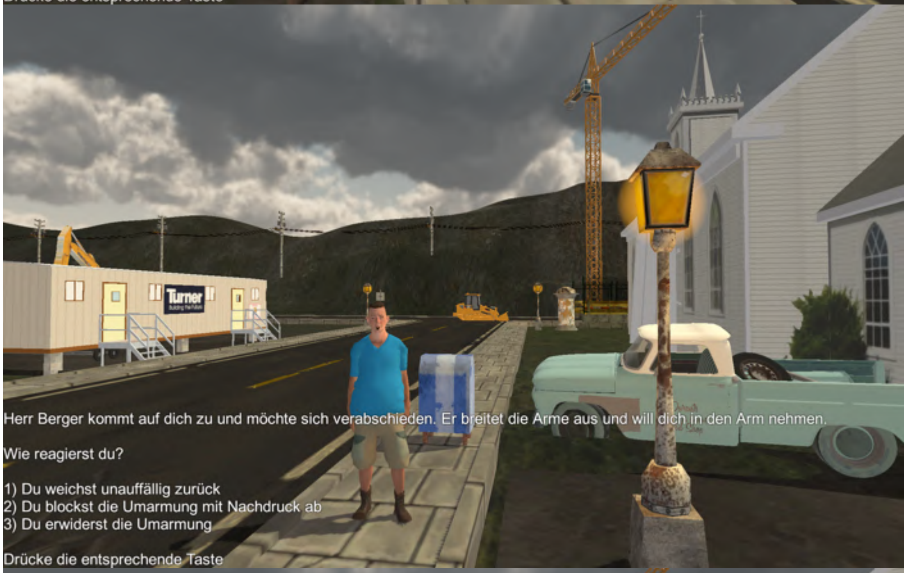
Turner
Herr Berger kommt auf dich zu und möchte sich verabschieden. Er breitet die Arme aus und will dich in den Arm nehmen.
Wie reagierst du?
1) Du weichst unauffällig zurück
2) Du blockst die Umarmung mit Nachdruck ab
3) Du erwiderst die Umarmung
Drücke die entsprechende Taste

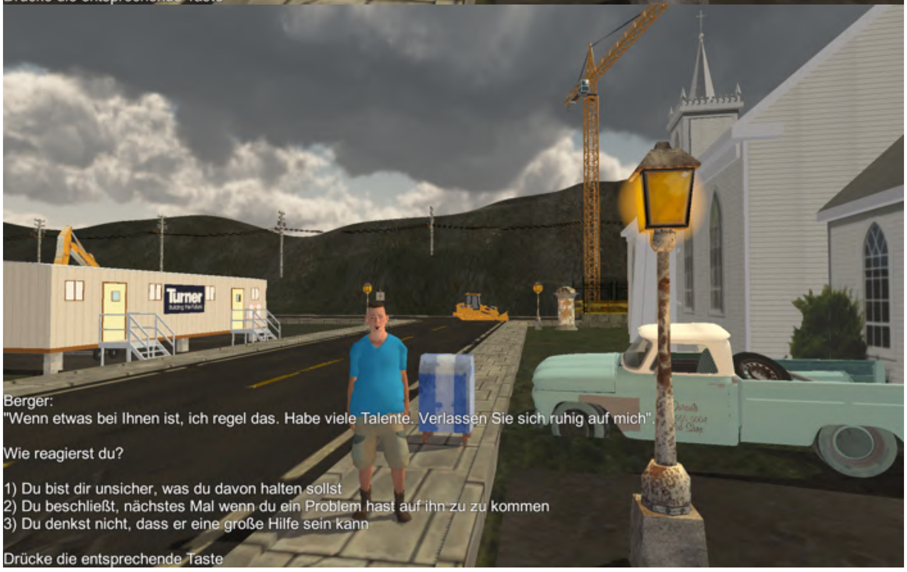
Turner
Berger:
"Wenn etwas bei Ihnen ist, ich regel das. Habe viele Talente. Verlassen Sie sich ruhig auf mich".
Wie reagierst du?
1) Du bist dir unsicher, was du davon halten sollst
2) Du beschließt, nächstes Mal wenn du ein Problem hast auf ihn zu zu kommen
3) Du denkst nicht, dass er eine große Hilfe sein kann
Drücke die entsprechende Taste

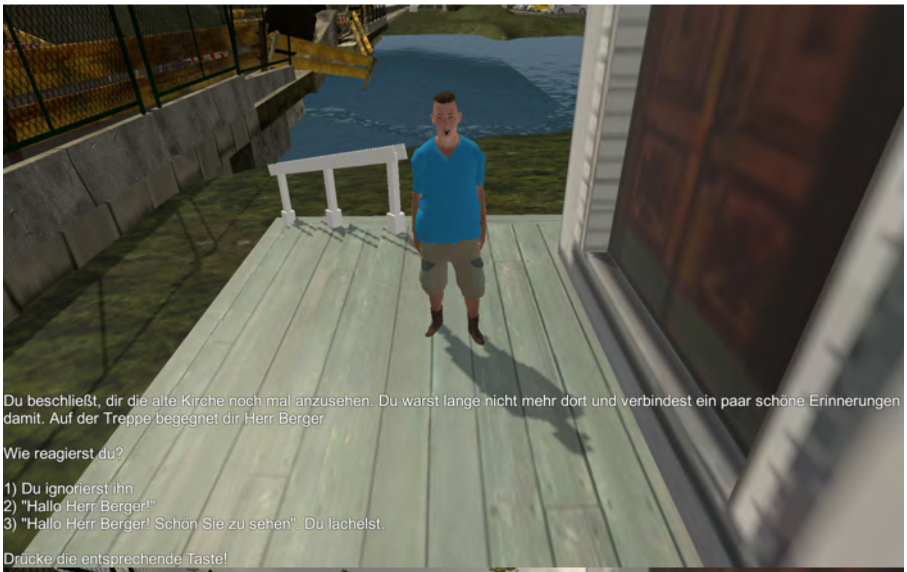
Du beschließt, dir die alte Kirche noch mal anzusehen. Du warst lange nicht mehr dort und verbindest ein paar schöne Erinnerungen damit. Auf der Treppe begegnet dir Herr Berger
Wie reagierst du?
1) Du ignorierst ihn
2) "Hallo Herr Berger!"
3) "Hallo Herr Berger! Schön Sie zu sehen". Du lächelst.
Drücke die entsprechende Taste!

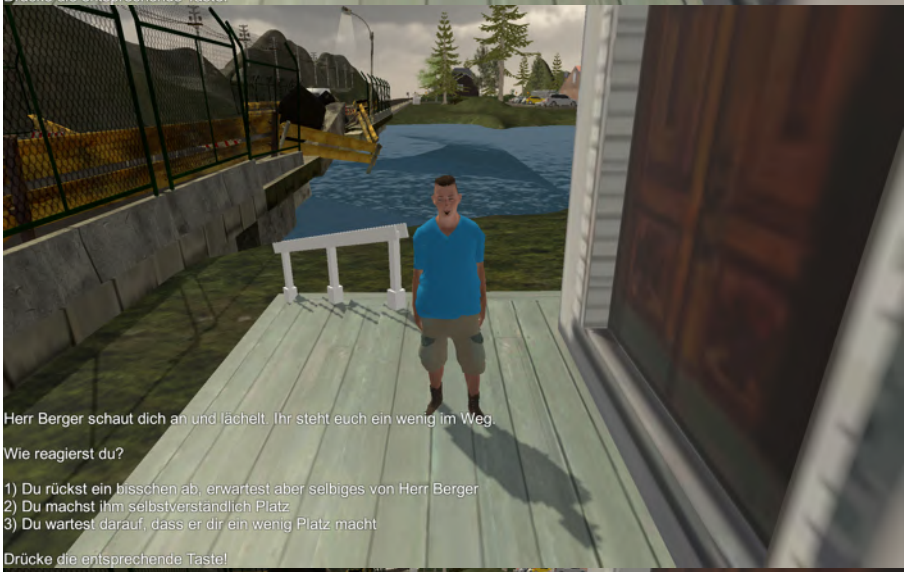
Herr Berger schaut dich an und lächelt. Ihr steht euch ein wenig im Weg.
Wie reagierst du?
1) Du rückst ein bisschen ab, erwartest aber selbiges von Herr Berger
2) Du machst ihm selbstverständlich Platz
3) Du wartest darauf, dass er dir ein wenig Platz macht
Drücke die entsprechende Taste!

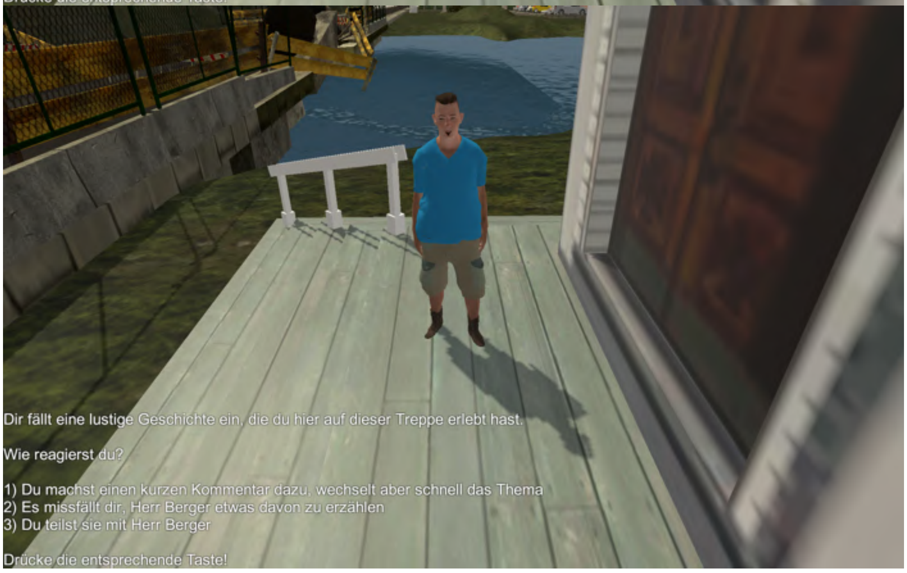
Dir fällt eine lustige Geschichte ein, die du hier auf dieser Treppe erlebt hast.
Wie reagierst du?
1) Du machst einen kurzen Kommentar dazu, wechselt aber schnell das Thema
2) Es missfällt dir, Herr Berger etwas davon zu erzählen
3) Du teilst sie mit Herr Berger
Drücke die entsprechende Taste!

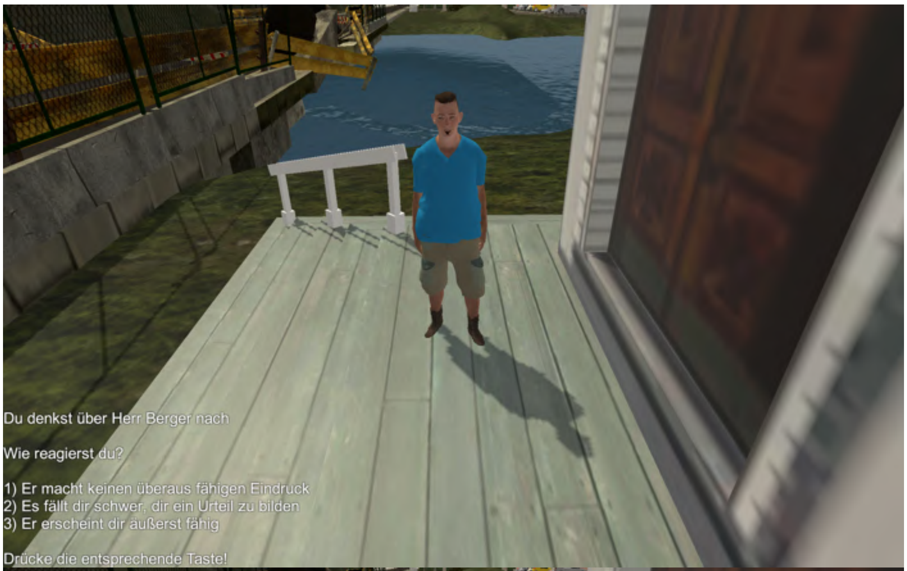
Du denkst über Herr Berger nach
Wie reagierst du?
1) Er macht keinen überaus fähigen Eindruck
2) Es fällt dir schwer, dir ein Urteil zu bilden
3) Er erscheint dir außerst fähig
Drücke die entsprechende Taste!

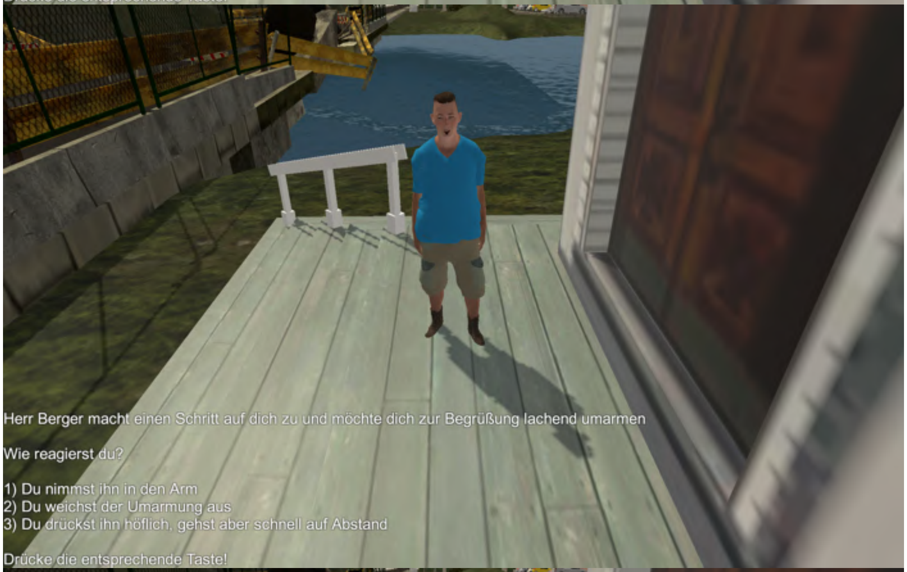
Herr Berger macht einen Schritt auf dich zu und möchte dich zur Begrüßung lachend umarmen
Wie reagierst du?
1) Du nimmst ihn in den Arm
2) Du weichst der Umarmung aus
3) Du drückst ihn höflich, gehst aber schnell auf Abstand
Drücke die entsprechende Taste!

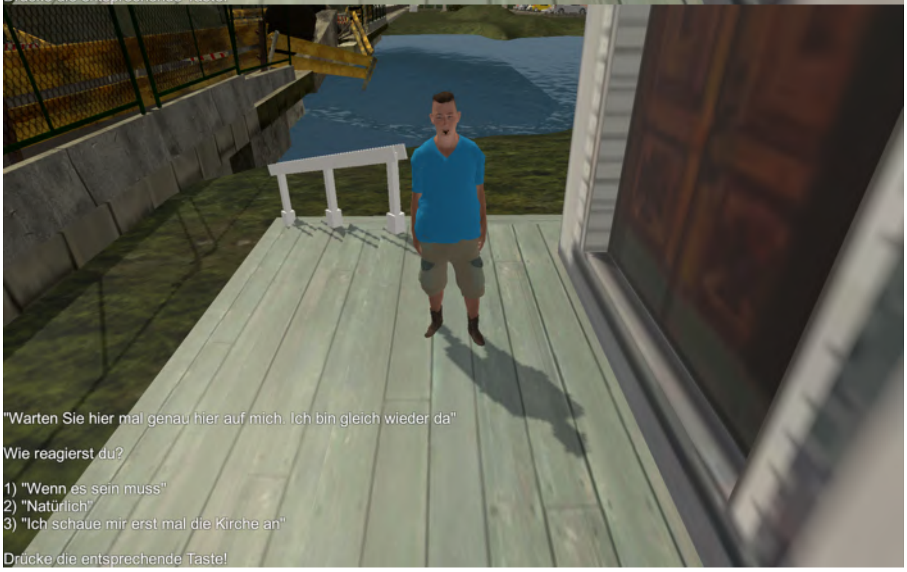
"Warten Sie hier mal genau hier auf mich. Ich bin gleich wieder da"
Wie reagierst du?
1) "Wenn es sein muss"
2) "Natürlich"
3) "Ich schaue mir erst mal die Kirche an"
Drücke die entsprechende Taste!

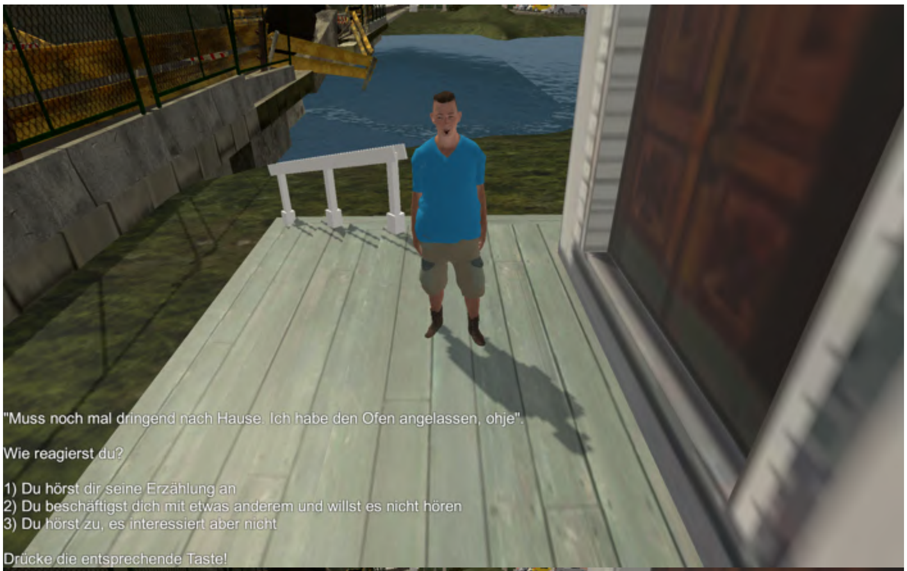
"Muss noch mal dringend nach Hause. Ich habe den Ofen angelassen, ohje".
Wie reagierst du?
1) Du hörst dir seine Erzählung an
2) Du beschäftigst dich mit etwas anderem und willst es nicht hören
3) Du hörst zu, es interessiert aber nicht
Drücke die entsprechende Taste!
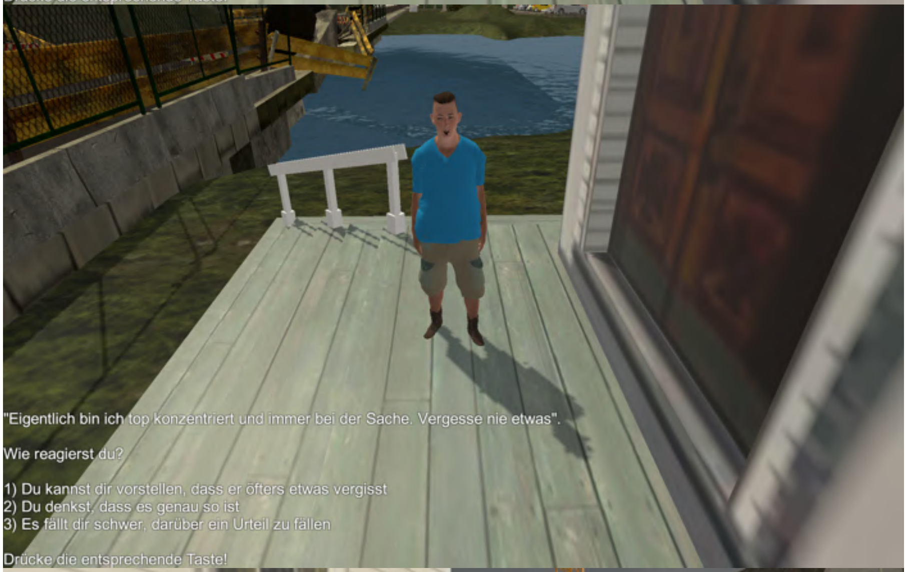
"Eigentlich bin ich top konzentriert und immer bei der Sache. Vergesse nie etwas".
Wie reagierst du?
1) Du kannst dir vorstellen, dass er öfters etwas vergisst
2) Du denkst, dass es genau so ist
3) Es fällt dir schwer, darüber ein Urteil zu fällen
Drücke die entsprechende Taste!
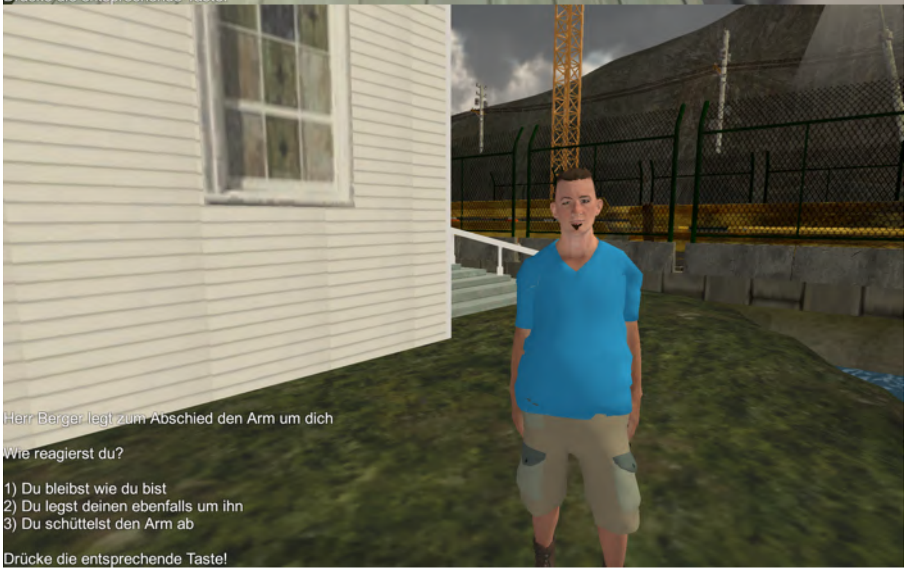
Herr Berger legt zum Abschied den Arm um dich
Wie reagierst du?
1) Du bleibst wie du bist
2) Du legst deinen ebenfalls um ihn
3) Du schüttelst den Arm ab
Drücke die entsprechende Taste!

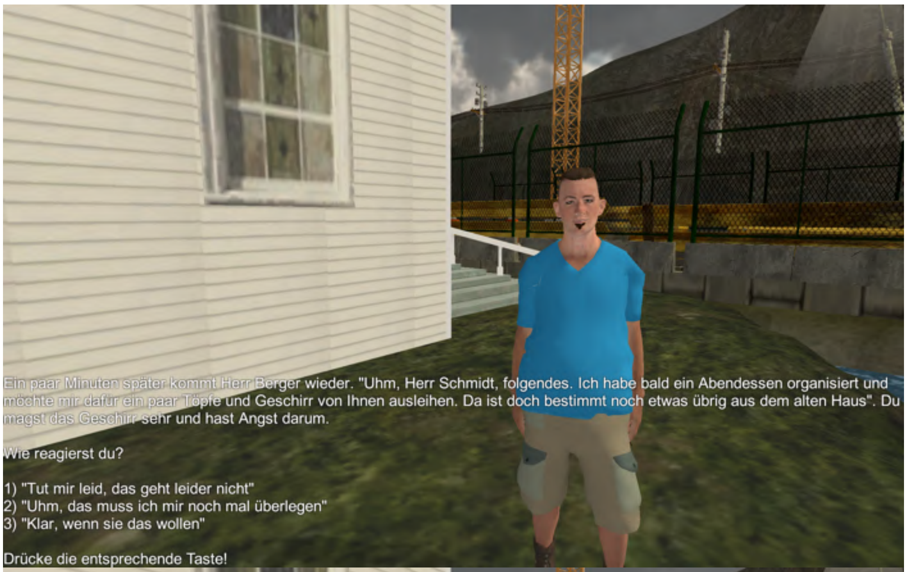
Ein paar Minuten später kommt Herr Berger wieder. "Uhm, Herr Schmidt, folgendes. Ich habe bald ein Abendessen organisiert und möchte mir dafür ein paar Töpfe und Geschirr von Ihnen ausleihen. Da ist doch bestimmt noch etwas übrig aus dem alten Haus". Du magst das Geschirr sehr und hast Angst darum.
Wie reagierst du?
1) "Tut mir leid, das geht leider nicht"
2) "Uhm, das muss ich mir noch mal überlegen"
3) "Klar, wenn sie das wollen"
Drücke die entsprechende Taste!

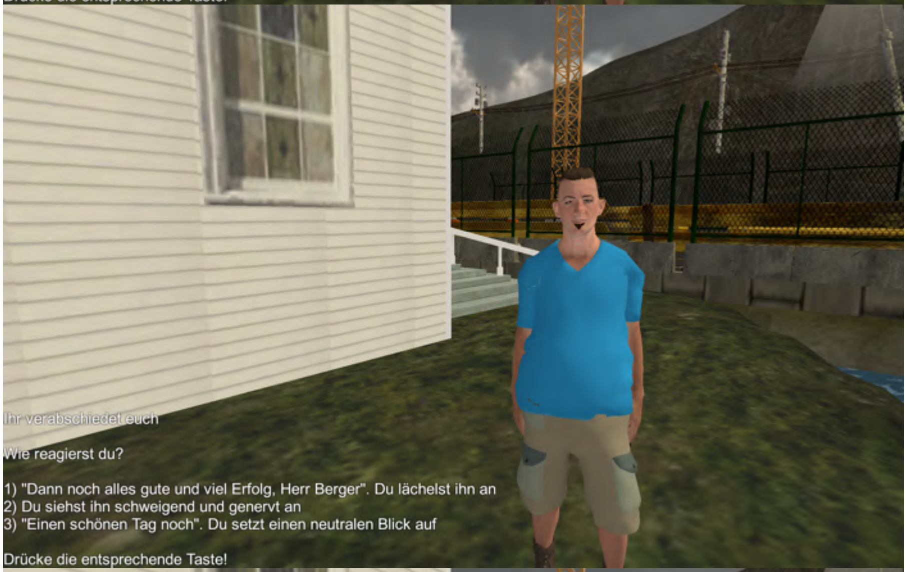
Ihr verabschiedet euch
Wie reagierst du?
1) "Dann noch alles gute und viel Erfolg, Herr Berger". Du lächelst ihn an
2) Du siehst ihn schweigend und genervt an
3) "Einen schönen Tag noch". Du setzt einen neutralen Blick auf
Drücke die entsprechende Taste!

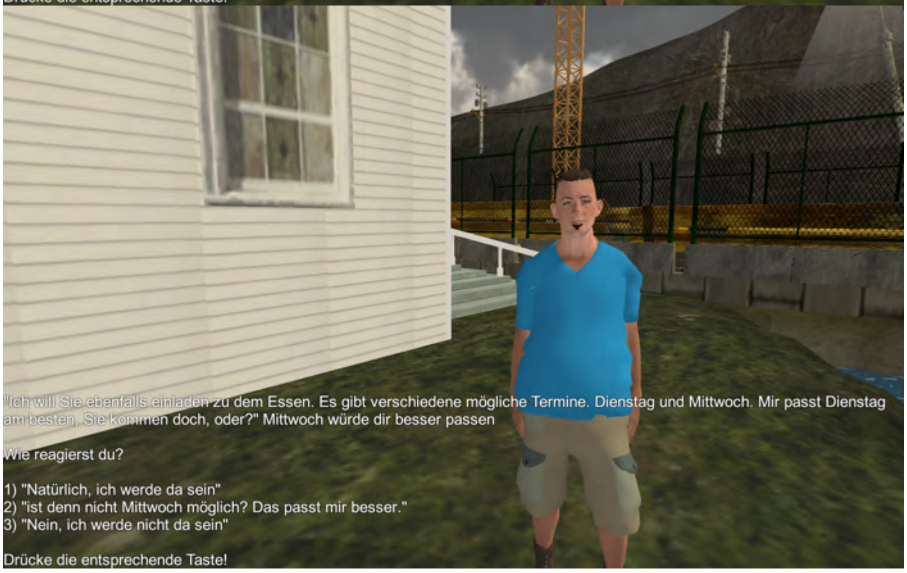
"Ich will Sie ebenfalls einladen zu dem Essen. Es gibt verschiedene mögliche Termine. Dienstag und Mittwoch. Mir passt Dienstag am besten. Sie kommen doch, oder?" Mittwoch würde dir besser passen
Wie reagierst du?
1) "Natürlich, ich werde da sein"
2) "ist denn nicht Mittwoch möglich? Das passt mir besser."
3) "Nein, ich werde nicht da sein"
Drücke die entsprechende Taste!

S5.2.4 Karoske (Low-Power/High-Affiliation, Remapping-Avatar)

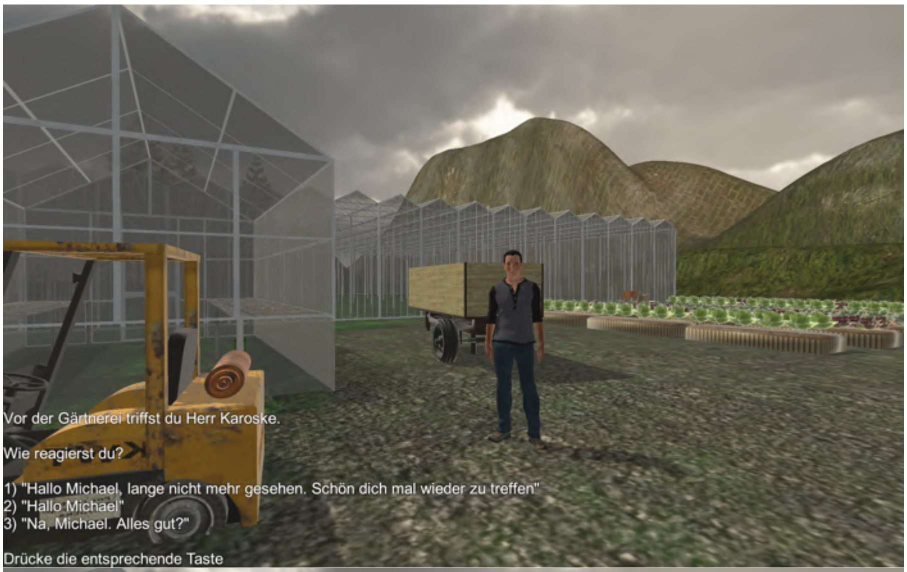

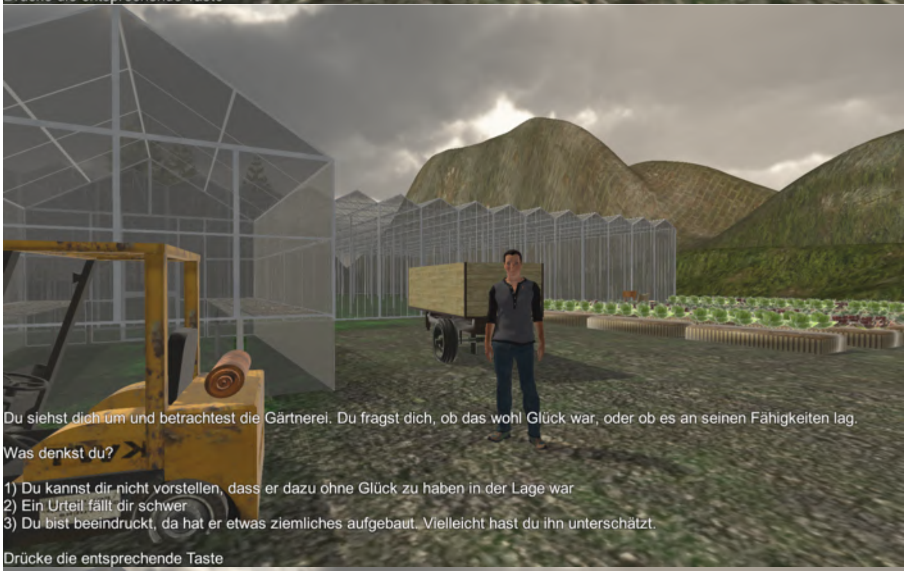

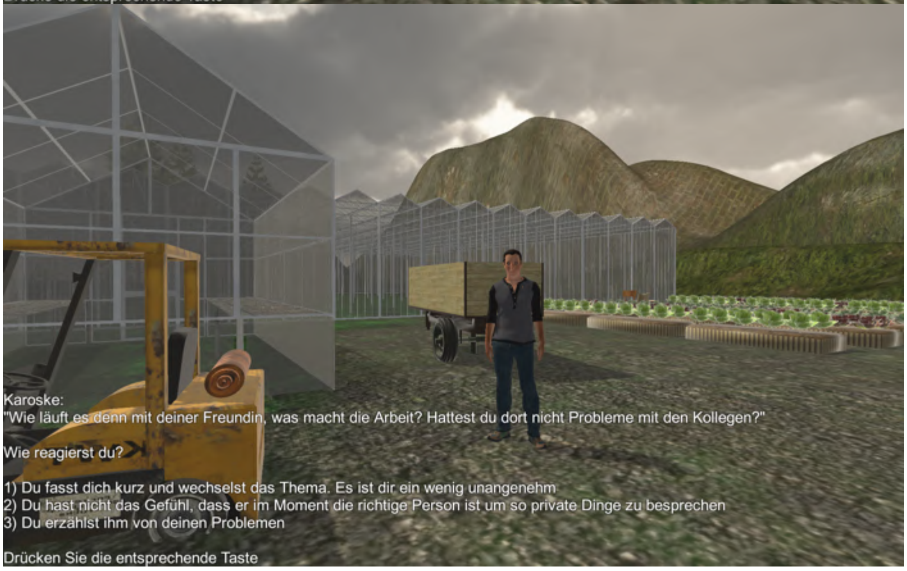

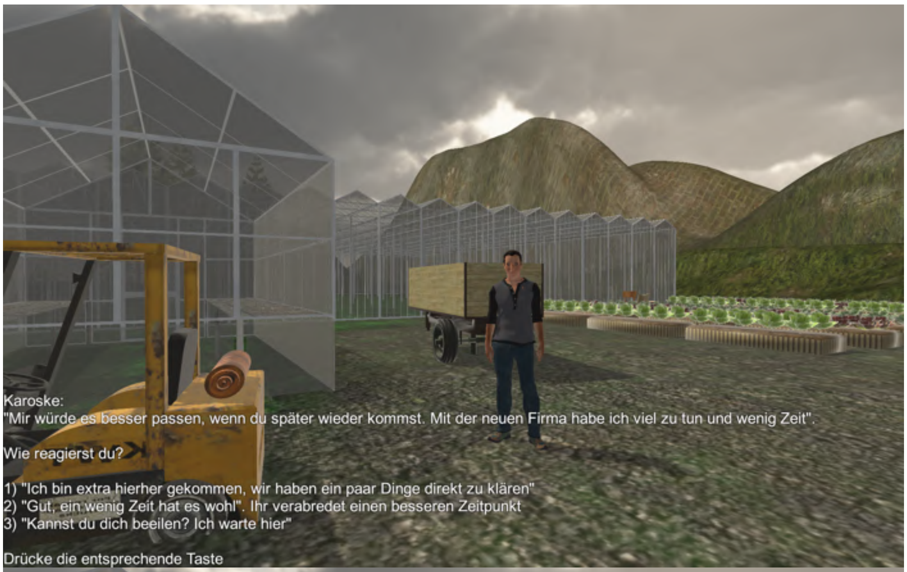
Karoske:
"Mir würde es besser passen, wenn du später wieder kommst. Mit der neuen Firma habe ich viel zu tun und wenig Zeit".
Wie reagierst du?
1) "Ich bin extra hierher gekommen, wir haben ein paar Dinge direkt zu klären"
2) "Gut, ein wenig Zeit hat es wohl". Ihr verabredet einen besseren Zeitpunkt
3) "Kannst du dich beeilen? Ich warte hier"
Drücke die entsprechende Taste

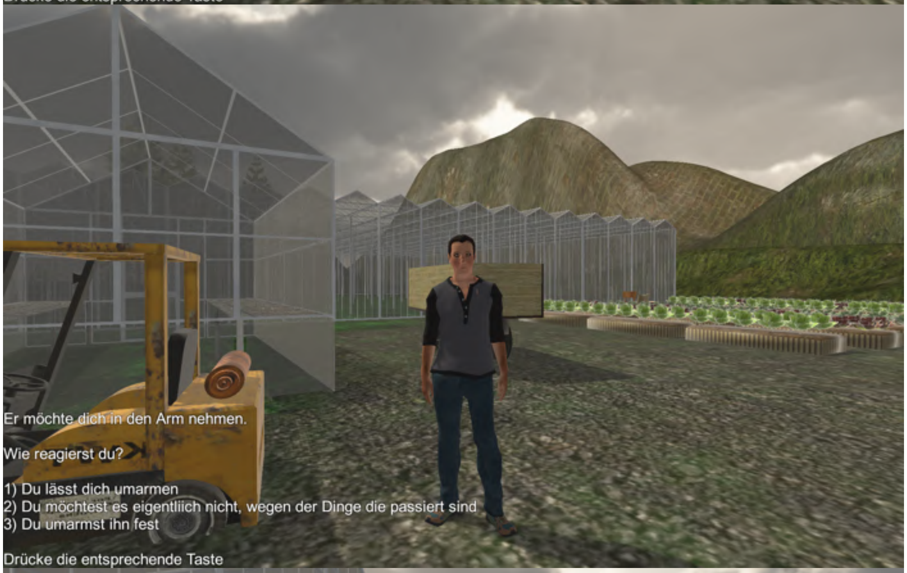
Er möchte dich in den Arm nehmen.
Wie reagierst du?
1) Du lässt dich umarmen
2) Du möchtest es eigentlich nicht, wegen der Dinge die passiert sind
3) Du umarmst ihn fest
Drücke die entsprechende Taste

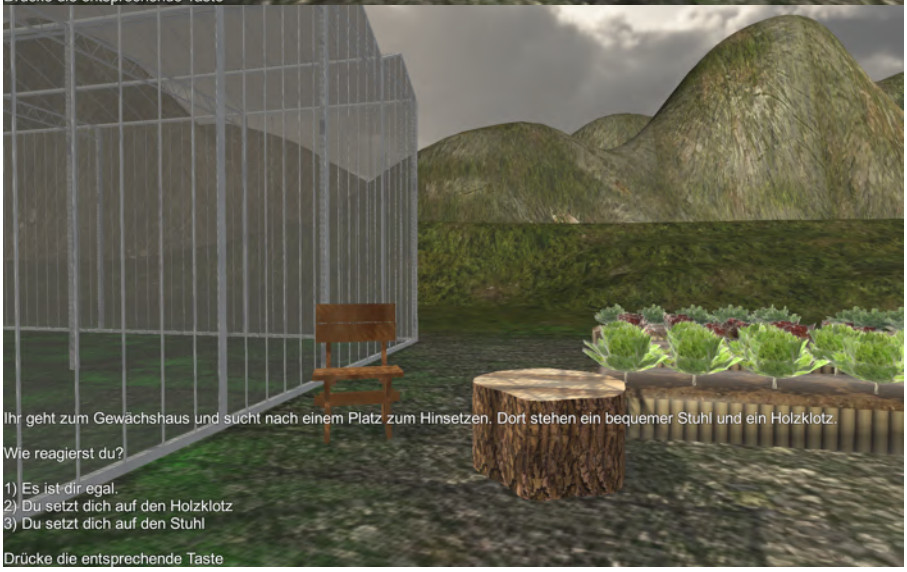
Ihr geht zum Gewächshaus und sucht nach einem Platz zum Hinsetzen. Dort stehen ein bequemer Stuhl und ein Holzklotz.
Wie reagierst du?
1) Es ist dir egal.
2) Du setzt dich auf den Holzklotz
3) Du setzt dich auf den Stuhl
Drücke die entsprechende Taste

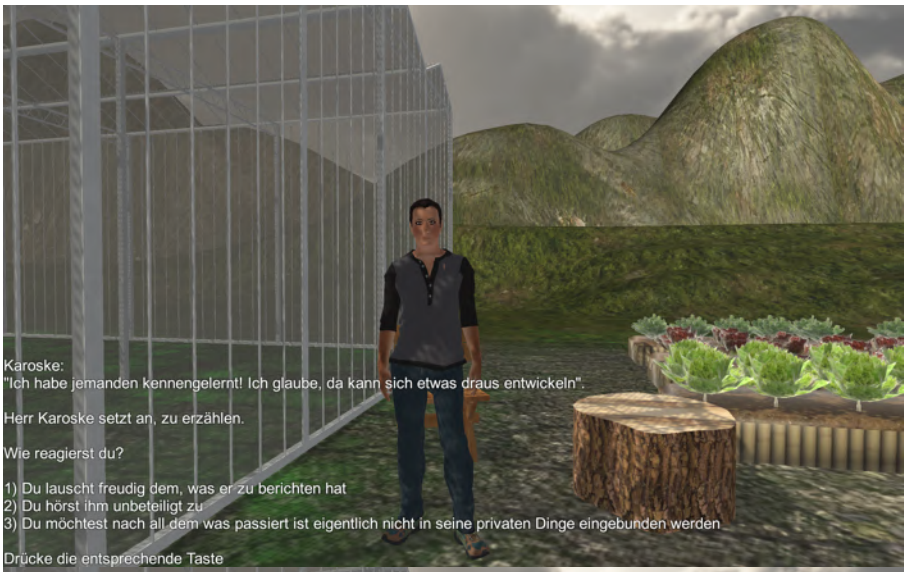
Karoske:
"Ich habe jemanden kennengelernt! Ich glaube, da kann sich etwas draus entwickeln".
Herr Karoske setzt an, zu erzählen.
Wie reagierst du?
1) Du lauscht freudig dem, was er zu berichten hat
2) Du hörst ihm unbeteiligt zu
3) Du möchtest nach all dem was passiert ist eigentlich nicht in seine privaten Dinge eingebunden werden
Drücke die entsprechende Taste

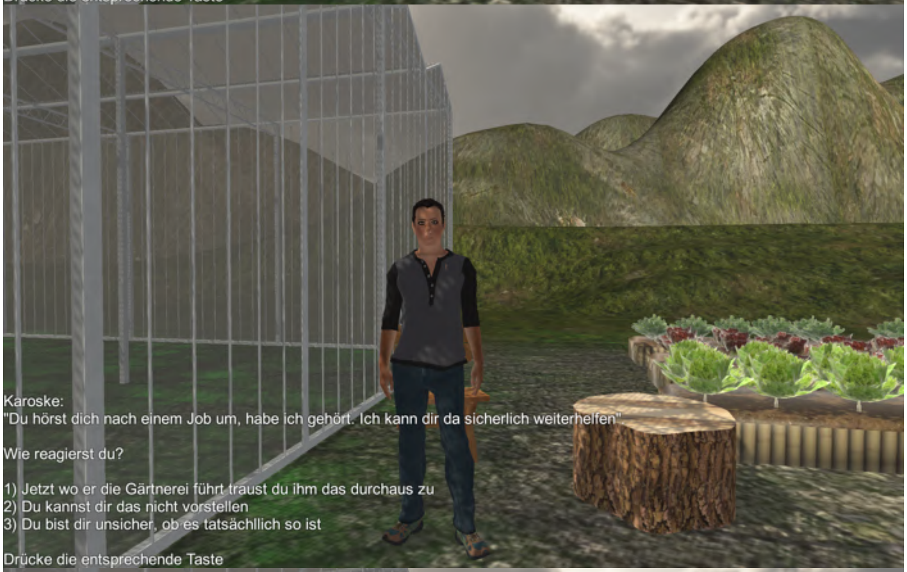
Karoske:
"Du hörst dich nach einem Job um, habe ich gehört. Ich kann dir da sicherlich weiterhelfen"
Wie reagierst du?
1) Jetzt wo er die Gärtnerei führt traust du ihm das durchaus zu
2) Du kannst dir das nicht vorstellen
3) Du bist dir unsicher, ob es tatsächllich so ist
Drücke die entsprechende Taste

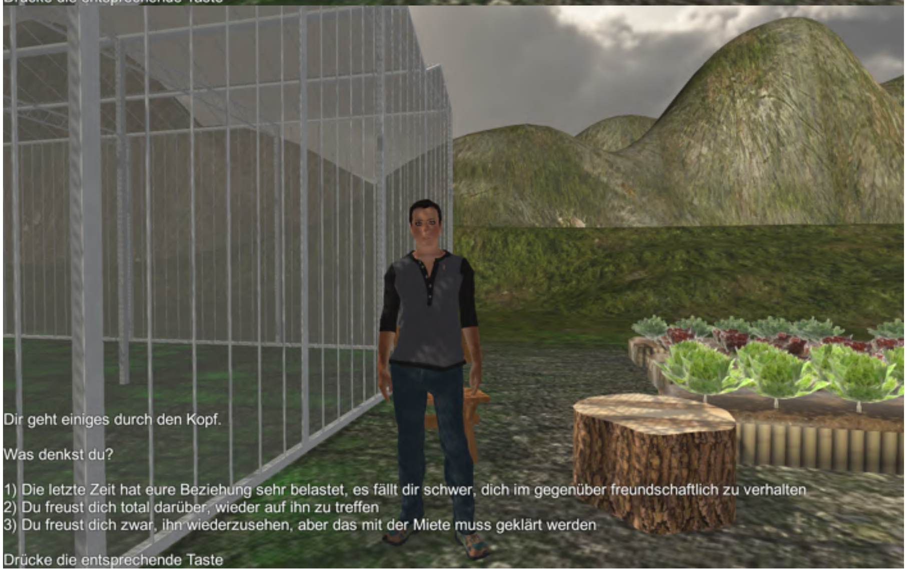
Dir geht einiges durch den Kopf.
Was denkst du?
1) Die letzte Zeit hat eure Beziehung sehr belastet, es fällt dir schwer, dich im gegenüber freundschaftlich zu verhalten
2) Du freust dich total darüber, wieder auf ihn zu treffen
3) Du freust dich zwar, ihn wiederzusehen, aber das mit der Miete muss geklärt werden
Drücke die entsprechende Taste

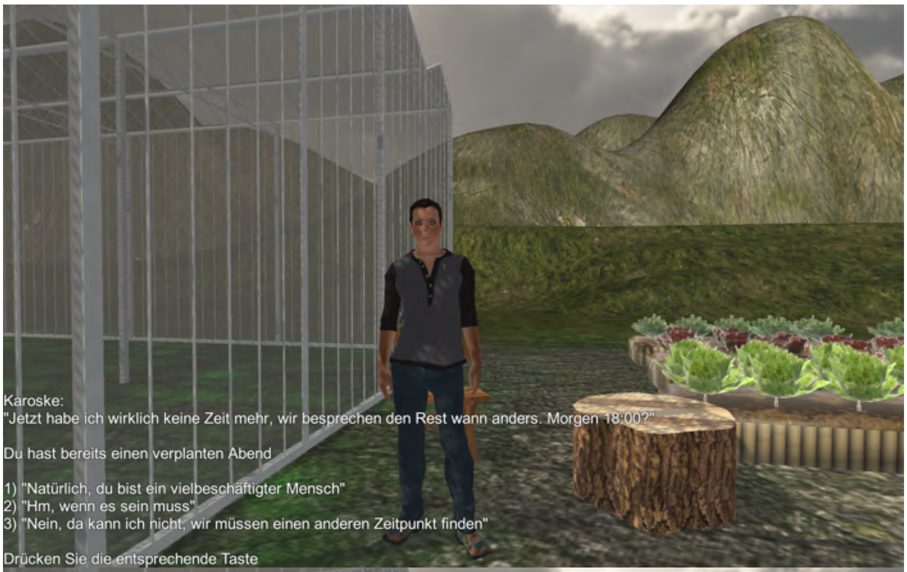
Karoske:
"Jetzt habe ich wirklich keine Zeit mehr, wir besprechen den Rest wann anders. Morgen 18:00?"
Du hast bereits einen verplanten Abend
1) "Natürlich, du bist ein vielbeschäftigter Mensch"
2) "Hm, wenn es sein muss"
3) "Nein, da kann ich nicht, wir müssen einen anderen Zeitpunkt finden"
Drücken Sie die entsprechende Taste

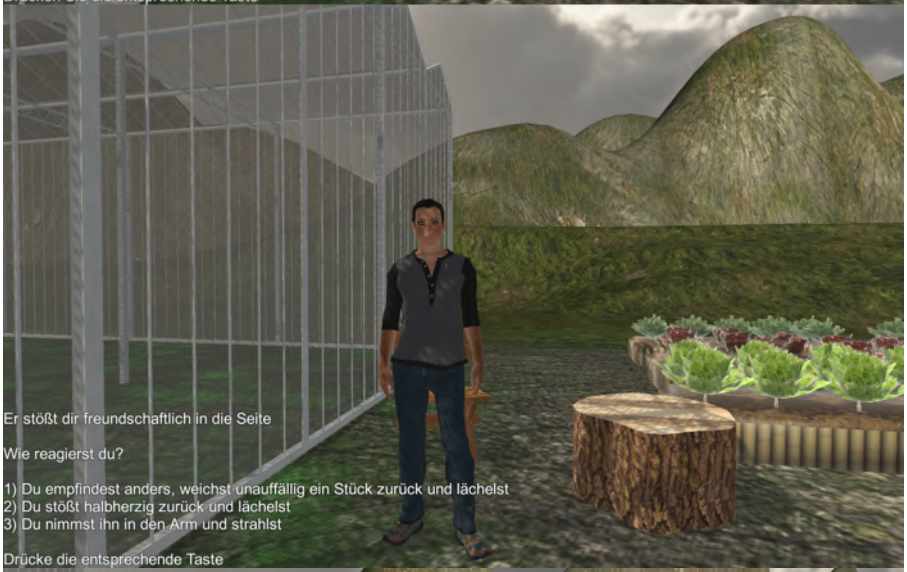
Er stößt dir freundschaftlich in die Seite
Wie reagierst du?
1) Du empfindest anders, weichst unauffällig ein Stück zurück und lächelst
2) Du stößt halbherzig zurück und lächelst
3) Du nimmst ihn in den Arm und strahlst
Drücke die entsprechende Taste

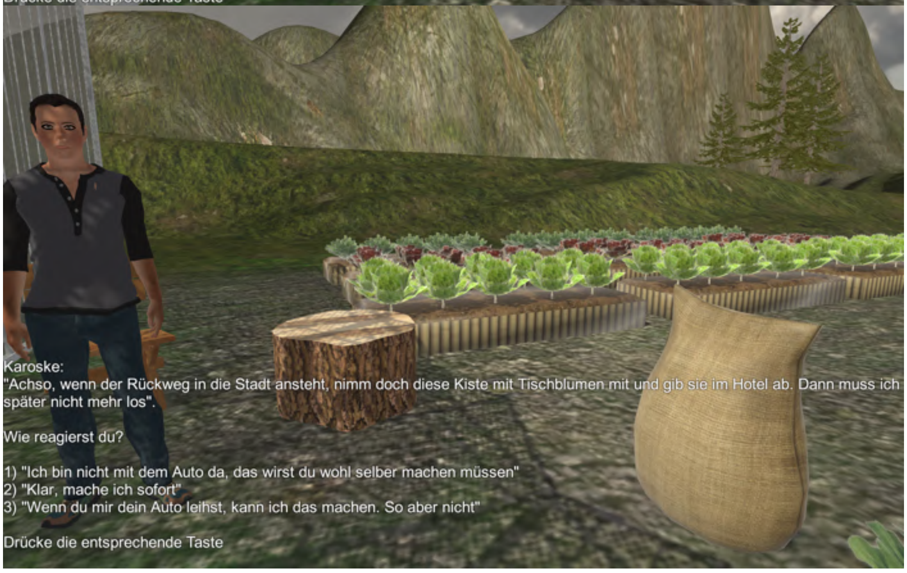
Karoske:
"Achso, wenn der Rückweg in die Stadt ansteht, nimm doch diese Kiste mit Tischblumen mit und gib sie im Hotel ab. Dann muss ich später nicht mehr los".
Wie reagierst du?
1) "Ich bin nicht mit dem Auto da, das wirst du wohl selber machen müssen"
2) "Klar, mache ich sofort"
3) "Wenn du mir dein Auto leihst, kann ich das machen. So aber nicht"
Drücke die entsprechende Taste

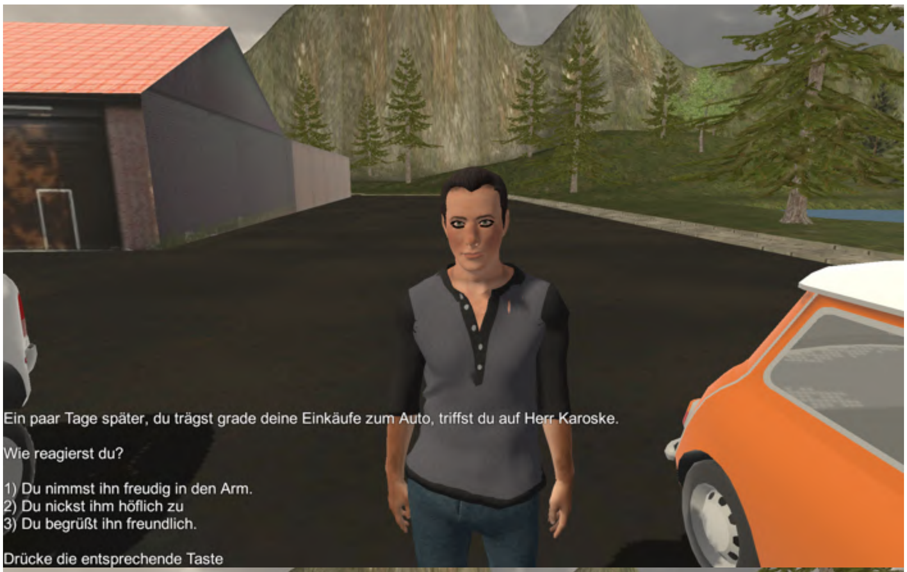
Ein paar Tage später, du trägst grade deine Einkäufe zum Auto, triffst du auf Herr Karoske.
Wie reagierst du?
1) Du nimmst ihn freudig in den Arm.
2) Du nickst ihm höflich zu
3) Du begrüßt ihn freundlich.
Drücke die entsprechende Taste

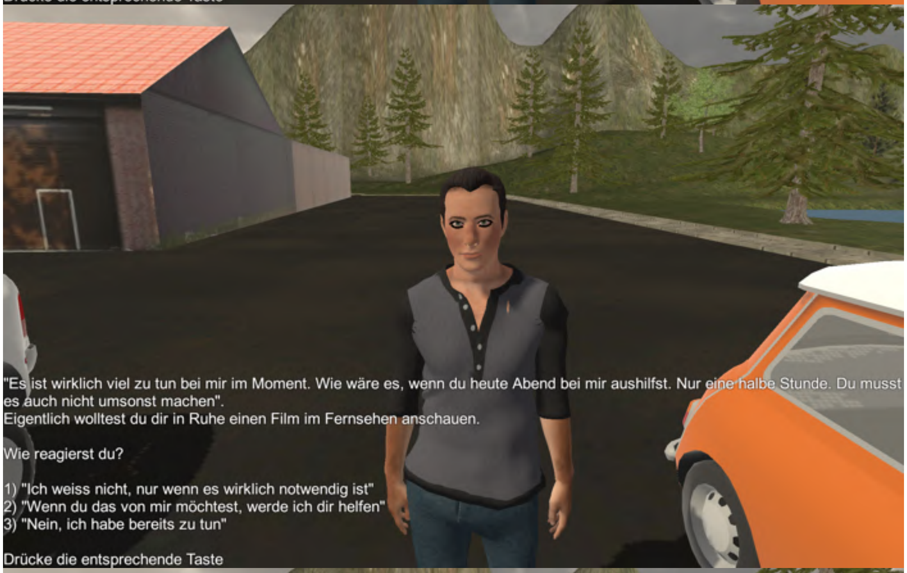
"Es ist wirklich viel zu tun bei mir im Moment. Wie wäre es, wenn du heute Abend bei mir aushilfst. Nur eine halbe Stunde. Du musst es auch nicht umsonst machen".
Eigentlich wolltest du dir in Ruhe einen Film im Fernsehen anschauen.
Wie reagierst du?
1) "Ich weiss nicht, nur wenn es wirklich notwendig ist"
2) "Wenn du das von mir möchtest, werde ich dir helfen"
3) "Nein, ich habe bereits zu tun"
Drücke die entsprechende Taste

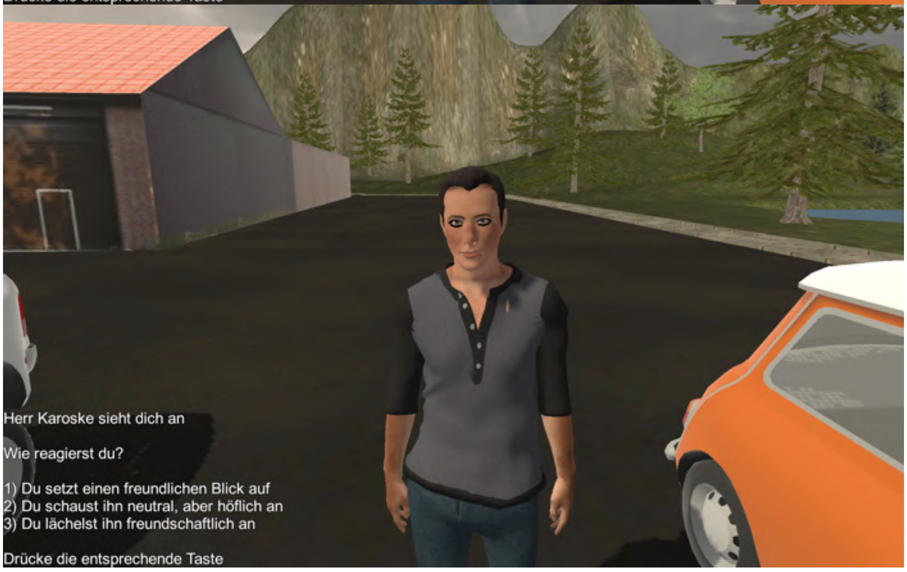
Herr Karoske sieht dich an
Wie reagierst du?
1) Du setzt einen freundlichen Blick auf
2) Du schaust ihn neutral, aber höflich an
3) Du lächelst ihn freundschaftlich an
Drücke die entsprechende Taste

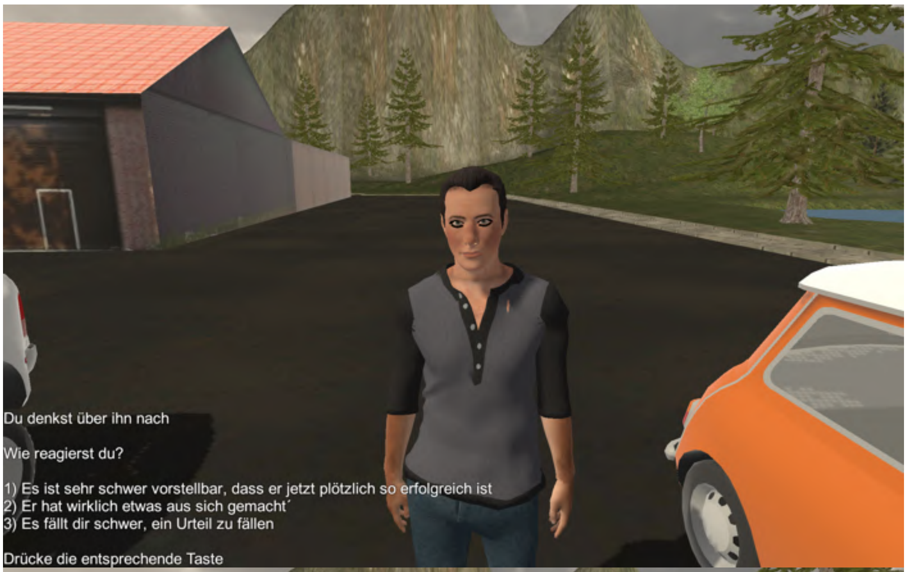
Du denkst über ihn nach
Wie reagierst du?
1) Es ist sehr schwer vorstellbar, dass er jetzt plötzlich so erfolgreich ist
2) Er hat wirklich etwas aus sich gemacht´
3) Es fällt dir schwer, ein Urteil zu fällen
Drücke die entsprechende Taste

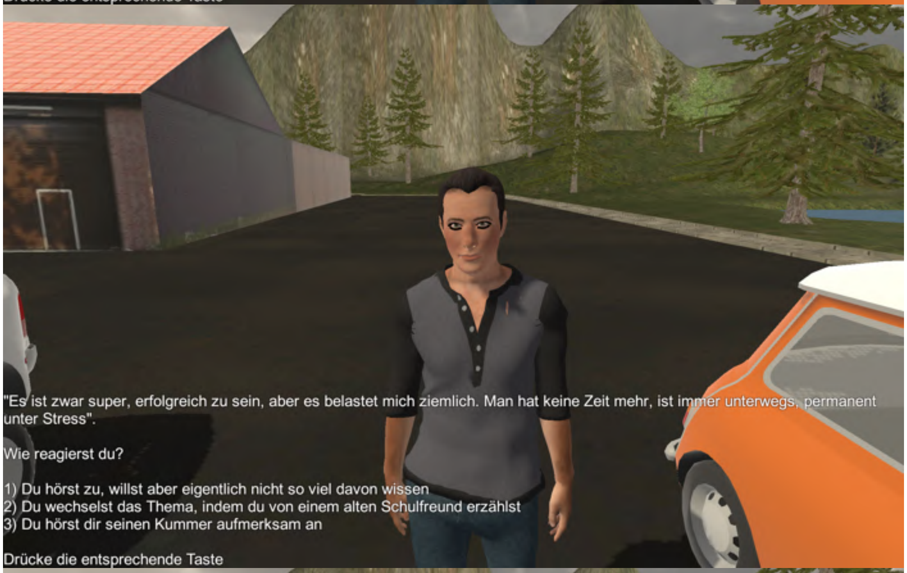
"Es ist zwar super, erfolgreich zu sein, aber es belastet mich ziemlich. Man hat keine Zeit mehr, ist immer unterwegs, permanent unter Stress".
Wie reagierst du?
1) Du hörst zu, willst aber eigentlich nicht so viel davon wissen
2) Du wechselst das Thema, indem du von einem alten Schulfreund erzählst
3) Du hörst dir seinen Kummer aufmerksam an
Drücke die entsprechende Taste

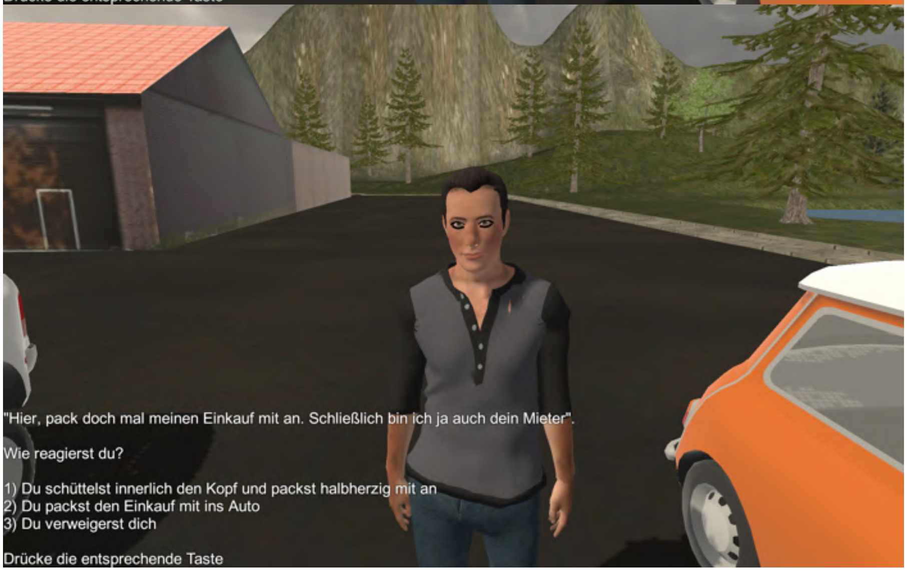
"Hier, pack doch mal meinen Einkauf mit an. Schließlich bin ich ja auch dein Mieter".
Wie reagierst du?
1) Du schüttelst innerlich den Kopf und packst halbherzig mit an
2) Du packst den Einkauf mit ins Auto
3) Du verweigerst dich
Drücke die entsprechende Taste

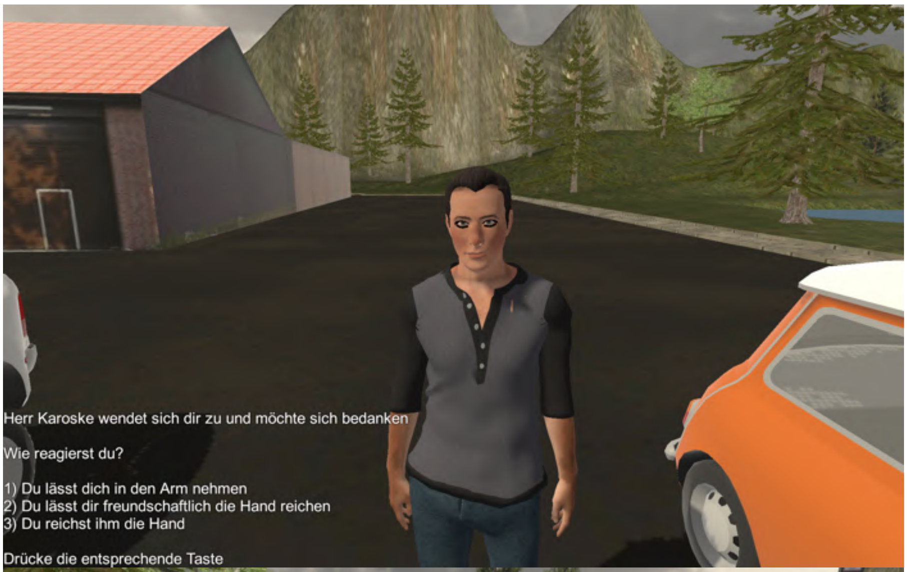
Herr Karoske wendet sich dir zu und möchte sich bedanken
Wie reagierst du?
1) Du lässt dich in den Arm nehmen
2) Du lässt dir freundschaftlich die Hand reichen
3) Du reichst ihm die Hand
Drücke die entsprechende Taste

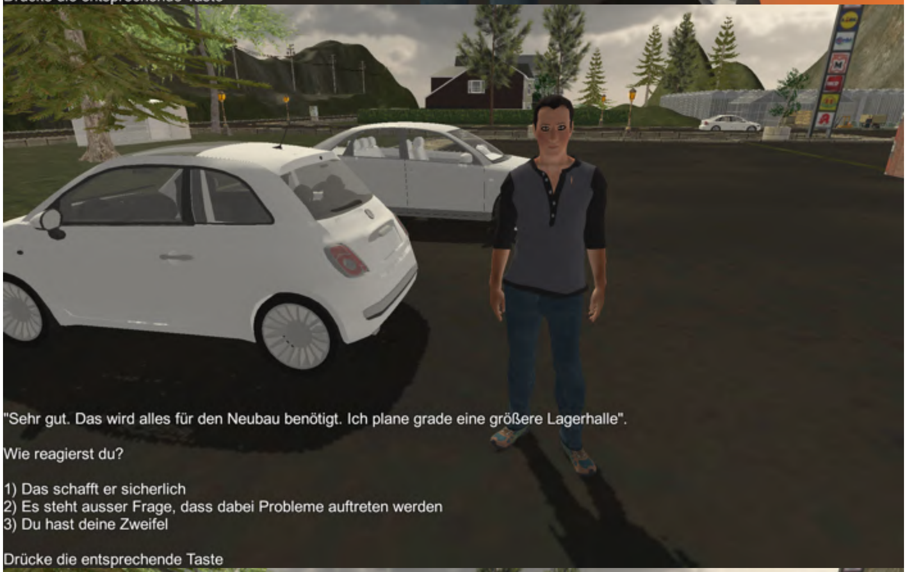
"Sehr gut. Das wird alles für den Neubau benötigt. Ich plane grade eine größere Lagerhalle".
Wie reagierst du?
1) Das schafft er sicherlich
2) Es steht ausser Frage, dass dabei Probleme auftreten werden
3) Du hast deine Zweifel
Drücke die entsprechende Taste

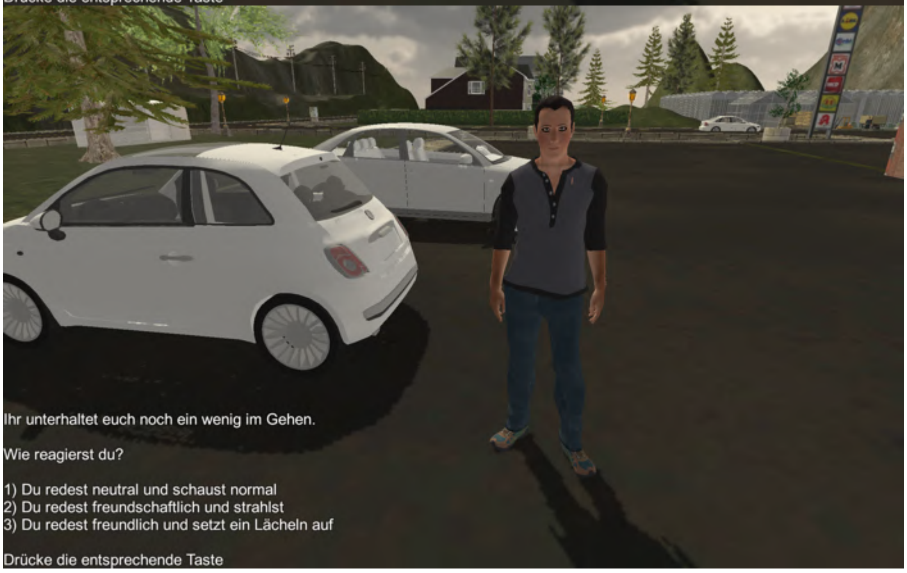
Ihr unterhaltet euch noch ein wenig im Gehen.
Wie reagierst du?
1) Du redest neutral und schaust normal
2) Du redest freundschaftlich und strahlst
3) Du redest freundlich und setzt ein Lächeln auf
Drücke die entsprechende Taste

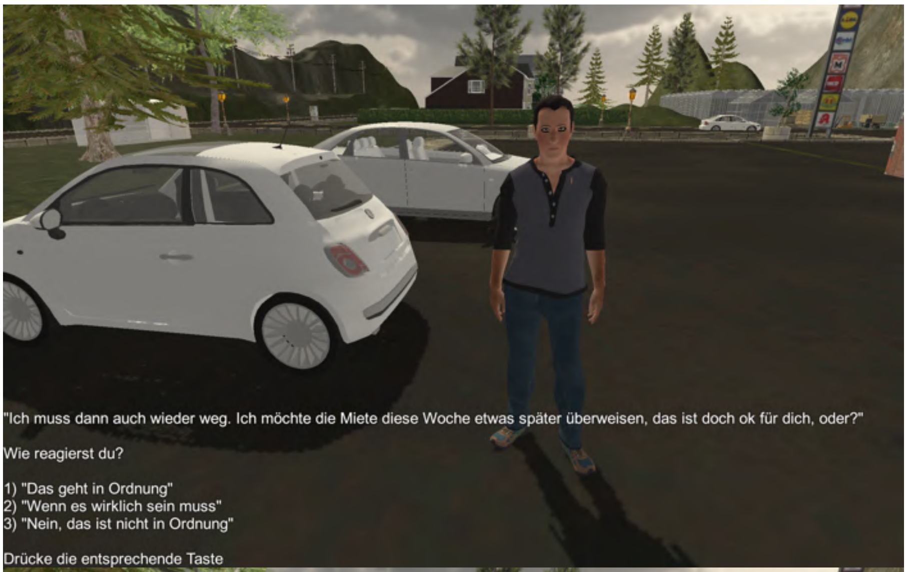
"Ich muss dann auch wieder weg. Ich möchte die Miete diese Woche etwas später überweisen, das ist doch ok für dich, oder?"
Wie reagierst du?
1) "Das geht in Ordnung"
2) "Wenn es wirklich sein muss"
3) "Nein, das ist nicht in Ordnung"
Drücke die entsprechende Taste

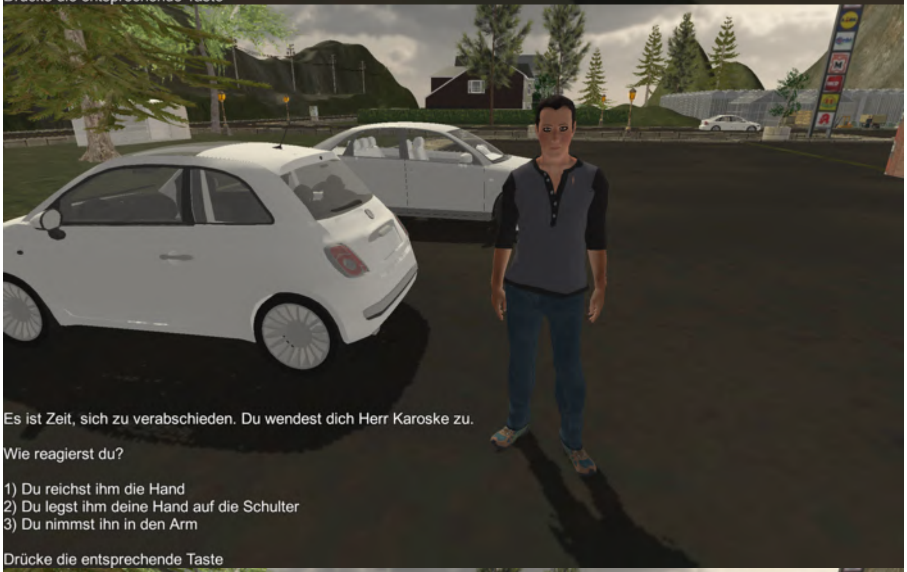
Es ist Zeit, sich zu verabschieden. Du wendest dich Herr Karoske zu.
Wie reagierst du?
1) Du reichst ihm die Hand
2) Du legst ihm deine Hand auf die Schulter
3) Du nimmst ihn in den Arm
Drücke die entsprechende Taste

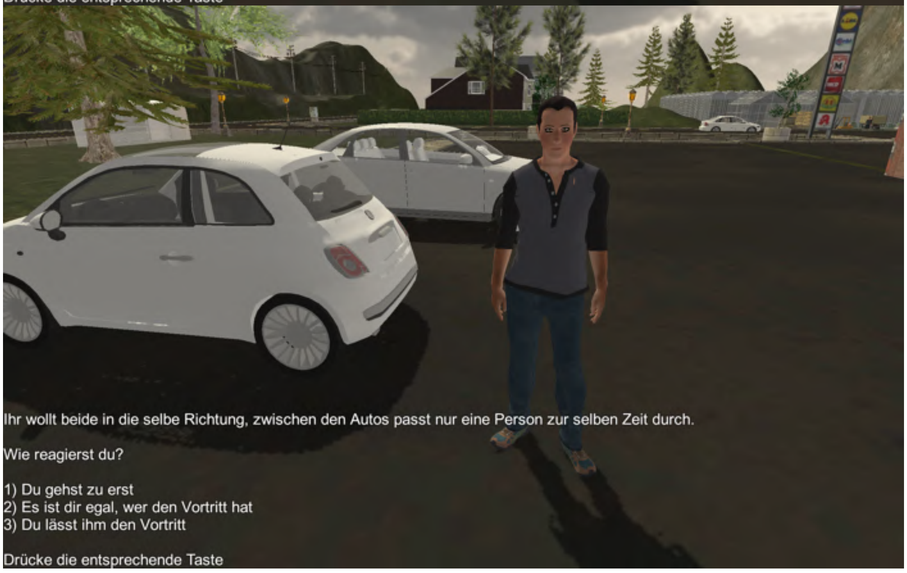
Ihr wollt beide in die selbe Richtung, zwischen den Autos passt nur eine Person zur selben Zeit durch.
Wie reagierst du?
1) Du gehst zu erst
2) Es ist dir egal, wer den Vortritt hat
3) Du lässt ihm den Vortritt
Drücke die entsprechende Taste
